# Design and Synthesis of BLU-654, a Potent and Selective Mutant KIT V654A Inhibitor for the Treatment of Imatinib-Resistant GIST

**DOI:** 10.1021/acs.jmedchem.5c03554

**Published:** 2026-03-10

**Authors:** Ludivine Moine, Wei Hu, Alison Davis, Emanuele Perola, Jian Guo, Kevin Barvian, Yeon Sook Choi, Alexandra Grassian, Joseph L. Kim, Omar K. Ahmad, Thomas A. Dineen

**Affiliations:** 376392 Blueprint Medicines Corporation, 45 Sidney St., Cambridge, Massachusetts 02139, United States

## Abstract

Gastrointestinal stromal tumor (GIST) is the most common type of sarcoma of the gastrointestinal tract, with approximately 5000 new cases annually in the USA. Approximately 80% of GIST cases are driven by activating mutations in *KIT* in exon 9 or 11. Resistance to present therapies like imatinib often arises from secondary KIT mutations, especially V654A (exon 13), which is the most frequent resistance mutation. Tyrosine kinase inhibitors (TKIs) currently approved for GIST can cause dose-limiting side effects due to off-target inhibition of other kinases. Herein, we report the discovery and optimization of BLU-654 (compound **18**), a highly potent and kinome-sparing KIT V654A inhibitor. Preclinical efficacy studies demonstrated its prolonged antitumor activity in a KIT V654A cell-derived xenograft mouse model. BLU-654 offers a potent and selective profile suitable for combination therapy for *KIT-*mutant GIST patients.

## Graphical abstract

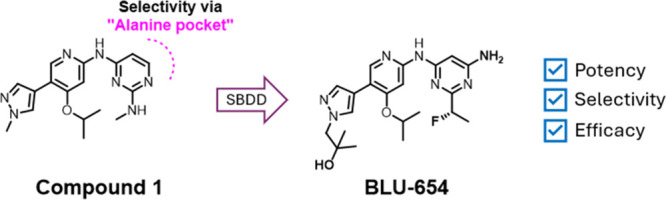

## Introduction

Gastrointestinal stromal tumor (GIST) is a sarcoma of the gastrointestinal tract, with approximately 5–6000 patients diagnosed per year in the USA. This corresponds to an annual incidence of 0.70 per 100,000 people.^1^ The majority of GIST cases, approximately 80–85%, are characterized by primary activating mutations in the *KIT* gene at exons 9 and 11 respectively, corresponding to the extracellular region or juxtamembrane domains in the KIT protein (Figure 1).^2^

**Figure 1:**
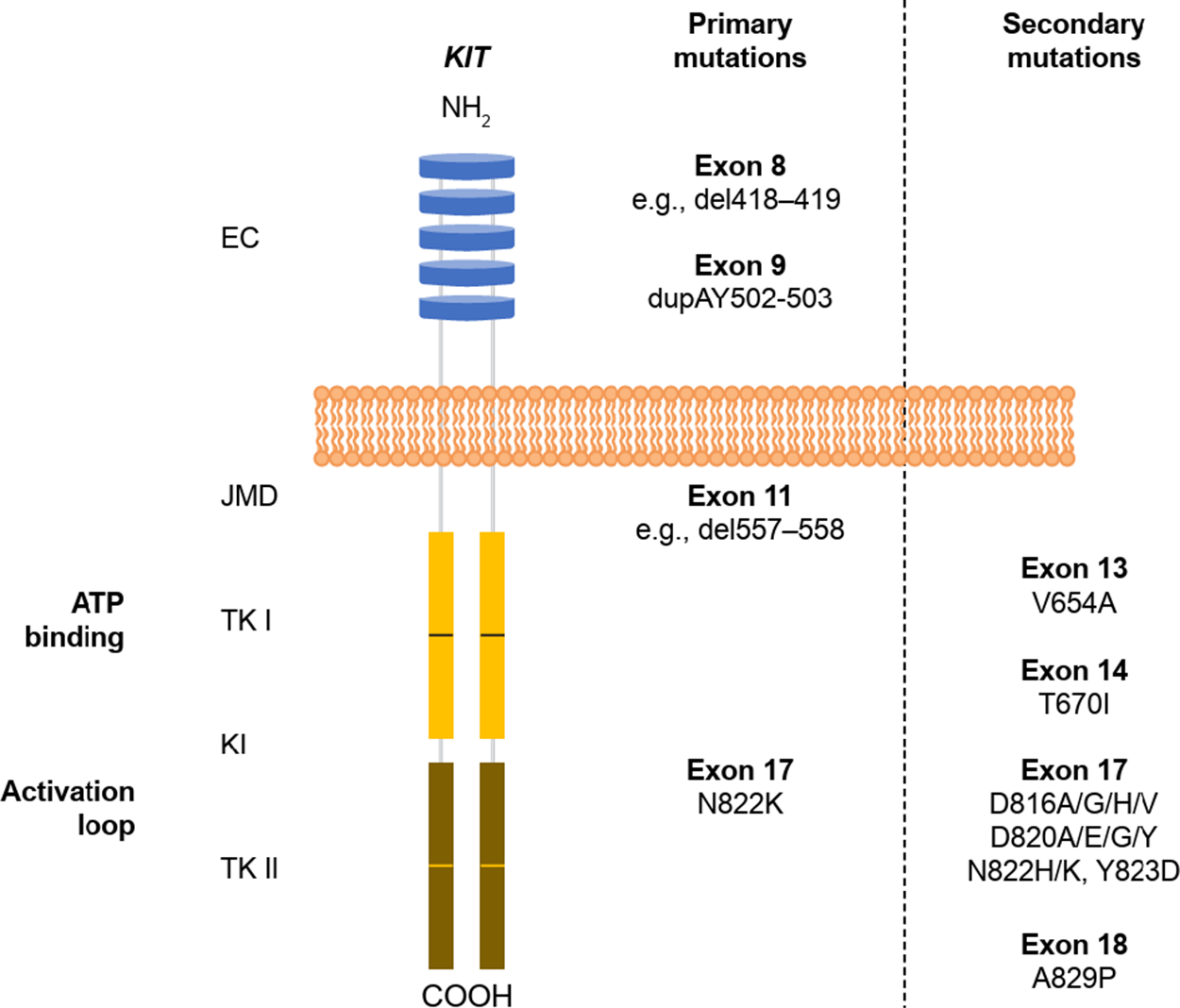
Key secondary mutations drive imatinib resistance to GIST treatment.^23^ Figure adapted from Masucci, M. T., et al., *Int. J. Mol. Sci.*, **2023**, 24, 6026. ATP, adenosine triphosphate; EC, extracellular domain; GIST, gastrointestinal stromal tumor JMD, juxtamembrane domain; TK I, tyrosine kinase domain I; and TK II, tyrosine kinase domain II.

KIT—a member of the receptor tyrosine kinase family—is activated by stem cell factor (SCF), leading to dimerization, kinase autophosphorylation, and subsequent downstream signaling. The mutations present in GISTs result in ligand-independent activation of the KIT kinase domain and subsequent constitutive signaling through proliferative and antiapoptotic pathways.^3^

For patients with metastatic GIST, frontline therapy with the tyrosine kinase inhibitor (TKI) imatinib,^4–6^ has had a revolutionary impact on patients given its effectiveness, with response rates of approximately 51–54% and median progression-free survival (PFS) of 19–23 months in a molecularly unselected population.^7–9^ *KIT* mutant patients who progress on imatinib frequently harbor one or more secondary resistance mutations within exons 13, 14, 17, or 18 (Figure 1) which are observed in 40% to over 80% of cases.^7–12^ New treatment line therapies such as sunitinib, regorafenib, and ripretinib (Figure 2A) displayed response rates of less than 10% and median PFS of approximately 5–6 months suggesting limited coverage of those mutations.^13–18^ Furthermore, these treatments have been associated with side-effects that can be linked to inhibition off-target kinases. Additional KIT inhibitors, such as avapritinib^19^ and bezuclastinib^20^ have been developed to target exon 17 resistance mutations more effectively. Recently, IDRX-42 (formerly M4205), a selective KIT inhibitor, was described to cover exon 11, 13, and 17 mutations, and is currently in phase 3 clinical trials.^21,22^

**Figure 2:**
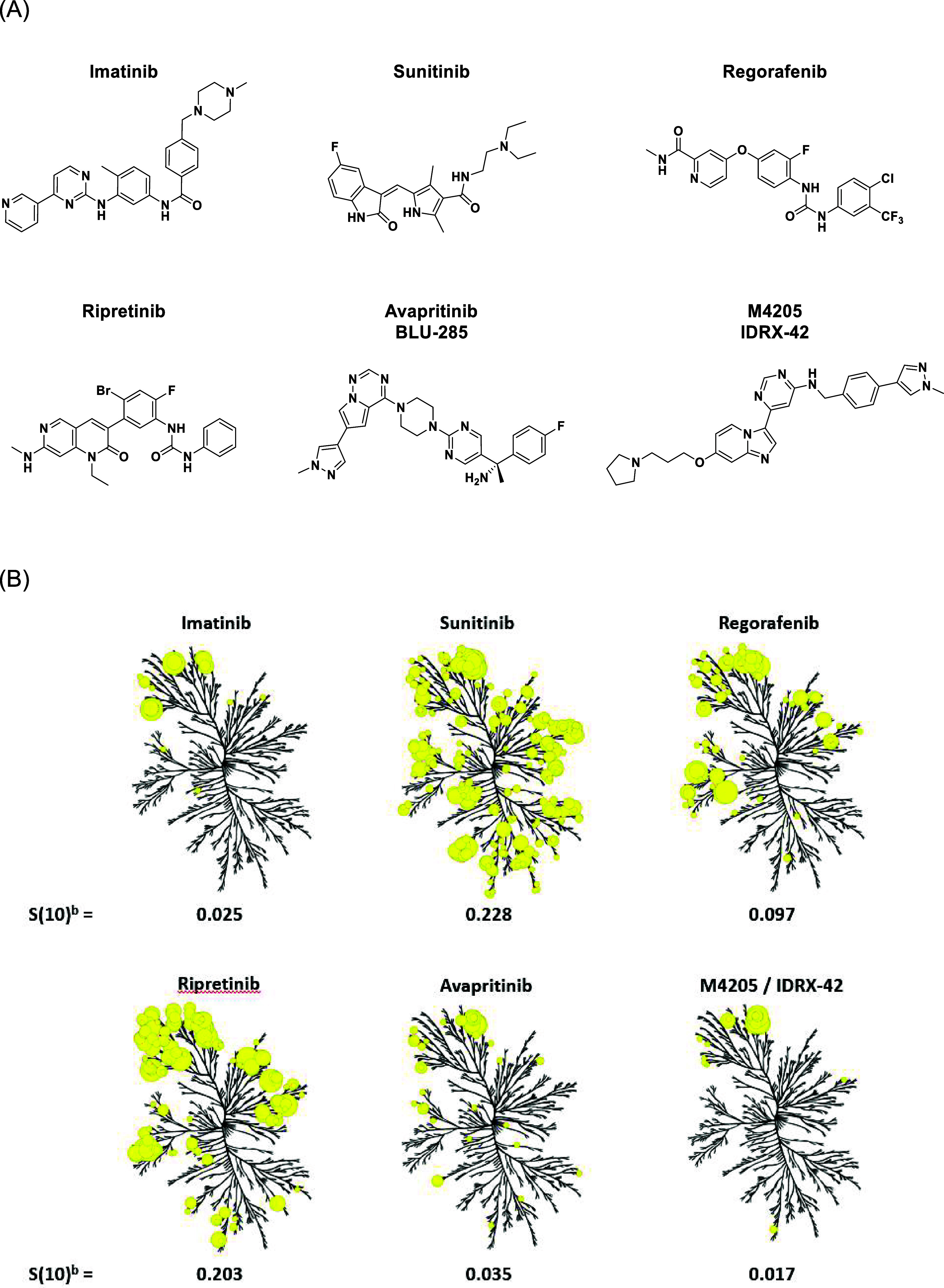
(A) Known KIT inhibitors structures and (B) their corresponding kinome tree^a^ and kinome score S(10)^b^. ^a^Kinome illustration reproduced courtesy of Cell Signaling Technology, Inc. (CST) (www.cellsignal.com ). The foregoing Web site is maintained by CST, and Blueprint Medicines Corporation is not responsible for its content; ^b^DiscoverX’s KINOMEscan selectivity profiling at 3 μM, *S*(10) = (number of nonmutant kinases with %Ctrl < 10)/(number of nonmutant kinases tested).

Among these resistance mutations, the KIT exon 13 V654A, located in the adenosine triphosphate (ATP)-binding site, is sensitive to sunitinib, but not other approved GIST therapies.^24,25^ Unfortunately, sunitinib has a high rate of dose-limiting side effects due to off-target kinase inhibition of wild-type KIT (WT KIT), platelet-derived growth factor receptors (PDGFRs), and vascular endothelial growth factor receptors (VEGFR), among others, as depicted by its kinome profile (Figure 2B).^26^

The heterogeneity of KIT secondary mutations limits the effectiveness of monotherapy. Combination strategies using TKIs with complementary activity, such as pairing a selective KIT V654A inhibitor with agents targeting exon 9 and 11 mutations, may improve outcomes in patients who have relapsed on imatinib.^27^ Developing a KIT V654A inhibitor with high kinome selectivity, particularly sparing WT KIT and PDGFRs, could also avoid exacerbating related adverse effects. Such combination regimens have the potential to establish mutation-agnostic, second-line therapies that enhance response rates and durability and may ultimately become frontline treatment for diverse GIST patient populations.

Herein, we report the discovery of **BLU-654**: a potent, mutant-selective, kinome-sparing, and peripherally restricted KIT V654A inhibitor with favorable pharmacokinetics (PK) and pharmaceutical properties, potentially compatible as a combination therapy with other KIT inhibitors.

## Results and Discussion

Mutations in exon 11 of KIT, such as V560G, are a primary driver of GIST^28^ and elicit sensitivity to imatinib. To model imatinib resistance and identify inhibitors with activity against KIT V654A in the relevant genetic context, we utilized the human mast cell line HMC1.1, which harbors a heterozygous KIT exon 11 V560G mutation. Analogous to the clinically relevant association of exon 11 and exon 13 mutations often observed in imatinib-resistant patients, we used an in vitro resistance screen with imatinib to generate a resistant clone with the V654A mutation in cis with the ex11 mutation, which is later referred to as HMC1.1 11/13 cell line.

Our primary objective was to identify chemical matter showcasing inhibition of mutant KIT autophosphorylation (pKIT 11/13) while demonstrating selectivity over WT KIT,^29^ PDGFRβ^30^ and the rest of the kinome. To evaluate kinome selectivity, we employed the DiscoverX KINOMEScan platform^31^ which provides a kinome score-referred to as *S*(10). *S*(10) represents the ratio between the number of nonmutant kinases with %Ctrl <10 at the tested inhibitor concentration and the total number of nonmutant kinases in the testing panel, where %Ctrl represents the percentage of residual activity for a specific kinase in the presence of inhibitor. The *S*(10) values reported in this study were determined at inhibitor concentration of 3 μM. We established a threshold of *S*(10) < 0.05 to indicate minimal off-target kinase binding.

Leveraging Blueprint Medicines’ proprietary library^32^ of agnostic kinase inhibitors profiled against >400 human kinases using the KINOMEscan platform^31^ enabled the rapid identification of compound **1** as a lead. It demonstrated a 7.7 nM cellular potency in pKIT 11/13 with 8-fold selectivity over pKIT WT and 20-fold selectivity over pPDGFRβ (Figure 3). However, **1** exhibited a high *S*(10) of 0.23, similar to the inhibitors sunitinib (*S*(10) = 0.228) and ripretinib (*S*(10) = 0.203), and markedly less selectivity than imatinib (*S*(10) = 0.025; Figure 1b). As a result, we prioritized maintaining potency while substantially improving kinome selectivity.

**Figure 3:**
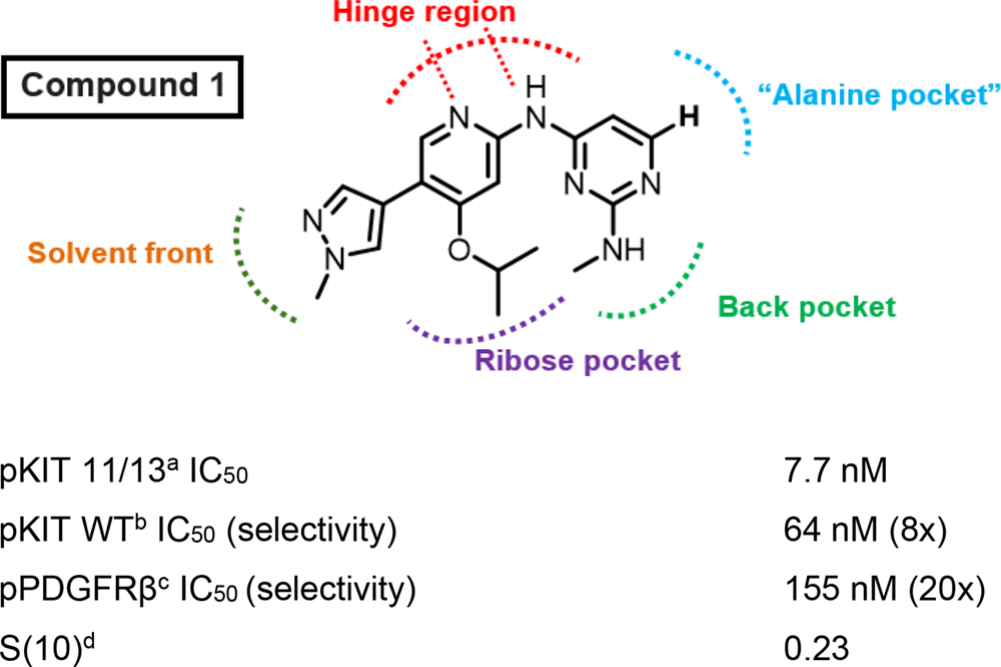
Identification of Hit 1 and its properties. ^a^HMC1.1 11/13 cells. ^b^Inhibition of wild-type KIT (WT KIT) autophosphorylation in the M07E (human factor-dependent myeloid leukemia) cell line following SCF stimulation. ^c^SW569 cells. ^d^DiscoverX KINOMEScan selectivity profiling at 3 μM, *S*(10) = (number of nonmutant kinases with %Ctrl <10)/(number of nonmutant kinases tested).

To gain a better understanding of how to further enhance selectivity over WT KIT and the broader kinome, we collected an X-ray structure of **1** bound to V654A mutated KIT protein. Compound **1** exhibits a two-point interaction with the hinge region: the central pyridine nitrogen forms a hydrogen bond with the backbone N–H of Cys673, and the aminopyridine N–H forms a hydrogen bond with the backbone carbonyl oxygen of Glu671 (Figure 4). The pyrazole is oriented toward the solvent front, while the ether alkyl chain extends into the ATP ribose binding region. This region will be later referred to as the “ribose pocket,” while substituents extending into this region will be referred to as “ribose groups.” The pyrimidine is deeply embedded in the ATP-binding site and is hydrogen bonded to the amino group of Lys623 through its N1 nitrogen. Additionally, the 2-methylamino NH forms a hydrogen bond with the backbone carbonyl oxygen of Asp810.

**Figure 4:**
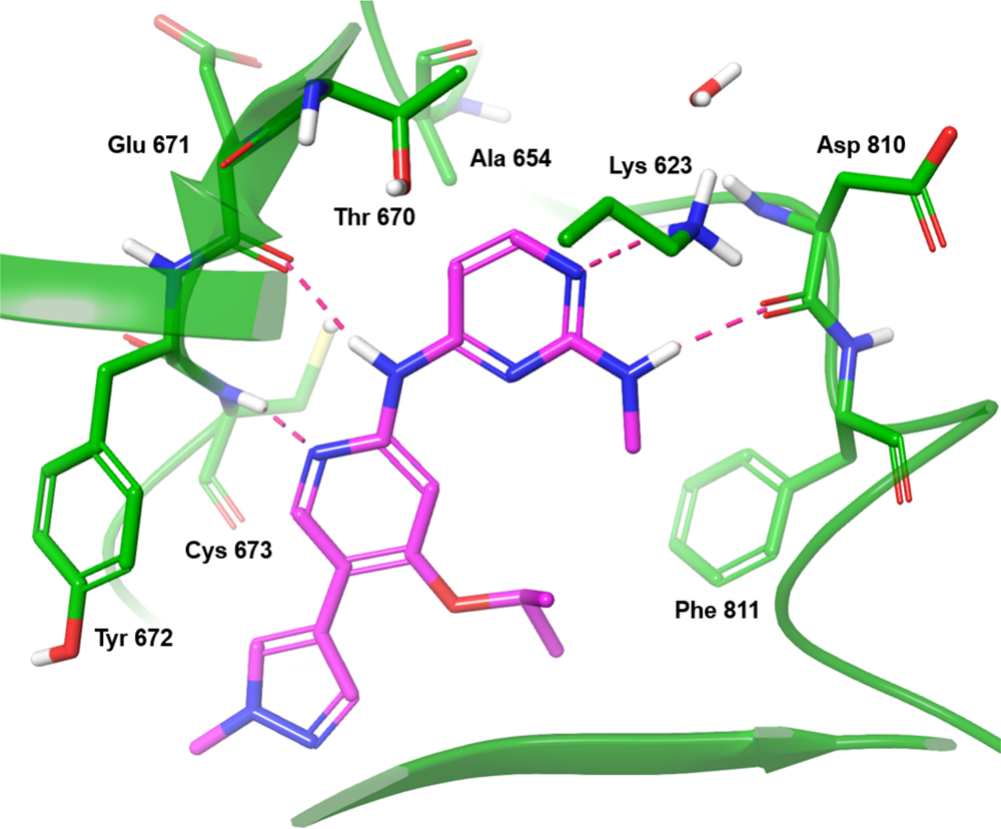
X-ray structure of Compound **1** bound to V654A mutated KIT protein. The positions of hydrogen atoms cannot be defined based on X-ray data at the resolution obtained for this structure. Hydrogen atoms were added and energy minimized using the Protein Preparation Workflow within Maestro (Schrodinger, Inc.). Polar hydrogens thus generated are included in this figure.

Interestingly, the mutation in KIT at position 654 from a larger residue (valine) to a smaller one (alanine) results in a new and uncommon pocket located deep in the ATP-binding site. This region of the kinase pocket is typically conserved across the kinome and Moslin et al. noted that only a few kinases have an alanine at this position, which is typically occupied by larger residues.^33^ We sought to leverage this rare “alanine pocket” by introducing a small substituent at the C6 position of the pyrimidine to further improve the selectivity windows over WT KIT, PDGFRα/β and other off-target kinases.

The structure–activity relationship (SAR) exploration of the pyrimidine 6 position provided evidence supporting our hypothesis that small groups are indeed tolerated (Table 1). The best substitutions provided an affinity boost against the V654A mutant while reducing kinome promiscuity and demonstrated excellent selectivity over WT KIT and PDGFRα/β off-targets (Table 1). A chlorine substituent as in **2**, offered a significant decrease in off-target kinase activity, as reflected by an improved *S*(10), while maintaining good potency on pKIT 11/13 (11 nM) and a 10-fold improvement in selectivity over WT KIT (78× vs 8× for **1**).

**Table 1:**
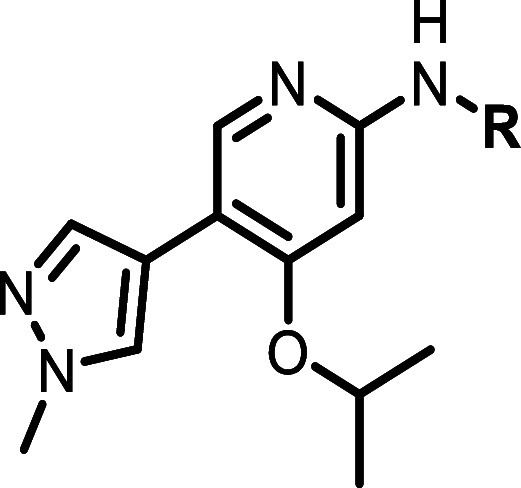

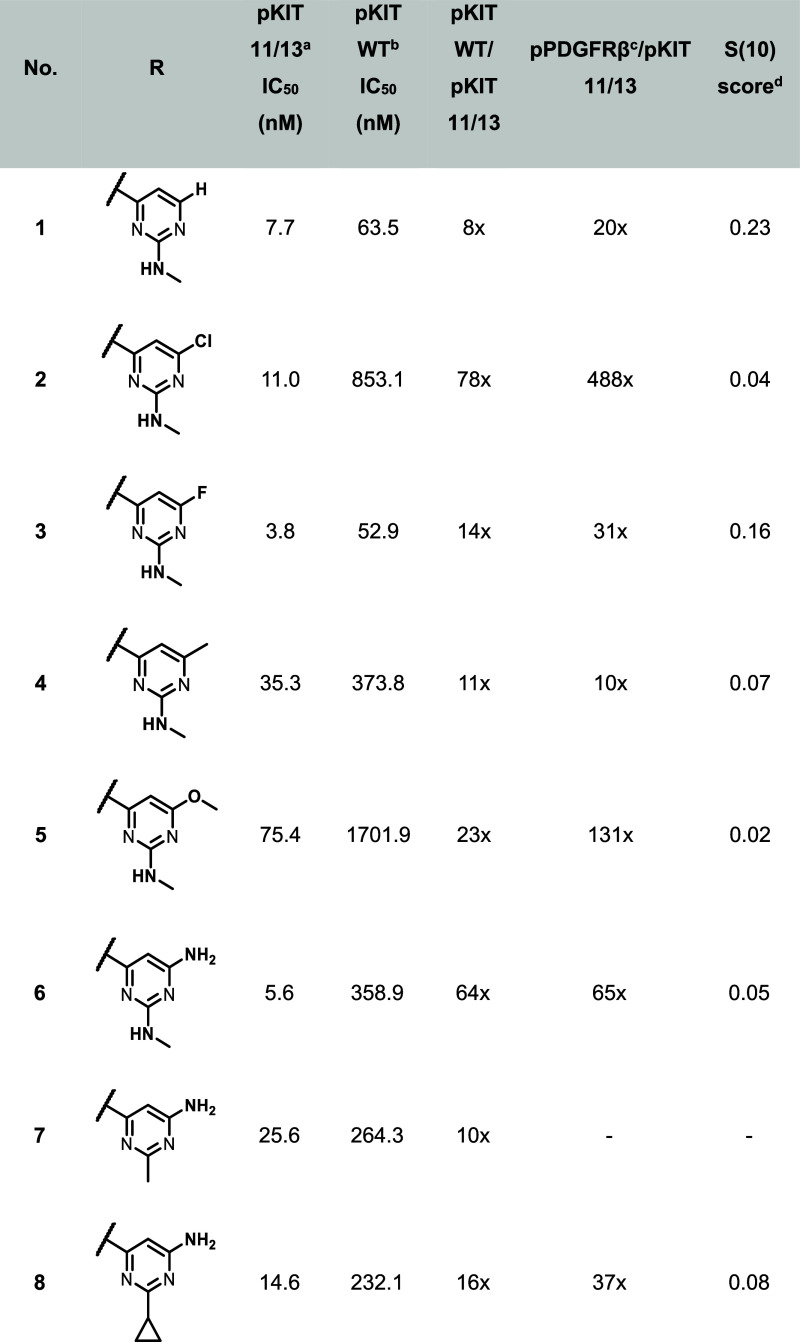
SAR Table of 2-Substituted Pyridine^a^

a Number of experiments for a given data point and standard error of the mean (SEM) available in the Supporting Information.

b HMC1.1 11/13 cell line.

c M-07e cell line.

d SW569 cell line.

e DiscoverX KINOMEScan selectivity profiling at 3 μM, *S*(10) = (number of nonmutant kinases with %Ctrl <10)/(number of nonmutant kinases tested); *n* = 1.

The fluorine substitution (**3**) was unable to achieve the same level of selectivity (14-fold over WT KIT and *S*(10) = 0.16), most likely due to its smaller size. While methyl and methoxy groups (entries 4 and 5, respectively) were able to reduce off-target kinase activity, they were less tolerated by the KIT V654A mutant, resulting in a 5-fold to 10-fold loss in activity. Alternatively, we were pleased to identify an anilino group (**6**) as another fruitful replacement which demonstrated single-digit nanomolar potency on pKIT 11/13 and 64-fold selectivity over WT KIT. Larger substitutions were also explored but were not well tolerated, leading to a significant loss of potency.

Based on these results, we selected chloro and amino groups (**2** and **6**, respectively) as the best alanine pocket substituents and further investigated substitutions at the pyrimidine 2-position to improve potency and absorption, distribution, metabolism, and excretion (ADME) properties. Simple alkyl chains such as methyl and cyclopropyl (**7** and **8**) were acceptable but had much lower selectivity windows over WT KIT due to erosion in potency on pKIT 11/13.

These findings highlighted the importance of substitutions at the pyrimidine 6-position in modulating both potency and selectivity, and they provided a solid foundation for further efforts aimed at enhancing the therapeutic profile of these compounds.

After identifying the most fruitful pyrimidine substituents on the right-hand side to drive potency and selectivity, we embarked on a scaffold-hopping exercise in search of additional hinge binders with diverse properties (Table 2). To that end, we envisioned tying the ribose group and solvent front motif together as a bicyclic system, such as a 2-aminonaphthyridine core (**9**, Table 2). Initially, we were pleased to observe that **9** maintained comparable potency and selectivity over WT KIT to **8** and had reduced kinome promiscuity compared with the corresponding 2-aminopyridyl analog **3** (*S*(10) = 0.03 vs 0.16). However, **9** was hindered by high metabolic instability in human liver microsomes and poor solubility.

**Table 2:**
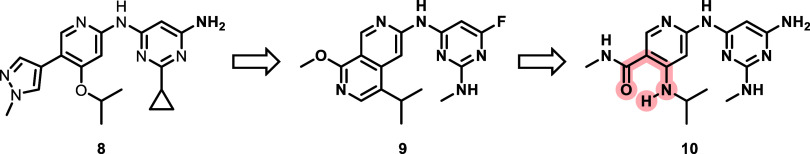
Core Swaps^a^

	**compound**		
	**8**	**9**	**10**
pKIT 11/13^b^ IC_50_ (nM)	14.6	12.4	13.0
pKIT WT^c^ IC_50_ (nM)	232.1	236.0	649.8
pKIT WT/pKIT 11/13	16×	19×	50×
pPDGFRβ^d^/pKIT 11/13	37×	55×	463×
human LM Cl_int_ ^e^ (mL min^–1^ kg^–1^)	16.2	32.4	5.1
FaSSIF (μM)	77	0.5	56
*S*(10) score^f^	0.08	0.03	0.02

a Number of experiments for a given data point and standard error of the mean (SEM) available in the Supporting Information.

b HMC1.1 11/13 cell line.

c M-07e cell line.

d SW569 cell line.

e Intrinsic clearance obtained from isolated human microsomes; *n* = 1.

f DiscoverX KINOMEScan selectivity profiling at 3 μM, *S*(10) = (number of nonmutant kinases with %Ctrl <10)/(number of nonmutant kinases tested); *n* = 1.

We then proposed a picolinamide core bearing a secondary amine-linked ribose group, which would provide intramolecular hydrogen bonding between the N–H at C4 and the carbonyl of the amide, mimicking the planarity of the naphthyridine. This new core, referred to as series B, recapitulated the cellular potencies of **9** while reducing microsomal turnover by 6-fold and boosting solubility by 10-fold.

We hypothesized that mobility in the P-loop could be a key differentiating factor for achieving our desired mutant and wild-type potency profiles. To explore this, we surveyed ribose groups with varying degrees of rigidity and solvent front groups via parallel syntheses to identify new potency and selectivity handles, with the potential to further modulate physicochemical properties for improved PK. Unfortunately, modification of the C4 substituent on the pyridine core to extend into the ribose pocket was largely unsuccessful in achieving our objectives. Similarly, only a few amide examples demonstrated adequate potency and provided no additional benefits. Consequently, these results limited further investigation in those regions.

We ultimately combined the optimally substituted picolinamide with various pyrimidine-bearing alternative back pocket groups resulting in the discovery of **11** (Figure 5). While this compound displayed a much-improved apparent permeability and lower efflux in Caco-2 cells, it suffered from high liver microsomes turnover and low stability in hepatocytes across species, with these effects being even more pronounced in rat. In PK experiments, despite compound **11** showcasing good bioavailability in rat (69%), we observed moderate to high clearance and short half-lives in rat and monkeys, indicating that further optimization would be necessary to advance this series.

**Figure 5:**
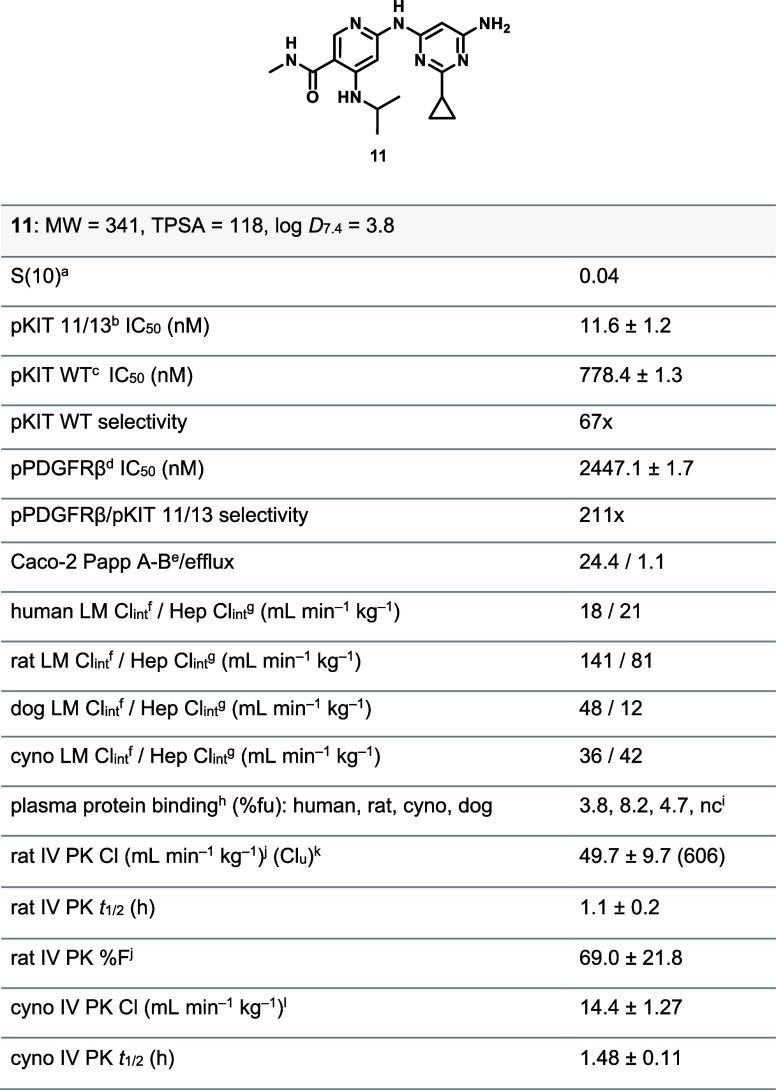
Structure and properties of Lead 11. Number of experiments for a given data point and standard error of the mean (SEM) available in the Supporting Information. ^a^DiscoverX KINOMEScan selectivity profiling at 3 μM, *S*(10) = (number of nonmutant kinases with %Ctrl <10)/(number of nonmutant kinases tested); *n* = 1. ^b^HMC1.1 11/13 cell line. ^c^M-07e cell line. ^d^SW569 cell line. ^e^10^–6^ cm·s^–1^. ^f^Intrinsic clearance obtained from isolated human, rat, dog, or monkey microsomes. ^g^Intrinsic clearance obtained from isolated human, rat, dog or monkey hepatocyte cells. ^h^Plasma–protein binding was determined by Rapid Equilibrium Dialysis (RED). ^i^Not collected. ^j^Male Sprague-Dawley rats (*n* = 3); IV dose = 0.5 mg/kg and PO dose = 5 mg/kg using 10% Solutol HS15 and 80%(20%HP-β-CD in saline). ^k^Cl_u_: unbound in vivo clearance (in vivo rat clearance/free fraction in rat), free fraction calculated from plasma protein binding determined by Rapid Equilibrium Dialysis (RED). ^l^Cynomolgus monkey (*n* = 3), IV dose = 0.5 mpk using 5% DMSO+5% Kolliphor HS15+90% saline and PO dosing was not performed.

To overcome the limitations observed with the amide vector and to highlight additional potential interactions that could increase affinity, we collected an X-ray structure of **11** bound to the autoinhibited KIT V654A mutant (Figure 6). The nicotinamide core retains interactions with the hinge region similar to those observed with **1**. The solvent front amide is twisted approximately 30 degrees relative to the pyridyl core, likely influenced by a hydrogen bond to the carbonyl oxygen of the hinge residue Cys673.

**Figure 6:**
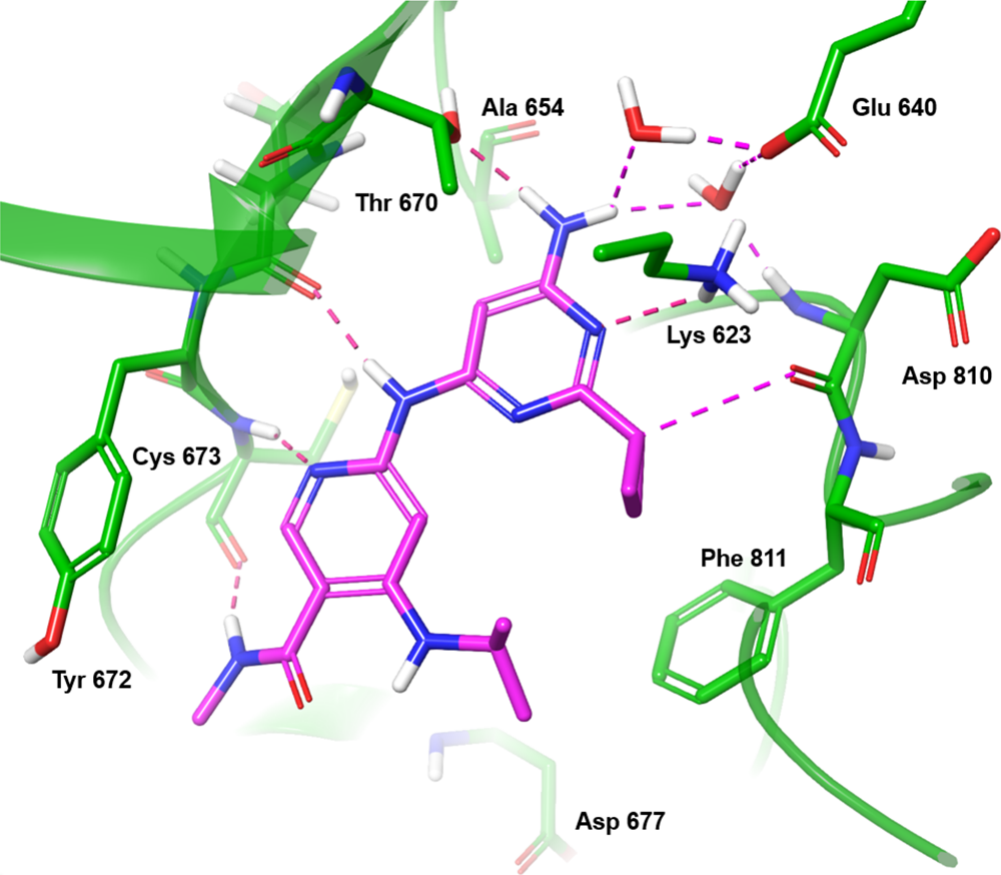
X-ray structure of Lead 11 bound to V654A mutant. See caption to Figure 4.

The structure corroborated the steep SAR observed when extending the amide, as adding an extra methyl group impinged upon either the residue before the hinge (Tyr672) or the hinge backbone, depending on its orientation (up or down). Notably, the placement of the polar NH_2_ group from the 2-aminopyrimidine into the alanine pocket caused the threonine gatekeeper residue to rotate, facilitating a hydrogen bond between the amino group and the side chain hydroxyl group. The amino group was also stabilized by water-mediated interactions with multiple surrounding residues, thus explaining why the NH_2_ group was tolerated.

Additionally, the methine hydrogen of the cyclopropyl group is positioned within weak hydrogen bonding distance to the Asp810 mainchain carbonyl of the DFG (aspartate-phenylalanine-glycine) loop. This structural insight provided a deeper understanding of the interactions at play and guided further efforts to improve activity.

Recognizing the potential interplay between the ribose group and the pyrimidine substituents in impacting selectivity, we revisited the ribose vector. Our objective was to engage the Asp677 residue in the ribose pocket to enhance activity against the V654A KIT mutant. Consequently, we focused on small and rigid ether groups. Unfortunately, this approach generally resulted in a loss of potency. This indicated that expanding into this space was not beneficial, a finding that could be the result of limited flexibility in this region.

Based on these observations, we aimed to replace the cyclopropyl motif with another group bearing a polarized hydrogen to further engage the amide backbone of Asp810. We identified a difluoromethyl group as a suitable alternative, yielding **12** (Table 3). This modification resulted in a 3-fold increase in potency and a reduction in rat clearance from 50 to 31 mL·min^–1^·kg^–1^. While good rat bioavailability was maintained, there was a significant attrition in the selectivity margin over WT KIT, which decreased from 67-fold to 7-fold.

**Table 3:**
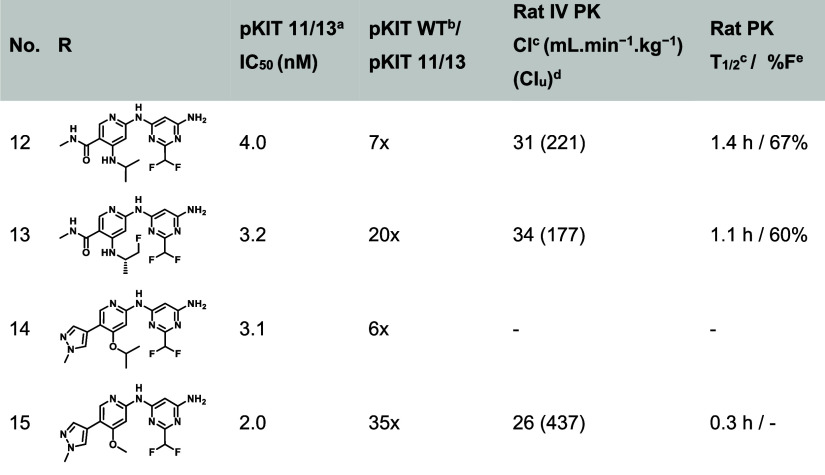
Strategies to Improve Selectivity over WT KIT and PK^a^

a Number of experiments for a given data point and standard error of the mean (SEM) available in the Supporting Information.

b HMC1.1 11/13 cell line.

c M-07e cell line.

d Male Sprague-Dawley rats (*n* = 3) were dosed at 1 mg/kg IV using the following formulation: solution of 10% DMSO, 10% solutol HS15, 80–“20% HP-β-CD in saline”.

e Clu: unbound in vivo clearance (in vivo rat clearance/free fraction in rat), free fraction calculated from plasma protein binding determined by Rapid Equilibrium Dialysis (RED).

f Male Sprague-Dawley rats (*n* = 3) were dosed at 5 mg/kg PO dose using the same formulation as IV.

Notably, a subtle modification of the amino group at the C4 position of the pyridine by adding one fluorine atom (**13**) partially rescued the selectivity, improving it from 7-fold to 21-fold. Despite achieving a good balance of potency, selectivity over WT KIT, and intrinsic clearance with the nicotinamide core, the limited toleration of amide substitution, combined with a steep SAR overall, low volume of distribution (Vd), and short half-lives in preclinical species, hampered further progression of this series.

Given that pyrazole as the solvent front group (series A) could provide more latitude for PK optimization and that the recent 2-difluoromethylpyrimidine back pocket group displayed enhanced potency, we combined these elements in **14**. Encouragingly, this combination resulted in a 5-fold increase in potency compared with **8** (3 vs 15 nM, respectively) though accompanied by attrition of selectivity, which decreased from 16-fold to 6-fold. Interestingly, truncating the ether motif to a simple methoxy group as in **15** had a profound impact on WT KIT potency, restoring selectivity to a more acceptable range (35-fold). Unfortunately, this compound still suffered from a low half-life in rat, not exceeding an hour.

Based on the trends observed for the two series, we thought that the pyrazole substituted pyridine (series A) would be better suited to both modulate potency and improve suboptimal exposure which was limited by a poor half-life and high clearance in rat. Our approach was to reduce logD and clearance by incorporating more polar substituents in the solvent front region. First, we extensively explored pyrazole replacements (data not shown) which had limited benefits. We also conducted a comprehensive survey of various N-substituted pyrazoles, including the basic amine derivative **16** (Table 4). This compound demonstrated remarkable potency and exhibited a 163-fold selectivity over WT KIT with reduced log *D* (0.7 vs 2.5 for **15**). Additionally, it showed a significantly improved half-life of approximately 6 h. However, compound **16** was associated with elevated rat clearance and suboptimal permeability. Additional pyrazoles bearing amino groups were evaluated but could not achieve better clearance or half-life in comparison to **16** (data not shown). We then converted the amino group to an alcohol and masked some of the polarity with methyl groups, yielding the tertiary alcohol **17**. This modification resulted in a substantial improvement in permeability, increasing Caco-2 Papp from 0.9 × 10^–6^ cm·s^–1^ for **16** to 29 × 10^–6^ cm·s^–1^ for **17**. Moreover, **17** mitigated the clearance concerns, albeit with a modest reduction in half-life (approximately 2-fold), which can be attributed to a significantly lower Vd compared with the basic amine **16**.

**Table 4:**
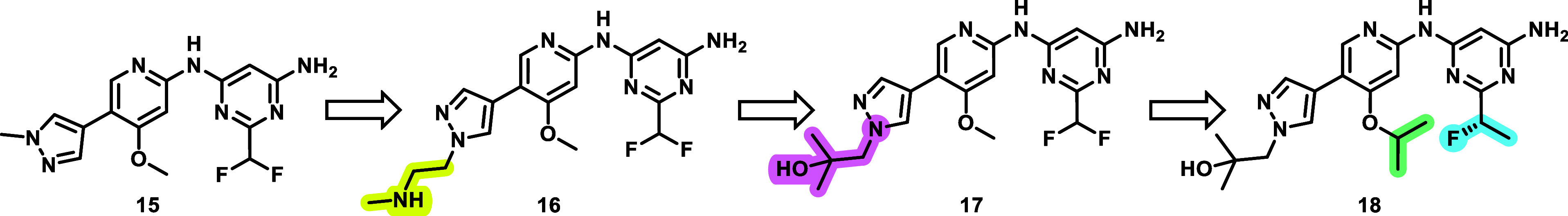
Lead Optimization to Improve the PK Profile^a^

	**compound**			
	**15**	**16**	**17**	**18**
logD_7.4_	2.5	0.7	2.4	3.2
pKIT 11/13^b^ IC_50_ (nM)	2.0	8.7	9.7	5.7
pKIT WT^c^/pKIT 11/13	35×	163×	33×	15×
Caco-2 *P*_app_ (10^–6^ cm/s)	41	0.9	29	26
human LM Cl_int_ ^d^	12.9	6.9	0^e^	0^e^
rat IV PK^e^ Cl^f^ (Cl_u_)^g^	26 (437)	58 (129)	16 (107)	15 (213)
rat IV PK *V*_ss_	0.7	18.4	2.3	3.0
rat PK *T*_1/2_ (h)	0.3	5.9	2.8	4.7

a Number of experiments for a given data point and standard error of the mean (SEM) available in the Supporting Information.

b HMC1.1 11/13 cell line.

c M-07e cell line.

d Intrinsic clearance obtained from isolated human microsomes (unit = mL·min^–1^·kg^–1^).

e Loss of compound was not observed in the assay run under the conditions described in the experimental section.

f Male Sprague-Dawley rats (*n* = 3) were dosed at 1 mg/kg IV using the following formulation: solution of 10% DMSO, 10% solutol HS15, 80–“20% HP-β-CD in saline” (in mL·min^–1^·kg^–1^).

g Cl_u_: unbound in vivo clearance (in vivo rat clearance/free fraction in rat), free fraction calculated from plasma protein binding determined by Rapid Equilibrium Dialysis (RED).

Further improvement of potency was achieved by expanding the ether moiety to an isopropoxy group and by substituting one of the fluorine atoms with a methyl group, affording the 1-fluoroethylpyrimidine group, favoring the *S* enantiomer as it was found significantly more potent than *R* in an earlier context (data not shown). The resulting **18** derivative displayed enhanced potency against KIT V654A, and maintained acceptable selectivity over WT KIT while demonstrating excellent intrinsic clearances and half-lives across preclinical species. Furthermore, **18** also maintained a substantial selectivity margin over PDGFRβ (219-fold) and exhibited a favorable kinome selectivity profile with an *S*(10) of 0.06 (Table 5 and Figure 7).

**Table 5:** In Vitro and In Vivo Profile of Compound **18** (BLU-654)^a^

**18**: MW = 429.5, TPSA = 124, log *D*_7.4_ = 3.3	
*S*(10)^b^	0.06
pKIT 11/13^c^ IC_50_ (nM)	5.7 ± 1.4
pKIT WT^d^ IC_50_ (nM)	82.7 ± 1.6
pKIT WT selectivity	15×
pPDGFRβ^e^ IC_50_ (nM)	1251.4 ± 1.5
pPDGFRβ/pKIT 11/13 selectivity	219×
Caco-2 *P*_app_ A-B^f^/efflux	25.6/1.3
FaSSIF at pH6.5 (μM)	79
human LM Cl_int_ ^g^/Hep Cl_int_ ^h^ (mL min^–1^ kg^–1^)	0/2
rat LM Cl_int_ ^g^/Hep Cl_int_ ^h^ (mL min^–1^ kg^–1^)	45775
dog LM Cl_int_ ^g^/Hep Cl_int_ ^h^ (mL min^–1^ kg^–1^)	0/71
cyno LM Cl_int_ ^g^/Hep Cl_int_ ^h^ (mL min^–1^ kg^–1^)	45738
plasma protein binding^i^ (%fu): human, rat, cyno, dog	8.4, 7.1, 13.9, 13.1
rat: Cl_p_ (mL min^–1^ kg^–1^), *t*_1/2_ (h), *V*_ss_ (L/kg), %F^j^	15.2, 4.7, 3.1, 123
cyno: Cl_p_ (mL min^–1^ kg^–1^), *t*_1/2_ (h), *V*_ss_ (L/kg), %F^k^	4.2, 2.9, 1.0
dog: Cl_p_ (mL min^–1^ kg^–1^), *t*_1/2_ (h), *V*_ss_ (L/kg), %F^l^	9.0, 5.3, 3.7, 94.8

a Number of experiments for a given data point and standard error of the mean (SEM) available in the Supporting Information.

b DiscoverX KINOMEScan selectivity profiling at 3 μM, *S*(10) = (number of nonmutant kinases with %Ctrl <10)/(number of nonmutant kinases tested).

c HMC1.1 11/13 cell line.

d M-07e cell line.

e SW569 cell line.

f 10^–6^ cm/s.

g Intrinsic clearance obtained from isolated human, rat, dog, or monkey microsomes.

h Intrinsic clearance obtained from isolated human, rat, dog, or monkey hepatocyte cells.

i Plasma–protein binding was determined by Rapid Equilibrium Dialysis (RED).

j Male Sprague-Dawley rats (*n* = 3); IV dose = 1 mg/kg and PO dose = 5 mg/kg using 10% Solutol HS15 and 80% (20%HP-β-CD in saline).

k Cynomolgus monkey (*n* = 3), IV dose = 0.5 mpk using 5% DMSO + 5% Kolliphor HS15 + 90% saline and PO dosing was not performed.

l Male beagle dogs, IV dose = 0.5 mg/kg and PO dose = 1.5 mg/kg 5%DMSO + 5%kolliphor HS 15 + 90%saline.

**Figure 7:**
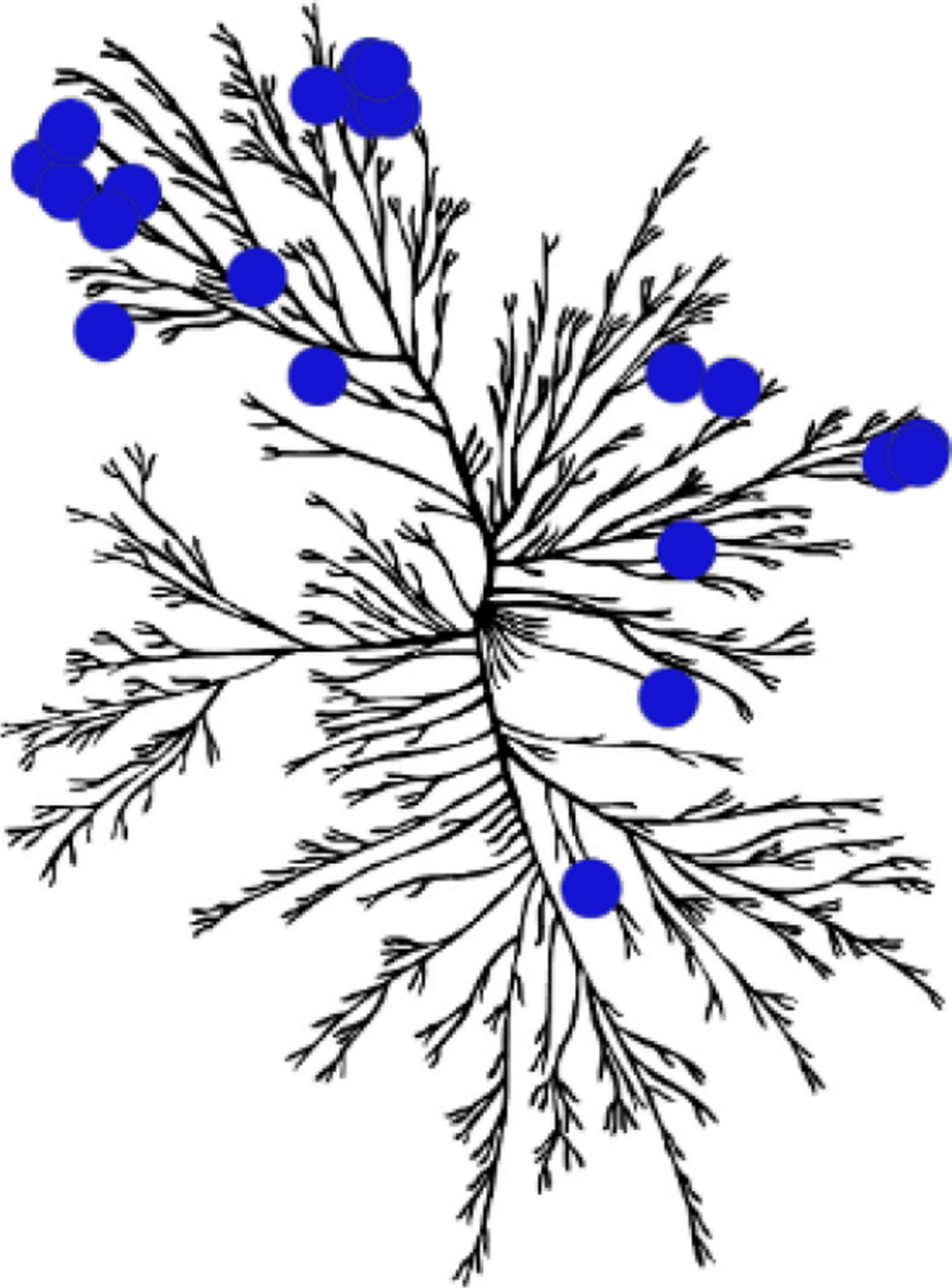
Kinome tree of Compound **18** (BLU-654). Kinome illustration reproduced courtesy of Cell Signaling Technology, Inc. (CST) (www.cellsignal.com). The foregoing Web site is maintained by CST and Blueprint Medicines is not responsible for its content.

Given its excellent potency and kinome selectivity, combined with an appealing PK profile, **18** was evaluated in a PK/PD study using a cell-derived xenograft (CDX) model in HMC1.1 11/13 cell line. Tumor-bearing mice were treated with a single dose of the compound at 1, 3, 10, and 30 mg/kg. Samples were harvested at 4, 10, and 24 h postdosing and assessed against the biomarker phosphorylated signal transducer and activator of transcription 5 (pSTAT5). STAT5 is constitutively activated by KIT mutants resulting in sustained neoplastic mast cell proliferation and survival. When KIT V654A autophosphorylation is inhibited, phosphorylation of STAT5 rapidly decreases, making pSTAT5 a sensitive readout of KIT activity.^34–36^ While pKIT was measured in vivo, the signal-to-noise ratio was low and generally not robust. Furthermore, we observed poor agreement with in vitro potency and poor alignment with in vivo activity and therefore was not used for PK/PD studies. Compound **18** elicited a dose and time-dependent pSTAT5 reduction with a free in vivo IC_50_ of 4.2 nM at 4 h, which correlated well with an in vitro pKIT IC_50_ of 5.7 nM (Figure 8B). Compound **18** also demonstrated dose-dependent exposures, exceeding the in vitro pKIT IC_50_ at most doses after 10 h postdosing and up to 24 h at the highest dose of 30 mg/kg (Figure 8A).

**Figure 8:**
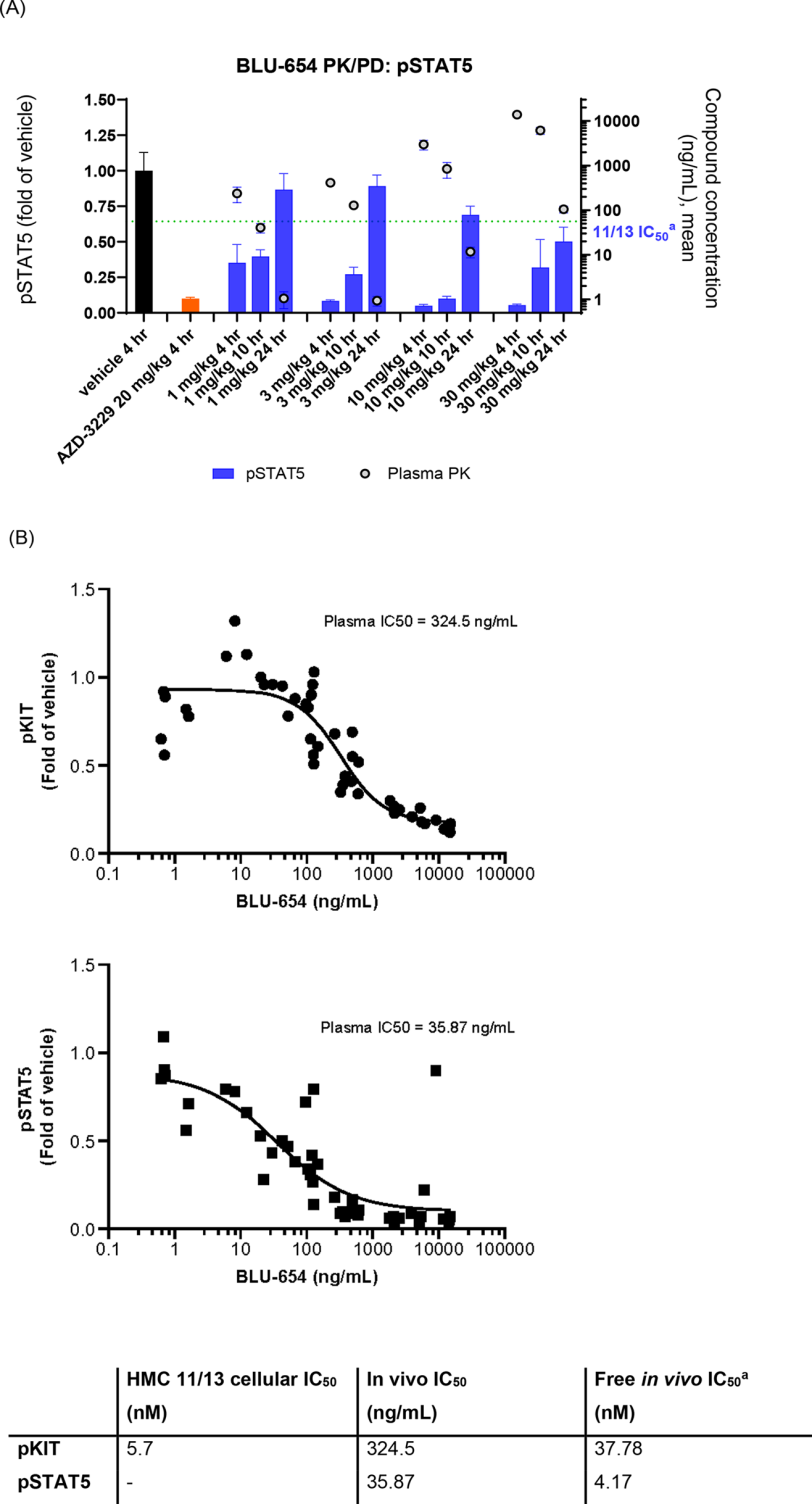
(A) pSTAT5 pharmacodynamic modulation and plasma concentration–time profile of BLU-654. ^a^Plasma protein binding corrected pKIT 11/13 IC_50_ using Fu = 0.05 (in NOD-SCID and measured by Rapid Equilibrium Dialysis) is 48.96 ng.mL^–1^ (B) PK/PD measurements in plasma and PK/PD relationship of BLU-654 in the HMC1.1 11/13 CDX model. ^a^Plasma protein binding corrected pKIT 11/13 IC_50_ using Fu = 0.05 (in NOD-SCID and measured by Rapid Equilibrium Dialysis).

Additionally, **18** was evaluated in an efficacy study using the HMC1.1 11/13 CDX model (Figure 9A) and benchmarked against AZD-3229, a potent KIT/PDGFRα inhibitor covering most clinically relevant mutations observed in GIST.^37–40^ Once tumors reached approximately 200 mm^3^ volume, mice were randomized to receive vehicle or **18** at 3, 10, 30, and 60 mg/kg once daily by oral gavage. Body weight was maintained within 10% margins across doses, which implies that **18** was well tolerated even at the highest dose of 60 mg/kg (Figure 9B). The lowest dose of 3 mg/kg led to slower tumor growth inhibition. After 27 days of dosing, tumors were no longer detectable in the 10, 30, and 60 mg/kg groups, and no regrowth occurred during the 48-day period following the final administration on par with the antitumor activity of AZD-3229 (20 mg/kg) in this model.^18,37^ At the lowest efficacious dose of 10 mg/kg, pSTAT5 was reduced by 95% over 4 h, and returned to 68% at 24h (Figure 8). In summary, compound **18** showed strong antitumor activity in the 11/13 CDX model combined with good tolerability and adequate reduction of PD marker.

**Figure 9:**
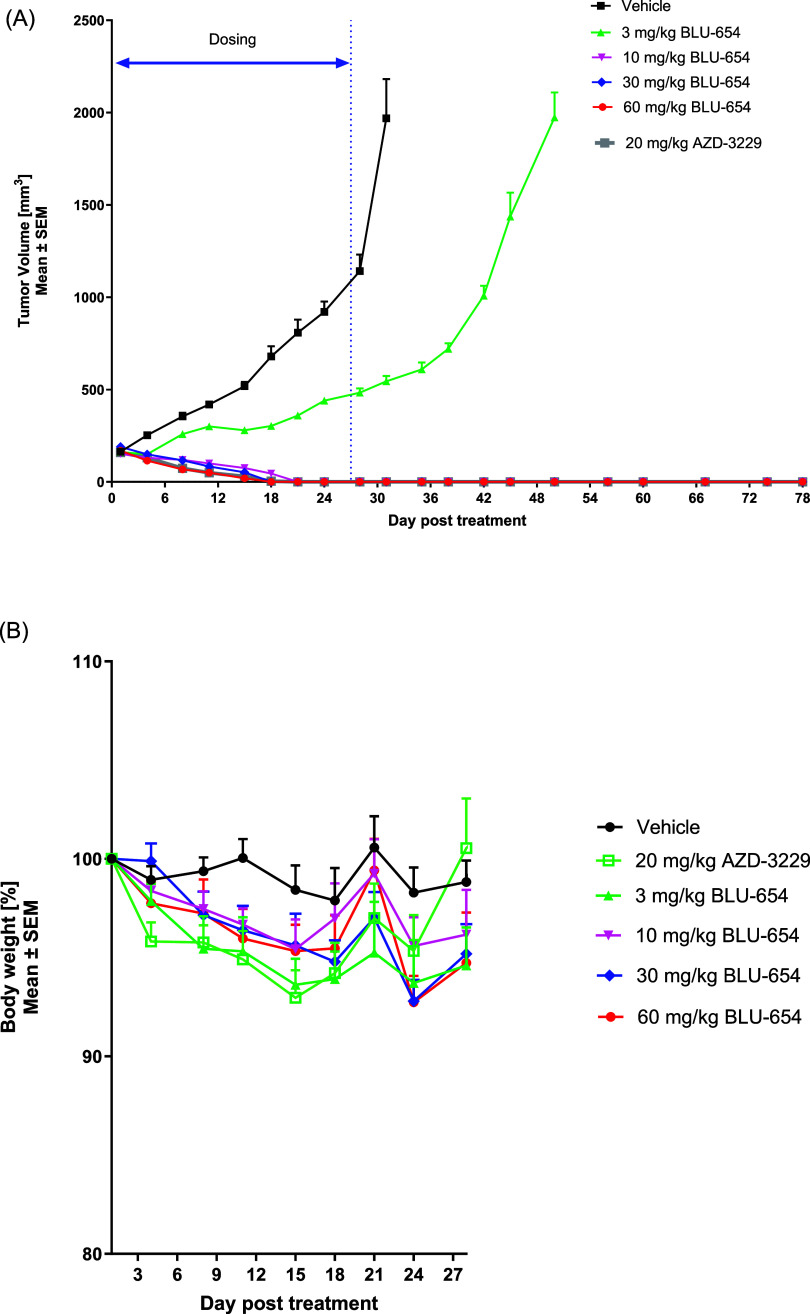
(A) Antitumor activity of Compound **18** (BLU-654) in an HMC1.1 11/13 CDX model and (B) changes in body weight of mice treated with Compound **18** (BLU-654). SEM, standard error of the mean.

Consequently, supported by excellent in vitro and in vivo profiles, **18** was selected as the development candidate **BLU-654**.

## Chemistry

Compounds from both series A and B were accessible by first introducing the ribose group R_2_ via aromatic nucleophilic substitution on the appropriate 4-halopyridyl core (Scheme 1). Then, solvent front motif R_3_ was installed via Suzuki coupling for series A or ester hydrolysis and amide coupling for series B. The right-hand side was attached by classical Buchwald-Hartwig coupling with the adequate 4-aminopyrimidine followed by deprotection as needed.

**Scheme 1:**
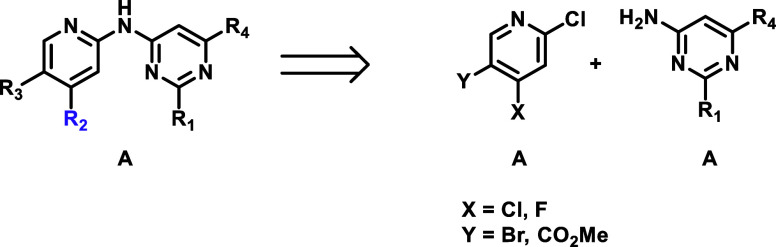
Retrosynthetic Example of Modular Access to Most SAR

In some instances, the required multisubstituted 4-aminopyrimidines were not easily accessible from advanced pyrimidine building blocks and necessitated additional steps for their constructions (Scheme 2). In particular, for the synthesis of **18**, the synthetic sequence started with the condensation of malonamide (**19**) with racemic ethyl 2-fluoropropanoate (**20**) which provided 2-(1-fluoroethyl)­pyrimidine-4,6-diol (**21**). This intermediate was then converted to its dichloro version using phosphorus oxychloride. Introduction of the amine was achieved by nucleophilic aromatic substitution (S_n_Ar) with 2,4-dimethoxybenzylamine and deprotection of the 4,4-dimethoxybenzyl (DMB) group under acidic conditions. Subsequent repetition of the S_n_Ar and chiral supercritical fluid chromatography (SFC) resolution afforded the enantiopure mono DMB-protected diaminopyrimidine key intermediate **25**. The absolute configuration of 25 was determined by X-ray structure (see Supporting Information).

**Scheme 2:**
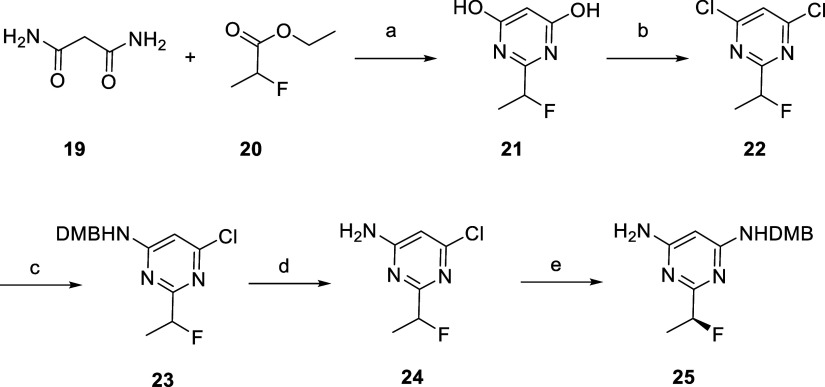
Synthesis of Back Pocket Pyrimidine **25** ^a^

a Reagents and conditions: (a) EtOH, EtONa, 100 °C, 66%, (b) POCl_3_, Et_3_N, toluene, 100 °C, (c) Dimethoxybenzylamine, Hunig’s base, NMP, 100 °C, (d) HCl, EtOAc, 84% (3 steps), and (e) dimethoxybenzylamine, Hunig’s base, NMP, 140 °C, 12%.

Introduction of the isopropoxy ribose group on the 5-bromo-2,4-dichloropyridine core (**26**) was achieved via S_n_Ar under basic conditions in good yield (Scheme 3). Solvent front tertiary alcohol was installed by Suzuki-Miyaura cross coupling under classical conditions using the appropriate commercially available pyrazole boronate ester. Subsequent C–N Buchwald–Hartwig coupling with intermediate **27** using the fourth generation precatalyst BrettPhos and final DMB deprotection under acidic conditions generated **BLU-654** in high overall yields.

**Scheme 3:**
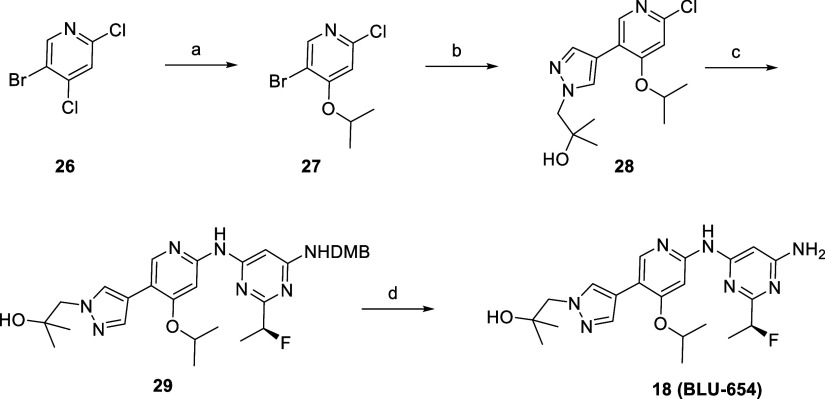
Synthesis of BLU-654^a^

a Reagents and conditions: (a) isopropanol, NaH, THF, 73%, (b) 2-methyl-1-(4-(4,4,5,5-tetramethyl-1,3,2-dioxaborolan-2-yl)-1*H*-pyrazol-1-yl)­propan-2-ol, PdCl2­(dppf), K_2_CO_3_, water, dioxane, 80 °C, 88%, (c) BrettPhos Pd G4, **25**, Cs_2_CO_3_, dioxane, 100 °C, 56%, and (d) TFA, DCM, 67%.

## Conclusions

In summary, we described the discovery efforts that led to the identification of BLU-654 (compound **18**), a kinome-sparing, potent, and selective KIT V654A inhibitor. Leveraging the proprietary annotated Blueprint Medicines compound library, we rapidly identified series A, which bears an aminopyridine core, and the related hit **1**. By exploiting an atypical shorter valine to alanine mutation in the active site of KIT, we successfully identified a key vector to drive selectivity over the wild-type protein while decreasing overall kinome promiscuity. Scaffold modifications resulted in a new nicotinamide core (series B) with improved ADME properties, leading to tool compound **11**. We explored the ribose region to further enhance potency and sought to improve PK properties via solvent front modifications by prosecuting multiple libraries in both series. In the end, series A offered more optimization latitude, and the introduction of a polar group in the solvent-exposed region led to an improved PK profile culminating in **18,** then named BLU-654.

Oral daily administration of BLU-654 as a single agent resulted in sustained antitumor activity in a CDX mouse model carrying a KIT exon 11/13 mutation. Importantly, this inhibitor was well tolerated and showed high selectivity over key off-targets and the overall kinome.

These findings support the potential use of BLU-654 in combination therapy for patients with imatinib-resistant GIST harboring the KIT V654A mutation. Pairing BLU-654 with agents targeting exon 9 and 11 mutations may enable mutation-agnostic, second-line regimens that improve response rates and outcomes across diverse GIST patient populations, while its high kinome selectivity—particularly sparing WT KIT and PDGFRα/β—should help mitigate the risk of related adverse effects.

## Experimental Section

### Compound Synthesis and Characterization

All solvents employed were commercially available anhydrous grade, and reagents were used as received unless otherwise noted. Compound purity of all compounds was assessed by high-performance liquid chromatography (HPLC) to confirm >95% purity. The liquid chromatography–mass spectrometry (LC–MS) data were obtained with an Agilent model-1260 LC system using an Agilent model 6120 mass spectrometer utilizing electrospray ionization as a form of atmospheric pressure ionization (ES-API) fitted with an Agilent Poroshel 120 (EC-C18, 2.7 μm particle size, 3.0 × 50 mm dimensions) reverse-phase column. The mobile phase consisted of a mixture of solvent 0.1% formic acid in water and 0.1% formic acid in acetonitrile. A constant gradient from 95% aqueous/5% organic to 5% aqueous/95% organic mobile phase over the course of 4 min was utilized. The flow rate was constant at 1 mL/min. Alternatively, the LC-MS data were obtained with a Shimadzu LC-MS system using a Shimadzu LC-MS mass spectrometer utilizing ESI ionization fitted with an Agilent (Poroshel HPH-C18 2.7 μm particle size, 3.0 × 50 mm dimensions) reverse-phase column. The mobile phase consisted of a mixture of solvent 5 mM NH_4_HCO_3_ (or 0.05% trifluoroacetic acid [TFA]) in water and acetonitrile. A constant gradient from 90% aqueous/10% organic to 5% aqueous/95% organic mobile phase over the course of 2 min was utilized. The flow rate was constant at 1.5 mL/min. Preparative HPLC was performed on a Shimadzu Discovery VPR Preparative system fitted with a Luna 5 μm C18(2) 100 Å, AXIA packed, 250 × 21.2 mm reverse-phase column. Alternatively, the preparative HPLC was performed on a Waters Preparative system fitted with Column: XBridge Shield RP18 OBD Column, 30 × 150 mm, 5 μm; the mobile phase consisted of a mixture of solvent water (10 mmol/L NH_4_CO3 + 0.05% ΝΗ_3_·Η_2_O) and acetonitrile. A constant gradient from 95% aqueous/5% organic to 5% aqueous/95% organic mobile phase over the course of 11 min was utilized. The flow rate was constant at 60 mL/min. Reactions carried out in a microwave were performed in a Biotage Initiator microwave unit. Silica gel chromatography was performed on a Teledyne Isco CombiFlash Rf unit, a Biotage Isolera Four unit, or a Biotage Isolera Prime unit. 1H nuclear magnetic resonance (NMR) spectra were obtained with a Varian 400 MHz Unity Inova 400 MHz NMR instrument, Avance 400 MHz Unity Inova 400 MHz NMR instrument, or an Avance 300 MHz Unity Inova 300 MHz NMR instrument. Unless otherwise indicated, all protons were reported in dimethyl sulfoxide (DMSO)-d6 solvent as parts-per million (ppm) with respect to residual DMSO (2.50 ppm). Chiral-HPLC was performed on an Agilent 1260 Preparative system. Chiral-supercritical fluid chromatography (SFC) purification was performed with a Waters preparative system.

#### 2-Chloro-4-isopropoxy-5-(1-methyl-1*H*-pyrazol-4-yl)­pyridine (SI-1)

##### Step 1: Synthesis of 5-Bromo-2-chloro-4-isopropoxypyridine (27)

To a solution of i-PrOH (6.60 g, 110 mmol) in THF (150 mL) at 0 °C was added NaH (6.60 g, 165 mmol), the reaction mixture was stirred at 0 °C for 30 min, then 5-bromo-2,4-dichloropyridine **26** (25 g, 110 mmol) in THF (100 mL) was added. The mixture was stirred at room temperature for 12 h. After that, the solution was quenched with water, then extracted with EtOAc. The organic layers were concentrated and purified by flash chromatography on silica gel eluting with PE/EtOAc (10:1–2:1) to get the title product as a white solid (20.0 g, 73% yield). LC-MS *m*/*z* = 250 [M + 1].

##### Step 2: Synthesis of 2-Chloro-4-isopropoxy-5-(1-methyl-1*H*-pyrazol-4-yl)­pyridine

A mixture of 5-bromo-2-chloro-4-isopropoxypyridine (10.0 g, 40 mmol), 1-methyl-4-(4,4,5,5-tetramethyl-1,3,2-dioxaborolan-2-yl)-1*H*-pyrazole (8.32 g, 40 mmol), Pd­(dppf)­Cl_2_ (3.26 g, 4.45 mmol) and K_2_CO_3_ (16.6 g, 120 mmol) in dioxane/water (100 mL/20 mL) was stirred at 90 °C under N_2_ for 4 h. The reaction mixture was partitioned between EtOAc and water. The aqueous layer was extracted with EtOAc, the combined organic layers were washed with brine and dried over anhydrous Na_2_SO_4_. The organic layers were concentrated and purified by flash chromatography on silica gel eluting with PE/EtOAc (3:1) to afford the title compound (5.1 g, 51% yield) as a white solid. LC-MS *m*/*z* = 252 [M + 1].

#### 4-Isopropoxy-5-(1-methyl-1*H*-pyrazol-4-yl)­pyridin-2-amine (SI-2)

##### Step 1: Synthesis of *N*-(Diphenylmethylene)-4-isopropoxy-5-(1-methyl-1*H*-pyrazol-4-yl)­pyridin-2-amine

A mixture of 2-chloro-4-isopropoxy-5-(1-methyl-1*H*-pyrazol-4-yl)­pyridine (3.0 g, 12 mmol), benzophenone imine (4.3 g, 24 mmol), Cs_2_CO_3_ (11.6 g, 36 mmol), Pd_2_(dba)_3_ (1.0 g, 1.2 mmol), XantPhos (0.58 g, 1.2 mmol) in dioxane (30 mL) was stirred at 100 °C under N_2_ for 4 h. LC-MS showed the reaction was completed. The reaction mixture was partitioned between EtOAc and water. The aqueous layer was extracted with EtOAc, then the combined organic layers were washed with brine and dried over anhydrous Na_2_SO_4_. The organic layers were concentrated and purified by flash chromatography on silica gel eluting with PE/EtOAc (2:1) to afford the title compound (4.0 g, 84% yield) as a white solid. LC-MS *m*/*z* = 397 [M + 1].

##### Step 2: Synthesis of 4-Isopropoxy-5-(1-methyl-1*H*-pyrazol-4-yl)­pyridin-2-amine

To a solution of *N*-(diphenylmethylene)-4-isopropoxy-5-(1-methyl-1*H*-pyrazol-4-yl)­pyridin-2-amine (4.0 g, 10 mmol) in DCM (20 mL) was added HCl/dioxane (20 mL), and the reaction mixture was stirred at room temperature for 4 h. The reaction mixture was filtered and was partitioned between DCM and saturated aqueous NaHCO_3_. The organic layer was washed with brine, dried over Na_2_SO_4_, filtered, and concentrated under reduced pressure to give the title product which was used for the next step without further purification (1.88 g, 80% yield). LC-MS *m*/*z* = 233 [M + 1].

#### 2-(Difluoromethyl)-*N* ^4^-(2,4-dimethoxybenzyl)­pyrimidine-4,6-diamine (SI-3)

##### Step 1: Synthesis of 1:2-(Difluoromethyl)­pyrimidine-4,6-diol

To a solution of malonamide (600 g, 5.88 mol) in EtOH (12 L) was added EtONa (799 g, 11.75 mol) and the solution stirred at room temperature for 1 h. Ethyl 2,2-difluoroacetate (875.11 g, 7.05 mol) was added dropwise and the reaction was heated at 90 °C for 15 h. The cooled reaction mixture was filtered, the solid washed with EtOH/EtOAc (1:1), and dried in vacuo to give the title compound (800 g, 71.4%) as a yellow solid.

##### Step 2: Synthesis of 2:4,6-Dichloro-2-(difluoromethyl)­pyrimidine

To a solution of 2-(difluoromethyl)­pyrimidine-4,6-diol (150 g, 740.31 mmol) in toluene (3 L) was added POCl_3_ (300.15 mL, 3.23 mol) dropwise followed by DIPEA (526.95 mL, 3.03 mol) and the reaction heated at 120 °C for 16 h. The cooled reaction mixture was concentrated under reduced pressure, the residue diluted with EtOAc (900 mL), and saturated aq. NaHCO_3_ was added to adjust the pH to 7–8. The layers were separated, the organic layer washed with brine (300 mL × 3), dried over Na_2_SO_4_, filtered, and concentrated under reduced pressure. The residue was purified by column chromatography on silica gel (I0 ∼5% EtOAc/PE) to give the title compound (85 g, 57.1%) as a yellow oil. ^1^H NMR (400 MHz, DMSO-*d*_6_) δ ppm 7.18–6.83 (m, 1 H) 8.31 (s, 1 H).

##### Step 3: Synthesis of 6-Chloro-2-(difluoromethyl)-*N*-(2,4-dimethoxybenzyl)­pyrimidin-4-amine

To a solution of 4,6-dichloro-2-(difluoromethyl)­pyrimidine (50.0 g, 251 mmol) and 2,4-dimethoxybenzylamine (46.2 g, 276 mmol) in NMP (250 mL) was added DIPEA (64.9 g, 502 mmol) and the reaction stirred at 140 °C for 2 h. The cooled reaction mixture was poured into water (500 mL) and extracted with EtOAc (400 mL × 3). The combined organic layers were washed with brine (500 mL), dried over Na_2_SO_4_, filtered, and the filtrate was concentrated under reduced pressure. The crude product was purified by silica gel chromatography (PE/EtOAc = 50/1–10/1) to give the title compound (60.0 g, 70.3% yield) as a yellow solid. LC-MS *m*/*z* = 330 [M + 1]; ^1^H NMR (400 MHz, DMSO-*d*_6_) δ: 8.61–8.23 (m, 1H), 7.24–7.06 (m, 1H), 6.80–6.44 (m, 4H), 4.50–4.23 (m, 2H), 3.79 (s, 3H), 3.74 (s, 3H).

##### Step 4: Synthesis of 4:6-Chloro-2-(difluoromethyl)­pyrimidin-4-amine

A solution of 6-chloro-2-(difluoromethyl)-*N*-(2,4-dimethoxybenzyl)­pyrimidin-4-amine (60.0 g, 176 mmol) in HCl/EtOAc (4 M, 257 mL) was stirred at room temperature for 12 h. The pH of the mixture was adjusted to about 8 with saturated aqueous NaHCO_3_ solution. The mixture was extracted with EtOAc (500 mL × 3) and the combined organic layer was evaporated under reduced pressure to give the title compound (30 g) which was used without further purification. LC-MS *m*/*z* = 180 [M + 1].

##### Step 5: Synthesis of 5:2-(Difluoromethyl)-*N* ^4^-(2,4-dimethoxybenzyl)­pyrimidine-4,6-diamine

The title compound was obtained as an off-white solid (25.31 g, 47.8% yield) from 6-chloro-2-(difluoromethyl)­pyrimidin-4-amine and 2,4-dimethoxybenzylamine following a similar procedure to that described in step 3. LC-MS *m*/*z* = 311.1 [M + 1]; ^1^H NMR (400 MHz, DMSO-*d*_6_) δ 7.16 (t, 1H), 7.07 (d, 1H), 6.58–6.19 (m, 5H), 5.35 (s, 1H), 4.24 (s, 2H), 3.80 (s, 3H), 3.73 (s, 3H).

#### 2-(Difluoromethyl)­pyrimidine-4,6-diamine hydrochloride (SI-4)

##### Step 1: Synthesis of 6: *N*,*N*′-(2-(Difluoromethyl)­pyrimidine-4,6-diyl)­bis­(1,1-diphenyl methanimine)

To a solution of 4,6-dichloro-2-(difluoromethyl)­pyrimidine (145 g, 721.41 mmol) in dioxane (2 L) was added benzophenone imine (326.86 g, 1.80 mol), Cs_2_CO_3_ (705.15 g, 2.16 mol), XantPhos (41.74 g, 72.14 mmol), and Pd_2_(dba)_3_ (33.03 g, 36.07 mmol) under N_2_ and the reaction was heated at 100 °C for 8 h. The cooled reaction mixture was filtered and concentrated under reduced pressure. The residue was purified by silica gel chromatography (0–10% EtOAc/PE) to give the title compound (250 g, 67%) as a yellow oil. ^1^H NMR (400 MHz, DMSO-*d*_6_) δ ppm 6.39–6.57 (m, 2H) 7.27–7.60 (m, 20H).

##### Step 2: Synthesis of 7:2-(Difluoromethyl)­pyrimidine-4,6-diamine hydrochloride

To a solution of *N*,*N*′-(2-(difluoromethyl)­pyrimidine-4,6-diyl)­bis­(1,1-diphenyl methanimine) (250 g, 511.74 mmol) in dioxane (1.5 L) was added HCl/dioxane (4 M, 800 mL) and the reaction stirred at room temperature for 16 h. The reaction was concentrated under reduced pressure and the residue was triturated with THF (1 L) for 30 min. The solid was filtered off to give the title compound (113 g, 68.6% yield) as a brown solid. LC-MS *m*/*z* = 160 [M + 1].

#### *N* ^4^-(4-Isopropoxy-5-(1-methyl-1*H*-pyrazol-4-yl)­pyridin-2-yl)-*N* ^2^-methylpyrimidine-2,4-diamine (1)

##### Step 1: Synthesis of 2-Chloro-*N*-(4-isopropoxy-5-(1-methyl-1*H*-pyrazol-4-yl)­pyridin-2-yl)­pyrimidin-4-amine

To a solution of **SI-1** (0.50 g, 2.15 mmol, 1.00 eq) in DMF (10 mL) was added Cs_2_CO_3_ (1.40 g, 4.31 mmol, 2.00 eq), XantPhos (125 mg, 215 μmol, 0.10 eq), and Pd_2_(dba)_3_ (197 mg, 215 μmol, 0.10 eq) in portion under N_2_, then 2,4-dichloropyrimidine (353 mg, 2.37 mmol, 1.10 eq) was added at this temperature. The reaction was stirred at room temperature for 12 h. The reaction mixture was filtered and the filter was concentrated under reduced pressure. The crude product was purified by prep-TLC (PE:EtOAc; 0:1, *R*_f_ = 0.5) to give the title compound (200.0 mg, 580 μmol, 27.0% yield) as a yellow solid. LC-MS *m*/*z* = 345.1 [M + 1]; ^1^H NMR (400 MHz, DMSO-*d*_6_) δ ppm 10.54 (br.s, 1H), 8.45 (s, 1H), 8.31 (d, *J* = 5.6 Hz, 1H), 8.07 (s, 1H), 7.91 (s, 1H), 7.70 (br.s, 1H), 7.43 (br.s, 1H), 4.71–4.74 (m, 1H), 3.87 (s, 3H),1.44 (d, *J* = 6.0 Hz, 6H).

##### Step 2: Synthesis of *N* ^4^-(4-Isopropoxy-5-(1-methyl-1*H*-pyrazol-4-yl)­pyridin-2-yl)-*N*2-methylpyrimidine-2,4-diamine

To a solution of 2-chloro-*N*-(4-isopropoxy-5-(1-methyl-1*H*-pyrazol-4-yl)­pyridin-2-yl)­pyrimidin-4-amine (50.0 mg, 145 μmol, 1.00 eq) and methanamine (19.6 mg, 290 μmol, 2.00 *eq*, HCl) in EtOH (2 mL) was added Et_3_N (44.0 mg, 435 μmol, 60.6 uL, 3.00 eq). The reaction was stirred at 80 °C for 30 h in a sealed tube. The mixture was adjusted to pH to 6–7 with HCl solution (4 N) and the residue was concentrated in vacuum. The residue was purified by prep-HPLC (column: Boston Prime C18 150 × 30 mm 5 μm; mobile phase: [water (0.1% TFA)—ACN]; B%: 15–45%, 9 min) to afford the title compound (16.8 mg, 36.7 μmol, 25.3% yield, TFA salt) as a light yellow solid. LC-MS *m*/*z* = 340.2 [M + 1]; ^1^H NMR (400 MHz, DMSO) δ 11.11 (s, 1H), 8.54 (s, 1H), 8.42 (s, 1H), 8.12 (s, 1H), 7.97 (d, *J* = 11.3 Hz, 2H), 6.59 (s, 1H), 4.84 (s, 1H), 3.89 (s, 3H), 2.99 (s, 3H), 1.42 (d, *J* = 6.0 Hz, 6H).

#### 6-Chloro-*N* ^4^-(4-isopropoxy-5-(1-methyl-1*H*-pyrazol-4-yl)­pyridin-2-yl)-*N*2-methylpyrimidine-2,4-diamine (2)

##### Step 1: Synthesis of 6-Chloro-*N* ^2^-methyl-pyrimidine-2,4-diamine

To a solution of 2,6-dichloropyrimidin-4-amine (500 mg, 3.04 mmol) and methanamine hydrochloride (307 mg, 4.56 mmol) in IPA (7 mL) was added DIPEA (1.96 g, 15.2 mmol) at room temperature. The mixture was stirred at 90 °C for 3 h. The reaction mixture was purified by silica gel chromatography (PE/EA = 1:1) to obtain the title product (300 mg, 62% yield) as a white solid. LC-MS *m*/*z* = 159 [M + 1].

##### Step 2: Synthesis of 6-Chloro-*N* ^4^-(4-isopropoxy-5-(1-methyl-1*H*-pyrazol-4-yl)­pyridin-2-yl)-*N* ^2^-methylpyrimidine-2,4-diamine

To a mixture of 6-chloro-*N* ^2^-methylpyrimidine-2,4-diamine (188 mg, 1.19 mmol) and 2-chloro-5-(1-methyl-1*H*-pyrazol-4-yl)-4-(propan-2-yloxy)­pyridine **SI-1** (150 mg, 597 μmol) in dioxane (5 mL) was added Pd_2_(dba)_3_ (86.5 mg, 94.5 μmol), XantPhos (109 mg, 189 μmol), and Cs_2_CO_3_ (585 mg, 1.79 mmol) at room temperature. The reaction mixture was stirred at 100 °C for 14 h under N_2_. The reaction mixture was concentrated and the residue was purified by silica gel chromatography (EA/MeOH = 9:1) then by Prep-HPLC (A:water­(10 mM NH_4_HCO_3_ and 0.025% NH_3_·H_2_O)), B: CAN; 45% B for 1 min, then 60% B in 7 min, stop at 15 min) to obtain the title product (18.7 mg, 8% yield) as a white solid. LC-MS *m*/*z* = 374 [M + 1]; ^1^H NMR (500 MHz, DMSO) δ 9.89 (s, 1H), 8.41 (s, 1H), 8.04 (s, 1H), 7.89 (s, 1H), 7.85 (s, 1H), 7.32 (s, 1H), 6.69 (s, 1H), 4.78 (s, 1H), 3.87 (s, 3H), 2.85 (s, 3H), 1.40 (d, *J* = 6.1 Hz, 6H).

#### 6-Fluoro-*N* ^4^-(4-isopropoxy-5-(1-methyl-1*H*-pyrazol-4-yl)­pyridin-2-yl)-*N*2-methylpyrimidine-2,4-diamine (3)

##### Step 1: Synthesis of 4,6-Difluoro-*N*-methylpyrimidin-2-amine

To a solution of 2,4,6-trifluoropyrimidine (1.0 g, 7.46 mmol) and methanamine hydrochloride (754 mg, 11.2 mmol) in IPA (10 mL) was added DIPEA (2.89 g, 22.4 mmol) at −20 °C, then was stirred and allowed to warm to room temperature gradually. The reaction mixture was purified by silica gel chromatography (PE/EA = 1:1) to obtain the title product (400 mg, 37% yield) as a yellow solid. LC-MS *m*/*z* = 146 [M + 1].

##### Step 2: Synthesis of 6-Fluoro-*N* ^2^-methylpyrimidine-2,4-diamine

A mixture of 4,6-difluoro-*N*-methylpyrimidin-2-amine (400 mg, 2.75 mmol) in NH_3_/dioxane (0.5 M, 10 mL) was stirred at 60 °C for 15 h. The reaction mixture was concentrated under reduced pressure to give the title product (300 mg, crude) which was used in the next step without further purification. LC-MS *m*/*z* = 143 [M + 1].

##### Step 3: Synthesis of 6-Fluoro-*N* ^4^-(4-isopropoxy-5-(1-methyl-1*H*-pyrazol-4-yl)­pyridin-2-yl)-*N* ^2^-methylpyrimidine-2,4-diamine

A mixture of 6-fluoro-*N* ^2^-methylpyrimidine-2,4-diamine (100 mg, 0.70 mmol), 2-chloro-5-(1-methyl-1*H*-pyrazol-4-yl)-4-(propan-2-yloxy)­pyridine **SI-1** (118 mg, 0.47 mmol), Pd_2_(dba)_3_ (43 mg, 47 μmol), XantPhos 54 mg, 94 μmol), and Cs_2_CO_3_ (154 mg, 1.41 mmol) in dioxane (8 mL) was stirred at 100 °C for 14 h under N_2_. The reaction mixture was concentrated then purified by silica gel chromatography (EA/MeOH = 9:1), then by Prep-HPLC (A: water­(10 mM NH_4_HCO_3_ & 0.025% NH_3_·H_2_O)), B: CAN; 45% B for 1 min, then 60% B in 7 min, stop at 15 min) to obtain the title product (54.6 mg, 32% yield) as a white solid. LC-MS *m*/*z* = 358 [M + 1]; ^1^H NMR (500 MHz, DMSO) δ 9.90 (s, 1H), 8.40 (s, 1H), 8.04 (s, 1H), 7.89 (d, *J* = 0.7 Hz, 1H), 7.82 (s, 1H), 7.25 (s, 1H), 6.32 (s, 1H), 4.78 (s, 1H), 3.88 (s, 3H), 2.87 (s, 2H), 1.41 (d, *J* = 6.0 Hz, 6H).

#### *N* ^4^-(4-Isopropoxy-5-(1-methyl-1*H*-pyrazol-4-yl)­pyridin-2-yl)-*N* ^2^,6-dimethylpyrimidine-2,4-diamine (4)

##### Step 1: Synthesis of *N* ^2^,6-Dimethylpyrimidine-2,4-diamine

A mixture of 2-chloro-6-methylpyrimidin-4-amine (1 g, 7.0 mmol) in MeNH_2_/THF (2.0 M, 10 mL) was stirred at 80 °C for 15 h. The reaction was evaporated in vacuum to give the title product (600 mg, crude) which was used in the next step directly without further purification. LC-MS *m*/*z* = 139 [M + 1].

##### Step 2: Synthesis of *N* ^4^-(4-Isopropoxy-5-(1-methyl-1*H*-pyrazol-4-yl)­pyridin-2-yl)-*N* ^2^,6-dimethylpyrimidine-2,4-diamine

To a mixture of *N* ^2^,6-dimethylpyrimidine-2,4-diamine (42 mg, 0.30 mmol) and 2-chloro-5-(1-methyl-1*H*-pyrazol-4-yl)-4-(propan-2-yloxy)­pyridine **SI-1** (50 mg, 0.20 mmol) in dioxane (5 mL) was added Pd_2_(dba)_3_ (18 mg, 20 μmol), XantPhos (23 mg, 40 μmol), and Cs_2_CO_3_ (196 mg, 0.60 mmol) at room temperature. The reaction mixture was stirred at 100 °C for 14 h under N_2_. The reaction mixture was concentrated and the residue was purified by silica gel chromatography (EA/MeOH = 9:1) then by Prep-HPLC (A:water­(10 mM NH_4_HCO_3_ & 0.025% NH_3_·H_2_O)), B: CAN; 45% B for 1 min, then 60% B in 7 min, stop at 15 min) to obtain the title product (56.1 mg, 79% yield) as a white solid. LC-MS *m*/*z* = 354 [M + 1]; ^1^H NMR (500 MHz, DMSO) δ 9.51 (s, 1H), 8.36 (s, 1H), 8.02 (s, 1H), 7.96 (s, 1H), 7.87 (d, *J* = 0.7 Hz, 1H), 6.65 (s, 1H), 6.43 (s, 1H), 4.88 – 4.71 (m, 1H), 3.87 (s, 3H), 2.84 (d, *J* = 4.7 Hz, 3H), 2.12 (s, 3H), 1.39 (d, *J* = 6.0 Hz, 6H).

#### *N* ^4^-(4-Isopropoxy-5-(1-methyl-1*H*-pyrazol-4-yl)­pyridin-2-yl)-6-methoxy-*N* ^2^-methylpyrimidine-2,4-diamine (5)

##### Step 1: Synthesis of 4-Chloro-6-methoxy-*N*-methylpyrimidin-2-amine

A mixture of 4,6-dichloro-*N*-methylpyrimidin-2-amine (450 mg, 2.54 mmol) and MeONa (686 mg, 12.7 mmol) in MeOH (10 mL) was stirred at 60 °C for 15 h. The reaction mixture was concentrated and the residue was purified by silica gel chromatography (PE/EA = 2:1) to obtain the title product (400 mg, 90% yield) as a yellow solid. LC-MS *m*/*z* = 174 [M + 1].

##### Step 2: Synthesis of *N* ^4^-(4-Isopropoxy-5-(1-methyl-1*H*-pyrazol-4-yl)­pyridin-2-yl)-6-methoxy-*N*2-methylpyrimidine-2,4-diamine

A mixture of 4-chloro-6-methoxy-*N*-methylpyrimidin-2-amine (119 mg, 0.68 mmol), 4-isopropoxy-5-(1-methyl-1*H*-pyrazol-4-yl)­pyridin-2-amine **SI-3** (80 mg, 0.34 mmol), Pd­(*t*-Bu_3_P)_2_ (30 mg, 68 μmol), and *t*-BuONa (98 mg, 1.02 mmol) in toluene (8 mL) was stirred at 80 °C for 14 h under N_2_. The reaction mixture was concentrated and the residue was purified by silica gel chromatography (EA/MeOH = 9:1) to obtain the crude product which was further purified with Prep-HPLC (A:water­(10 mM NH_4_HCO_3_ and 0.025% NH_3_·H_2_O)), B: CAN; 45% B for 1 min, then 60% B in 7 min, stop at 15 min) to obtain the title product (9.0 mg, 7% yield) as a white solid. LC-MS *m*/*z* = 370 [M + 1]; 1H NMR (400 MHz, DMSO-d6) δ: 9.40 (s, 1H), 8.34 (s, 1H), 8.02 (s, 1H), 7.87 (s, 1H), 7.80 (s, 1H), 6.77 (s, 1 H), 6.14 (s, 1H), 4.80 (s, 1H), 3.87 (s, 3H), 3.77 (s, 3H), 2.85 (d, *J* = 4.7 Hz, 3H), 1.40 (d, *J* = 6.0 Hz, 6H).

#### *N* ^4^-(4-Isopropoxy-5-(1-methyl-1*H*-pyrazol-4-yl)­pyridin-2-yl)-*N*2-methylpyrimidine-2,4,6-triamine (6)

##### Step 1: Synthesis of *N*4,*N*6-Dibenzyl-*N*2-methylpyrimidine-2,4,6-triamine

To a solution of 4,6-dichloro-*N*-methylpyrimidin-2-amine (1.0 g, 5.65 mmol) in benzylamine (8 mL) was stirred at 200 °C under microwave for 1 h. LC-MS showed the reaction was completed. The mixture was purified by flash column chromatography (PE/EtOAc = 1/3) to afford the title compound (1.0 g, 56% yield) as a yellow solid. LC-MS *m*/*z* = 320 [M + 1].

##### Step 2: Synthesis of *N* ^2^-Methylpyrimidine-2,4,6-triamine

To a solution of *N* ^4^,*N* ^6^-dibenzyl-*N* ^2^-methylpyrimidine-2,4,6-triamine (1.0 g, 3.13 mmol) in DCM (10 mL) was added trifluoromethanesulfonic acid (5 mL) slowly at 0 °C and stirred at 0 °C for 1 h. The mixture was basified with 10% NaOH to pH ∼ 10. Then the mixture was evaporated *in vacuo* and the residue was purified by flash column chromatography (DCM/MeOH = 10/1) to afford the title compound (380 mg, 87% yield) as a white solid. LC-MS *m*/*z* = 140 [M + 1].

##### Step 3: Synthesis of *N* ^4^-(4-Isopropoxy-5-(1-methyl-1*H*-pyrazol-4-yl)­pyridin-2-yl)-*N* ^2^-methylpyrimidine-2,4,6-triamine

A mixture of *N* ^2^-methylpyrimidine-2,4,6-triamine (250 mg, 1.80 mmol), 2-chloro-4-isopropoxy-5-(1-methyl-1*H*-pyrazol-4-yl)­pyridine **SI-1** (300 mg, 1.20 mmol), BrettPhos Pd G3 (110 mg, 0.12 mmol), and KOAc (353 mg, 3.60 mmol) in dioxane (25 mL) was stirred at 100 °C for 16 h under a nitrogen atmosphere. The reaction mixture was concentrated and the residue was purified by silica gel chromatography (EA/MeOH = 6:1) to obtain the crude product which was further purified with Prep-HPLC (A: water­(10 mM NH_4_HCO_3_ and 0.025% NH_3_·H_2_O)), B: CAN; 45% B for 1 min, then 60% B in 7 min, stop at 15 min) to obtain the title product (67.8 mg, 15% yield) as a white solid. LC-MS *m*/*z* = 355 [M + 1]; ^1^H NMR (500 MHz, DMSO) δ 9.03 (s, 1H), 8.28 (s, 1H), 8.00 (s, 1H), 7.85 (d, *J* = 0.9 Hz, 1H), 7.79 (s, 1H), 6.03 (s, 1H), 5.94 (s, 2H), 5.85 (s, 1H), 4.75 (p, *J* = 6.0 Hz, 1H), 3.87 (s, 3H), 2.79 (d, *J* = 4.8 Hz, 3H), 1.39 (d, *J* = 6.0 Hz, 6H).

#### *N* ^4^-(4-Isopropoxy-5-(1-methyl-1*H*-pyrazol-4-yl)­pyridin-2-yl)-2-methylpyrimidine-4,6-diamine (7)

##### Step 1: Synthesis of *N* ^4^,*N* ^6^-Dibenzyl-2-methylpyrimidine-4,6-diamine

A solution of 4,6-dichloro-2-methylpyrimidine (1.00 g, 6.13 mmol) in benzylamine (8 mL) was stirred at 130 °C for 16 h. The mixture was filtered and the filtrate was concentrated *in vacuo,* then purified by reversed-phase chromatography (0.1% NH_3_·H_2_O in water/ACN) to give the title compound (1.30 g, 55% yield) as a yellow solid.

##### Step 2: Synthesis of 2-Methylpyrimidine-4,6-diamine

A mixture of *N* ^4^,*N* ^6^-dibenzyl-2-methylpyrimidine-4,6-diamine (1.00 g, 3.29 mmol) in dichloromethane (8 mL) was added to trifluoromethanesulfonic acid (6.80 g, 45.31 mmol). The mixture was stirred at 25 °C for 0.5 h. The reaction mixture was then basified with 10% sodium hydroxide to pH ∼ 10. The mixture was concentrated under reduced pressure then purified by silica gel chromatography (dichloromethane/methanol = 10/1)) to give the title compound (200 mg, 49% yield) as a yellow solid. ^1^H NMR (400 MHz, 6*d*-DMSO) δ ppm 6.10 (s, 4H), 5.25 (s, 1H), 2.11 (s, 3H).

##### Step 3: Synthesis of *N* ^4^-(4-isopropoxy-5-(1-methyl-1*H*-pyrazol-4-yl)­pyridin-2-yl)-2-methylpyrimidine-4,6-diamine

To a solution of 2-methylpyrimidine-4,6-diamine (73.9 mg, 596 μmol) and **SI-1** (50.0 mg, 199 μmol) in dioxane (3 mL) was added Cs_2_CO_3_ (194 mg, 596 μmol), BrettPhos Pd G4 (18.29 mg, 19.86 μmol), and BrettPhos (10.66 mg, 19.86 μmol). The mixture was stirred at 100 °C for 1 h. The reaction mixture was concentrated under reduced pressure then purified by prep-HPLC (column: Waters Xbridge C18 150 × 50 mm × 10 μm; mobile phase: [water­(10 mM NH_4_HCO_3_)-ACN]; B%: 15–45%,10 min) to give the title compound (13.0 mg, 19% yield) as a white solid. LC-MS *m*/*z* = 340 [M + 1]; ^1^H NMR (400 MHz, CDCl_3_) δ 8.34 (s, 1H), 7.88 (s, 1H), 7.77 (s, 1H), 7.67 (s, 1H), 7.20 (s, 1H), 6.58 (s, 1H), 4.82 (s, 2H), 4.74 (p, *J* = 6.1 Hz, 1H), 3.98 (s, 3H), 2.47 (s, 3H), 1.49 (d, *J* = 6.0 Hz, 6H).

#### 2-Cyclopropyl-*N* ^4^-(4-isopropoxy-5-(1-methyl-1*H*-pyrazol-4-yl)­pyridin-2-yl)­pyrimidine-4,6-diamine (8)

##### Step 1: Synthesis of *N*,*N*′-(2-Cyclopropylpyrimidine-4,6-diyl)­bis­(1,1-diphenylmethanimine)

A mixture of 4,6-dichloro-2-cyclopropylpyrimidine (650 mg, 3.45 mmol), benzophenone imine (1.87 g, 10.3 mmol), Pd_2_(dba)_3_ (311 mg, 0.34 mmol), XantPhos (393 mg, 0.68 mmol), and Cs_2_CO_3_ (3.37 g, 10.3 mmol) in dioxane (50 mL) was stirred at 100 °C for 16 h under a nitrogen atmosphere. The reaction mixture was cooled to room temperature and diluted with dioxane, filtered, and the filtrate was directly concentrated to dryness under reduced. The resulting crude product was purified by silica gel chromatography eluting with DCM/MeOH (15/1) to afford the title compound (400 mg, 24% yield) as a yellow solid. LC-MS *m*/*z* = 479 [M + 1].

##### Step 2: Synthesis of 2-Cyclopropylpyrimidine-4,6-diamine

To a solution of *N*,*N*′-(2-cyclopropylpyrimidine-4,6-diyl)­bis­(1,1-diphenylmethanimine (400 mg, 0.836 mmol) in methanol (10 mL) was added hydroxylamine (0.16 g, 50 wt % in water, 2.51 mmol) at room temperature under a nitrogen atmosphere. After addition, the reaction mixture was heated to 70 °C and stirred for 12 h. The reaction mixture was directly concentrated under reduced pressure. The residue was dissolved into DCM and washed with water. The aqueous layer was basified with NaHCO_3_ to pH > 10, then extracted with DCM. The combined organic layers were concentrated under reduced pressure then purified by flash column chromatography on silica gel eluting with DCM/MeOH (10/1) to afford the title compound (70 mg, 56% yield) as a yellow solid. LC-MS *m*/*z* = 151 [M + 1].

##### Step 3: Synthesis of 2-Cyclopropyl-*N* ^4^-(4-isopropoxy-5-(1-methyl-1*H*-pyrazol-4-yl)­pyridin-2-yl)­pyrimidine-4,6-diamine

A mixture of 2-cyclopropylpyrimidine-4,6-diamine (70 mg, 0.46 mmol), 2-chloro-4-isopropoxy-5-(1-methyl-1*H*-pyrazol-4-yl)­pyridine **SI-1** (117 mg, 0.46 mmol), Pd_2_(dba)_3_ (82 mg, 0.09 mmol), XantPhos (104 mg, 0.18 mmol), and Cs_2_CO_3_ (451 mg, 1.38 mmol) in dioxane (5 mL) was stirred at 100 °C for 16 h under a nitrogen atmosphere. The reaction mixture was cooled to room temperature and diluted with dioxane, filtered, and the filtrate was concentrated under reduced pressure and purified by flash column chromatography on silica gel eluting with DCM/MeOH (5/1) to afford the title compound (17.5 mg, 10% yield) as a white solid. LC-MS *m*/*z* = 366 [M + 1]; ^1^H NMR (500 MHz, DMSO) δ 9.35 (s, 1H), 8.36 (s, 1H), 8.06 (d, *J* = 0.7 Hz, 1H), 7.91 (d, *J* = 0.8 Hz, 1H), 7.59 (s, 1H), 6.50 (s, 1H), 6.39 (s, 2H), 4.77 (p, *J* = 6.0 Hz, 1H), 3.93 (s, 3H), 1.88 (tt, *J* = 8.1, 4.7 Hz, 1H), 1.47 (d, *J* = 6.0 Hz, 6H), 0.98 (dt, *J* = 5.4, 2.8 Hz, 2H), 0.92 (dt, *J* = 8.3, 3.1 Hz, 2H).

#### *N* ^4^-(5-Isopropyl-8-methoxy-2,7-naphthyridin-3-yl)-*N* ^2^-methylpyrimidine-2,4,6-triamine (9)

##### Step 1: Synthesis of 6-Chloro-4-(prop-1-en-2-yl)-2,7-naphthyridin-1­(2*H*)-one

A mixture of 6-chloro-4-iodo-1,2-dihydro-2,7-naphthyridin-1-one (3.0 g, 9.8 mmol), 4,4,5,5-tetramethyl-2-(prop-1-en-2-yl)-1,3,2-dioxaborolane (2.45 g, 14.6 mmol), TEA (2.97 g, 29.4 mmol), and Pd­(dppf)­Cl_2_ (0.72 g, 0.98 mol) in THF/DMF/water (60/20/20 mL) was heated to 70 °C for 3 h under N_2_. The reaction mixture was filtered, and the filtrate was extracted with EtOAc. The combined organic layers were concentrated under reduced pressure and purified by silica gel chromatography eluting with PE/EtOAc (1:1) to afford the title compound (1.3 g, 60% yield) as a light brown solid. LC-MS *m*/*z* = 221 [M + 1].

##### Step 2: Synthesis of 6-Chloro-4-isopropyl-2,7-naphthyridin-1­(2*H*)-one

A mixture of 6-chloro-4-(prop-1-en-2-yl)-1,2-dihydro-2,7-naphthyridin-1-one (1.3 g, 5.9 mmol) and PtO_2_ (1.3 g, 5.7 mmol) in EtOAc (50 mL) was stirred at room temperature for 1.5 h under H_2_ atmosphere. The reaction was filtered, and the filtrate was concentrated under reduced pressure to give the title compound (800 mg, crude), which was used for the next step without further purification. LC-MS *m*/*z* = 223 [M + 1].

##### Step 3: Synthesis of 1,6-Dichloro-4-isopropyl-2,7-naphthyridine

A mixture of 6-chloro-4-isopropyl-2,7-naphthyridin-1­(2*H*)-one (800 mg, 3.60 mmol) and POCl_3_ (1.67 mL, 18 mmol) in toluene (20 mL) was stirred at 110 °C for 3 h. LC-MS showed the reaction was completed. The reaction mixture was diluted with DCM and washed with water and brine. The organic layer was concentrated under reduced pressure and purified by flash column chromatography on silica gel eluting with DCM/MeOH (10/1) to give the title compound (400 mg, 46% yield) as a yellow solid. LC-MS *m*/*z* = 241 [M + 1].

##### Step 4: Synthesis of 6-Chloro-4-isopropyl-1-methoxy-2,7-naphthyridine

A mixture of 1,6-dichloro-4-isopropyl-2,7-naphthyridine (400 mg, 1.67 mmol) and K_2_CO_3_ (228 mg, 2.5 mmol) in MeOH (5 mL) was stirred at room temperature for 3 h. The reaction mixture was diluted with DCM and washed with water and brine. The organic layer was concentrated, and the residue was purified by flash column chromatography on silica gel eluting with PE/EA (1/1) to give the title compound (200 mg, 50% yield) as a yellow solid. LC-MS *m*/*z* = 237 [M + 1].

##### Step 5: Synthesis of *N* ^4^-(5-Isopropyl-8-methoxy-2,7-naphthyridin-3-yl)-*N* ^2^-methylpyrimidine-2,4,6-triamine

A mixture of *N* ^2^-methylpyrimidine-2,4,6-triamine (70 mg, 0.50 mmol), 6-chloro-4-isopropyl-1-methoxy-2,7-naphthyridine (60 mg, 0.25 mmol), Pd_2_(dba)_3_ (45 mg, 0.05 mmol), XantPhos (58 mg, 0.10 mmol), and Cs_2_CO_3_ (245 mg, 0.75 mmol) in dioxane (5 mL) was stirred at 100 °C for 5 h under a nitrogen atmosphere. The reaction mixture was concentrated under reduced pressure and purified by silica gel chromatography (EA/MeOH = 3:1) then Prep-HPLC (A: water­(10 mM NH_4_HCO_3_ and 0.025% NH_3_·H_2_O)), B: CAN; 45% B for 1 min, then 60% B in 7 min, stop at 15 min) to obtain the title product (7.3 mg, 8% yield) as a white solid. LC-MS *m*/*z* = 340 [M + 1]; ^1^H NMR (500 MHz, DMSO) δ 9.72 (s, 1H), 9.20 (s, 1H), 8.75 (s, 1H), 7.94 (s, 1H), 6.27 (s, 1H), 6.10 (s, 2H), 5.64 (s, 1H), 4.03 (s, 3H), 3.43 – 3.35 (m, 1H), 2.89 (d, *J* = 4.7 Hz, 3H), 1.32 (d, *J* = 6.8 Hz, 6H).

#### 6-((6-Amino-2-(methylamino)­pyrimidin-4-yl)­amino)-4-(isopropylamino)-*N*-methylnicotinamide (10)

##### Step 1: Synthesis of Methyl 6-Chloro-4-(isopropylamino)­nicotinate

To a solution of methyl 4,6-dichloropyridine-3-carboxylate (50 g, 243 mmol) and propan-2-amine (43.0 g, 728 mmol) in CH_3_CN (500 mL) was added DIPEA (94.0 g, 728 mmol) at room temperature under a nitrogen atmosphere. After 16 h, the reaction mixture was concentrated under reduced pressure. The residue was dissolved in ethyl acetate, washed with water and brine. The organic layer was dried over anhydrous sodium sulfate, filtered, and the filtrate was concentrated under reduced pressure. The residue was purified by flash column chromatography on silica gel eluting with PE/EtOAc (3/1) to give the title compound (42 g, 76% yield) as a yellow solid. LC-MS *m*/*z* = 229 [M + 1].

##### Step 2: Synthesis of 6-Chloro-4-(isopropylamino)­nicotinic Acid

To a solution of methyl 6-chloro-4-(isopropylamino)­nicotinate (42 g, 183.7 mmol) in THF (200 mL), MeOH (120 mL), and H_2_O (80 mL) was added LiOH.H_2_O (15.4 g, 367 mmol) at 0 °C under a nitrogen atmosphere. The reaction mixture was then allowed to warm to room temperature and stirred for 12 h. The reaction mixture was acidified by adding 2 M HCl (aq) until pH 5–6. The precipitate was collected by filtration, then dried in vacuum to give the title compound (36.5 g, 92% yield) as a white solid which was used for next step without further purification. LC-MS *m*/*z* = 215 [M + 1].

##### Step 3: Synthesis of 6-Chloro-4-(isopropylamino)-*N*-methylnicotinamide

To a solution of methanamine hydrochloride (17.2 g, 255 mmol), Et_3_N (51.5 g, 510 mmol), and 6-chloro-4-(isopropylamino)­nicotinic acid (36.5 g, 170 mmol) in DMF (300 mL) was added HATU (97 g, 255 mmol). Then the reaction mixture was stirred for 12 h at room temperature. The organic layer was washed with water and brine, dried over anhydrous sodium sulfate, filtered, and the filtrate was concentrated under reduced pressure. The residue was purified by flash column chromatography on silica gel eluting with DCM/MeOH (10/1) to give the title compound (26 g, 67% yield) as a white solid. LC-MS *m*/*z* = 228 [M + 1].

##### Step 4: Synthesis of 6-((6-Amino-2-(methylamino)­pyrimidin-4-yl)­amino)-4-(isopropylamino)-*N*-methylnicotinamide

A mixture of *N* ^2^-methylpyrimidine-2,4,6-triamine (97 mg, 0.70 mmol), 6-chloro-4-(isopropylamino)-*N*-methylnicotinamide (80 mg, 0.35 mmol), X-Phos Pd G2 (24 mg, 0.03 mmol), and Cs_2_CO_3_ (343 mg, 1.05 mmol) in dioxane (8 mL) was stirred at 100 °C for 5 h under a nitrogen atmosphere. The reaction mixture was cooled to room temperature and diluted with dioxane, filtered, and the filtrate was directly concentrated under reduced pressure The residue was purified with Prep-HPLC (A:water­(10 mM NH_4_HCO_3_ and 0.025% NH_3_·H2O), B: CAN; 45% B for 1 min, then 60% B in 7 min, stop at 15 min) to obtain the title product (19.5 mg, 17% yield) as a white solid. LC-MS *m*/*z* = 331 [M + 1]; 1H NMR (400 MHz, DMSO-d6) δ ^1^H NMR (500 MHz, DMSO) δ 8.98 (s, 1H), 8.39 (d, *J* = 7.5 Hz, 1H), 8.26 (s, 1H), 8.21 (q, *J* = 4.5 Hz, 1H), 7.42 (s, 1H), 6.02 (s, 1H), 5.93 (s, 2H), 5.74 (s, 1H), 3.65 (h, *J* = 6.5 Hz, 1H), 2.79 (d, *J* = 4.8 Hz, 3H), 2.71 (d, *J* = 4.4 Hz, 3H), 1.19 (d, *J* = 6.3 Hz, 6H).

#### 6-((6-Amino-2-cyclopropylpyrimidin-4-yl)­amino)-4-(isopropylamino)-*N*-methylnicotinamide (11)

##### Step 1: Synthesis of *N*-(*tert*-Butyl)-6-chloro-2-cyclopropylpyrimidin-4-amine

A mixture of 4,6-dichloro-2-cyclopropylpyrimidine (52.0 g, 275 mmol), 2-methylpropan-2-amine (24.2 g, 330 mmol), and *N*,*N*-diisopropylethylamine (106 g, 825 mmol) in NMP (1.0 L) was stirred at 100 °C for 1 h. The reaction mixture was cooled to room temperature and diluted with ethyl acetate, then washed with water and brine. The organic layer was dried over anhydrous sodium sulfate, filtered, and the filtrate was concentrated to dryness under reduced pressure. The resulting crude product was purified by flash column chromatography on silica gel eluting with PE/EtOAc (2/1) to afford the title compound (52.2 g, 87% yield) as a white solid. LC-MS *m*/*z* = 226 [M + 1].

##### Step 2: Synthesis of *N*-(*tert*-Butyl)-2-cyclopropyl-6-((diphenylmethylene)­amino)­pyrimidin-4-amine

A mixture of *N*-(*tert*-butyl)-6-chloro-2-cyclopropylpyrimidin-4-amine (52.2 g, 231 mmol), benzophenone imine­(62.9 g, 346 mmol), Pd_2_(dba)_3_ (21.2 g, 23.1 mmol), XantPhos (13.4 g, 23.1 mol), and Cs_2_CO_3_ (226 g, 694 mmol) in dioxane (1.2 L) was stirred at 100 °C for 16 h under a nitrogen atmosphere. The reaction mixture was cooled to room temperature and diluted with dioxane, filtered, and the filtrate was directly concentrated to dryness under reduced pressure. The crude was purified by flash column chromatography on silica gel eluting with DCM/MeOH (15/1) to afford the title compound (57.5 g, 67% yield) as a yellow solid. LC-MS *m*/*z* = 371 [M + 1].

##### Step 3: Synthesis of *N* ^4^-(*tert*-Butyl)-2-cyclopropylpyrimidine-4,6-diamine

To a solution of methyl *N*-(*tert-*butyl)-2-cyclopropyl-6-((diphenylmethylene)­amino)­pyrimidin-4-amine (57.5 g, 155 mmol) in methanol (600 mL) was added hydroxylamine (30.8 g, 50 wt % in water, 466 mmol) at room temperature under a nitrogen atmosphere. After addition, the reaction mixture was heated to 70 °C and stirred for 12 h. The reaction mixture was concentrated under reduced pressure. The residue was dissolved into DCM and washed with water. The aqueous layer was basified with NaHCO_3_ to pH > 10, then extracted with DCM. The combined organic layers were concentrated under reduced pressure. The resulting crude product was purified by flash column chromatography on silica gel eluting with DCM/MeOH (10/1) to afford the title compound (20.0 g, 62% yield) as a yellow solid. LC-MS *m*/*z* = 207 [M + 1].

##### Step 4: Synthesis of 6-((6-(*tert*-Butylamino)-2-cyclopropylpyrimidin-4-yl)­amino)-4-(isopropylamino)-*N*-methylnicotinamide

A mixture of *N* ^4^-(*tert*-butyl)-2-cyclopropylpyrimidine-4,6-diamine (20.0 g, 97.0 mmol), 6-chloro-4-(isopropylamino)-*N*-methylnicotinamide (22.1 g, 97.0 mmol), Pd­(*t*-Bu_3_P)_2_ (10.0 g, 19.4 mmol), and Cs_2_CO_3_ (94.8 g, 291 mmol) in dioxane (300 mL) was stirred at 100 °C for 16 h under a nitrogen atmosphere. The reaction mixture was cooled to room temperature and diluted with dioxane, filtered, and the filtrate was concentrated under reduced pressure. The residue was purified by flash column chromatography on silica gel eluting with DCM/MeOH (10/1) to afford the title compound (21.5 g, 55% yield) as a yellow solid. LC-MS *m*/*z* = 398 [M + 1].

##### Step 5: Synthesis of 6-((6-Amino-2-cyclopropylpyrimidin-4-yl)­amino)-4-(isopropylamino)-*N*-methylnicotinamide

A mixture of 6-((6-(*tert*-butylamino)-2-cyclopropylpyrimidin-4-yl)­amino)-4-(isopropylamino)-*N*-methylnicotinamide (21.5 g, 54.1 mmol) in TFA (100 mL) was stirred at 70 °C for 16 h. The reaction mixture was concentrated under reduced pressure and basified with a saturated aqueous solution of K_2_CO_3_ to pH ∼ 10. The resulting mixture was concentrated under reduced pressure. The residue was purified by flash column chromatography on silica gel eluting with DCM/MeOH (10/1) to afford the title compound (10.9 g, 59% yield) as a yellow solid. LC-MS *m*/*z* = 342 [M + 1].^1^HNMR (500 MHz, DMSO-*d6*) δ 9.24 (s, 1H), 8.39 (d, *J* = 7.3 Hz, 1H), 8.28 (s, 1H), 8.25 (q, *J* = 4.5 Hz, 1H), 7.21 (s, 1H), 6.32 (s, 2H), 6.29 (s, 1H), 3.62 (h, *J* = 6.5 Hz, 1H), 2.72 (d, *J* = 4.5 Hz, 3H), 1.81 (tt, *J* = 8.1, 4.7 Hz, 1H), 1.22 (d, *J* = 6.3 Hz, 6H), 0.92 (dt, *J* = 5.1, 2.8 Hz, 2H), 0.86 (dt, *J* = 8.2, 3.0 Hz, 2H).

##### 6-((6-Amino-2-(difluoromethyl)­pyrimidin-4-yl)­amino)-4-(isopropylamino)-*N*-methylnicotinamide (12)

The following compound was synthesized following procedures similar to that described for compound 10, step 4 above using 6-chloro-4-(isopropylamino)-*N*-methylnicotinamide and 2-(difluoromethyl)­pyrimidine-4,6-diamine and purifying by flash column chromatography on silica gel eluting with 0–20% MeOH/DCM to afford the title compound (13.8 mg, 6.8% yield) LC-MS *m*/*z* = 352 [M + 1].^1^H NMR (400 MHz, DMSO-*d*_6_) δ 10.35 (s, 1H), 8.82 (s, 1H), 8.42 (s, 1H), 8.33 (s, 1H), 7.05 (s, 2H), 6.72 (d, *J* = 6.2 Hz, 1H), 6.51 (d, J = 54.7 Hz, 1H), 6.31 (s, 1H), 3.74–3.61 (m, 2H), 2.75 (d, *J* = 4.4 Hz, 3H), 1.24 (d, *J* = 6.3 Hz, 6H).

#### (*S*)-6-((6-Amino-2-(difluoromethyl)­pyrimidin-4-yl)­amino)-4-((1-fluoropropan-2-yl)­amino)-*N*-methylnicotinamide (13)

##### Step 1: Synthesis of Methyl (*S*)-6-Chloro-4-((1-fluoropropan-2-yl)­amino)­nicotinate

A mixture of methyl 4,6-dichloropyridine-3-carboxylate (300 mg, 1.45 mmol), (2*S*)-1-fluoropropan-2-amine hydrochloride (246 mg, 2.17 mmol), and DIPEA (561 mg, 4.35 mmol) in dioxane (5 mL) was stirred at 140 °C for 16 h. The cooled reaction was quenched with water and extracted with EtOAc. The organic layer was washed with water and brine, dried over Na_2_SO_4_, filtered, and concentrated. The residue was purified by column chromatography on silica gel eluting with EtOAc/PE (1/2) to get the title compound (200 mg, 53% yield) as a white solid. LC-MS *m*/*z* = 247 [M + 1].

##### Step 2: Synthesis of (*S*)-6-Chloro-4-((1-fluoropropan-2-yl)­amino)­nicotinic acid

A mixture of methyl (*S*)-6-chloro-4-((1-fluoropropan-2-yl)­amino)­nicotinate (200 mg, 0.81 mmol), LiOH (57.9 mg, 2.42 mmol), and H_2_O (1 mL) in THF (4 mL) was stirred at room temperature for 16 h. HCl (1 N) was added to adjust the pH ∼ 3, the mixture was diluted with water and extracted with EtOAc. The combined organic layers were dried over Na_2_SO_4_, filtered, and concentrated to give the title compound (200 mg, 100% yield) as a white solid used as is without purification. LC-MS *m*/*z* = 233 [M + 1].

##### Step 3: Synthesis of (*S*)-6-Chloro-4-((1-fluoropropan-2-yl)­amino)-*N*-methyl nicotinamide

A mixture of 4,6-dichloro-*N*-methylnicotinamide (200 mg, 0.975 mmol), (2*S*)-1-fluoropropan-2-amine hydrochloride (110 mg, 0.975 mmol), and DIPEA (376 mg, 2.92 mmol) in dioxane (5 mL) was stirred in a sealed tube at 140 °C for 16 h. The reaction was quenched with H_2_O and extracted into EtOAc. The combined organics were washed (H_2_O and brine), dried (Na_2_SO_4_), and concentrated under reduced pressure. The residue was purified by column chromatography on silica gel (10:1 to 1:1 PE/EtOAc) to afford the title compound (210 mg, 82% yield) as a white solid. LC-MS *m*/*z* = 246 [M + 1].

##### Step 4: Synthesis of (*S*)-6-((6-Amino-2-(difluoromethyl)­pyrimidin-4-yl)­amino)-4-((1-fluoropropan-2-yl)­amino)-*N*-methylnicotinamide

A mixture of (*S*)-6-chloro-4-((1-fluoropropan-2-yl)­amino)-*N*-methyl nicotinamide (150 mg, 610 μmol), 2-(difluoromethyl)­pyrimidine-4,6-diamine (117 mg, 731 μmol), Cs_2_CO_3_ (397 mg, 1.22 mmol), and Pd­(*t*Bu_3_P)_2_ (155 mg, 305 μmol) in dioxane (4 mL) was stirred at 100 °C for 16 h under N_2_. The reaction mixture was purified by flash chromatography on silica gel eluting with MeOH/DCM 1:10 then by Prep-HPLC (Mobile phase: A = water­(0.1% NH4HCO3), B = acetonitrile; Gradient: B = 15–95% in 18 min; Column: Xtimate 10um 150A 21.2 × 250 mm) to afford the title compound (41 mg, 18% yield) as a white solid. LC-MS *m*/*z* = 370 [M + 1]. 1H-NMR (400 MHz, DMSO-d6) δ^1^H NMR (500 MHz, DMSO) δ 9.69 (s, 1H), 8.55 (d, *J* = 7.6 Hz, 1H), 8.35 (d, *J* = 6.1 Hz, 2H), 7.11 (s, 1H), 6.91 (s, 2H), 6.81 (s, 1H), 6.49 (t, *J* = 54.9 Hz, 1H), 4.53 (qd, *J* = 9.4, 4.5 Hz, 1H), 4.43 (qd, *J* = 9.3, 4.5 Hz, 1H), 3.82–3.66 (m, 1H), 2.73 (d, *J* = 4.4 Hz, 3H), 1.23 (dd, *J* = 6.5, 1.2 Hz, 3H).

##### 2-(Difluoromethyl)-*N* ^4^-(4-isopropoxy-5-(1-methyl-1*H*-pyrazol-4-yl)­pyridin-2-yl)­pyrimidine-4,6-diamine (14)

To a mixture of 2-(difluoromethyl)­pyrimidine-4,6-diamine hydrochloride (**SI-4**) (25.00 mg, 156.13 mmol,) and 2-chloro-4-isopropoxy-5-(1-methyl-1*H*-pyrazol-4-yl)­pyridine (**SI-1**) (39.30 mg, 156.13 mmol) in dioxane (2.00 mL) was added BrettPhos Pd G_4_ (14.37 mg, 15.61 mmol) and Cs_2_CO_3_ (101.74 mg, 312.26 mmol). The reaction was heated at 90 °C for 2 h under N_2_. The reaction mixture was concentrated under reduced pressure then purified by prep-HPLC (column: Phenomenex Gemini-NX C18 75 × 30 mm × 3 μm;mobile phase: [water­(0.04%NH_3_H_2_O + 10 mM NH_4_HCO_3_)-ACN]; B%: 25–55%, 8 min) to give the title compound (22.40 mg, 37.1% yield) as a white solid. LC-MS *m*/*z* = 376 [M + 1]; ^1^H NMR (500 MHz, DMSO) δ 9.73 (s, 1H), 8.34 (s, 1H), 8.03 (s, 1H), 7.88 (d, *J* = 0.8 Hz, 1H), 7.40 (s, 1H), 6.91 (d, *J* = 5.0 Hz, 3H), 6.50 (t, *J* = 54.9 Hz, 1H), 4.68 (p, *J* = 6.0 Hz, 1H), 3.88 (s, 3H), 1.41 (d, *J* = 6.0 Hz, 6H).

#### 2-(Difluoromethyl)-*N* ^4^-(4-methoxy-5-(1-methyl-1*H*-pyrazol-4-yl)­pyridin-2-yl)­pyrimidine-4,6-diamine (15)

##### Step 1: Synthesis of 2-Chloro-4-fluoro-5-(4,4,5,5-tetramethyl-1,3,2-dioxaborolan-2-yl)­pyridine

A mixture of 2-chloro-4-fluoropyridine (10 g, 76.0 mmol), bis­(pinacolato)­diboron (9.64 g, 38.0 mmol), [Ir­(OMe)­(1,5-cod)]_2_ (251 mg, 0.380 mmol), and dtbpy (270 mg, 0.760 mmol) in THF (150 mL) was stirred at 80 °C for 16 h. The mixture was concentrated in vacuo and the residue was purified by column chromatography on silica gel eluting with EtOAc/PE (1/10) to afford the title compound (15 g, 77% yield) as colorless oil. LC-MS *m*/*z* = 258 [M + 1].

##### Step 2: Synthesis of 2-Chloro-4-fluoro-5-(1-methyl-1*H*-pyrazol-4-yl)­pyridine

A mixture of 2-chloro-4-fluoro-5-(4,4,5,5-tetramethyl-1,3,2-dioxaborolan-2-yl)­pyridine (15 g, 58.36 mmol), 4-bromo-1-methyl-1*H*-pyrazole (9.4 g, 58.36 mmol), K_2_CO_3_ (16.1 g, 116.73 mmol), and Pd­(dppf)­Cl_2_ (4.27 g, 5.83 mmol) in dioxane/water (80 mL/20 mL) was stirred at 80 °C for 6 h. The mixture was concentrated in vacuo and the residue was purified by column chromatography on silica gel eluting with EtOAc/PE (1/2) to afford the title compound (5 g, 41% yield) as a brown solid. ^1^H NMR (400 MHz, DMSO-*d*_6_) δ ppm 8.83–8.80 (m, 1H), 8.27–8.26 (m, 1H), 8.00 (s, 1H), 7.69–7.67 (m, 1H), 3.90 (s, 3H).

##### Step 3: Synthesis of 2-Chloro-4-methoxy-5-(1-methyl-1*H*-pyrazol-4-yl)­pyridine

To a mixture of 2-chloro-4-fluoro-5-(1-methyl-1*H*-pyrazol-4-yl)­pyridine (73.7 mg, 0.3 mmol) and MeOH (14.4 mg, 0.45 mmol) in THF (4 mL) was added *t*-BuOK (101 mg, 0.9 mmol) and the mixture was stirred at 100 °C for 3 h. The reaction mixture was concentrated by Speedvac to afford 2-chloro-4-methoxy-5-(1-methyl-1*H*-pyrazol-4-yl)­pyridine which was used in the following step without further purification.

##### Step 4: Synthesis of 2-(Difluoromethyl)-*N* ^4^-(4-methoxy-5-(1-methyl-1*H*-pyrazol-4-yl)­pyridin-2-yl)­pyrimidine-4,6-diamine

To a solution 2-chloro-4-methoxy-5-(1-methyl-1*H*-pyrazol-4-yl)­pyridine (0.27 mmol) and 2-(difluoromethyl)­pyrimidine-4,6-diamine (51.8 g, 0.32 mmol) in *t*-AmOH (3 mL) was added Cs_2_CO_3_ (263.9 g, 0.81 mmol) and BrettPhos Pd G3 (12.24 mg, 0.014 mmol) and the mixture was stirred at 120 °C for 2 h under N_2_. The reaction was diluted with H_2_O (3.0 mL) and extracted with EtOAc (3 × 10 mL). The combined organic layers were evaporated to dryness by Speedvac and the residue purified by prep-HPLC-C to give the title compound (14 mg, 9% yield); LC-MS *m*/*z* = 348 [M + 1]; ^1^H NMR (500 MHz, DMSO) δ 9.79 (s, 1H), 8.34 (s, 1H), 8.06 (s, 1H), 7.87 (d, *J* = 0.8 Hz, 1H), 7.35 (s, 1H), 6.96 (s, 1H), 6.90 (s, 2H), 6.50 (t, *J* = 54.9 Hz, 1H), 3.89 (s, 3H), 3.86 (s, 3H).

#### 2-(Difluoromethyl)-*N* ^4^-(4-methoxy-5-(1-(2-(methylamino)­ethyl)-1*H*-pyrazol-4-yl)­pyridin-2-yl)­pyrimidine-4,6-diamine (16)

##### Step 1: Synthesis of 5-Bromo-2-chloro-4-methoxypyridine

To a solution of MeOH (704 mg, 22.0 mmol) in THF (30 mL) at 0 °C was added NaH (60% dispersion, 1.76 g, 44.0 mmol). The mixture was stirred at 0 °C for 30 min, then 5-bromo-2,4-dichloropyridine (5 g, 22.0 mmol) in THF (10 mL) was added. The reaction was stirred at room temperature for 12 h. The solution was quenched with H_2_O, extracted with EtOAc, and the combined organic layers were concentrated in vacuo. The residue was purified by silica gel chromatography to give the title compound as a white solid (3.5 g, 72%). LC-MS *m*/*z* = 222 [M + H]^+^.

##### Step 2: Synthesis of *tert*-Butyl Methyl­(2-(4-(4,4,5,5-tetramethyl-1,3,2-dioxaborolan-2-yl)-1*H*-pyrazol-1-yl)­ethyl)­carbamate

To a solution of 4-(4,4,5,5-tetramethyl-1,3,2-dioxaborolan-2-yl)-1*H*-pyrazole (4.50 g, 23.19 mmol) and *tert*-butyl *N*-(2-hydroxyethyl)-*N*-methyl-carbamate (4.06 g, 23.19 mmol) in THF (30 mL) was added DIAD (5.16 g, 25.51 mmol) and PPh_3_ (6.69 g, 25.51 mmol). The reaction was stirred at room temperature for 12 h under N_2_. The reaction mixture was concentrated under reduced pressure and the residue was purified by prep-HPLC-A to give the title compound (2.11 g, 25.9%) as a yellow oil.

##### Step 3: Synthesis of *tert*-Butyl­(2-(4-(6-chloro-4-methoxypyridin-3-yl)-1*H*-pyrazol-1-yl)­ethyl)­(methyl)­carbamate

To a solution of 5-bromo-2-chloro-4-methoxypyridine (46 mg, 0.207 mmol) and *tert*-butyl methyl­(2-(4-(4,4,5,5-tetramethyl-1,3,2-dioxaborolan-2-yl)-1*H*-pyrazol-1-yl)­ethyl)­carbamate (79.89 mg, 0.227 mmol) in EtOH (3 mL) and H_2_O (0.20 mL) was added Pd­(amphos)­Cl_2_ (14.64 mg, 0.0207 mmol) and KOAc (40.59 mg, 0.413 mmol). The reaction mixture was stirred at 80 °C for 2 h under N_2_. The cooled reaction mixture was concentrated under reduced pressure and the residue was purified by prep-TLC (PE:EtOAc = 0:1) to give the title compound (50 mg, 65.9%) as a yellow oil.

##### Step 4: Synthesis of *tert*-Butyl­(2-(4-(6-((6-amino-2-(difluoromethyl)­pyrimidin-4-yl)­amino)-4-methoxypyridin-3-yl)-1*H*-pyrazol-1-yl)­ethyl)­(methyl)­carbamate

The following compound was synthesized following procedures similar to that described for compound **15**, step 4 above using *tert*-butyl­(2-(4-(6-chloro-4-methoxypyridin-3-yl)-1*H*-pyrazol-1-yl)­ethyl)­(methyl)­carbamate to afford the title compound which was used as is in the next without further purification.

##### Step 5: Synthesis of 2-(Difluoromethyl)-*N* ^4^-(4-methoxy-5-(1-(2-(methylamino)­ethyl)-1*H*-pyrazol-4-yl)­pyridin-2-yl)­pyrimidine-4,6-diamine

To a solution of *tert*-butyl (2-(4-(6-((6-amino-2-(difluoromethyl)­pyrimidin-4-yl)­amino)-4-methoxypyridin-3-yl)-1*H*-pyrazol-1-yl)­ethyl)­(methyl)­carbamate in DCM (2 mL) was added TFA (1 mL, 13.51 mmol) and the reaction was stirred at room temperature for 1 h. The reaction mixture was concentrated under reduced pressure to give a residue and then adjusted with NH_3_.H_2_O to pH 5–6 to give the title compound (9.80 mg, 24.6%) as a pale yellow solid. LC-MS *m*/*z* = 391 [M + H]^+^.^1^H NMR (400 MHz, DMSO) δ 9.80 (s, 1H), 8.36 (s, 1H), 8.10 (s, 1H), 7.90 (s, 1H), 7.38 (s, 1H), 6.97 (s, 1H), 6.91 (s, 2H), 6.52 (t, *J* = 54.8 Hz, 1H), 4.19 (t, *J* = 6.3 Hz, 2H), 3.90 (s, 3H), 2.89 (t, *J* = 6.3 Hz, 2H), 2.30 (s, 3H).

#### 1-(4-(6-((6-Amino-2-(difluoromethyl)­pyrimidin-4-yl)­amino)-4-methoxypyridin-3-yl)-1*H*-pyrazol-1-yl)-2-methylpropan-2-ol (17)

##### Step 1: Synthesis of 1-(4-(6-Chloro-4-methoxypyridin-3-yl)-1*H*-pyrazol-1-yl)-2-methylpropan-2-ol

A mixture of 2-chloro-5-iodo-4-methoxypyridine (Step 1 of compound **16**), 2-methyl-1-(4-(4,4,5,5-tetramethyl-1,3,2-dioxaborolan-2-yl)-1*H*-pyrazol-1-yl)­propan-2-ol (891 mg, 3.35 mmol), Pd­(dppf)­Cl_2_ (204 mg, 0.279 mmol), and K_2_CO_3_ (770 mg, 5.58 mmol) in dioxane (12 mL) and H_2_O (3 mL) was stirred at 90 °C for 4 h under N_2_. The cooled mixture was concentrated in vacuo to give the crude product which was purified by silica gel chromatography (PE:EtOAc = 1:2) to give the title compound (560 mg, 71.5%) as a yellow oil. LC-MS *m*/*z* = 282 [M + 1].

##### Step 2: Synthesis of 1-(4-(6-((2-(Difluoromethyl)-6-((2,4-dimethoxybenzyl)­amino) pyrimidin-4-yl)­amino)-4-methoxypyridin-3-yl)-1*H*-pyrazol-1-yl)-2-methylpropan-2-ol

A mixture of 1-(4-(6-chloro-4-methoxypyridin-3-yl)-1*H*-pyrazol-1-yl)-2-methylpropan-2-ol (560 mg, 1.99 mmol), 2-(difluoromethyl)-*N* ^4^-(2,4-dimethoxybenzyl)­pyrimidine-4,6-diamine (679 mg, 2.19 mmol, **SI-3**), Pd­(*t*-Bu_3_P)_2_ (200 mg, 0.392 mmol), and Cs_2_CO_3_ (1.29 g, 3.98 mmol) in dioxane (12 mL) was stirred at 100 °C for 16 h under N_2_. The mixture was poured into water (80 mL) and extracted with EtOAc (100 mL × 3). The combined organic layers were concentrated in vacuo and the crude product was purified by silica gel chromatography (PE:EtOAc = 1:10) to give the title compound (480 mg, 43.6%) as a yellow oil. LC-MS *m*/*z* = 556 [M + 1].

##### Step 3: Synthesis of 1-(4-(6-((6-Amino-2-(difluoromethyl)­pyrimidin-4-yl)­amino)-4-methoxypyridin-3-yl)-1*H*-pyrazol-1-yl)-2-methylpropan-2-ol

A solution of 1-(4-(6-((2-(difluoromethyl)-6-((2,4-dimethoxybenzyl)­amino)­pyrimidin-4-yl)­amino)-4-methoxypyridin-3-yl)-1*H*-pyrazol-1-yl)-2-methylpropan-2-ol (480 mg, 0.86 mmol) in TFA (2 mL) and DCM (2 mL) was stirred at room temperature for 2 h. The mixture was concentrated in vacuo and a saturated aqueous NaHCO_3_ solution (20 mL) was added. The precipitate was filtered and the solid was dissolved in MeOH (30 mL). The precipitate was filtered, and the filtrate was concentrated to give the crude product which was washed with DCM. The precipitate was filtered to provide the title compound (294 mg, 84%) as a light yellow solid. LC-MS *m*/*z* = 406 [M + 1].^1^HNMR (500 MHz, DMSO-*d*_6_) δ 10.56 (s, 1H), 8.46 (s, 1H), 8.12 (s, 1H), 7.93 (s, 1H), 7.22 (s, 2H), 6.82–6.47 (m, 2H), 4.06 (s, 2H), 4.00 (s, 3H), 1.09 (s, 6H).

#### (*S*)-1-(4-(6-((6-Amino-2-(1-fluoroethyl)­pyrimidin-4-yl)­amino)-4-isopropoxypyridin-3-yl)-1*H*-pyrazol-1-yl)-2-methylpropan-2-ol (18)

##### Step 1: Synthesis of 2-(1-Fluoroethyl)­pyrimidine-4,6-diol (21)

To a solution of malonamide (30 g, 294 mmol) in EtOH (300 mL) was added EtONa (40.0 g, 588 mmol) and the solution stirred at 20 °C for 1 h. Ethyl 2-fluoropropanoate (38.7 g, 323 mmol) was added dropwise and the reaction stirred at 100 °C for 2 h. The pH of the mixture was adjusted to pH = 6 with 1 N HCl, and then concentrated in vacuo. The residue was adjusted to pH ∼ 2 with 1 N HCl and filtered to provide the title compound (31 g, 66% yield) as a yellow solid, which was used in the next step without further purification. LC-MS *m*/*z* = 159 [M + 1].

##### Step 2: Synthesis of 4,6-Dichloro-2-(1-fluoroethyl)­pyrimidine (22)

To a stirred solution of 2-(1-fluoroethyl)­pyrimidine-4,6-diol (31 g, 196 mmol) in toluene (300 mL) was added POCl_3_ (72.8 mL, 784 mmol) at room temperature. Triethylamine (54.4 mL, 392 mmol) was added dropwise and the reaction stirred at 100 °C for 2 h. The reaction mixture was poured into warm water and extracted with EtOAc (×3). The combined organic layers were washed with brine, dried over Na_2_SO_4_, filtered, and the filtrate was evaporated under reduced pressure to give the title compound (40 gcrude) directly used in the next step without further purification.

##### Step 3: Synthesis of 6-Chloro-*N*-(2,4-dimethoxybenzyl)-2-(1-fluoroethyl)­pyrimidin-4-amine (23)

A solution of 4,6-dichloro-2-(1-fluoroethyl)­pyrimidine (40 g, 196 mmol), 2,4-dimethoxybenzylamine (32.7 g, 196 mmol), and DIPEA (50.5 g, 392 mmol) in NMP (150 mL) was stirred at 100 °C for 1 h. The mixture was poured into water (500 mL) and extracted with EtOAc (300 mL × 3). The combined organics were washed with brine (300 mL × 3), dried over Na_2_SO_4_, and evaporated under reduced pressure to give the title compound (22 g, crude) as a yellow solid, which was used in the next step directly. LC-MS *m*/*z* = 326 [M + 1].

##### Step 4: Synthesis of 6-Chloro-2-(1-fluoroethyl)­pyrimidin-4-amine (24)

A solution of 6-chloro-*N*-(2,4-dimethoxybenzyl)-2-(1-fluoroethyl)­pyrimidin-4-amine (22 g, 67.6 mmol) in HCl/EtOAc (4.0 M, 85 mL) was stirred at room temperature for 12 h. The mixture was concentrated in vacuo, the pH of the residue adjusted to pH ∼ 8 with saturated NaHCO_3_ (aq) solution, and the mixture extracted with DCM (300 mL × 3). The combined organic layers were washed with brine (300 mL), dried over Na_2_SO_4_, and concentrated in vacuo. The residue was triturated with PE:EtOAc (1:1) and filtered to give the title compound (10 g, 84% yield over 3 steps) as a gray solid. LC-MS *m*/*z* = 176 [M + 1].

##### Step 5: Synthesis of (*S*)-*N* ^4^-(2,4-Dimethoxybenzyl)-2-(1-fluoroethyl)­pyrimidine-4,6-diamine (25) and (*R*)-*N* ^4^-(2,4-Dimethoxybenzyl)-2-(1-fluoroethyl)­pyrimidine-4,6-diamine

A solution of 6-chloro-2-(1-fluoroethyl)­pyrimidin-4-amine (10.0 g, 57 mmol), 2,4-dimethoxybenzylamine (9.5 g, 57 mmol), and DIPEA (29.3 g, 228 mmol) in NMP (60 mL) was stirred at 140 °C for 2 h. The cooled mixture was poured into water (500 mL), extracted with EtOAc (300 mL × 3), and the combined organic layers were washed with brine (300 mL × 2), dried over Na_2_SO_4_, and concentrated. The residue was purified by silica gel chromatography to provide the racemic mixture (6.6 g, 37% yield) as a yellow solid. This product was further purified by SFC, using an OZ 20 × 250 mm, 10 μm (Daicel) column, eluting with 35% MeOH (0.2% MeOH/NH_3_) at 100 g/min, to obtain the first eluting enantiomer: (*S*)-*N* ^4^-(2,4-dimethoxybenzyl)-2-(1-fluoroethyl)­pyrimidine-4,6-diamine (2.1 g, **25**) as a yellow solid. LC-MS *m*/*z* = 307 [M + 1]. Further elution provided the second enantiomer, (*R*)-*N* ^4^-(2,4-dimethoxybenzyl)-2-(1-fluoroethyl)­pyrimidine-4,6-diamine.

##### Step 6: Synthesis of 1-(4-(6-Chloro-4-isopropoxypyridin-3-yl)-1*H*-pyrazol-1-yl)-2-methylpropan-2-ol (28)

A mixture of 5-bromo-2-chloro-4-isopropoxypyridine (2.0 g, 8.06 mmol, **27**), 2-methyl-1-(4-(4,4,5,5-tetramethyl-1,3,2-dioxaborolan-2-yl)-1*H*-pyrazol-1-yl)­propan-2-ol (2.15 g, 8.06 mmol), K_2_CO_3_ (3.34 g, 24.2 mmol), and Pd­(dppf)­Cl_2_ (592 mg, 0.81 mmol) in dioxane/water (20/4 mL) was stirred at 80 °C for 3 h. The cooled mixture was concentrated in vacuo and the residue was purified by column chromatography on silica gel (PE/EtOAc = 10/1 to 2/1) to afford the title compound (2.2 g, 88% yield) as a yellow solid. LC-MS *m*/*z* = 310 [M + 1].

##### Step 7: Synthesis of (*S*)-1-(4-(6-((6-((2,4-Dimethoxybenzyl)­amino)-2-(1-fluoroethyl)­pyrimidin-4-yl)­amino)-4-isopropoxypyridin-3-yl)-1*H*-pyrazol-1-yl)-2-methylpropan-2-ol (29)

A mixture of 1-(4-(6-chloro-4-isopropoxypyridin-3-yl)-1*H*-pyrazol-1-yl)-2-methylpropan-2-ol (800 mg, 2.59 mmol, **28**), (*S*)-*N* ^4^-(2,4-dimethoxybenzyl)-2-(1-fluoroethyl)­pyrimidine-4,6-diamine (800 mg, 2.59 mmol, **25**), BrettPhos Pd G4 (397 mg, 0.26 mmol), and Cs_2_CO_3_ (2.54 g, 7.77 mmol) in dioxane (20 mL) was stirred at 100 °C for 16 h. The cooled mixture was concentrated *in vacuo* and the residue was purified by column chromatography on silica gel (EtOAc/MeOH = 10/1 to 3/1) to afford the title compound (840 mg, 56% yield) as a yellow solid. LC-MS *m*/*z* = 580 [M + 1].

##### Step 8: Synthesis of (*S*)-1-(4-(6-((6-Amino-2-(1-fluoroethyl)­pyrimidin-4-yl)­amino)-4-isopropoxypyridin-3-yl)-1*H*-pyrazol-1-yl)-2-methylpropan-2-ol (18)

To a solution of (*S*)-1-(4-(6-((6-((2,4-dimethoxybenzyl)­amino)-2-(1-fluoroethyl)­pyrimidin-4-yl)­amino)-4-isopropoxypyridin-3-yl)-1*H*-pyrazol-1-yl)-2-methylpropan-2-ol (840 mg, 1.45 mmol, **29**) in DCM (5 mL) was added TFA (10 mL) and the reaction stirred at room temperature for 4 h. The mixture was concentrated in vacuo and the residue was purified by prep HPLC-B to afford the title compound (421.1 mg, 67% yield) as a white solid. LC-MS *m*/*z*= 430 [M + 1]; ^1^H NMR (500 MHz, DMSO) δ 9.55 (s, 1H), 8.35 (s, 1H), 8.06 (d, *J* = 0.8 Hz, 1H), 7.89 (d, *J* = 0.7 Hz, 1H), 7.56 (s, 1H), 6.69 (s, 1H), 6.63 (s, 2H), 5.31 (dq, *J* = 48.5, 6.5 Hz, 1H), 4.76–4.66 (m, 2H), 4.05 (s, 2H), 1.58 (dd, *J* = 24.2, 6.5 Hz, 3H), 1.41 (dd, *J* = 6.0, 1.5 Hz, 6H), 1.09 (s, 6H).

## Supplementary Material





## Notes

General considerations: synthesis; synthesis and characterization of key intermediates; synthesis and characterization of compounds **1–18**; purity analysis for compound **18**; Kinome data for compound 18; crystallography; biology; quality control of tested compounds and molecular formula strings; in vivo pharmacokinetics and pharmacodynamics studies; spectroscopic data of compounds **1–18**; NMR spectra of compounds **1–18** (PDF)
Molecular formula strings (CSV)

## Notes

Crystal structures have been deposited in the RCSB PDB with accession codes 9Z1L and 9Z2S, and coordinates will be released upon publication.

## Abbreviations

ACN: acetonitrile
AcOH: acetic acid
BLK: B-lymphocyte kinase
BMPR2: bone morphogenetic protein receptor type 2
BrettPhos Pd G2: chloro­(2-dicyclohexylphosphino-3,6-dimethoxy-2′,4′,6′-triisopropyl-1,1′-biphenyl)­(2′-amino-1,1′-biphenyl-2-yl)­palladium­(II)
BrettPhos: palladacycle
BrettPhos Pd G3: [(2-dicyclohexylphosphino-3,6-dimethoxy-2′,4′,6′-triisopropyl-1,1′-biphenyl)-2-(2′-amino-1,1′-biphenyl)]­palladium­(II) methanesulfonate
BrettPhos Pd G4: (SP-4-3)-dicyclohexyl­[3,6-dimethoxy-2′,4′,6′-tris­(1-methylethyl)­[1,1′-biphenyl]-2-yl]­phosphine-κP­[2′-(methylamino-κN)­[1,1′-biphenyl]-2-yl-κC]
Cs_2_CO_3_: cesium carbonate
DCM: dichloromethane
DIAD: diisopropyl azodicarboxylate
Dioxane: 1,4-dioxane
DIPEA: *N*,*N*-diisopropylethylamine
DMF: dimethylformamide
DMSO: dimethyl sulfoxide
EtOAc: ethyl acetate
EtOH: ethanol
EtONa: sodium ethanolate
h: hr, hrs, hours
H_2_O: water
HATU: [dimethylamino­(triazolo­[4,5-*b*]­pyridin-3-yloxy)­methylidene]-dimethylazanium hexafluorophosphate
Hck: hematopoietic cell kinase
HCl: hydrogen chloride
HPLC: high-performance liquid chromatography
I4KB: Phosphatidylinositol 4-kinase beta
KF: potassium fluoride
Lck: lymphocyte-specific protein tyrosine kinase
LC-MS: liquid chromatography–mass spectrometry
MeOH: methanol
MINK: misshapen/NIK-related kinase
Na_2_CO_3_: sodium carbonate, anhydrous
Na_2_SO_4_: sodium sulfate
NaH: sodium hydride
NaHCO_3_: sodium hydrogen carbonate
NMR: nuclear magnetic resonance
Pd­(PPh_3_)_4_: tetrakis­(triphenylphosphine)­palladium
Pd_2_(dba)_3_: tris­(dibenzylidene-acetone)­dipalladium­(0)
PE: petroleum ether
pKIT: phosphorylated KIT
pPDGFRb: phosphorylated pdgfrb
ppm: parts-per million
rt/RT: room temperature
Speedvac: Savant SC250EXP speedvac concentrator
T3P: 2,4,6-tripropyl-1,3,5,2λ^5^,4λ^5^,6λ^5^-trioxatriphosphinane 2,4,6-trioxide
t-AmOH: tertiary amyl alcohol
*t*-BuOK: potassium 2-methylpropan-2-olate
TEA: triethylamine
TFA: trifluoroacetic acid
THF: tetrahydrofuran
Toluene: toluene
WT: weight
XantPhos: (5-diphenylphosphanyl-9,9-dimethylxanthen-4-yl)-diphenylphosphane

## References

[1] Patel N., Benipal B. (2019). Incidence of Gastrointestinal Stromal Tumors in the
United States from 2001–2015: A United States Cancer Statistics
Analysis of 50 States. Cureus.

[2] Antonescu C. R. (2011). The GIST
Paradigm: Lessons for Other Kinase-Driven Cancers. J. Pathol..

[3] Lennartsson J., Rönnstrand L. (2012). Stem Cell
Factor Receptor/c-Kit: From Basic Science
to Clinical Implications. Physiol. Rev..

[4] Manley P. W., Cowan-Jacob S. W., Buchdunger E., Fabbro D., Fendrich G., Furet P., Meyer T., Zimmermann J. (2002). Imatinib:
A Selective Tyrosine Kinase Inhibitor. Eur.
J. Cancer.

[5] Lopes L. F., Bacchi C. E. (2010). Imatinib Treatment
for Gastrointestinal Stromal Tumour
(GIST). J. Cell. Mol. Med..

[6] Ma G. L., Murphy J. D., Martinez M. E., Sicklick J. K. (2015). Epidemiology of
Gastrointestinal Stromal Tumors in the Era of Histology Codes: Results
of a Population-Based Study. Cancer Epidemiol.
Biomarkers Prev..

[7] Mohammadi M., Roets E., Bleckman R. F., Oosten A. W., Grunhagen D., Desar I. M. E., Bonenkamp H., Reyners A. K. L., van
Etten B., Hartgrink H., Fiocco M., Schrage Y., Steeghs N., Gelderblom H. (2025). Impact of Mutation Profile on Outcomes
of Neoadjuvant Therapy in GIST. Cancers (Basel)..

[8] Corless C. L., Fletcher J. A., Heinrich M. C. (2004). Biology
of Gastrointestinal Stromal
Tumors. J. Clin. Oncol..

[9] Heinrich M. C., Corless C. L., Demetri G. D., Blanke C. D., von Mehren M., Joensuu H., McGreevey L. S., Chen C.-J., Van den
Abbeele A. D., Druker B. J., Kiese B., Eisenberg B., Roberts P. J., Singer S., Fletcher C. D. M., Silberman S., Dimitrijevic S., Fletcher J. A. (2003). Kinase Mutations and Imatinib Response
in Patients With Metastatic Gastrointestinal Stromal Tumor. J. Clin. Oncol..

[10] Antonescu C. R., Besmer P., Guo T., Arkun K., Hom G., Koryotowski B., Leversha M. A., Jeffrey P. D., Desantis D., Singer S., Brennan M. F., Maki R. G., DeMatteo R. P. (2005). Acquired
Resistance to Imatinib in Gastrointestinal Stromal Tumor Occurs through
Secondary Gene Mutation. Clin. Cancer Res..

[11] Heinrich M. C., Corless C. L., Blanke C. D., Demetri G. D., Joensuu H., Roberts P. J., Eisenberg B. L., von Mehren M., Fletcher C. D. M., Sandau K., McDougall K., Ou W. b., Chen C. J., Fletcher J. A. (2006). Molecular Correlates
of Imatinib Resistance in Gastrointestinal Stromal Tumors. J. Clin. Oncol..

[12] Wardelmann E., Merkelbach-Bruse S., Pauls K., Thomas N., Schildhaus H. U., Heinicke T., Speidel N., Pietsch T., Buettner R., Pink D., Reichardt P., Hohenberger P. (2006). Polyclonal
Evolution of Multiple Secondary KIT Mutations in Gastrointestinal
Stromal Tumors under Treatment with Imatinib Mesylate. Clin. Cancer Res..

[13] Nishida T., Kanda T., Nishitani A., Takahashi T., Nakajima K., Ishikawa T., Hirota S. (2008). Secondary Mutations
in the Kinase Domain of the *KIT* Gene Are Predominant
in Imatinib-resistant Gastrointestinal Stromal Tumor. Cancer Sci..

[14] Desai J., Shankar S., Heinrich M. C., Fletcher J. A., Fletcher C. D., Manola J., Morgan J. A., Corless C. L., George S., Tuncali K., Silverman S. G., Van den Abbeele A. D., van Sonnenberg E., Demetri G. D. (2007). Clonal Evolution
of Resistance to
Imatinib in Patients with Metastatic Gastrointestinal Stromal Tumors. Clin. Cancer Res..

[15] Kee D., Zalcberg J. R. (2012). Current and Emerging
Strategies for the Management
of Imatinib-Refractory Advanced Gastrointestinal Stromal Tumors. Ther. Adv. in Med. Oncol..

[16] Hao Z., Sadek I. (2016). Sunitinib: The Antiangiogenic
Effects and Beyond. Onco. Targets Ther..

[17] Farag S., Smith M. J., Fotiadis N., Constantinidou A., Jones R. L. (2020). Revolutions in Treatment Options
in Gastrointestinal
Stromal Tumours (GISTs): The Latest Updates: Revolutions in Treatment
Options in GIST. Curr. Treat. Opti. Oncol..

[18] Demetri G. D., Reichardt P., Kang Y. K., Blay J. Y., Rutkowski P., Gelderblom H., Hohenberger P., Leahy M., von Mehren M., Joensuu H., Badalamenti G., Blackstein M., Le Cesne A., Schöffski P., Maki R. G., Bauer S., Nguyen B. B., Xu J., Nishida T., Chung J., Kappeler C., Kuss I., Laurent D., Casali P. G. (2013). Efficacy
and Safety of Regorafenib for Advanced Gastrointestinal Stromal Tumours
After Failure of Imatinib and Sunitinib (GRID): An International,
Multicentre, Randomised, Placebo-Controlled, Phase 3 Trial. Lancet.

[19] Demetri G. D., van Oosterom A. T., Garrett C. R., Blackstein M. E., Shah M. H., Verweij J., McArthur G., Judson I. R., Heinrich M. C., Morgan J. A., Desai J., Fletcher C. D., George S., Bello C. L., Huang X., Baum C. M., Casali P. G. (2006). Efficacy and Safety
of Sunitinib in Patients with Advanced
Gastrointestinal Stromal Tumour After Failure of Imatinib: A Randomised
Controlled Trial. Lancet.

[20] Blay J. Y., Serrano C., Heinrich M. C., Zalcberg J., Bauer S., Gelderblom H., Schoffski P., Jones R. L., Attia S., D’Amato G., Chi P., Reichardt P., Meade J., Shi K., Ruiz-Soto R., George S., von Mehren M. (2020). Ripretinib in Patients with Advanced
Gastrointestinal Stromal Tumours (INVICTUS): A Double-Blind, Randomised,
Placebo-Controlled, Phase 3 Trial. Lancet Oncol..

[21] Kettle J. G., Anjum R., Barry E., Bhavsar D., Brown C., Boyd S., Campbell A., Goldberg K., Grondine M., Guichard S., Hardy C. J., Hunt T., Jones R. D. O., Li X., Moleva O., Ogg D., Overman R. C., Packer M. J., Pearson S., Schimpl M., Shao W., Smith A., Smith J. M., Stead D., Stokes S., Tucker M., Ye Y. (2018). Discovery of *N* -(4-{[5-Fluoro-7-(2-Methoxyethoxy)­Quinazolin-4-yl]­Amino}­phenyl)-2-[4-(Propan-2-yl)-1*H*-1,2,3-Triazol-1-yl]­Acetamide (AZD3229), a Potent Pan-KIT
Mutant Inhibitor for the Treatment of Gastrointestinal Stromal Tumors. J. Med. Chem..

[22] Gebreyohannes Y. K., Wozniak A., Zhai M.-E., Wellens J., Cornillie J., Vanleeuw U., Evans E., Gardino A. K., Lengauer C., Debiec-Rychter M., Sciot R., Schöffski P. (2019). Robust Activity
of Avapritinib, Potent and Highly Selective Inhibitor of Mutated KIT,
in Patient-Derived Xenograft Models of Gastrointestinal Stromal Tumors. Clin. Cancer Res..

[23] Haddox, C. L. ; Ballman, K. V. ; von Mehren, M. ; Heinrich, M. C. ; Bialick, S. ; Jacene, H. ; Shah, H. ; Crompton, B. D. ; Okuno, S. H. ; Fletcher, J. ; Wagner, A. J. SARC044: A Phase II Trial of Bezuclastinib in Combination with Sunitinib in Patients with GIST Who Progressed on Sunitinib Monotherapy. J. Clin.Oncol., 2025, 43. 10.1200/JCO.2025.43.16_suppl.TPS11579.

[24] Blum A., Dorsch D., Linde N., Brandstetter S., Buchstaller H. P., Busch M., Glaser N., Grädler U., Ruff A., Petersson C., Schieferstein H., Sherbetjian E., Esdar C. (2023). Identification of M4205—A
Highly Selective Inhibitor of KIT Mutations for Treatment of Unresectable
Metastatic or Recurrent Gastrointestinal Stromal Tumors. J. Med. Chem..

[25] Study Details NCT07218926 sponsored by GlaxoSmithKline; A Study of IDRX-42 (GSK6042981) Versus (vs) Sunitinib in Participants With Gastrointestinal Stromal Tumors After Imatinib Therapy. 2026. https://clinicaltrials.gov/study/NCT07218926?intr=IDRX-42&rank=1.

[26] Masucci M. T., Motti M. L., Minopoli M., Di Carluccio G., Carriero M. V. (2023). Emerging Targeted Therapeutic Strategies
to Overcome
Imatinib Resistance of Gastrointestinal Stromal Tumors. Int. J. Mol. Sci..

[27] Suzuki R., Lee J. H., Krishna S. G., Ramireddy S., Qiao W., Weston B., Ross W. A., Bhutani M. S. (2013). Repeat
Endoscopic Ultrasound-Guided Fine Needle Aspiration for Solid Pancreatic
Lesions at a Tertiary Referral Center Will Alter the Initial Inconclusive
Result. J. Gastrointest. Liver Dis..

[28] Heinrich M. C., Maki R. G., Corless C. L., Antonescu C. R., Harlow A., Griffith D., Town A., McKinley A., Ou W. Bin, Fletcher J. A., Fletcher C. D. M., Huang X., Cohen D. P., Baum C. M., Demetri G. D. (2008). Primary
and Secondary
Kinase Genotypes Correlate with the Biological and Clinical Activity
of Sunitinib in Imatinib-Resistant Gastrointestinal Stromal Tumor. J. Clin. Oncol..

[29] Nishida T., Blay J. Y., Hirota S., Kitagawa Y., Kang Y. K. (2016). The Standard
Diagnosis, Treatment, and Follow-up of Gastrointestinal Stromal Tumors
Based on Guidelines. Gastric Cancer..

[30] De
Sutter L., Wozniak A., Verreet J., Vanleeuw U., De Cock L., Linde N., Drechsler C., Esdar C., Sciot R., Schöffski P. (2023). Anti-Tumor
Efficacy of the Novel KIT Inhibitor IDRX-42 (Formerly M4205) in Patient-
and Cell Line-Derived Xenograft Models of Gastrointestinal Stromal
Tumor (GIST). Clin. Cancer Res..

[31] Emile J.-F. (2010). Diagnostic
Criteria, Specific Mutations, and Genetic Predisposition in Gastrointestinal
Stromal Tumors. Appl. Clin. Genet..

[32] KIT wild-type activity was assessed by measuring inhibition of KIT autophosphorylation in the M07E cell line (human factor-dependent myeloid leukemia) following SCF stimulation referred to as pKIT WT.

[33] Inhibition of PDGFR-β autophosphorylation was evaluated in the SW579 human thyroid squamous cell carcinoma cell line after stimulation with PDGF ligand referred to as pPDGFR-β.

[34] Eurofins. KINOMEscan - World’s largest kinase assay panel (489 Kinases). Https://Www.Eurofinsdiscovery.Com/Solution/Kinomescan-Technology.

[35] Eno M. S., Brubaker J. D., Campbell J. E., De Savi C., Guzi T. J., Williams B. D., Wilson D., Wilson K., Brooijmans N., Kim J., Özen A., Perola E., Hsieh J., Brown V., Fetalvero K., Garner A., Zhang Z., Stevison F., Woessner R., Singh J., Timsit Y., Kinkema C., Medendorp C., Lee C., Albayya F., Zalutskaya A., Schalm S., Dineen T. A. (2022). Discovery
of BLU-945, a Reversible,
Potent, and Wild-Type-Sparing Next-Generation EGFR Mutant Inhibitor
for Treatment-Resistant Non-Small-Cell Lung Cancer. J. Med. Chem..

[36] Moslin R., Zhang Y., Wrobleski S. T., Lin S., Mertzman M., Spergel S., Tokarski J. S., Strnad J., Gillooly K., McIntyre K. W., Zupa-Fernandez A., Cheng L., Sun H., Chaudhry C., Huang C., D’Arienzo C., Heimrich E., Yang X., Muckelbauer J. K., Chang C., Tredup J., Mulligan D., Xie D., Aranibar N., Chiney M., Burke J. R., Lombardo L., Carter P. H., Weinstein D. S. (2019). Identification of N-Methyl Nicotinamide
and N-Methyl Pyridazine-3-Carboxamide Pseudokinase Domain Ligands
as Highly Selective Allosteric Inhibitors of Tyrosine Kinase 2 (TYK2). J. Med. Chem..

[37] Chaix A., Lopez S., Voisset E., Gros L., Dubreuil P., De Sepulveda P. (2011). Mechanisms
of STAT Protein Activation by Oncogenic
KIT Mutants in Neoplastic Mast Cells. J. Biol.
Chem..

[38] Peter B., Bibi S., Eisenwort G., Wingelhofer B., Berger D., Stefanzl G., Blatt K., Herrmann H., Hadzijusufovic E., Hoermann G., Hoffmann T., Schwaab J., Jawhar M., Willmann M., Sperr W. R., Zuber J., Sotlar K., Horny H.-P., Moriggl R., Reiter A., Arock M., Valent P. (2018). Drug-Induced Inhibition of Phosphorylation
of STAT5 Overrides Drug Resistance in Neoplastic Mast Cells. Leukemia.

[39] Baumgartner C., Cerny-Reiterer S., Sonneck K., Mayerhofer M., Gleixner K. V., Fritz R., Kerenyi M., Boudot C., Gouilleux F., Kornfeld J.-W., Sillaber C., Moriggl R., Valent P. (2009). Expression
of Activated STAT5 in Neoplastic Mast Cells
in Systemic Mastocytosis. Am. J. Pathol..

[40] Banks E., Grondine M., Bhavsar D., Barry E., Kettle J. G., Reddy V. P., Brown C., Wang H., Mettetal J. T., Collins T., Adeyemi O., Overman R., Lawson D., Harmer A. R., Reimer C., Drew L., Packer M. J., Cosulich S., Jones R. DO., Shao W., Wilson D., Guichard S., Fawell S., Anjum R. (2020). Discovery and Pharmacological
Characterization of AZD3229, a Potent KIT/PDGFRα Inhibitor for
Treatment of Gastrointestinal Stromal Tumors. Sci. Transl. Med..

