# Supplementary material for: Design and Synthesis of BLU-654, a Potent and Selective Mutant KIT V654A Inhibitor for the Treatment of Imatinib-Resistant GIST
Source: J Med Chem. 2026 Mar 10;69(8):9083–103. doi: 10.1021/acs.jmedchem.5c03554 (PMC13126683; doi:10.1021/acs.jmedchem.5c03554)
Supplement: Supplementary file 1 [file jm5c03554_si_001.pdf]

Supporting Information

Design and synthesis of BLU-654, a potent and selective mutant KIT V654A inhibitor for the treatment of imatinib-resistant GIST

Ludivine Moine*,[1] Wei Hu,[1] Alison Davis, Emanuele Perola,[1] Jian Guo, [1] Kevin Barvian,[1] Yeon Sook Choi, Alexandra Grassian,[1] Joseph L Kim,[1] Omar K Ahmad,[1] Thomas A Dineen[1]

*Corresponding author

[1]*Blueprint Medicines Corporation, Cambridge, MA 02139, USA*

Co-corresponding Author:

Ludivine Moine

Blueprint Medicines Corporation

Cambridge, Massachusetts 02139, United States

Email: lmoine@blueprintmedicines.com;

Table of Contents

1. General Considerations: Synthesis **......7**

2. Synthesis & Characterization of Key Intermediates **......9**

Synthesis of 2-chloro-4-isopropoxy-5-(1-methyl-1*H*-pyrazol-4-yl)pyridine (SI-1) 9

Step 1: synthesis of 5-bromo-2-chloro-4-isopropoxypyridine (27) 9

Step 2: Synthesis of 2-chloro-4-isopropoxy-5-(1-methyl-1H-pyrazol-4-yl)pyridine. 9

Synthesis of 4-isopropoxy-5-(1-methyl-1*H*-pyrazol-4-yl)pyridin-2-amine (SI-2) 10

Step 1: Synthesis of N-(diphenylmethylene)-4-isopropoxy-5-(1-methyl-1H-pyrazol-4-yl)pyridin-2-amine. 10

Step 2: Synthesis of 4-isopropoxy-5-(1-methyl-1H-pyrazol-4-yl)pyridin-2-amine. 10

Synthesis of 2-(difluoromethyl)-*N*^{4}-(2,4-dimethoxybenzyl)pyrimidine-4,6-diamine (SI-3) ..11

Step 1: Synthesis of 2-(difluoromethyl)pyrimidine-4,6-diol. 11

Step 2: Synthesis of 4,6-dichloro-2-(difluoromethyl)pyrimidine. 11

Step 3: Synthesis of 6-chloro-2-(difluoromethyl)-N-(2,4-dimethoxybenzyl)pyrimidin-4-amine. 12

Step 4: Synthesis of 4: 6-chloro-2-(difluoromethyl)pyrimidin-4-amine. 12

Step 5: Synthesis of 5: 2-(difluoromethyl)-N^4-(2,4-dimethoxybenzyl)pyrimidine-4,6-diamine. 12

Synthesis of 2-(difluoromethyl)pyrimidine-4,6-diamine hydrochloride (SI-4) 13

Step 1: Synthesis of 6: N,N'-(2-(difluoromethyl)pyrimidine-4,6-diyl)bis(1,1-diphenyl methanimine). 13

Step 2: Synthesis of 7: 2-(difluoromethyl)pyrimidine-4,6-diamine hydrochloride. 13

3. Synthesis & Characterization of Compounds 1-18 **......15**

Compound 1 : Synthesis of *N*^{4}-(4-isopropoxy-5-(1-methyl-1*H*-pyrazol-4-yl)pyridin-2-yl)-N^2-methylpyrimidine-2,4-diamine 15

Step 1: Synthesis of 2-chloro-N-(4-isopropoxy-5-(1-methyl-1H-pyrazol-4-yl)pyridin-2-yl)pyrimidin-4-amine. 15

Step 2: Synthesis of N^4-(4-isopropoxy-5-(1-methyl-1H-pyrazol-4-yl)pyridin-2-yl)-N2-methylpyrimidine-2,4-diamine. 15

Compound 2. Synthesis of 6-chloro-*N*^{4}-(4-isopropoxy-5-(1-methyl-1*H*-pyrazol-4-yl)pyridin-2-yl)-N2-methylpyrimidine-2,4-diamine. 16

Step 1: Synthesis of 6-chloro-N^2-methyl-pyrimidine-2,4-diamine. 16

Step 2: Synthesis of 6-chloro-N^4-(4-isopropoxy-5-(1-methyl-1H-pyrazol-4-yl)pyridin-2-yl)-N^2-methylpyrimidine-2,4-diamine. 16

Compound 3. Synthesis of 6-fluoro-*N*^{4}-(4-isopropoxy-5-(1-methyl-1*H*-pyrazol-4-yl)pyridin-2-yl)-N2-methylpyrimidine-2,4-diamine 17

Step 1: Synthesis of 4,6-difluoro-N-methylpyrimidin-2-amine. 17

Step 2: Synthesis of 6-fluoro-N^2-methylpyrimidine-2,4-diamine. 17

Step 3. Synthesis of 6-fluoro-N^4-(4-isopropoxy-5-(1-methyl-1H-pyrazol-4-yl)pyridin-2-yl)-N^2-methylpyrimidine-2,4-diamine. 18

Compound 4. Synthesis of *N*^{4}-(4-isopropoxy-5-(1-methyl-1*H*-pyrazol-4-yl)pyridin-2-yl)-N^2,6-dimethylpyrimidine-2,4-diamine 18

Step 1: Synthesis of N^2,6-dimethylpyrimidine-2,4-diamine. 18

Step 2: Synthesis of N^4-(4-isopropoxy-5-(1-methyl-1H-pyrazol-4-yl)pyridin-2-yl)-N^2,6-dimethylpyrimidine-2,4-diamine. 19

Compound 5. Synthesis of *N*^{4}-(4-isopropoxy-5-(1-methyl-1*H*-pyrazol-4-yl)pyridin-2-yl)-6-methoxy-*N*^{2}-methylpyrimidine-2,4-diamine..19

Step 1: Synthesis of 4-chloro-6-methoxy-N-methylpyrimidin-2-amine. 19

Step 2: Synthesis of N^4-(4-isopropoxy-5-(1-methyl-1H-pyrazol-4-yl)pyridin-2-yl)-6-methoxy-N2-methylpyrimidine-2,4-diamine. 20

Compound 6. Synthesis of *N*^{4}-(4-isopropoxy-5-(1-methyl-1*H*-pyrazol-4-yl)pyridin-2-yl)-N2-methylpyrimidine-2,4,6-triamine 20

Step 1: Synthesis of N4,N6-dibenzyl-N2-methylpyrimidine-2,4,6-triamine. 20

Step 2: Synthesis of N^2-methylpyrimidine-2,4,6-triamine 21

Step 3: Synthesis of N^4-(4-isopropoxy-5-(1-methyl-1H-pyrazol-4-yl)pyridin-2-yl)-N^2-methylpyrimidine-2,4,6-triamine. 21

Compound 7. Synthesis of *N*^{4}-(4-isopropoxy-5-(1-methyl-1*H*-pyrazol-4-yl)pyridin-2-yl)-2-methylpyrimidine-4,6-diamine..21

Step 1: Synthesis of N^4,N^6-dibenzyl-2-methylpyrimidine-4,6-diamine.................................22

Step 2: Synthesis of 2-methylpyrimidine-4,6-diamine. 22

Step 3: Synthesis of N^4-(4-isopropoxy-5-(1-methyl-1H-pyrazol-4-yl)pyridin-2-yl)-2-methylpyrimidine-4,6-diamine. 22

Compound 8. Synthesis of 2-cyclopropyl-*N*^{4}-(4-isopropoxy-5-(1-methyl-1*H*-pyrazol-4-yl)pyridin-2-yl)pyrimidine-4,6-diamine 23

Step 1: Synthesis of N,N'-(2-cyclopropylpyrimidine-4,6-diyl)bis(1,1-diphenylmethanimine). 23

Step 2: Synthesis of 2-cyclopropylpyrimidine-4,6-diamine. 23

Step 3. Synthesis of 2-cyclopropyl-N^4-(4-isopropoxy-5-(1-methyl-1H-pyrazol-4-yl)pyridin-2-yl)pyrimidine-4,6-diamine. 24

Compound 9. Synthesis of *N*^{4}-(5-isopropyl-8-methoxy-2,7-naphthyridin-3-yl)-N^2-methylpyrimidine-2,4,6-triamine. 24

Step 1: Synthesis of 6-chloro-4-(prop-1-en-2-yl)-2,7-naphthyridin-1(2H)-one. 25

Step 2: Synthesis of 6-chloro-4-isopropyl-2,7-naphthyridin-1(2H)-one. 25

Step 3: Synthesis of 1,6-dichloro-4-isopropyl-2,7-naphthyridine..25

Step 4: Synthesis of 6-chloro-4-isopropyl-1-methoxy-2,7-naphthyridine. 26

Step 5: Synthesis of N^4-(5-isopropyl-8-methoxy-2,7-naphthyridin-3-yl)-N^2-methylpyrimidine-2,4,6-triamine. 26

Compound 10. Synthesis of 6-((6-amino-2-(methylamino)pyrimidin-4-yl)amino)-4-(isopropylamino)-N-methylnicotinamide 26

Step 1: Synthesis of methyl 6-chloro-4-(isopropylamino)nicotinate. 27

Step 2: Synthesis of 6-chloro-4-(isopropylamino)nicotinic acid. 27

Step 3: Synthesis of 6-chloro-4-(isopropylamino)-N-methylnicotinamide. 27

Step 4: Synthesis of 6-((6-amino-2-(methylamino)pyrimidin-4-yl)amino)-4-(isopropylamino)-N-methylnicotinamide. 28

Compound 11. Synthesis of 6-((6-amino-2-cyclopropylpyrimidin-4-yl)amino)-4-(isopropylamino)-N-methylnicotinamide. 28

Step 1: Synthesis of N-(tert-butyl)-6-chloro-2-cyclopropylpyrimidin-4-amine. 29

Step 2: Synthesis of N-(tert-butyl)-2-cyclopropyl-6-((diphenylmethylene)amino) pyrimidin-4-amine. 29

Step 3: Synthesis of N^4-(tert-butyl)-2-cyclopropylpyrimidine-4,6-diamine. 29

Step 4: Synthesis of 6-((6-(tert-butylamino)-2-cyclopropylpyrimidin-4-yl)amino)-4-(isopropylamino)-N-methylnicotinamide. 30

Step 5: Synthesis of 6-((6-amino-2-cyclopropylpyrimidin-4-yl)amino)-4-(isopropylamino)-N-methylnicotinamide 30

Compound 12. Synthesis of 6-((6-amino-2-(difluoromethyl)pyrimidin-4-yl)amino)-4-(isopropylamino)-N-methylnicotinamide 31

Compound 13. Synthesis of (S)-6-((6-amino-2-(difluoromethyl)pyrimidin-4-yl)amino)-4-((1-fluoropropan-2-yl)amino)-N-methylnicotinamide 31

Step 1: Synthesis of methyl (S)-6-chloro-4-((1-fluoropropan-2-yl)amino)nicotinate. 32

Step 2: Synthesis of (S)-6-chloro-4-((1-fluoropropan-2-yl)amino)nicotinic acid. 32

Step 3: Synthesis of (S)-6-chloro-4-((1-fluoropropan-2-yl)amino)-N-methyl nicotinamide. ..32

Step 4: Synthesis of (S)-6-((6-amino-2-(difluoromethyl)pyrimidin-4-yl)amino)-4-((1-fluoropropan-2-yl)amino)-N-methylnicotinamide. 33

Compound 14. Synthesis of 2-(difluoromethyl)-*N*^{4}-(4-isopropoxy-5-(1-methyl-1*H*-pyrazol-4-yl)pyridin-2-yl)pyrimidine-4,6-diamine. 33

Compound 15. Synthesis of 2-(difluoromethyl)-*N*^{4}-(4-methoxy-5-(1-methyl-1*H*-pyrazol-4-yl)pyridin-2-yl)pyrimidine-4,6-diamine. 34

Step 1: Synthesis of 2-chloro-4-fluoro-5-(4,4,5,5-tetramethyl-1,3,2-dioxaborolan-2-yl)pyridine. 34

Step 2: Synthesis of 2-chloro-4-fluoro-5-(1-methyl-1H-pyrazol-4-yl)pyridine. 34

Step 3. Synthesis of 2-chloro-4-methoxy-5-(1-methyl-1H-pyrazol-4-yl)pyridine. 35

Step 4: Synthesis of 2-(difluoromethyl)-N4-(4-methoxy-5-(1-methyl-1H-pyrazol-4-yl)pyridin-2-yl)pyrimidine-4,6-diamine. 35

Compound 16. Synthesis of 2-(difluoromethyl)-*N*4-(4-methoxy-5-(1-(2-(methylamino)ethyl)-1*H*-pyrazol-4-yl)pyridin-2-yl)pyrimidine-4,6-diamine. 36

Step 1: Synthesis of 5-bromo-2-chloro-4-methoxypyridine. 36

Step 2: Synthesis of tert-butyl methyl(2-(4-(4,4,5,5-tetramethyl-1,3,2-dioxaborolan-2-yl)-1H-pyrazol-1-yl)ethyl)carbamate. 36

Step 3: Synthesis of tert-butyl (2-(4-(6-chloro-4-methoxypyridin-3-yl)-1H-pyrazol-1-yl)ethyl)(methyl)carbamate. 37

Step 4: Synthesis of tert-butyl (2-(4-(6-((6-amino-2-(difluoromethyl)pyrimidin-4-yl)amino)-4-methoxypyridin-3-yl)-1H-pyrazol-1-yl)ethyl)(methyl)carbamate. 37

Step 5: Synthesis of 2-(difluoromethyl)-N4-(4-methoxy-5-(1-(2-(methylamino)ethyl)-1H-pyrazol-4-yl)pyridin-2-yl)pyrimidine-4,6-diamine. 37

Compound 17. Synthesis of 1-(4-(6-((6-amino-2-(difluoromethyl)pyrimidin-4-yl)amino)-4-methoxypyridin-3-yl)-1*H*-pyrazol-1-yl)-2-methylpropan-2-ol. 38

Step 1: Synthesis of 1-(4-(6-chloro-4-methoxypyridin-3-yl)-1H-pyrazol-1-yl)-2-methylpropan-2-ol. 38

Step 2: Synthesis of 1-(4-(6-((2-(difluoromethyl)-6-((2,4-dimethoxybenzyl)amino) pyrimidin-4-yl)amino)-4-methoxypyridin-3-yl)-1H-pyrazol-1-yl)-2-methylpropan-2-ol. 39

Step 3: Synthesis of 1-(4-(6-((6-amino-2-(difluoromethyl)pyrimidin-4-yl)amino)-4-methoxypyridin-3-yl)-1H-pyrazol-1-yl)-2-methylpropan-2-ol. 39

Compound 18. Synthesis of (S)-1-(4-(6-((6-amino-2-(1-fluoroethyl)pyrimidin-4-yl)amino)-4-isopropoxypyridin-3-yl)-1*H*-pyrazol-1-yl)-2-methylpropan-2-ol 40

Step 1. Synthesis of 2-(1-fluoroethyl)pyrimidine-4,6-diol (21). 40

Step 2: Synthesis of 4,6-dichloro-2-(1-fluoroethyl)pyrimidine (22). 40

Step 3: Synthesis of 6-chloro-N-(2,4-dimethoxybenzyl)-2-(1-fluoroethyl)pyrimidin-4-amine (23). 41

Step 4: Synthesis of 6-chloro-2-(1-fluoroethyl)pyrimidin-4-amine (24). 41

Step 5: Synthesis of (S)-N4-(2,4-dimethoxybenzyl)-2-(1-fluoroethyl) pyrimidine-4,6-diamine (25) and (R)-N4-(2,4-dimethoxybenzyl)-2-(1-fluoroethyl)pyrimidine-4,6-diamine. 42

Step 6: Synthesis of 1-(4-(6-chloro-4-isopropoxypyridin-3-yl)-1H-pyrazol-1-yl)-2-methylpropan-2-ol (28). 42

Step 7: Synthesis of (S)-1-(4-(6-((6-((2,4-dimethoxybenzyl)amino)-2-(1-fluoroethyl)pyrimidin-4-yl)amino)-4-isopropoxypyridin-3-yl)-1H-pyrazol-1-yl)-2-methylpropan-2-ol (29). 43

Step 8: Synthesis of (S)-1-(4-(6-((6-amino-2-(1-fluoroethyl)pyrimidin-4-yl)amino)-4-isopropoxypyridin-3-yl)-1H-pyrazol-1-yl)-2-methylpropan-2-ol (18). 43

4. Purity Analysis for Compound 18* *44

5. Kinome data for compound 18* *47

6. Crystallography* *48

X-ray data collection and refinement statistics for compounds 1 and 11 48

X-ray of compound 25 to confirm stereochemistry 50

7. Biology* *53

Inhibition of KIT Autophosphorylation in HMC1.1 11/13 Cells by MSD assay 53

Inhibition of KIT Autophosphorylation in M-07e Cells by MSD assay 54

Inhibition of PDGFRβ Autophosphorylation in SW579 Cells by HTRF 56

8. Quality control of tested compounds and molecular formula strings* *57

9. In Vivo Pharmacokinetics and Pharmacodynamics Studies* *59

10. Spectroscopic data of Compounds 1-18* *61

11. NMR spectra of Compounds 1-18* *79

1. General Considerations: Synthesis

All solvents employed were commercially available anhydrous grade, and reagents were used as received unless otherwise noted. Compound purity of all compounds was assessed by high-performance liquid chromatography (HPLC) to confirm >95% purity. The liquid chromatography-mass spectrometry (LC-MS) data were obtained with an Agilent model-1260 LC system using an Agilent model 6120 mass spectrometer utilizing ES-API ionization fitted with an Agilent Poroshel 120 (EC-C18, 2.7 μm particle size, 3.0 x 50 mm dimensions) reversed-phase column. The mobile phase consisted of a mixture of solvent 0.1% formic acid in water and 0.1% formic acid in acetonitrile. A constant gradient from 95% aqueous/5% organic to 5% aqueous/95% organic mobile phase over the course of 4 min was utilized. The flow rate was constant at 1 mL/min. Alternatively, the LC-MS data were obtained with a Shimadzu LC-MS system using an Shimadzu LC-MS mass spectrometer utilizing ESI ionization fitted with an Agilent (Poroshel HPH-C18 2.7 μm particle size, 3.0 x 50 mm dimensions) reversed-phase column. The mobile phase consisted of a mixture of solvent 5 mM NH_4HCO_3 (or 0.05% TFA) in water and acetonitrile. A constant gradient from 90% aqueous/10% organic to 5% aqueous/95% organic mobile phase over the course of 2 min was utilized. The flow rate was constant at 1.5 mL/min. Preparative HPLC was performed on a Shimadzu Discovery VPR Preparative system fitted with a Luna 5 μm C18(2) 100 Å, AXIA packed, 250 x 21.2 mm reversed-phase column. Alternatively, the preparative HPLC was performed on a Waters Preparative system fitted with Column: XBridge Shield RP18 OBD Column, 30*150 mm, 5 μm; the mobile phase consisted of a mixture of solvent water (10 mmol/L NH_4CO_3 + 0.05% NH_3•H_20) and acetonitrile. A constant gradient from 95% aqueous/5% organic to 5% aqueous/95% organic mobile phase over the course of 11 min was utilized. The flow rate was constant at 60 mL/min. Reactions carried out in a microwave were performed in a Biotage Initiator microwave unit. Silica gel chromatography was performed on a Teledyne Isco CombiFlash Rf unit, a BiotageR Isolera Four unit, or a BiotageR Isolera Prime unit. ^{1}H nuclear magnetic resonance (NMR) spectra were obtained with a Varian 400 MHz Unity Inova 400 MHz NMR

instrument, Avance 400 MHz Unity Inova 400 MHz NMR instrument, or an Avance 300 MHz Unity Inova 300 MHz NMR instrument. Unless otherwise indicated, all protons were reported in DMSO-d6 solvent as parts-per million (ppm) with respect to residual DMSO (2.50 ppm). Chiral-HPLC was performed on an Agilent 1260 Preparative system. Chiral-SFC purification was performed with a Waters preparative system.

Supplementary Table 1. Preparative HPLC codes and associated method conditions.

Prep-HPLC Code	Conditions
HPLC-A	Welch Xtimate C18 250 x 70 mm x 10 μm; water (0.05% NH_3H_2O)-MeCN
HPLC-B	Welch Xtimate 150A 250 x 21.2 mm x 10 μm; water (0.1% NH_4HCO_3), 15-95%
HPLC-C	Welch Xtimate C18 150 x 25 mm x 5 μm; water (0.05% NH_3H_2O)-MeCN

2. Synthesis & Characterization of Key Intermediates

Synthesis of 2-chloro-4-isopropoxy-5-(1-methyl-1*H*-pyrazol-4-yl)pyridine (SI-1)

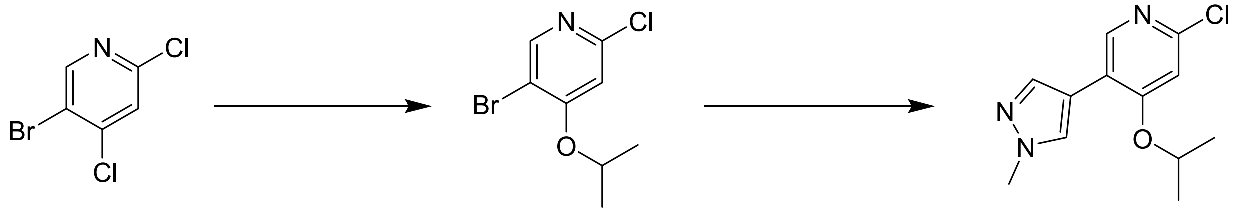

26 **27** **SI-1**

Step 1: synthesis of 5-bromo-2-chloro-4-isopropoxypyridine (27).

To a solution of i-PrOH (6.60 g, 110 mmol) in THF (150 mL) at 0 ^{o}C was added NaH (6.60 g, 165 mmol), the reaction mixture was stirred at 0 ^{o}C for 30 min, then 5-bromo-2,4-dichloropyridine **26** (25 g, 110 mmol) in THF (100 mL) was added. The mixture was stirred at room temperature for 12 h. After that, the solution was quenched with water, then extracted with EtOAc. The organic layers were concentrated and purified by flash chromatography on silica gel eluting with PE/EtOAc (10:1~2:1) to get the title product as a white solid (20.0 g, 73% yield). LC-MS m/z = 250 [M+1].

Step 2: Synthesis of 2-chloro-4-isopropoxy-5-(1-methyl-1H-pyrazol-4-yl)pyridine.

A mixture of 5-bromo-2-chloro-4-isopropoxypyridine (10.0 g, 40 mmol), 1-methyl-4-(4,4,5,5-tetramethyl-1,3,2-dioxaborolan-2-yl)-1*H*-pyrazole (8.32 g, 40 mmol), Pd(dppf)Cl_2 (3.26 g, 4.45 mmol) and K_2CO_3 (16.6 g, 120 mmol) in dioxane/water (100 mL/20 mL) was stirred at 90 ^{o}C under N_2 for 4 h. The reaction mixture was partitioned between EtOAc and water. The aqueous layer was extracted with EtOAc, the combined organic layers were washed with brine and dried over anhydrous Na_2SO_4. The organic layers were concentrated and purified by flash chromatography on silica gel eluting with PE/EtOAc (3:1) to afford the title compound (5.1 g, 51% yield) as a white solid. LC-MS m/z = 252 [M+1].

Synthesis of 4-isopropoxy-5-(1-methyl-1*H*-pyrazol-4-yl)pyridin-2-amine (SI-2)

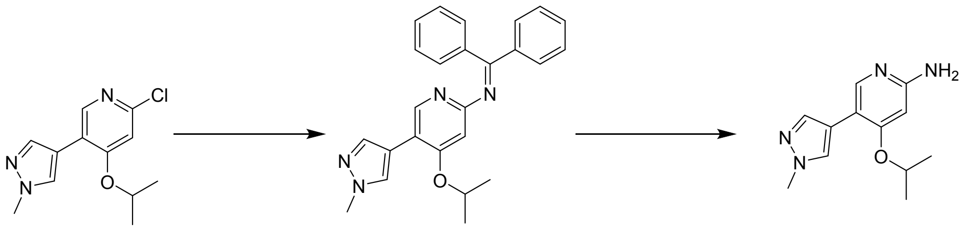

SI-1 SI-2

Step 1: Synthesis of N-(diphenylmethylene)-4-isopropoxy-5-(1-methyl-1H-pyrazol-4-yl)pyridin-2-amine.

A mixture of 2-chloro-4-isopropoxy-5-(1-methyl-1*H*-pyrazol-4-yl)pyridine (3.0 g, 12 mmol), benzophenone imine (4.3 g, 24 mmol), Cs_2CO_3 (11.6 g, 36 mmol), $Pd_2(dba)_3$ (1.0 g, 1.2 mmol), XantPhos (0.58 g, 1.2 mmol) in dioxane (30 mL) was stirred at 100 °C under N_2 for 4 h. LC-MS showed the reaction was completed. The reaction mixture was partitioned between EtOAc and water. The aqueous layer was extracted with EtOAc, then the combined organic layers were washed with brine and dried over anhydrous Na_2SO_4. The organic layers were concentrated and purified by flash chromatography on silica gel eluting with PE/EtOAc (2:1) to afford the title compound (4.0 g, 84% yield) as a white solid. LC-MS m/z = 397 [M+1].

Step 2: Synthesis of 4-isopropoxy-5-(1-methyl-1*H*-pyrazol-4-yl)pyridin-2-amine.

To a solution of *N*-(diphenylmethylene)-4-isopropoxy-5-(1-methyl-1*H*-pyrazol-4-yl)pyridin-2-amine (4.0 g, 10 mmol) in DCM (20 mL) was added HCl/dioxane (20 mL), and the reaction mixture was stirred at room temperature for 4 h. The reaction mixture was filtered and was partitioned between DCM and saturated aqueous $NaHCO_3$. The organic layer was washed with brine, dried over Na_2SO_4, filtered, and concentrated under reduced pressure to give the title product which was used for the next step without further purification (1.88 g, 80% yield). LC-MS m/z = 233 [M+1] .

Synthesis of 2-(difluoromethyl)-*N*[4]-(2,4-dimethoxybenzyl)pyrimidine-4,6-diamine (SI-3)

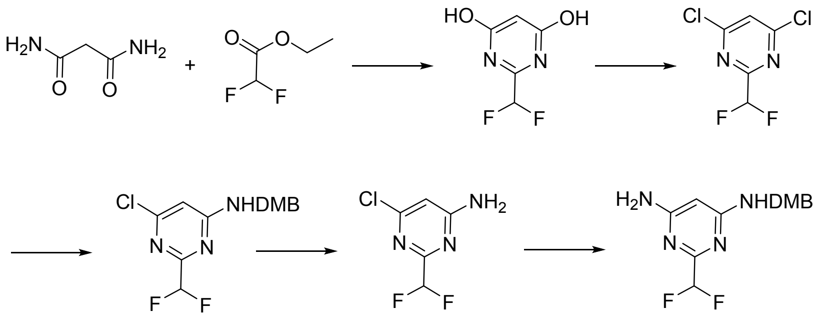

Step 1: Synthesis of ~~1:~~ 2-(difluoromethyl)pyrimidine-4,6-diol.

To a solution of malonamide (600 g, 5.88 mol) in EtOH (12 L) was added EtONa (799 g, 11.75 mol) and the solution stirred at room temperature for 1 h. Ethyl 2,2-difluoroacetate (875.11 g, 7.05 mol) was added dropwise and the reaction was heated at 90 °C for 15 h. The cooled reaction mixture was filtered, the solid washed with EtOH/EtOAc (1:1), and dried *in vacuo* to give the title compound (800 g, 71.4%) as a yellow solid.

Step 2: Synthesis of ~~2:~~ 4,6-dichloro-2-(difluoromethyl)pyrimidine.

To a solution of 2-(difluoromethyl)pyrimidine-4,6-diol (150 g, 740.31 mmol) in toluene (3 L) was added $POCl_3$ (300.15 mL, 3.23 mol) dropwise followed by DIPEA (526.95 mL, 3.03 mol) and the reaction heated at 120 °C for 16 h. The cooled reaction mixture was concentrated under reduced pressure, the residue diluted with EtOAc (900 mL), and saturated aq. $NaHCO_3$ was added to adjust the pH to 7-8. The layers were separated, the organic layer washed with brine (300 mL x 3), dried over Na_2SO_4, filtered, and concentrated under reduced pressure. The residue was purified by column chromatography on silica gel (I0~5% EtOAc/PE) to give the title compound (85 g, 57.1%) as a yellow oil. ^{1}H NMR (400 MHz, DMSO-d_6) δ ppm 7.18-6.83 (m, 1 H) 8.31 (s, 1 H).

Step 3: Synthesis of 6-chloro-2-(difluoromethyl)-N-(2,4-dimethoxybenzyl)pyrimidin-4-amine.

To a solution of 4,6-dichloro-2-(difluoromethyl)pyrimidine (50.0 g, 251 mmol) and 2,4-dimethoxybenzylamine (46.2 g, 276 mmol) in NMP (250 mL) was added DIPEA (64.9 g, 502 mmol) and the reaction stirred at 140 °C for 2 h. The cooled reaction mixture was poured into water (500 mL) and extracted with EtOAc (400 mL x 3). The combined organic layers were washed with brine (500 mL), dried over Na_2SO_4, filtered, and the filtrate was concentrated under reduced pressure. The crude product was purified by silica gel chromatography (PE/EtOAc =50/1 to 10/1) to give the title compound (60.0 g, 70.3% yield) as a yellow solid. LC-MS m/z = 330 [M+1]; ^{1}H NMR (400 MHz, DMSO-d_6) δ: 8.61-8.23 (m, 1H), 7.24-7.06 (m, 1H), 6.80-6.44 (m, 4H), 4.50-4.23 (m, 2H), 3.79 (s, 3H), 3.74 (s, 3H).

Step 4: Synthesis of 4: 6-chloro-2-(difluoromethyl)pyrimidin-4-amine.

A solution of 6-chloro-2-(difluoromethyl)-*N*-(2,4-dimethoxybenzyl)pyrimidin-4-amine (60.0 g, 176 mmol) in HCl/EtOAc (4 M, 257 mL) was stirred at room temperature for 12 h. The pH of the mixture was adjusted to about 8 with saturated aqueous $NaHCO_3$ solution. The mixture was extracted with EtOAc (500 mL x 3) and the combined organic layer was evaporated under reduced pressure to give the title compound (30 g) which was used without further purification. LC-MS m/z = 180 [M+1].

Step 5: Synthesis of 5: 2-(difluoromethyl)-N^4-(2,4-dimethoxybenzyl)pyrimidine-4,6-diamine.

The title compound was obtained as an off-white solid (25.31 g, 47.8% yield) from 6-chloro-2-(difluoromethyl)pyrimidin-4-amine and 2,4-dimethoxybenzylamine following a similar procedure to that described in step 3. LC-MS m/z = 311.1 [M+1]; ^{1}H NMR (400

MHz, DMSO-d_6) δ 7.16 (t, 1H), 7.07 (d, 1H), 6.58-6.19 (m, 5H), 5.35 (s, 1H), 4.24 (s, 2H), 3.80 (s, 3H), 3.73 (s, 3H).

Synthesis of 2-(difluoromethyl)pyrimidine-4,6-diamine hydrochloride (SI-4)

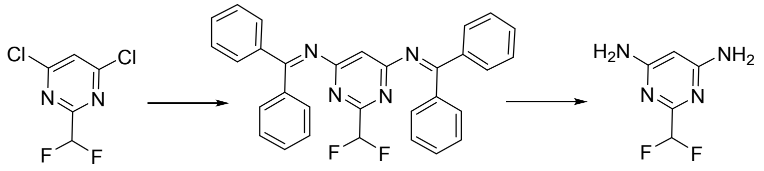

Step 1: Synthesis of 6: N,N'-(2-(difluoromethyl)pyrimidine-4,6-diyl)bis(1,1-diphenyl methanimine).

To a solution of 4,6-dichloro-2-(difluoromethyl)pyrimidine (145 g, 721.41 mmol) in dioxane (2 L) was added benzophenone imine (326.86 g, 1.80 mol), Cs_2CO_3 (705.15 g, 2.16 mol), XantPhos (41.74 g, 72.14 mmol), and $Pd_2(dba)_3$ (33.03 g, 36.07 mmol) under N_2 and the reaction was heated at 100 °C for 8 h. The cooled reaction mixture was filtered and concentrated under reduced pressure. The residue was purified by silica gel chromatography (0~10 % EtOAc/PE) to give the title compound (250 g, 67%) as a yellow oil. ^1H NMR (400 MHz, DMSO-d_6) δ ppm 6.39-6.57 (m, 2H) 7.27-7.60 (m, 20H).

Step 2: Synthesis of 7: 2-(difluoromethyl)pyrimidine-4,6-diamine hydrochloride.

To a solution of *N*,*N*'-(2-(difluoromethyl)pyrimidine-4,6-diyl)bis(1,1-diphenyl methanimine) (250 g, 511.74 mmol) in dioxane (1.5 L) was added HCl/dioxane (4 M, 800 mL) and the reaction stirred at room temperature for 16 h. The reaction was concentrated under reduced pressure and the residue was triturated with THF (1 L) for 30 min. The solid was filtered off to give the title compound (113 g, 68.6% yield) as a brown solid. LC-MS m/z = 160 [M+1].

3. Synthesis & Characterization of Compounds 1-18

Compound 1 : Synthesis of *N*4-(4-isopropoxy-5-(1-methyl-1*H*-pyrazol-4-yl)pyridin-2-yl)-N2-methylpyrimidine-2,4-diamine

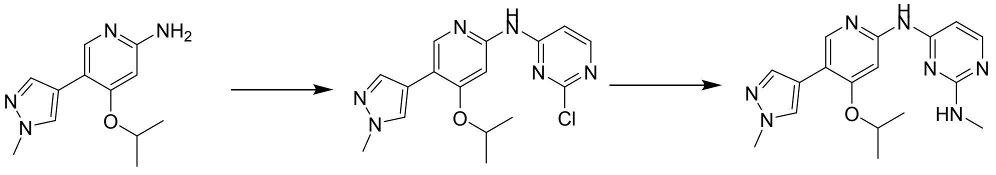

Step 1: Synthesis of 2-chloro-N-(4-isopropoxy-5-(1-methyl-1H-pyrazol-4-yl)pyridin-2-yl)pyrimidin-4-amine.

To a solution of **SI-1** (0.50 g, 2.15 mmol, 1.00 *eq*) in DMF (10 mL) was added Cs_2CO_3 (1.40 g, 4.31 mmol, 2.00 *eq*), XantPhos (125 mg, 215 μmol, 0.10 *eq*), and $Pd_2(dba)_3$ (197 mg, 215 μmol, 0.10 *eq*) in portion under N_2, then 2,4-dichloropyrimidine (353 mg, 2.37 mmol, 1.10 *eq*) was added at this temperature. The reaction was stirred at room temperature for 12 h. The reaction mixture was filtered and the filter was concentrated under reduced pressure. The crude product was purified by prep-TLC (PE:EtOAc; 0:1, Rf = 0.5) to give the title compound (200.0 mg, 580 μmol, 27.0% yield) as a yellow solid. LC-MS m/z = 345.1 [M+1]; ^{1}H NMR (400 MHz, DMSO-d_6) δ ppm 10.54 (br.s, 1H), 8.45 (s, 1H), 8.31 (d, J = 5.6 Hz, 1H), 8.07 (s, 1H), 7.91 (s, 1H), 7.70 (br.s, 1H), 7.43 (br.s, 1H), 4.71-4.74 (m, 1H), 3.87 (s, 3H),1.44 (d, *J* =6.0 Hz, 6H).

Step 2: Synthesis of N4-(4-isopropoxy-5-(1-methyl-1H-pyrazol-4-yl)pyridin-2-yl)-N2-methylpyrimidine-2,4-diamine.

To a solution of 2-chloro-*N*-(4-isopropoxy-5-(1-methyl-1*H*-pyrazol-4-yl)pyridin-2-yl)pyrimidin-4-amine (50.0 mg, 145 μmol, 1.00 *eq*) and methanamine (19.6 mg, 290 μmol, 2.00 *eq*, HCl) in EtOH (2 mL) was added Et_3N (44.0 mg, 435 μmol, 60.6 uL, 3.00 *eq*). The reaction was stirred at 80 °C for 30 h in a sealed tube. The mixture was

adjusted to pH to 6-7 with HCl solution (4 N) and the residue was concentrated in vacuum. The residue was purified by prep-HPLC (column: Boston Prime C18 150*30 mm 5 μm; mobile phase: [water (0.1% TFA) - ACN]; B%: 15%-45%, 9 min) to afford the title compound (16.8 mg, 36.7 μmol, 25.3% yield, TFA salt) as a light yellow solid. LC-MS m/z = 340.2 [M+1]; ^{1}H NMR (400 MHz, DMSO) δ 11.11 (s, 1H), 8.54 (s, 1H), 8.42 (s, 1H), 8.12 (s, 1H), 7.97 (d, *J* = 11.3 Hz, 2H), 6.59 (s, 1H), 4.84 (s, 1H), 3.89 (s, 3H), 2.99 (s, 3H), 1.42 (d, *J* = 6.0 Hz, 6H).

Compound 2. Synthesis of 6-chloro-*N*^{4}-(4-isopropoxy-5-(1-methyl-1*H*-pyrazol-4-yl)pyridin-2-yl)-N2-methylpyrimidine-2,4-diamine.

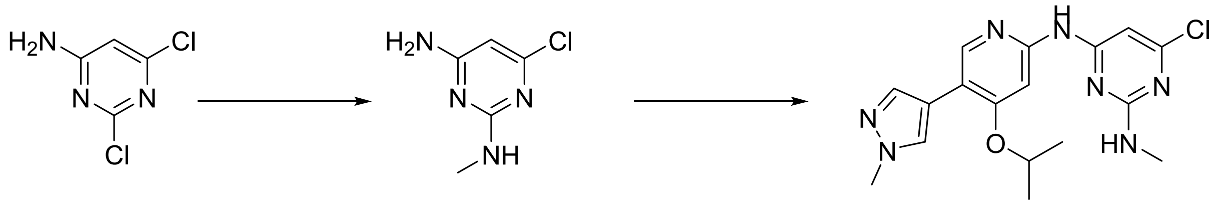

Step 1: Synthesis of 6-chloro-N^2-methyl-pyrimidine-2,4-diamine.

To a solution of 2,6-dichloropyrimidin-4-amine (500 mg, 3.04 mmol) and methanamine hydrochloride (307 mg, 4.56 mmol) in IPA (7 mL) was added DIPEA (1.96 g, 15.2 mmol) at room temperature. The mixture was stirred at 90 °C for 3 h. The reaction mixture was purified by silica gel chromatography (PE/EA=1:1) to obtain the title product (300 mg, 62% yield) as a white solid. LC-MS m/z = 159 [M+1].

Step 2: Synthesis of 6-chloro-N^4-(4-isopropoxy-5-(1-methyl-1H-pyrazol-4-yl)pyridin-2-yl)-N^2-methylpyrimidine-2,4-diamine.

To a mixture of 6-chloro-*N*^{2}-methylpyrimidine-2,4-diamine (188 mg, 1.19 mmol) and 2-chloro-5-(1-methyl-1*H*-pyrazol-4-yl)-4-(propan-2-yloxy)pyridine **SI-1** (150 mg, 597 μmol) in dioxane (5 mL) was added $Pd_2(dba)_3$ (86.5 mg, 94.5 μmol), XantPhos (109 mg, 189 μmol), and Cs_2CO_3 (585 mg, 1.79 mmol) at room temperature. The reaction mixture

was stirred at 100 °C for 14 h under N_2. The reaction mixture was concentrated and the residue was purified by silica gel chromatography (EA/MeOH=9:1) then by Prep-HPLC (A:water(10 mM NH_4HCO_3 & 0.025% $NH_3 \cdot H_2O$)), B: CAN; 45% B for 1 min, then 60% B in 7 min, stop at 15 min) to obtain the title product (18.7 mg, 8% yield) as a white solid. LC-MS m/z = 374 [M+1]; ^{1}H NMR (500 MHz, DMSO) δ 9.89 (s, 1H), 8.41 (s, 1H), 8.04 (s, 1H), 7.89 (s, 1H), 7.85 (s, 1H), 7.32 (s, 1H), 6.69 (s, 1H), 4.78 (s, 1H), 3.87 (s, 3H), 2.85 (s, 3H), 1.40 (d, *J* = 6.1 Hz, 6H).

Compound 3. Synthesis of 6-fluoro-*N*⁴-(4-isopropoxy-5-(1-methyl-1*H*-pyrazol-4-yl)pyridin-2-yl)-N2-methylpyrimidine-2,4-diamine

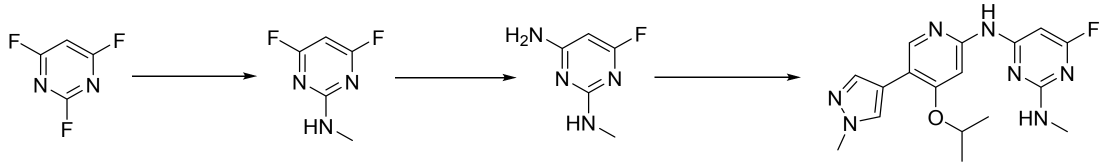

Step 1: Synthesis of 4,6-difluoro-N-methylpyrimidin-2-amine.

To a solution of 2,4,6-trifluoropyrimidine (1.0 g, 7.46 mmol) and methanamine hydrochloride (754 mg, 11.2 mmol) in IPA (10 mL) was added DIPEA (2.89 g, 22.4 mmol) at -20 °C, then was stirred and allowed to warm to room temperature gradually. The reaction mixture was purified by silica gel chromatography (PE/EA=1:1) to obtain the title product (400 mg, 37% yield) as a yellow solid. LC-MS m/z = 146 [M+1].

Step 2: Synthesis of 6-fluoro-N²-methylpyrimidine-2,4-diamine.

A mixture of 4,6-difluoro-*N*-methylpyrimidin-2-amine (400 mg, 2.75 mmol) in NH_3/dioxane (0.5 M, 10 mL) was stirred at 60 °C for 15 h. The reaction mixture was concentrated under reduced pressure to give the title product (300 mg, crude) which was used in the next step without further purification. LC-MS m/z = 143 [M+1].

Step 3. Synthesis of 6-fluoro-N^4-(4-isopropoxy-5-(1-methyl-1H-pyrazol-4-yl)pyridin-2-yl)-N^2-methylpyrimidine-2,4-diamine.

A mixture of 6-fluoro-*N*^{2}-methylpyrimidine-2,4-diamine (100 mg, 0.70 mmol), 2-chloro-5-(1-methyl-1*H*-pyrazol-4-yl)-4-(propan-2-yloxy)pyridine **SI-1** (118 mg, 0.47 mmol), $Pd_2(dba)_3$ (43 mg, 47 μmol), XantPhos 54 mg, 94 μmol), and Cs_2CO_3 (154 mg, 1.41 mmol) in dioxane (8 mL) was stirred at 100 ^oC for 14 h under N_2. The reaction mixture was concentrated then purified by silica gel chromatography (EA/MeOH=9:1), then by Prep-HPLC (A:water(10 mM NH_4HCO_3 & 0.025% $NH_3 \cdot H_2O$)), B: CAN; 45% B for 1 min, then 60% B in 7 min, stop at 15 min) to obtain the title product (54.6 mg, 32% yield) as a white solid. LC-MS m/z = 358 [M+1]; ^{1}H NMR (500 MHz, DMSO) δ 9.90 (s, 1H), 8.40 (s, 1H), 8.04 (s, 1H), 7.89 (d, *J* = 0.7 Hz, 1H), 7.82 (s, 1H), 7.25 (s, 1H), 6.32 (s, 1H), 4.78 (s, 1H), 3.88 (s, 3H), 2.87 (s, 2H), 1.41 (d, *J* = 6.0 Hz, 6H).

Compound 4. Synthesis of *N*^{4}-(4-isopropoxy-5-(1-methyl-1*H*-pyrazol-4-yl)pyridin-2-yl)-N^2,6-dimethylpyrimidine-2,4-diamine

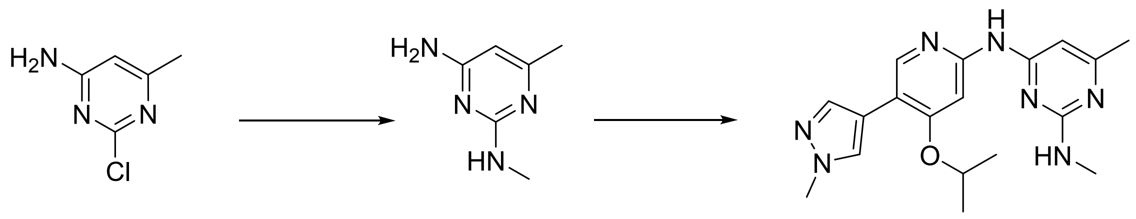

Step 1: Synthesis of N^2,6-dimethylpyrimidine-2,4-diamine.

A mixture of 2-chloro-6-methylpyrimidin-4-amine (1 g, 7.0 mmol) in $MeNH_2$/THF (2.0 M, 10 mL) was stirred at 80 °C for 15 h. The reaction was evaporated in vacuum to give the title product (600 mg, crude) which was used in the next step directly without further purification. LC-MS m/z = 139 [M+1].

Step 2: Synthesis of N⁴-(4-isopropoxy-5-(1-methyl-1H-pyrazol-4-yl)pyridin-2-yl)-N²,6-dimethylpyrimidine-2,4-diamine.

To a mixture of *N*²,6-dimethylpyrimidine-2,4-diamine (42 mg, 0.30 mmol) and 2-chloro-5-(1-methyl-1*H*-pyrazol-4-yl)-4-(propan-2-yloxy)pyridine **SI-1** (50 mg, 0.20 mmol) in dioxane (5 mL) was added $Pd_2(dba)_3$ (18 mg, 20 µmol), XantPhos (23 mg, 40 µmol), and Cs_2CO_3 (196 mg, 0.60 mmol) at room temperature. The reaction mixture was stirred at 100 °C for 14 h under N_2. The reaction mixture was concentrated and the residue was purified by silica gel chromatography (EA/MeOH=9:1) then by Prep-HPLC (A:water(10 mM NH_4HCO_3 & 0.025% $NH_3 \cdot H_2O$)), B: CAN; 45% B for 1 min, then 60% B in 7 min, stop at 15 min) to obtain the title product (56.1 mg, 79% yield) as a white solid. LC-MS m/z = 354 [M+1]; ^{1}H NMR (500 MHz, DMSO) δ 9.51 (s, 1H), 8.36 (s, 1H), 8.02 (s, 1H), 7.96 (s, 1H), 7.87 (d, *J* = 0.7 Hz, 1H), 6.65 (s, 1H), 6.43 (s, 1H), 4.88 – 4.71 (m, 1H), 3.87 (s, 3H), 2.84 (d, *J* = 4.7 Hz, 3H), 2.12 (s, 3H), 1.39 (d, *J* = 6.0 Hz, 6H).

Compound 5. Synthesis of *N*⁴-(4-isopropoxy-5-(1-methyl-1*H*-pyrazol-4-yl)pyridin-2-yl)-6-methoxy-*N*²-methylpyrimidine-2,4-diamine

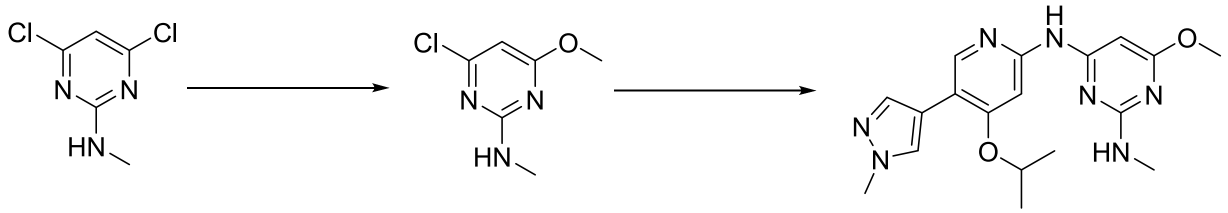

Step 1: Synthesis of 4-chloro-6-methoxy-N-methylpyrimidin-2-amine.

A mixture of 4,6-dichloro-*N*-methylpyrimidin-2-amine (450 mg, 2.54 mmol) and MeONa (686 mg, 12.7 mmol) in MeOH (10 mL) was stirred at 60 °C for 15 h. The reaction mixture was concentrated and the residue was purified by silica gel chromatography (PE/EA=2:1) to obtain the title product (400 mg, 90% yield) as a yellow solid. LC-MS m/z = 174 [M+1].

Step 2: Synthesis of N4-(4-isopropoxy-5-(1-methyl-1H-pyrazol-4-yl)pyridin-2-yl)-6-methoxy-N2-methylpyrimidine-2,4-diamine.

A mixture of 4-chloro-6-methoxy-*N*-methylpyrimidin-2-amine (119 mg, 0.68 mmol), 4-isopropoxy-5-(1-methyl-1*H*-pyrazol-4-yl)pyridin-2-amine **SI-3** (80 mg, 0.34 mmol), Pd(t-$Bu_3P)_2$ (30 mg, 68 μmol), and t-BuONa (98 mg, 1.02 mmol) in toluene (8 mL) was stirred at 80 °C for 14 h under N_2. The reaction mixture was concentrated and the residue was purified by silica gel chromatography (EA/MeOH=9:1) to obtain the crude product which was further purified with Prep-HPLC (A:water(10 mM NH_4HCO_3 & 0.025% $NH_3 \cdot H_2O$)), B: CAN; 45% B for 1 min, then 60% B in 7 min, stop at 15 min) to obtain the title product (9.0 mg, 7% yield) as a white solid. LC-MS m/z = 370 [M+1]; 1H NMR (400 MHz, DMSO-d6) δ: 9.40 (s, 1H), 8.34 (s, 1H), 8.02 (s, 1H), 7.87 (s, 1H), 7.80 (s, 1H), 6.77 (s, 1 H), 6.14 (s, 1H), 4.80 (s, 1H), 3.87 (s, 3H), 3.77 (s, 3H), 2.85 (d, *J* = 4.7 Hz, 3H), 1.40 (d, *J* = 6.0 Hz, 6H).

Compound 6. Synthesis of *N*4-(4-isopropoxy-5-(1-methyl-1*H*-pyrazol-4-yl)pyridin-2-yl)-N2-methylpyrimidine-2,4,6-triamine

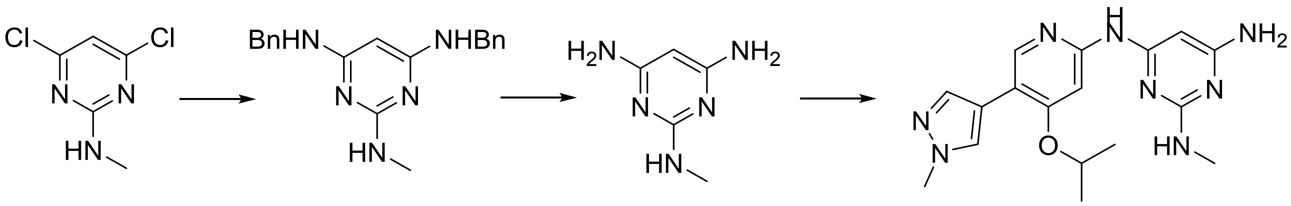

Step 1: Synthesis of N4,N6-dibenzyl-N2-methylpyrimidine-2,4,6-triamine.

To a solution of 4,6-dichloro-*N*-methylpyrimidin-2-amine (1.0 g, 5.65 mmol) in benzylamine (8 mL) was stirred at 200 °C under microwave for 1 h. LC-MS showed the reaction was completed. The mixture was purified by flash column chromatography (PE/EtOAc = 1/3) to afford the title compound (1.0 g, 56% yield) as a yellow solid. LC-MS m/z = 320 [M+1].

Step 2: Synthesis of N²-methylpyrimidine-2,4,6-triamine

To a solution of *N*⁴,*N*⁶-dibenzyl-*N*²-methylpyrimidine-2,4,6-triamine (1.0 g, 3.13 mmol) in DCM (10 mL) was added trifluoromethanesulfonic acid (5 mL) slowly at 0 °C and stirred at 0 °C for 1 h. The mixture was basified with 10% NaOH to pH ~ 10. Then the mixture was evaporated *in vacuo* and the residue was purified by flash column chromatography (DCM /MeOH = 10/1) to afford the title compound (380 mg, 87% yield) as a white solid. LC-MS m/z = 140 [M+1].

Step 3: Synthesis of N⁴-(4-isopropoxy-5-(1-methyl-1H-pyrazol-4-yl)pyridin-2-yl)-N²-methylpyrimidine-2,4,6-triamine.

A mixture of *N*²-methylpyrimidine-2,4,6-triamine (250 mg, 1.80 mmol), 2-chloro-4-isopropoxy-5-(1-methyl-1*H*-pyrazol-4-yl)pyridine **SI-1** (300 mg, 1.20 mmol), BrettPhos Pd G3 (110 mg, 0.12 mmol), and KOAc (353 mg, 3.60 mmol) in dioxane (25 mL) was stirred at 100 °C for 16 h under a nitrogen atmosphere. The reaction mixture was concentrated and the residue was purified by silica gel chromatography (EA/MeOH=6:1) to obtain the crude product which was further purified with Prep-HPLC (A:water(10 mM NH_4HCO_3 & 0.025% $NH_3 \cdot H_2O$)), B: CAN; 45% B for 1 min, then 60% B in 7 min, stop at 15 min) to obtain the title product (67.8 mg, 15% yield) as a white solid. LC-MS m/z = 355 [M+1]; ^{1}H NMR (500 MHz, DMSO) δ 9.03 (s, 1H), 8.28 (s, 1H), 8.00 (s, 1H), 7.85 (d, *J* = 0.9 Hz, 1H), 7.79 (s, 1H), 6.03 (s, 1H), 5.94 (s, 2H), 5.85 (s, 1H), 4.75 (p, *J* = 6.0 Hz, 1H), 3.87 (s, 3H), 2.79 (d, *J* = 4.8 Hz, 3H), 1.39 (d, *J* = 6.0 Hz, 6H).

Compound 7. Synthesis of *N*⁴-(4-isopropoxy-5-(1-methyl-1*H*-pyrazol-4-yl)pyridin-2-yl)-2-methylpyrimidine-4,6-diamine

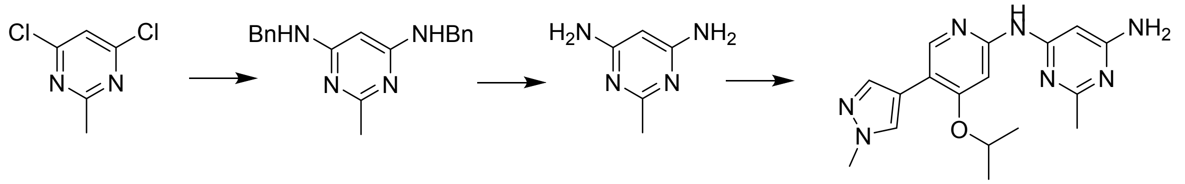

Step 1: Synthesis of N^4,N^6-dibenzyl-2-methylpyrimidine-4,6-diamine.

A solution of 4,6-dichloro-2-methylpyrimidine (1.00 g, 6.13 mmol) in benzylamine (8 mL) was stirred at 130 °C for 16 h. The mixture was filtered and the filtrate was concentrated *in vacuo,* then purified by reversed-phase chromatography (0.1% $NH_3.H_2O$ in water/ACN) to give the title compound (1.30 g, 55% yield) as a yellow solid.

Step 2: Synthesis of 2-methylpyrimidine-4,6-diamine.

A mixture of N^4,N^6-dibenzyl-2-methylpyrimidine-4,6-diamine (1.00 g, 3.29 mmol) in dichloromethane (8 mL) was added to trifluoromethanesulfonic acid (6.80 g, 45.31 mmol). The mixture was stirred at 25 °C for 0.5 h. The reaction mixture was then basified with 10% sodium hydroxide to pH ~ 10. The mixture was concentrated under reduced pressure then purified by silica gel chromatography (dichloromethane/methanol = 10/1)) to give the title compound (200 mg, 49% yield) as a yellow solid. ^{1}H NMR (400 MHz, 6*d*-DMSO) δ ppm 6.10 (s, 4H), 5.25 (s, 1H), 2.11 (s, 3H).

Step 3: Synthesis of N^4-(4-isopropoxy-5-(1-methyl-1H-pyrazol-4-yl)pyridin-2-yl)-2-methylpyrimidine-4,6-diamine.

To a solution of 2-methylpyrimidine-4,6-diamine (73.9 mg, 596 μmol) and **SI-1** (50.0 mg, 199 μmol) in dioxane (3 mL) was added Cs_2CO_3 (194 mg, 596 μmol), BrettPhos Pd G4 (18.29 mg, 19.86 μmol), and BrettPhos (10.66 mg, 19.86 μmol). The mixture was stirred at 100 °C for 1 h. The reaction mixture was concentrated under reduced pressure then purified by prep-HPLC (column: Waters Xbridge C18 150 x 50 mm x 10 μm; mobile phase: [water(10 mM NH_4HCO_3)-ACN]; B%: 15%-45%,10 min) to give the title compound (13.0 mg, 19% yield) as a white solid. LC-MS m/z = 340 [M+1]; ^{1}H NMR (400 MHz, $CDCl_3$) δ 8.34 (s, 1H), 7.88 (s, 1H), 7.77 (s, 1H), 7.67 (s, 1H), 7.20 (s, 1H), 6.58 (s, 1H), 4.82 (s, 2H), 4.74 (p, *J* = 6.1 Hz, 1H), 3.98 (s, 3H), 2.47 (s, 3H), 1.49 (d, *J* = 6.0 Hz, 6H).

Compound 8. Synthesis of 2-cyclopropyl-*N*[4]-(4-isopropoxy-5-(1-methyl-1*H*-pyrazol-4-yl)pyridin-2-yl)pyrimidine-4,6-diamine

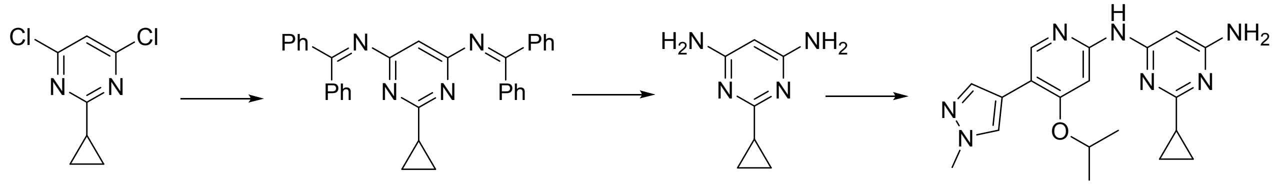

Step 1: Synthesis of N,N'-(2-cyclopropylpyrimidine-4,6-diyl)bis(1,1-diphenylmethanimine).

A mixture of 4,6-dichloro-2-cyclopropylpyrimidine (650 mg, 3.45 mmol), benzophenone imine (1.87 g, 10.3 mmol), $Pd_2(dba)_3$ (311 mg, 0.34 mmol), XantPhos (393 mg, 0.68 mmol), and Cs_2CO_3 (3.37 g, 10.3 mmol) in dioxane (50 mL) was stirred at 100 ^oC for 16 h under a nitrogen atmosphere. The reaction mixture was cooled to room temperature and diluted with dioxane, filtered, and the filtrate was directly concentrated to dryness under reduced. The resulting crude product was purified by silica gel chromatography eluting with DCM/MeOH (15/1) to afford the title compound (400 mg, 24% yield) as a yellow solid. LC-MS m/z = 479 [M+1].

Step 2: Synthesis of 2-cyclopropylpyrimidine-4,6-diamine.

To a solution of N,N'-(2-cyclopropylpyrimidine-4,6-diyl)bis(1,1-diphenylmethanimine (400 mg, 0.836 mmol) in methanol (10 mL) was added hydroxylamine (0.16 g, 50% wt in water, 2.51 mmol) at room temperature under a nitrogen atmosphere. After addition, the reaction mixture was heated to 70 ^oC and stirred for 12 h. The reaction mixture was directly concentrated under reduced pressure. The residue was dissolved into DCM and washed with water. The aqueous layer was basified with $NaHCO_3$ to pH > 10, then extracted with DCM. The combined organic layers were concentrated under reduced pressure then purified by flash column chromatography on silica gel eluting with

DCM/MeOH (10/1) to afford the title compound (70 mg, 56% yield) as a yellow solid. LC-MS m/z = 151 [M+1].

Step 3. Synthesis of 2-cyclopropyl-N[4]-(4-isopropoxy-5-(1-methyl-1H-pyrazol-4-yl)pyridin-2-yl)pyrimidine-4,6-diamine.

A mixture of 2-cyclopropylpyrimidine-4,6-diamine (70 mg, 0.46 mmol), 2-chloro-4-isopropoxy-5-(1-methyl-1*H*-pyrazol-4-yl)pyridine **SI-1** (117 mg, 0.46 mmol), $Pd_2(dba)_3$ (82 mg, 0.09 mmol), XantPhos (104 mg, 0.18 mmol), and Cs_2CO_3 (451 mg, 1.38 mmol) in dioxane (5 mL) was stirred at 100 ^oC for 16 h under a nitrogen atmosphere. The reaction mixture was cooled to room temperature and diluted with dioxane, filtered, and the filtrate was concentrated under reduced pressure and purified by flash column chromatography on silica gel eluting with DCM/MeOH (5/1) to afford the title compound (17.5 mg, 10% yield) as a white solid. LC-MS m/z = 366 [M+1]; ^1H NMR (500 MHz, DMSO) δ 9.35 (s, 1H), 8.36 (s, 1H), 8.06 (d, *J* = 0.7 Hz, 1H), 7.91 (d, *J* = 0.8 Hz, 1H), 7.59 (s, 1H), 6.50 (s, 1H), 6.39 (s, 2H), 4.77 (p, *J* = 6.0 Hz, 1H), 3.93 (s, 3H), 1.88 (tt, *J* = 8.1, 4.7 Hz, 1H), 1.47 (d, *J* = 6.0 Hz, 6H), 0.98 (dt, *J* = 5.4, 2.8 Hz, 2H), 0.92 (dt, *J* = 8.3, 3.1 Hz, 2H).

Compound 9. Synthesis of *N*[4]-(5-isopropyl-8-methoxy-2,7-naphthyridin-3-yl)-N[2]-methylpyrimidine-2,4,6-triamine.

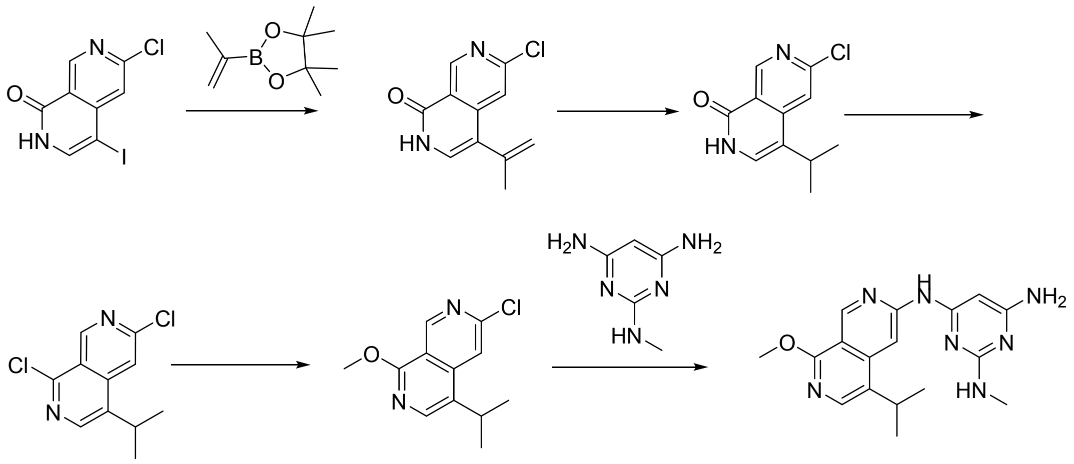

Step 1: Synthesis of 6-chloro-4-(prop-1-en-2-yl)-2,7-naphthyridin-1(2H)-one.

A mixture of 6-chloro-4-iodo-1,2-dihydro-2,7-naphthyridin-1-one (3.0 g, 9.8 mmol), 4,4,5,5-tetramethyl-2-(prop-1-en-2-yl)-1,3,2-dioxaborolane (2.45 g, 14.6 mmol), TEA (2.97 g, 29.4 mmol), and Pd(dppf)Cl_2 (0.72 g, 0.98 mol) in THF/DMF/water (60 ml/ 20 mL/20 mL) was heated to 70 °C for 3 h under N_2. The reaction mixture was filtered, and the filtrate was extracted with EtOAc. The combined organic layers were concentrated under reduced pressure and purified by silica gel chromatography eluting with PE/EtOAc (1:1) to afford the title compound (1.3 g, 60% yield) as a light brown solid. LC-MS m/z = 221 [M+1].

Step 2: Synthesis of 6-chloro-4-isopropyl-2,7-naphthyridin-1(2H)-one.

A mixture of 6-chloro-4-(prop-1-en-2-yl)-1,2-dihydro-2,7-naphthyridin-1-one (1.3 g, 5.9 mmol) and PtO_2 (1.3 g, 5.7 mmol) in EtOAc (50 mL) was stirred at room temperature for 1.5 h under H_2 atmosphere. The reaction was filtered, and the filtrate was concentrated under reduced pressure to give the title compound (800 mg, crude), which was used for the next step without further purification. LC-MS m/z = 223 [M+1].

Step 3: Synthesis of 1,6-dichloro-4-isopropyl-2,7-naphthyridine.

A mixture of 6-chloro-4-isopropyl-2,7-naphthyridin-1(2*H*)-one (800 mg, 3.60 mmol) and $POCl_3$ (1.67 mL, 18 mmol) in toluene (20 mL) was stirred at 110 ^oC for 3 h. LC-MS showed the reaction was completed. The reaction mixture was diluted with DCM and washed with water and brine. The organic layer was concentrated under reduced pressure and purified by flash column chromatography on silica gel eluting with DCM/MeOH (10/1) to give the title compound (400 mg, 46% yield) as a yellow solid. LC-MS m/z = 241 [M+1].

Step 4: Synthesis of 6-chloro-4-isopropyl-1-methoxy-2,7-naphthyridine.

A mixture of 1,6-dichloro-4-isopropyl-2,7-naphthyridine (400 mg, 1.67 mmol) and K_2CO_3 (228 mg, 2.5 mmol) in MeOH (5 mL) was stirred at room temperature for 3 h. The reaction mixture was diluted with DCM and washed with water and brine. The organic layer was concentrated, and the residue was purified by flash column chromatography on silica gel eluting with PE/EA (1/1) to give the title compound (200 mg, 50% yield) as a yellow solid. LC-MS m/z = 237 [M+1].

Step 5: Synthesis of N^4-(5-isopropyl-8-methoxy-2,7-naphthyridin-3-yl)-N^2-methylpyrimidine-2,4,6-triamine.

A mixture of *N*^{2}-methylpyrimidine-2,4,6-triamine (70 mg, 0.50 mmol), 6-chloro-4-isopropyl-1-methoxy-2,7-naphthyridine (60 mg, 0.25 mmol), $Pd_2(dba)_3$ (45 mg, 0.05 mmol), XantPhos (58 mg, 0.10 mmol), and Cs_2CO_3 (245 mg, 0.75 mmol) in dioxane (5 mL) was stirred at 100 ^oC for 5 h under a nitrogen atmosphere. The reaction mixture was concentrated under reduced pressure and purified by silica gel chromatography (EA/MeOH=3:1) then Prep-HPLC (A:water(10 mM NH_4HCO_3 & 0.025% $NH_3 \cdot H_2O$)), B: CAN; 45% B for 1 min, then 60% B in 7 min, stop at 15 min) to obtain the title product (7.3 mg, 8% yield) as a white solid. LC-MS m/z = 340 [M+1]; ^{1}H NMR (500 MHz, DMSO) δ 9.72 (s, 1H), 9.20 (s, 1H), 8.75 (s, 1H), 7.94 (s, 1H), 6.27 (s, 1H), 6.10 (s, 2H), 5.64 (s, 1H), 4.03 (s, 3H), 3.43 – 3.35 (m, 1H), 2.89 (d, *J* = 4.7 Hz, 3H), 1.32 (d, *J* = 6.8 Hz, 6H).

Compound 10. Synthesis of 6-((6-amino-2-(methylamino)pyrimidin-4-yl)amino)-4-(isopropylamino)-N-methylnicotinamide

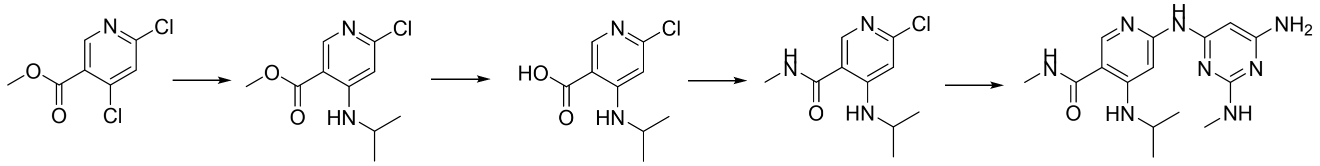

Step 1: Synthesis of methyl 6-chloro-4-(isopropylamino)nicotinate.

To a solution of methyl 4,6-dichloropyridine-3-carboxylate (50 g, 243 mmol) and propan-2-amine (43.0 g, 728 mmol) in CH_3CN (500 mL) was added DIPEA (94.0 g, 728 mmol) at room temperature under a nitrogen atmosphere. After 16 h, the reaction mixture was concentrated under reduced pressure. The residue was dissolved in ethyl acetate, washed with water and brine. The organic layer was dried over anhydrous sodium sulfate, filtered, and the filtrate was concentrated under reduced pressure. The residue was purified by flash column chromatography on silica gel eluting with PE/EtOAc (3/1) to give the title compound (42 g, 76% yield) as a yellow solid. LC-MS m/z = 229 [M+1].

Step 2: Synthesis of 6-chloro-4-(isopropylamino)nicotinic acid.

To a solution of methyl 6-chloro-4-(isopropylamino)nicotinate (42 g, 183.7 mmol) in THF (200 mL), MeOH (120 mL), and H_2O (80 mL) was added $LiOH.H_2O$ (15.4 g, 367 mmol) at 0 ^oC under a nitrogen atmosphere. The reaction mixture was then allowed to warm to room temperature and stirred for 12 hours. The reaction mixture was acidified by adding 2M HCl (aq.) until pH 5~6. The precipitate was collected by filtration, then dried in vacuum to give the title compound (36.5 g, 92% yield) as a white solid which was used for next step without further purification. LC-MS m/z = 215 [M+1].

Step 3: Synthesis of 6-chloro-4-(isopropylamino)-N-methylnicotinamide.

To a solution of methanamine hydrochloride (17.2 g, 255 mmol), Et_3N (51.5 g, 510 mmol), and 6-chloro-4-(isopropylamino)nicotinic acid (36.5 g, 170 mmol) in DMF (300 mL) was added HATU (97 g, 255 mmol). Then the reaction mixture was stirred for 12 h at room temperature. The organic layer was washed with water and brine, dried over anhydrous sodium sulfate, filtered, and the filtrate was concentrated under reduced pressure. The residue was purified by flash column chromatography on silica gel eluting

with DCM/MeOH (10/1) to give the title compound (26 g, 67% yield) as a white solid. LC-MS m/z = 228 [M+1].

Step 4: Synthesis of 6-((6-amino-2-(methylamino)pyrimidin-4-yl)amino)-4-(isopropylamino)-N-methylnicotinamide.

A mixture of *N*[2]-methylpyrimidine-2,4,6-triamine (97 mg, 0.70 mmol), 6-chloro-4-(isopropylamino)-N-methylnicotinamide (80 mg, 0.35 mmol), X-Phos Pd G2 (24 mg, 0.03 mmol), and Cs_2CO_3 (343 mg, 1.05 mmol) in dioxane (8 mL) was stirred at 100 ^oC for 5 h under a nitrogen atmosphere. The reaction mixture was cooled to room temperature and diluted with dioxane, filtered, and the filtrate was directly concentrated under reduced pressure The residue was purified with Prep-HPLC (A:water(10 mM NH_4HCO_3 & 0.025% $NH_3 \cdot H2O$), B: CAN; 45% B for 1 min, then 60% B in 7 min, stop at 15 min) to obtain the title product (19.5 mg, 17% yield) as a white solid. LC-MS m/z = 331 [M+1]; 1H NMR (400 MHz, DMSO-d6) δ ^1H NMR (500 MHz, DMSO) δ 8.98 (s, 1H), 8.39 (d, *J* = 7.5 Hz, 1H), 8.26 (s, 1H), 8.21 (q, *J* = 4.5 Hz, 1H), 7.42 (s, 1H), 6.02 (s, 1H), 5.93 (s, 2H), 5.74 (s, 1H), 3.65 (h, *J* = 6.5 Hz, 1H), 2.79 (d, *J* = 4.8 Hz, 3H), 2.71 (d, *J* = 4.4 Hz, 3H), 1.19 (d, *J* = 6.3 Hz, 6H).

Compound 11. Synthesis of 6-((6-amino-2-cyclopropylpyrimidin-4-yl)amino)-4-(isopropylamino)-N-methylnicotinamide.

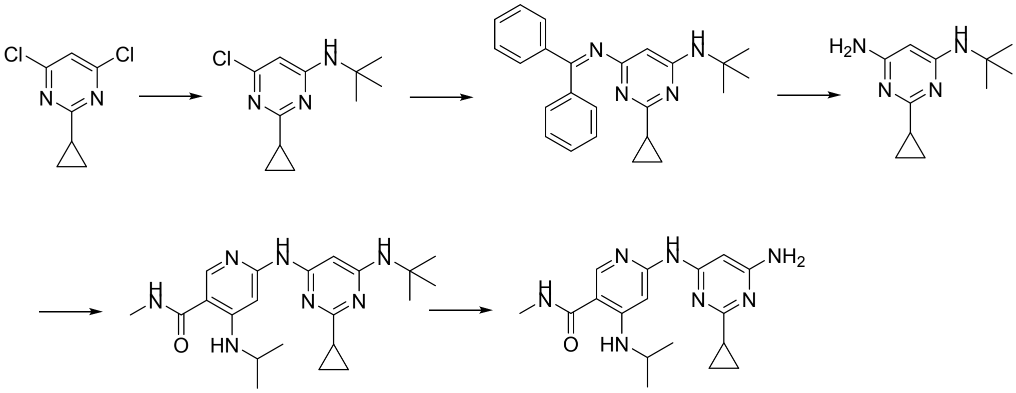

Step 1: Synthesis of N-(tert-butyl)-6-chloro-2-cyclopropylpyrimidin-4-amine.

A mixture of 4,6-dichloro-2-cyclopropylpyrimidine (52.0 g, 275 mmol), 2-methylpropan-2-amine (24.2 g, 330 mmol), and *N*,*N*-diisopropylethylamine (106 g, 825 mmol) in NMP (1.0 L) was stirred at 100 ^{o}C for 1 h. The reaction mixture was cooled to room temperature and diluted with ethyl acetate, then washed with water and brine. The organic layer was dried over anhydrous sodium sulfate, filtered, and the filtrate was concentrated to dryness under reduced pressure. The resulting crude product was purified by flash column chromatography on silica gel eluting with PE/EtOAc (2/1) to afford the title compound (52.2 g, 87% yield) as a white solid. LC-MS m/z = 226 [M+1].

Step 2: Synthesis of N-(tert-butyl)-2-cyclopropyl-6-((diphenylmethylene)amino) pyrimidin-4-amine.

A mixture of *N*-(*tert*-butyl)-6-chloro-2-cyclopropylpyrimidin-4-amine (52.2 g, 231 mmol), benzophenone imine(62.9 g, 346 mmol), $Pd_2(dba)_3$ (21.2 g, 23.1 mmol), XantPhos (13.4 g, 23.1 mol), and Cs_2CO_3 (226 g, 694 mmol) in dioxane (1.2 L) was stirred at 100 ^{o}C for 16 h under a nitrogen atmosphere. The reaction mixture was cooled to room temperature and diluted with dioxane, filtered, and the filtrate was directly concentrated to dryness under reduced pressure. The crude was purified by flash column chromatography on silica gel eluting with DCM/MeOH (15/1) to afford the title compound (57.5 g, 67% yield) as a yellow solid. LC-MS m/z = 371 [M+1].

Step 3: Synthesis of N^4-(tert-butyl)-2-cyclopropylpyrimidine-4,6-diamine.

To a solution of methyl *N*-(*tert*-butyl)-2-cyclopropyl-6-((diphenylmethylene)amino)pyrimidin-4-amine (57.5 g, 155 mmol) in methanol (600 mL) was added hydroxylamine (30.8 g, 50% wt in water, 466 mmol) at room temperature under a nitrogen atmosphere. After addition, the reaction mixture was heated to 70 ^{o}C and stirred for 12 h. The reaction mixture was concentrated under reduced pressure.

The residue was dissolved into DCM and washed with water. The aqueous layer was basified with $NaHCO_3$ to pH > 10, then extracted with DCM. The combined organic layers were concentrated under reduced pressure. The resulting crude product was purified by flash column chromatography on silica gel eluting with DCM/MeOH (10/1) to afford the title compound (20.0 g, 62% yield) as a yellow solid. LC-MS m/z = 207 [M+1].

Step 4: Synthesis of 6-((6-(tert-butylamino)-2-cyclopropylpyrimidin-4-yl)amino)-4-(isopropylamino)-N-methylnicotinamide.

A mixture of N^4-(tert-butyl)-2-cyclopropylpyrimidine-4,6-diamine (20.0 g, 97.0 mmol), 6-chloro-4-(isopropylamino)-N-methylnicotinamide (22.1 g, 97.0 mmol), $Pd(t\text{-}Bu_3P)_2$ (10.0 g, 19.4 mmol), and Cs_2CO_3 (94.8 g, 291 mmol) in dioxane (300 mL) was stirred at 100 ^oC for 16 h under a nitrogen atmosphere. The reaction mixture was cooled to room temperature and diluted with dioxane, filtered, and the filtrate was concentrated under reduced pressure. The residue was purified by flash column chromatography on silica gel eluting with DCM/MeOH (10/1) to afford the title compound (21.5 g, 55% yield) as a yellow solid. LC-MS m/z = 398 [M+1].

Step 5: Synthesis of 6-((6-amino-2-cyclopropylpyrimidin-4-yl)amino)-4-(isopropylamino)-N-methylnicotinamide

A mixture of 6-((6-(tert-butylamino)-2-cyclopropylpyrimidin-4-yl)amino)-4-(isopropylamino)-*N*-methylnicotinamide (21.5 g, 54.1 mmol) in TFA (100 mL) was stirred at 70 ^oC for 16 h. The reaction mixture was concentrated under reduced pressure and basified with a saturated aqueous solution of K_2CO_3 to pH ~ 10. The resulting mixture was concentrated under reduced pressure. The residue was purified by flash column chromatography on silica gel eluting with DCM/MeOH (10/1) to afford the title compound (10.9 g, 59% yield) as a yellow solid. LC-MS m/z = 342 [M+1]. ^{1}HNMR (500 MHz, DMSO-*d6*) δ 9.24 (s, 1H), 8.39 (d, J = 7.3 Hz, 1H), 8.28 (s, 1H), 8.25 (q, J = 4.5 Hz, 1H), 7.21 (s, 1H), 6.32 (s, 2H), 6.29 (s, 1H), 3.62 (h, J = 6.5 Hz, 1H), 2.72 (d, J = 4.5

Hz, 3H), 1.81 (tt, *J* = 8.1, 4.7 Hz, 1H), 1.22 (d, *J* = 6.3 Hz, 6H), 0.92 (dt, *J* = 5.1, 2.8 Hz, 2H), 0.86 (dt, *J* = 8.2, 3.0 Hz, 2H).

Compound 12. Synthesis of 6-((6-amino-2-(difluoromethyl)pyrimidin-4-yl)amino)-4-(isopropylamino)-N-methylnicotinamide

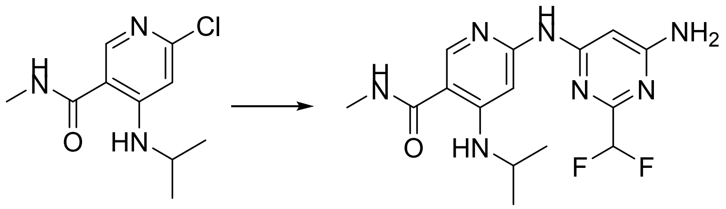

The following compound was synthesized following procedures similar to that described for compound 10, step 4 above using 6-chloro-4-(isopropylamino)-*N*-methylnicotinamide and 2-(difluoromethyl)pyrimidine-4,6-diamine and purifying by flash column chromatography on silica gel eluting with 0-20% MeOH/DCM to afford the title compound (13.8 mg, 6.8% yield) LC-MS m/z = 352 [M+1]. ^{1}H NMR (400 MHz, DMSO-d_6) δ 10.35 (s, 1H), 8.82 (s, 1H), 8.42 (s, 1H), 8.33 (s, 1H), 7.05 (s, 2H), 6.72 (d, J = 6.2 Hz, 1H), 6.51 (d, J = 54.7 Hz, 1H), 6.31 (s, 1H), 3.74 – 3.61 (m, 2H), 2.75 (d, J = 4.4 Hz, 3H), 1.24 (d, J = 6.3 Hz, 6H).

Compound 13. Synthesis of (S)-6-((6-amino-2-(difluoromethyl)pyrimidin-4-yl)amino)-4-((1-fluoropropan-2-yl)amino)-N-methylnicotinamide

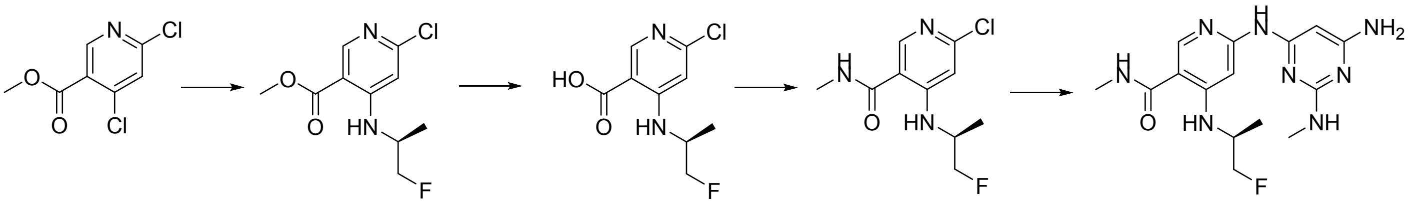

Step 1: Synthesis of methyl (S)-6-chloro-4-((1-fluoropropan-2-yl)amino)nicotinate.

A mixture of methyl 4,6-dichloropyridine-3-carboxylate (300 mg, 1.45 mmol), (2*S*)-1-fluoropropan-2-amine hydrochloride (246 mg, 2.17 mmol), and DIPEA (561 mg, 4.35 mmol) in dioxane (5 mL) was stirred at 140 °C for 16 h. The cooled reaction was quenched with water and extracted with EtOAc. The organic layer was washed with water and brine, dried over Na_2SO_4, filtered, and concentrated. The residue was purified by column chromatography on silica gel eluting with EtOAc/PE (1/2) to get the title compound (200 mg, 53% yield) as a white solid. LC-MS m/z = 247 [M+1].

Step 2: Synthesis of (S)-6-chloro-4-((1-fluoropropan-2-yl)amino)nicotinic acid.

A mixture of methyl (*S*)-6-chloro-4-((1-fluoropropan-2-yl)amino)nicotinate (200 mg, 0.81 mmol), LiOH (57.9 mg, 2.42 mmol), and H_2O (1 mL) in THF (4 mL) was stirred at room temperature for 16 h. HCl (1N) was added to adjust the pH ~ 3, the mixture was diluted with water and extracted with EtOAc. The combined organic layers were dried over Na_2SO_4, filtered, and concentrated to give the title compound (200 mg, 100% yield) as a white solid used as is without purification. LC-MS m/z = 233 [M+1].

Step 3: Synthesis of (S)-6-chloro-4-((1-fluoropropan-2-yl)amino)-N-methyl nicotinamide.

A mixture of 4,6-dichloro-*N*-methylnicotinamide (200 mg, 0.975 mmol), (2S)-1-fluoropropan-2-amine hydrochloride (110 mg, 0.975 mmol), and DIPEA (376 mg, 2.92 mmol) in dioxane (5 mL) was stirred in a sealed tube at 140 °C for 16 h. The reaction was quenched with H_2O and extracted into EtOAc. The combined organics were washed (H_2O and brine), dried (Na_2SO_4), and concentrated under reduced pressure. The residue was purified by column chromatography on silica gel (10:1 to 1:1 PE/EtOAc) to afford the title compound (210 mg, 82% yield) as a white solid. LC-MS m/z = 246 [M+1].

Step 4: Synthesis of (S)-6-((6-amino-2-(difluoromethyl)pyrimidin-4-yl)amino)-4-((1-fluoropropan-2-yl)amino)-N-methylnicotinamide.

A mixture of (*S*)-6-chloro-4-((1-fluoropropan-2-yl)amino)-*N*-methyl nicotinamide (150 mg, 610 μmol), 2-(difluoromethyl)pyrimidine-4,6-diamine (117 mg, 731 μmol), Cs_2CO_3 (397 mg, 1.22 mmol), and $Pd(tBu_3P)_2$ (155 mg, 305 μmol) in dioxane (4 mL) was stirred at 100 °C for 16 h under N_2. The reaction mixture was purified by flash chromatography on silica gel eluting with MeOH/DCM 1:10 then by Prep-HPLC (Mobile phase: A = water(0.1% NH4HCO3), B = acetonitrile; Gradient: B = 15%-95% in 18 min; Column: Xtimate 10um 150A 21.2×250mm) to afford the title compound (41 mg, 18% yield) as a white solid. LC-MS m/z = 370 [M+1]. 1H-NMR (400 MHz, DMSO-d6) δ ^{1}H NMR (500 MHz, DMSO) δ 9.69 (s, 1H), 8.55 (d, *J* = 7.6 Hz, 1H), 8.35 (d, *J* = 6.1 Hz, 2H), 7.11 (s, 1H), 6.91 (s, 2H), 6.81 (s, 1H), 6.49 (t, *J* = 54.9 Hz, 1H), 4.53 (qd, *J* = 9.4, 4.5 Hz, 1H), 4.43 (qd, *J* = 9.3, 4.5 Hz, 1H), 3.82 – 3.66 (m, 1H), 2.73 (d, *J* = 4.4 Hz, 3H), 1.23 (dd, *J* = 6.5, 1.2 Hz, 3H).

Compound 14. Synthesis of 2-(difluoromethyl)-*N*[4]-(4-isopropoxy-5-(1-methyl-1*H*-pyrazol-4-yl)pyridin-2-yl)pyrimidine-4,6-diamine.

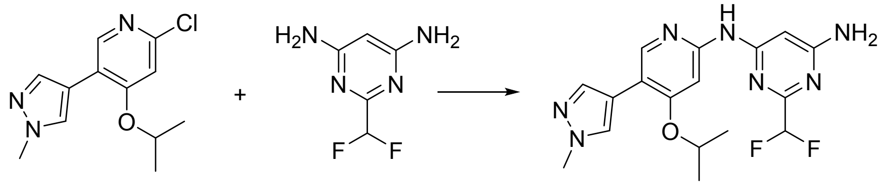

To a mixture of 2-(difluoromethyl)pyrimidine-4,6-diamine hydrochloride (**SI-4**) (25.00 mg, 156.13 mmol,) and 2-chloro-4-isopropoxy-5-(1-methyl-1*H*-pyrazol-4-yl)pyridine (**SI-1**) (39.30 mg, 156.13 mmol) in dioxane (2.00 mL) was added BrettPhos Pd G_4 (14.37 mg, 15.61 mmol) and Cs_2CO_3 (101.74 mg, 312.26 mmol). The reaction was heated at 90 °C for 2 h under N_2. The reaction mixture was concentrated under reduced pressure then purified by *prep*-HPLC (column: Phenomenex Gemini-NX C18 75*30mm*3um;mobile phase: [water(0.04%NH_3H_2O+10mM NH_4HCO_3)-ACN]; B%:

25%-55%, 8min) to give the title compound (22.40 mg, 37.1% yield) as a white solid. LC-MS m/z = 376 [M+1]; ^{1}H NMR (500 MHz, DMSO) δ 9.73 (s, 1H), 8.34 (s, 1H), 8.03 (s, 1H), 7.88 (d, *J* = 0.8 Hz, 1H), 7.40 (s, 1H), 6.91 (d, *J* = 5.0 Hz, 3H), 6.50 (t, *J* = 54.9 Hz, 1H), 4.68 (p, *J* = 6.0 Hz, 1H), 3.88 (s, 3H), 1.41 (d, *J* = 6.0 Hz, 6H).

Compound 15. Synthesis of 2-(difluoromethyl)-*N*^{4}-(4-methoxy-5-(1-methyl-1*H*-pyrazol-4-yl)pyridin-2-yl)pyrimidine-4,6-diamine.

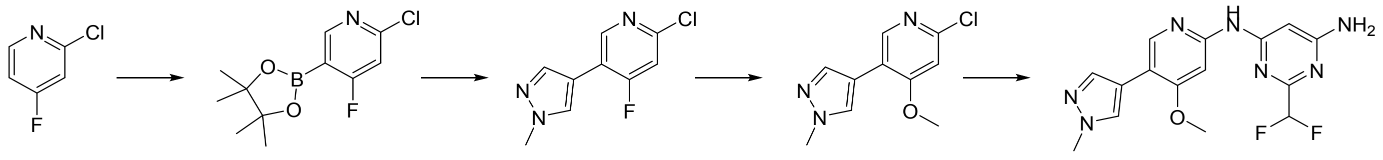

Step 1: Synthesis of 2-chloro-4-fluoro-5-(4,4,5,5-tetramethyl-1,3,2-dioxaborolan-2-yl)pyridine.

A mixture of 2-chloro-4-fluoropyridine (10 g, 76.0 mmol), bis(pinacolato)diboron (9.64 g, 38.0 mmol), $[Ir(OMe)(1,5\text{-cod})]_2$ (251 mg, 0.380 mmol), and dtbpy (270 mg, 0.760 mmol) in THF (150 mL) was stirred at 80 °C for 16 h. The mixture was concentrated *in vacuo* and the residue was purified by column chromatography on silica gel eluting with EtOAc/PE (1/10) to afford the title compound (15 g, 77% yield) as colorless oil. LC-MS m/z = 258 [M+1].

Step 2: Synthesis of 2-chloro-4-fluoro-5-(1-methyl-1H-pyrazol-4-yl)pyridine.

A mixture of 2-chloro-4-fluoro-5-(4,4,5,5-tetramethyl-1,3,2-dioxaborolan-2-yl)pyridine (15 g, 58.36 mmol), 4-bromo-1-methyl-1*H*-pyrazole (9.4 g, 58.36 mmol), K_2CO_3 (16.1 g, 116.73 mmol), and $Pd(dppf)Cl_2$ (4.27 g, 5.83 mmol) in dioxane/water (80 mL/ 20 mL) was stirred at 80 ^{o}C for 6 h. The mixture was concentrated *in vacuo* and the residue was purified by column chromatography on silica gel eluting with EtOAc/PE (1/2) to afford the title compound (5 g, 41% yield) as a brown solid. ^{1}H-NMR (400 MHz, DMSO-

d_6) δ ppm 8.83-8.80 (m, 1H), 8.27-8.26 (m, 1H), 8.00 (s, 1H), 7.69-7.67 (m, 1H), 3.90 (s, 3H).

Step 3. Synthesis of 2-chloro-4-methoxy-5-(1-methyl-1H-pyrazol-4-yl)pyridine.

To a mixture of 2-chloro-4-fluoro-5-(1-methyl-1*H*-pyrazol-4-yl)pyridine (73.7 mg, 0.3 mmol) and MeOH (14.4 mg, 0.45 mmol) in THF (4 mL) was added t-BuOK (101 mg, 0.9 mmol) and the mixture was stirred at 100 °C for 3 h. The reaction mixture was concentrated by Speedvac to afford 2-chloro-4-methoxy-5-(1-methyl-1*H*-pyrazol-4-yl)pyridine which was used in the following step without further purification.

Step 4: Synthesis of 2-(difluoromethyl)-N^4-(4-methoxy-5-(1-methyl-1H-pyrazol-4-yl)pyridin-2-yl)pyrimidine-4,6-diamine.

To a solution 2-chloro-4-methoxy-5-(1-methyl-1*H*-pyrazol-4-yl)pyridine (0.27 mmol) and 2-(difluoromethyl)pyrimidine-4,6-diamine (51.8 g, 0.32 mmol) in *t*-AmOH (3 mL) was added Cs_2CO_3 (263.9 g, 0.81 mmol) and BrettPhos Pd G3 (12.24 mg, 0.014 mmol) and the mixture was stirred at 120 ºC for 2 h under N_2. The reaction was diluted with H_2O (3.0 mL) and extracted with EtOAc (3 x 10 mL). The combined organic layers were evaporated to dryness by Speedvac and the residue purified by prep-HPLC-C to give the title compound (14 mg, 9% yield); LC-MS m/z = 348 [M+1]; ^{1}H NMR (500 MHz, DMSO) δ 9.79 (s, 1H), 8.34 (s, 1H), 8.06 (s, 1H), 7.87 (d, *J* = 0.8 Hz, 1H), 7.35 (s, 1H), 6.96 (s, 1H), 6.90 (s, 2H), 6.50 (t, *J* = 54.9 Hz, 1H), 3.89 (s, 3H), 3.86 (s, 3H).

Compound 16. Synthesis of 2-(difluoromethyl)-*N*[4]-(4-methoxy-5-(1-(2-(methylamino)ethyl)-1*H*-pyrazol-4-yl)pyridin-2-yl)pyrimidine-4,6-diamine.

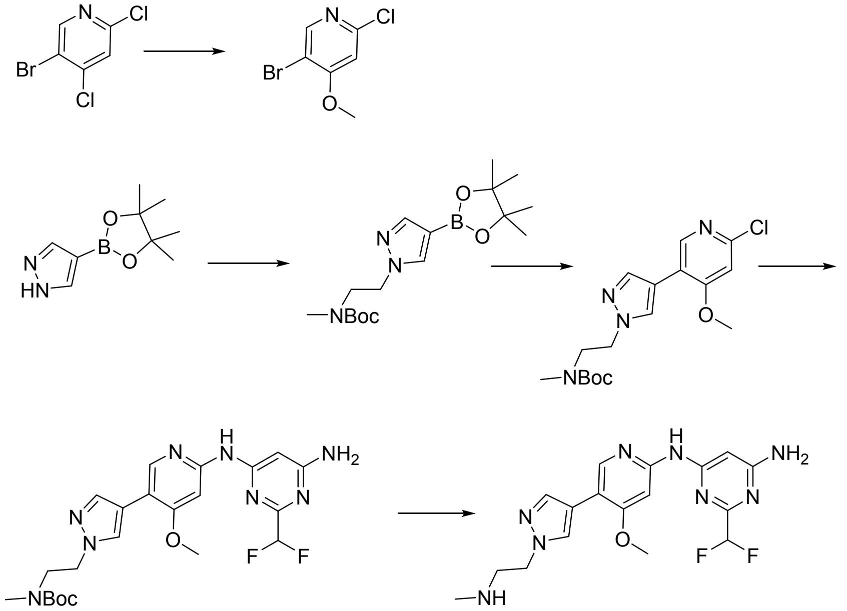

Step 1: Synthesis of 5-bromo-2-chloro-4-methoxypyridine.

To a solution of MeOH (704 mg, 22.0 mmol) in THF (30 mL) at 0 °C was added NaH (60% dispersion, 1.76 g, 44.0 mmol). The mixture was stirred at 0 °C for 30 min, then 5-bromo-2,4-dichloropyridine (5 g, 22.0 mmol) in THF (10 mL) was added. The reaction was stirred at room temperature for 12 h. The solution was quenched with H_2O, extracted with EtOAc, and the combined organic layers were concentrated *in vacuo*. The residue was purified by silica gel chromatography to give the title compound as a white solid (3.5 g, 72%). LC-MS m/z = 222 $[M+H]^+$.

Step 2: Synthesis of tert-butyl methyl(2-(4-(4,4,5,5-tetramethyl-1,3,2-dioxaborolan-2-yl)-1H-pyrazol-1-yl)ethyl)carbamate.

To a solution of 4-(4,4,5,5-tetramethyl-1,3,2-dioxaborolan-2-yl)-1*H*-pyrazole (4.50 g, 23.19 mmol) and tert-butyl *N*-(2-hydroxyethyl)-*N*-methyl-carbamate (4.06 g, 23.19

mmol) in THF (30 mL) was added DIAD (5.16 g, 25.51 mmol) and PPh_3 (6.69 g, 25.51 mmol). The reaction was stirred at room temperature for 12 h under N_2. The reaction mixture was concentrated under reduced pressure and the residue was purified by prep-HPLC-A to give the title compound (2.11 g, 25.9%) as a yellow oil.

Step 3: Synthesis of tert-butyl (2-(4-(6-chloro-4-methoxypyridin-3-yl)-1H-pyrazol-1-yl)ethyl)(methyl)carbamate.

To a solution of 5-bromo-2-chloro-4-methoxypyridine (46 mg, 0.207 mmol) and tert-butyl methyl(2-(4-(4,4,5,5-tetramethyl-1,3,2-dioxaborolan-2-yl)-1*H*-pyrazol-1-yl)ethyl)carbamate (79.89 mg, 0.227 mmol) in EtOH (3 mL) and H_2O (0.20 mL) was added Pd(amphos)Cl_2 (14.64 mg, 0.0207 mmol) and KOAc (40.59 mg, 0.413 mmol). The reaction mixture was stirred at 80 °C for 2 h under N_2. The cooled reaction mixture was concentrated under reduced pressure and the residue was purified by prep-TLC (PE: EtOAc = 0:1) to give the title compound (50 mg, 65.9%) as a yellow oil.

Step 4: Synthesis of tert-butyl (2-(4-(6-((6-amino-2-(difluoromethyl)pyrimidin-4-yl)amino)-4-methoxypyridin-3-yl)-1H-pyrazol-1-yl)ethyl)(methyl)carbamate.

The following compound was synthesized following procedures similar to that described for compound 15, step 4 above using *tert*-butyl (2-(4-(6-chloro-4-methoxypyridin-3-yl)-1*H*-pyrazol-1-yl)ethyl)(methyl)carbamate to afford the title compound which was used as is in the next without further purification.

Step 5: Synthesis of 2-(difluoromethyl)-N^4-(4-methoxy-5-(1-(2-(methylamino)ethyl)-1H-pyrazol-4-yl)pyridin-2-yl)pyrimidine-4,6-diamine.

To a solution of tert-butyl (2-(4-(6-((6-amino-2-(difluoromethyl)pyrimidin-4-yl)amino)-4-methoxypyridin-3-yl)-1*H*-pyrazol-1-yl)ethyl)(methyl)carbamate in DCM (2 mL) was added TFA (1 mL, 13.51 mmol) and the reaction was stirred at room temperature for 1

h. The reaction mixture was concentrated under reduced pressure to give a residue and then adjusted with $NH_3.H_2O$ to pH 5-6 to give the title compound (9.80 mg, 24.6%) as a pale yellow solid. LC-MS m/z = 391 $[M+H]^+$. ^1H NMR (400 MHz, DMSO) δ 9.80 (s, 1H), 8.36 (s, 1H), 8.10 (s, 1H), 7.90 (s, 1H), 7.38 (s, 1H), 6.97 (s, 1H), 6.91 (s, 2H), 6.52 (t, *J* = 54.8 Hz, 1H), 4.19 (t, *J* = 6.3 Hz, 2H), 3.90 (s, 3H), 2.89 (t, *J* = 6.3 Hz, 2H), 2.30 (s, 3H).

Compound 17. Synthesis of 1-(4-(6-((6-amino-2-(difluoromethyl)pyrimidin-4-yl)amino)-4-methoxypyridin-3-yl)-1*H*-pyrazol-1-yl)-2-methylpropan-2-ol.

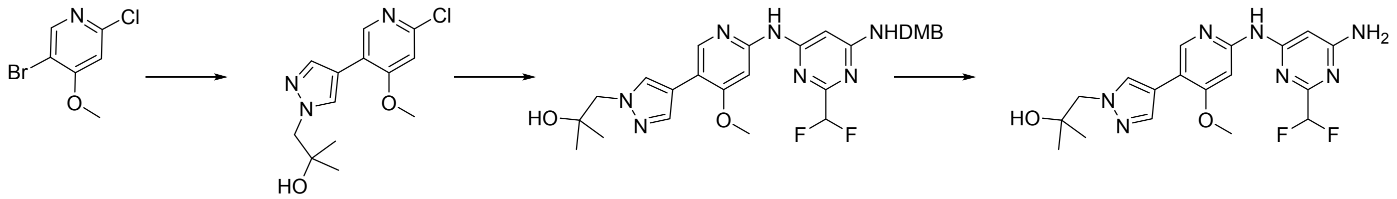

Step 1: Synthesis of 1-(4-(6-chloro-4-methoxypyridin-3-yl)-1H-pyrazol-1-yl)-2-methylpropan-2-ol.

A mixture of 2-chloro-5-iodo-4-methoxypyridine (Step 1 of compound 16), 2-methyl-1-(4-(4,4,5,5-tetramethyl-1,3,2-dioxaborolan-2-yl)-1*H*-pyrazol-1-yl)propan-2-ol (891 mg, 3.35 mmol), $Pd(dppf)Cl_2$ (204 mg, 0.279 mmol), and K_2CO_3 (770 mg, 5.58 mmol) in dioxane (12 mL) and H_2O (3 mL) was stirred at 90 °C for 4 h under N_2. The cooled mixture was concentrated *in vacuo* to give the crude product which was purified by silica gel chromatography (PE: EtOAc =1:2) to give the title compound (560 mg, 71.5%) as a yellow oil. LC-MS m/z = 282 [M+1].

Step 2: Synthesis of 1-(4-(6-((2-(difluoromethyl)-6-((2,4-dimethoxybenzyl)amino) pyrimidin-4-yl)amino)-4-methoxypyridin-3-yl)-1H-pyrazol-1-yl)-2-methylpropan-2-ol.

A mixture of 1-(4-(6-chloro-4-methoxypyridin-3-yl)-1*H*-pyrazol-1-yl)-2-methylpropan-2-ol (560 mg, 1.99 mmol), 2-(difluoromethyl)-N^4-(2,4-dimethoxybenzyl)pyrimidine-4,6-diamine (679 mg, 2.19 mmol,**SI-3**), Pd(t-$Bu_3P)_2$ (200 mg, 0.392 mmol), and Cs_2CO_3 (1.29 g, 3.98 mmol) in dioxane (12 mL) was stirred at 100 °C for 16 h under N_2. The mixture was poured into water (80 mL) and extracted with EtOAc (100 mL x 3). The combined organic layers were concentrated *in vacuo* and the crude product was purified by silica gel chromatography (PE: EtOAc =1:10) to give the title compound (480 mg, 43.6%) as a yellow oil. LC-MS m/z = 556 [M+1].

Step 3: Synthesis of 1-(4-(6-((6-amino-2-(difluoromethyl)pyrimidin-4-yl)amino)-4-methoxypyridin-3-yl)-1H-pyrazol-1-yl)-2-methylpropan-2-ol.

A solution of 1-(4-(6-((2-(difluoromethyl)-6-((2,4-dimethoxybenzyl)amino)pyrimidin-4-yl)amino)-4-methoxypyridin-3-yl)-1*H*-pyrazol-1-yl)-2-methylpropan-2-ol (480 mg, 0.86 mmol) in TFA (2 mL) and DCM (2 mL) was stirred at room temperature for 2 h. The mixture was concentrated *in vacuo* and a saturated aqueous $NaHCO_3$ solution (20 mL) was added. The precipitate was filtered and the solid was dissolved in MeOH (30 mL). The precipitate was filtered, and the filtrate was concentrated to give the crude product which was washed with DCM. The precipitate was filtered to provide the title compound (294 mg, 84%) as a light yellow solid. LC-MS m/z = 406 [M+1]. ^{1}HNMR (500 MHz, DMSO-d_6) δ 10.56 (s, 1H), 8.46 (s, 1H), 8.12 (s, 1H), 7.93 (s, 1H), 7.22 (s, 2H), 6.82 – 6.47 (m, 2H), 4.06 (s, 2H), 4.00 (s, 3H), 1.09 (s, 6H).

Compound 18. Synthesis of (S)-1-(4-(6-((6-amino-2-(1-fluoroethyl)pyrimidin-4-yl)amino)-4-isopropoxypyridin-3-yl)-1*H*-pyrazol-1-yl)-2-methylpropan-2-ol

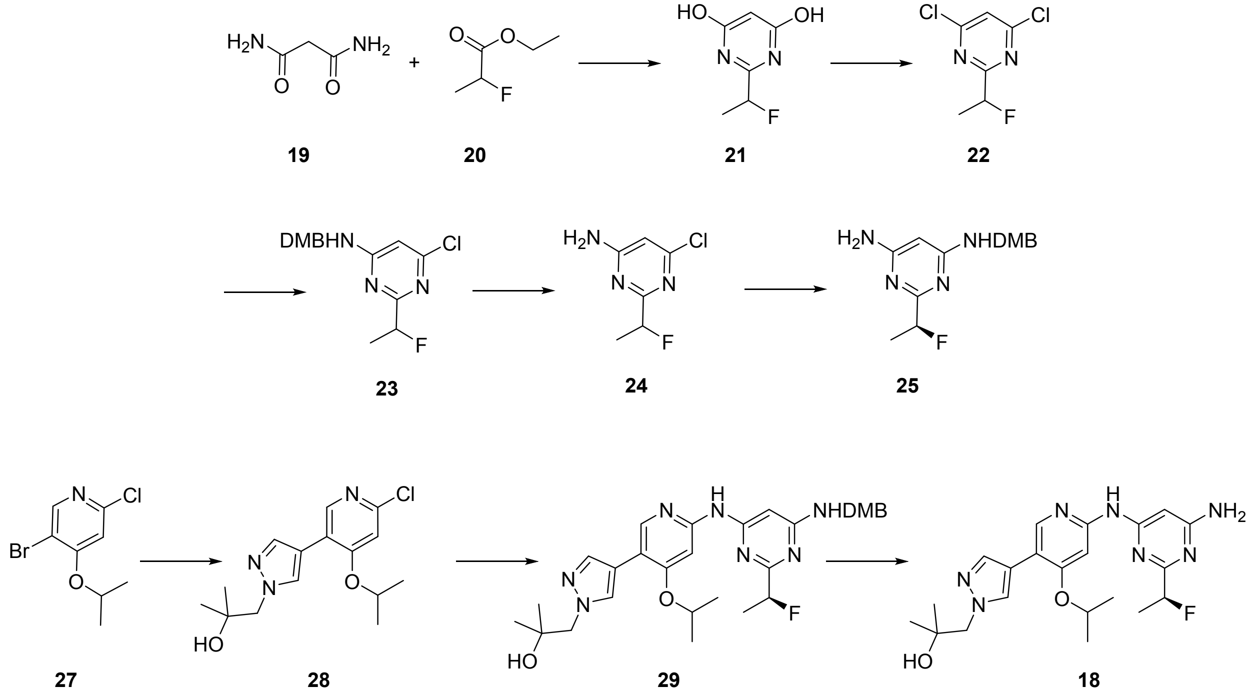

Step 1. Synthesis of 2-(1-fluoroethyl)pyrimidine-4,6-diol (21).

To a solution of malonamide (30 g, 294 mmol) in EtOH (300 mL) was added EtONa (40.0 g, 588 mmol) and the solution stirred at 20 °C for 1 h. Ethyl 2-fluoropropanoate (38.7 g, 323 mmol) was added dropwise and the reaction stirred at 100 °C for 2 h. The pH of the mixture was adjusted to pH = 6 with 1 N HCl, and then concentrated *in vacuo*. The residue was adjusted to pH ~ 2 with 1 N HCl and filtered to provide the title compound (31 g, 66% yield) as a yellow solid, which was used in the next step without further purification. LC-MS m/z = 159 [M+1].

Step 2: Synthesis of 4,6-dichloro-2-(1-fluoroethyl)pyrimidine (22).

To a stirred solution of 2-(1-fluoroethyl)pyrimidine-4,6-diol (31 g, 196 mmol) in toluene (300 mL) was added $POCl_3$ (72.8 mL, 784 mmol) at room temperature. Triethylamine

(54.4 mL, 392 mmol) was added dropwise and the reaction stirred at 100 °C for 2 h. The reaction mixture was poured into warm water and extracted with EtOAc (x 3). The combined organic layers were washed with brine, dried over Na_2SO_4, filtered, and the filtrate was evaporated under reduced pressure to give the title compound (40 gcrude) directly used in the next step without further purification.

Step 3: Synthesis of 6-chloro-N-(2,4-dimethoxybenzyl)-2-(1-fluoroethyl)pyrimidin-4-amine (23).

A solution of 4,6-dichloro-2-(1-fluoroethyl)pyrimidine (40 g, 196 mmol), 2,4-dimethoxybenzylamine (32.7 g, 196 mmol), and DIPEA (50.5 g, 392 mmol) in NMP (150 mL) was stirred at 100 °C for 1 h. The mixture was poured into water (500 mL) and extracted with EtOAc (300 mL x 3). The combined organics were washed with brine (300 mL x 3), dried over Na_2SO_4, and evaporated under reduced pressure to give the title compound (22 g, crude) as a yellow solid, which was used in the next step directly. LC-MS m/z = 326 [M+1].

Step 4: Synthesis of 6-chloro-2-(1-fluoroethyl)pyrimidin-4-amine (24).

A solution of 6-chloro-*N*-(2,4-dimethoxybenzyl)-2-(1-fluoroethyl)pyrimidin-4-amine (22 g, 67.6 mmol) in HCl/EtOAc (4.0 M, 85 mL) was stirred at room temperature for 12 h. The mixture was concentrated *in vacuo*, the pH of the residue adjusted to pH ~ 8 with saturated $NaHCO_3$ (aq.) solution, and the mixture extracted with DCM (300 mL x 3). The combined organic layers were washed with brine (300 mL), dried over Na_2SO_4, and concentrated *in vacuo*. The residue was triturated with PE:EtOAc (1:1) and filtered to give the title compound (10 g, 84% yield over 3 steps) as a gray solid. LC-MS m/z = 176 [M+1].

Step 5: Synthesis of (S)-N⁴-(2,4-dimethoxybenzyl)-2-(1-fluoroethyl) pyrimidine-4,6-diamine (25) and (R)-N⁴-(2,4-dimethoxybenzyl)-2-(1-fluoroethyl)pyrimidine-4,6-diamine.

A solution of 6-chloro-2-(1-fluoroethyl)pyrimidin-4-amine (10.0 g, 57 mmol), 2,4-dimethoxybenzylamine (9.5 g, 57 mmol), and DIPEA (29.3 g, 228 mmol) in NMP (60 mL) was stirred at 140 °C for 2 h. The cooled mixture was poured into water (500 mL), extracted with EtOAc (300 mL x 3), and the combined organic layers were washed with brine (300 mL x 2), dried over Na_2SO_4, and concentrated. The residue was purified by silica gel chromatography to provide the racemic mixture (6.6 g, 37% yield) as a yellow solid. This product was further purified by SFC, using an OZ 20 x 250 mm, 10 µm (Daicel) column, eluting with 35% MeOH (0.2% MeOH/NH_3) at 100 g/min, to obtain the first eluting enantiomer: (*S*)-*N*^{4}-(2,4-dimethoxybenzyl)-2-(1-fluoroethyl)pyrimidine-4,6-diamine (2.1 g, **25**) as a yellow solid. LC-MS m/z = 307 [M+1]. Further elution provided the second enantiomer, (*R*)-*N*^{4}-(2,4-dimethoxybenzyl)-2-(1-fluoroethyl)pyrimidine-4,6-diamine.

Step 6: Synthesis of 1-(4-(6-chloro-4-isopropoxypyridin-3-yl)-1H-pyrazol-1-yl)-2-methylpropan-2-ol (28).

A mixture of 5-bromo-2-chloro-4-isopropoxypyridine (2.0 g, 8.06 mmol, **27**), 2-methyl-1-(4-(4,4,5,5-tetramethyl-1,3,2-dioxaborolan-2-yl)-1*H*-pyrazol-1-yl)propan-2-ol (2.15 g, 8.06 mmol), K_2CO_3 (3.34 g, 24.2 mmol), and Pd(dppf)Cl_2 (592 mg, 0.81 mmol) in dioxane/water (20 mL/ 4 mL) was stirred at 80 °C for 3 h. The cooled mixture was concentrated *in vacuo* and the residue was purified by column chromatography on silica gel (PE/EtOAc = 10/1 to 2/1) to afford the title compound (2.2 g, 88% yield) as a yellow solid. LC-MS m/z = 310 [M+1].

Step 7: Synthesis of (S)-1-(4-(6-((6-((2,4-dimethoxybenzyl)amino)-2-(1-fluoroethyl)pyrimidin-4-yl)amino)-4-isopropoxypyridin-3-yl)-1H-pyrazol-1-yl)-2-methylpropan-2-ol (29).

A mixture of 1-(4-(6-chloro-4-isopropoxypyridin-3-yl)-1*H*-pyrazol-1-yl)-2-methylpropan-2-ol (800 mg, 2.59 mmol, **28**), (*S*)-N^4-(2,4-dimethoxybenzyl)-2-(1-fluoroethyl)pyrimidine-4,6-diamine (800 mg, 2.59 mmol, **25**), BrettPhos Pd G4 (397 mg, 0.26 mmol), and Cs_2CO_3 (2.54 g, 7.77 mmol) in dioxane (20 mL) was stirred at 100 °C for 16 h. The cooled mixture was concentrated *in vacuo* and the residue was purified by column chromatography on silica gel (EtOAc/MeOH = 10/1 to 3/1) to afford the title compound (840 mg, 56% yield) as a yellow solid. LC-MS m/z = 580 [M+1].

Step 8: Synthesis of (S)-1-(4-(6-((6-amino-2-(1-fluoroethyl)pyrimidin-4-yl)amino)-4-isopropoxypyridin-3-yl)-1H-pyrazol-1-yl)-2-methylpropan-2-ol (18).

To a solution of (*S*)-1-(4-(6-((6-((2,4-dimethoxybenzyl)amino)-2-(1-fluoroethyl)pyrimidin-4-yl)amino)-4-isopropoxypyridin-3-yl)-1*H*-pyrazol-1-yl)-2-methylpropan-2-ol (840 mg, 1.45 mmol, **29**) in DCM (5 mL) was added TFA (10 mL) and the reaction stirred at room temperature for 4 h. The mixture was concentrated *in vacuo* and the residue was purified by prep HPLC-B to afford the title compound (421.1 mg, 67% yield) as a white solid. LC-MS m/z= 430 [M+1]; ^{1}H NMR (500 MHz, DMSO) δ 9.55 (s, 1H), 8.35 (s, 1H), 8.06 (d, *J* = 0.8 Hz, 1H), 7.89 (d, *J* = 0.7 Hz, 1H), 7.56 (s, 1H), 6.69 (s, 1H), 6.63 (s, 2H), 5.31 (dq, *J* = 48.5, 6.5 Hz, 1H), 4.76 – 4.66 (m, 2H), 4.05 (s, 2H), 1.58 (dd, *J* = 24.2, 6.5 Hz, 3H), 1.41 (dd, *J* = 6.0, 1.5 Hz, 6H), 1.09 (s, 6H).

4. Purity Analysis for Compound 18

Mol. Formula	$C_{21}H_{28}FN_7O_2$
Structure Name	
Mol. Wt.	429.50
Net Wt.	61.36 g

Analytical Tests and Results

Analytical Tests	Results
Description	white solid
^{1}H-NMR (400 MHz, DMSO)	Consistent with the structure
ESI-LCMS	m/z = 430 $[M+H]^+$
LCMS	RT=6.729 min, 100%(214nm)

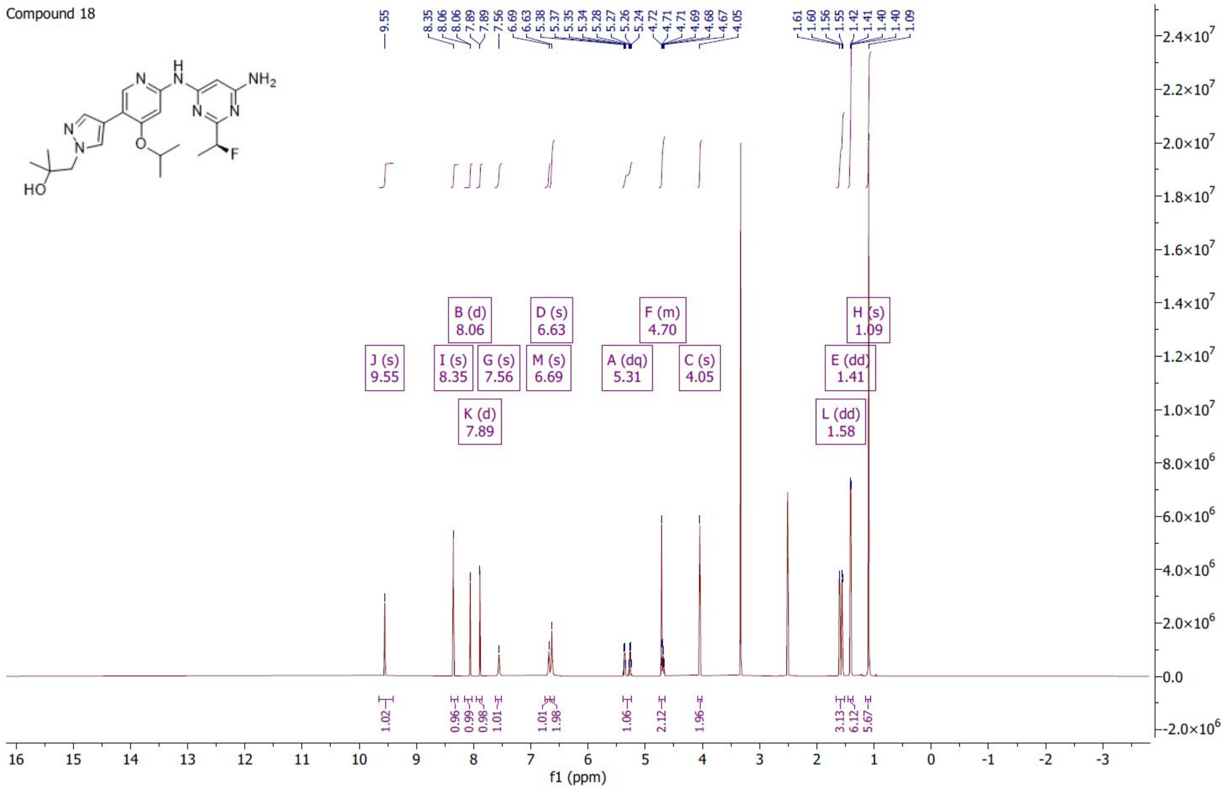

^{1}H NMR (500 MHz, DMSO) δ 9.55 (s, 1H), 8.35 (s, 1H), 8.06 (d, J = 0.8 Hz, 1H), 7.89 (d, J = 0.7 Hz, 1H), 7.56 (s, 1H), 6.69 (s, 1H), 6.63 (s, 2H), 5.31 (dq, J = 48.5, 6.5 Hz, 1H), 4.76 – 4.66 (m, 2H), 4.05 (s, 2H), 1.58 (dd, J = 24.2, 6.5 Hz, 3H), 1.41 (dd, J = 6.0, 1.5 Hz, 6H), 1.09 (s, 6H).

LC/MS Report

```
File D:\DATA\C59\MONITOR\N-429-P-82928-LCMSA039.D
Injection Date    :  8 Dec 21   7:59 am +0800       Tgt Mass(EZX) : 429.00
Sample Name       : N-429-P                              Location : P1-C-02
Acq. Operator     : C59-Monitor                               Inj : 1
Spec. Reported    : MS Integration                     Inj Volume : 2 ul
Acq. Method       : D:\METHODS\1-POS-15MIN.M
Analysis Method   : D:\METHODS\1-POS-15MIN.M
Sample Info : Easy-Access Method: '1-POS-15MIN'  429.00
Method Info : Mobile Phase: A:Water(10mM NH4HCO3)   B:ACN
              Gradient: 5% B increase to 95%B within 8min, 95%B for 7min.
              Flow Rate: 1.8ml/min
              Column: XBridge C18,4.6*150mm,3.5um
              Column Temperature: 45 C
              Detection:UV(214,4nm) and MS(ESI,Pos mode,110 to 1500 amu)
```

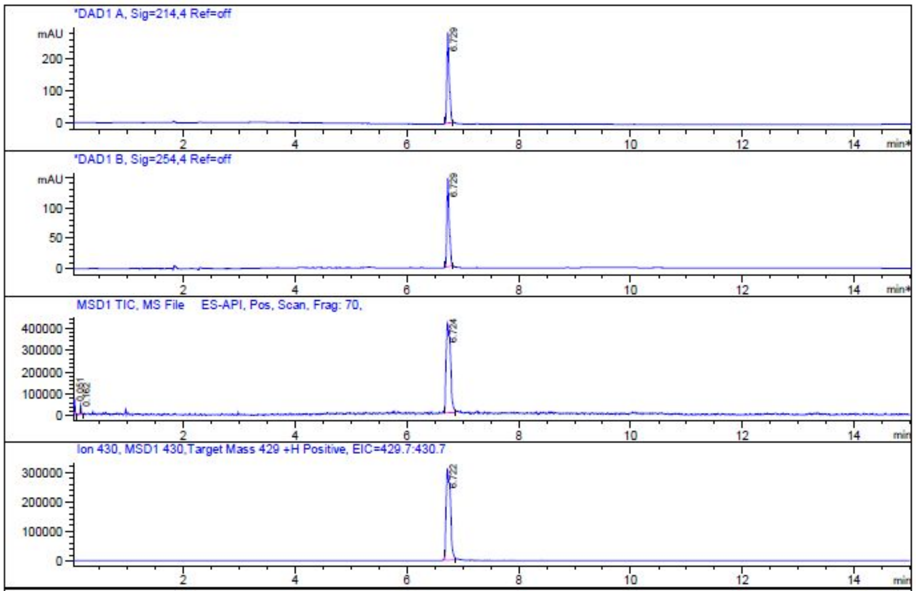

Integration Results for DAD1 A, Sig=214,4 Ref=off

RetTim	Width	Area	Height	Area%
6.73	0.04	841.78	285.61	100.00

Integration Results for DAD1 B, Sig=254,4 Ref=off

RetTim	Width	Area	Height	Area%
6.73	0.04	430.81	146.95	100.00

LC/MS Report

Ret. Time: 0.05 <<<< POSITIVE SPECTRA >>>>

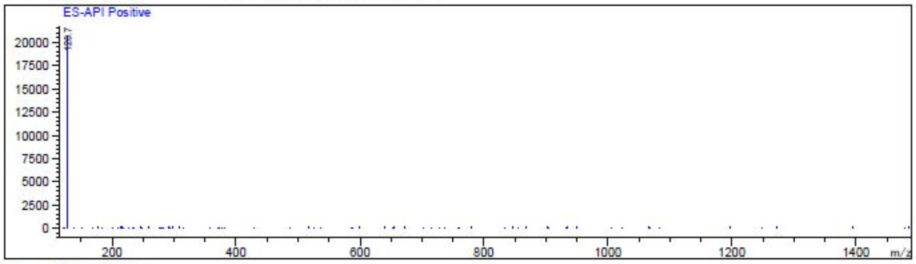

Ret. Time: 0.16

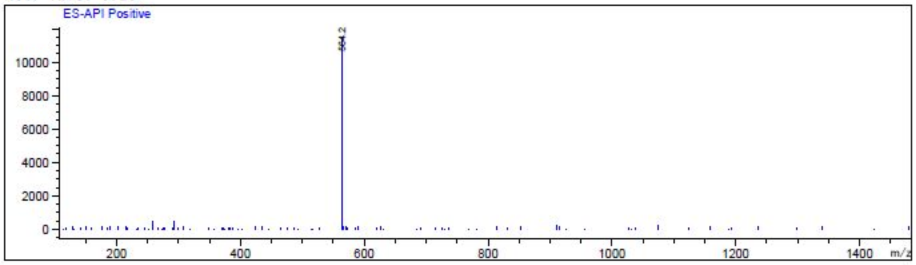

Ret. Time: 6.72

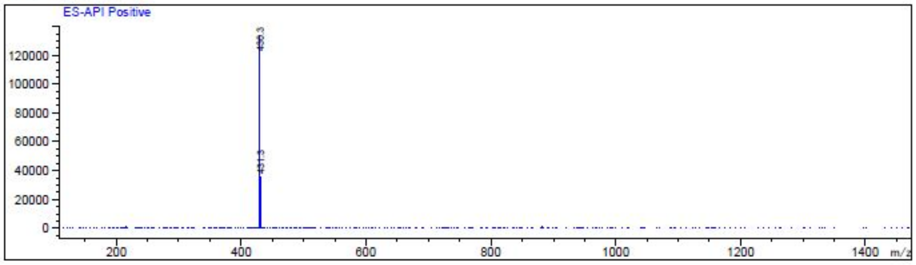

5. Kinome data for compound 18

Human, wild type kinases in the DiscoverX panel with binding %Cntrl < 7 in the primary screen were chosen for follow up Kd determinations. Kinases with an Alanine at the equivalent position to 654 in KIT are highlighted in the table.

**Kinase**	**Compound 18**
S(333nM)	0.060
KIT(V559D,V654A)	0.4
**TYK2(JH2)**	**5.5**
**MINK**	**8.7**
BUB1	20
PDGFRA	15.5
KIT	55.5
FLT3	68.5
PDGFRB	74
BLK	14.5
LCK	37
**BMPR2**	**85**
**TYRO3**	**88.5**
YES	119
FGR	125
HCK	170
PIKFYVE	190
SRC	240
PIK4CB	
MEK5	265

6. Crystallography

X-ray data collection and refinement statistics for compounds 1 and 11

A KIT protein construct encompassing cytoplasmic domain residues 544-935 with kinase insert domain residues 694-753 deleted and replaced with a Thr/Ser insert (1) was further modified by mutagenesis to introduce the V654A mutation. Protein expression and purification followed previously described protocols (1). Crystals of compound **1** were grown via sitting-drop vapor diffusion starting with 4.7 mg/ml V654A mutant protein complexed with a 5-fold molar excess of inhibitor, mixed 1:1 with and equilibrated over a reservoir of 8 % PEG 4000, 0.1 M Na-citrate, pH 5.0, 10% 2-propanol. Resultant crystals were cryo-protected by addition of 25% MPD (final), frozen, and a 1.54A data set collected at the European synchrotron radiation source (Grenoble, FR, beamline ID23.1).

Crystals of compound **11** were grown via sitting-drop vapor diffusion starting with 9.9 mg/ml V654A mutant protein complexed with a 5-fold molar excess of inhibitor, mixed 1:1 with and equilibrated over a reservoir of 8 % PEG 4000, 0.1 M Hepes/NaOH, pH 8.2. Resultant crystals were cryo-protected by addition of 30% ethylene glycol (final), frozen, and a 2.1A data set collected at the Diamond Light Source (Didcot, UK, beamline i04).

Structures were determined by molecular replacement using the program PHASER (2) and pdb entry 3G0E as the search model. Structure refinement was carried out using REFMAC5 (3). Crystal structures have been deposited in the RCSB PDB with accession codes 9Z1L and 9Z2S, and coordinates will be released upon publication.

(1) Mol, C.D.; Lim, K.B.; Sridhar, V.; Zou, H.; Chien, E.Y.T; Sang, Bi-Ching; Nowakowski, J.; Kassel, D.B.; Cronin, C.N.; McRee, D.E. Structure of a KIT Product Complex Reveals the Basis for Kinase Transactivation. *J Biol Chem.* **2003**, *278*, 31461.

(2) McCoy, A.J.; Grosse-Kunstleve, R.W.; Adams, P.D.; Winn, M.D.; Storoni, L.C.; Read, R.J. Phaser Crystallographic Software. *J. Appl. Crys* **2007**, *40*, 658.

(3) Murshudov, G.N.; Skubak, P.; Lebedev, A.A.; Pannu, N.S.; Steiner, A.; Nicholls, R.A.; Winn, M.D.; Long, F.; Vagin, A.A. REFMAC5 for the Refinement of Macromolecular Crystal Structures. *Acta Crystallogr* **2011**, *D67*, 355.

	**Compound 1**	**Compound 11**
**Accession Code**	**9Z1L**	**9Z2S**
**Data collection:**		
**Wavelength**	0.886	0.980
**Resolution range (Å)**	54.25 – 1.54 (1.75 – 1.54)	29.06 – 2.10 (2.16 – 2.10)
**Space group**	$P4_32_12$	$P2_12_12_1$
**Unit cell**	56.3 56.3 202.7	47.7 79.9 92.2
	90.0 90.0 90.0	90.0 90.0 90.0
**Unique reflections**	27422 (1371)	20964 (1729)
**Multiplicity**	1.7 (1.7)	4.9 (4.7)
**Completeness (%)**	55.7 (8.9)	90.0 (99.8)
**Mean I/sigma(I)**	12.1 (1.7)	12.1 (2.0)
**R-meas**	0.280 (2.60)	0.070 (0730)
**Refinement:**		
**Resolution range (Å)**	54.25 – 1.54 (1.58 – 1.54)	29.08 – 2.10 (2.15 – 2.10)
**Reflections used in refinement**	26027 (35)	19898 (1443)
**Reflections used for R-free**	1395 (0)	1035 (88)
**Completeness (%)**	55.7 (1.0)	98.6 (99.6)
**R-work**	0.165 (0.220)	0.167 (0.264)
**R-free**	0.206 (0.000)	0.236 (0.267)
**Number of non-hydrogen atoms**	3104	3065
**r.m.s.d. bonds (Å)**	0.003	0.003

**r.m.s.d. angles (°)**	0.985	1.190

X-ray of compound 25 to confirm stereochemistry

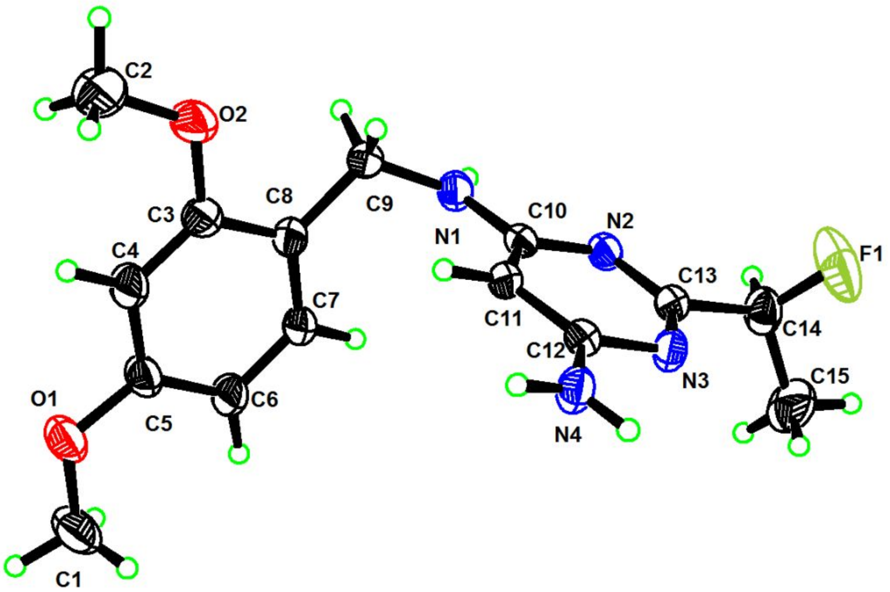

References

Dolomanov, O. V., Bourhis, L. J., Gildea, R. J., Howard, J. A. K. & Puschmann, H. (2009). *J. Appl. Cryst.* **42**, 339–341.

Sheldrick, G. M. (2015). *Acta Cryst.* C**71**, 3–8.

(220201127aa1_0m)

Crystal data

$4(C_{15}H_{19}FN_4O_2)\cdot C_4H_8O_2$	$Z = 1$
$M_r = 1313.47$	$F(000) = 696$
Triclinic, $P1$	$D_x = 1.246$ Mg m^{-3}
$a = 10.3814$ (2) Å	Cu $K\alpha$ radiation, $\lambda = 1.54178$ Å
$b = 11.5516$ (2) Å	Cell parameters from 9952 reflections

c = 15.9308 (3) Å	θ = 2.8–74.4°
α = 93.795 (1)°	μ = 0.78 mm^{-1}
β = 91.138 (1)°	T = 170 K
γ = 113.125 (1)°	Block, colourless
V = 1750.90 (6) Å^3	0.08 × 0.05 × 0.04 mm

Data collection

D8 VENTURE diffractometer	10057 reflections with $I > 2\sigma(I)$
ϕ and ω scans	R_{int} = 0.047
Absorption correction: multi-scan *SADABS2016*/2 (Bruker,2016/2) was used for absorption correction. wR2(int) was 0.1676 before and 0.0603 after correction. The Ratio of minimum to maximum transmission is 0.8774. The λ/2 correction factor is Not present.	θ_{max} = 74.5°, θ_{min} = 2.8°
T_{min} = 0.661, T_{max} = 0.754	h = -12→12
33945 measured reflections	k = -14→14
12373 independent reflections	l = -19→19

Refinement

Refinement on F^2	Hydrogen site location: mixed
Least-squares matrix: full	H-atom parameters constrained
$R[F^2 > 2\sigma(F^2)]$ = 0.063	$w = 1/[\sigma^2(F_o^2) + (0.1099P)^2 + 0.5849P]$ where $P = (F_o^2 + 2F_c^2)/3$
$wR(F^2)$ = 0.198	$(\Delta/\sigma)_{max}$ = 0.005
S = 1.09	$\Delta\rangle_{max}$ = 0.41 e Å^{-3}
12373 reflections	$\Delta\rangle_{min}$ = -0.44 e Å^{-3}
881 parameters	Absolute structure: Flack x determined using 3292 quotients [(I+)-(I-)]/[(I+)+(I-)] (Parsons, Flack and Wagner, Acta Cryst. B69 (2013) 249-259).
29 restraints	Absolute structure parameter: 0.14 (10)

Special details: Geometry. All esds (except the esd in the dihedral angle between two l.s. planes) are estimated using the full covariance matrix. The cell esds are taken into account individually in the

estimation of esds in distances, angles and torsion angles; correlations between esds in cell parameters are only used when they are defined by crystal symmetry. An approximate (isotropic) treatment of cell esds is used for estimating esds involving l.s. planes.

7. Biology

Inhibition of KIT Autophosphorylation in HMC1.1 11/13 Cells by MSD assay

The goal of this assay was to assess the percent inhibition of autophosphorylation of KIT in HMC1.1 cell (harboring V654A exon 13 mutation) when treated with compounds in a dose-dependent manner.

HMC1.1 KIT V654A (or HMC1.1 11/13) cells were maintained in IMDM medium supplemented with 20% Calf Serum with Iron and 100 units/mL Penicillin-Streptomycin and grown in a 37°C humidified tissue culture incubator.

Seeding was performed as follows:

The cells were first washed with phenol red-free, serum-free IMDM, then seeded in the same medium at 50,000 cells per well in 50µL volume in a U-bottom 96 well plate. The plate was covered and incubated at 37°C in a humidified tissue culture incubator for 4 hours.

Compound dosing was performed as follows:

1. Add the compound from prediluted source plate to the cell plate, The final layout is 8-point, 4-fold serial titration of compounds covering the desired dose range.
2. Cover and incubate cells at 37°C in a humidified tissue culture incubator for 90 minutes. Pellet cells at 4°C and remove media.
3. Lysate the cells by adding 1X AlphaLISA Lysis Buffer supplemented with protease inhibitor cocktail. Cover and shake cells at 4°C on a plate shaker at 600rpm for 30 minutes. The lysate can be used fresh or stored in -20°C freezer until use.
4. Spin the plate at 4°C before proceeding to the MSD step.

The MSD was performed according to manufacturer's protocol and as follows:

1. The MSD kit used for this assay is Phospho KIT (Tyr721) Assay Whole Cell Lysate Kit (MSD: 515DPD). Reagents including blocking buffer, wash buffer,

antibody solution and stop buffer were prepared according to manufacturer's protocol instruction.

2. All incubation steps were done by covering plate and incubating it at room temperature on a plate shaker at 700rpm for one hour. And all wash steps were done three times with 150µL Tris wash buffer per well.
3. Add 150µL per well of blocking buffer to the MSD plate, incubate and followed by washing three times
4. Transfer 25µL sample of lysate to the MSD plate, incubate and followed by washing three times.
5. Add 25µL detection antibody solution to the MSD plate, incubate and followed by washing three times.
6. Add 150µL per well of read buffer. Read plate on MSD Sector Imager within 5 minutes of adding read buffer.

Inhibition of KIT Autophosphorylation in M-07e Cells by MSD assay

The goal of this assay was to assess the percent inhibition of autophosphorylation of KIT in M-07e cells (expressing wild type KIT) when treated with compounds in a dose-dependent manner.

M-07e cells were maintained in IMDM media supplemented with 10% FBS, 5 ng/ml GM-CSF and 100 units/mL Penicillin-Streptomycin and grown in a 37°C humidified tissue culture incubator.

<u>Seeding was performed as follows:</u>
The cells were first washed with phenol red-free, serum-free IMDM, then seeded in the same medium at 100,000 cells per well in 50µL volume in a U-bottom 96 well plate. The plate was covered and incubated at 37°C in a humidified tissue culture incubator for 4 hours.

<u>Compound dosing was performed as follows:</u>

1. Add the compound from prediluted source plate to the cell plate, The final layout is 8-point, 4-fold serial titration of compounds covering the desired dose range.
2. Add SCF solution to each well at the final concentration of 50 ng/mL final concentration. Cover the plate and shake at room temperature on a plate shaker at 450 rpm for 10 minutes.
3. Lysate the cells by adding AlphaLISA Lysis Buffer supplemented with protease inhibitor cocktail. Cover and shake cells at 4°C on a plate shaker at 600 rpm for 30 minutes. The lysate can be used fresh or stored in -20°C freezer until use.
4. Spin the plate at 4°C before proceeding to the MSD step.

<u>The MSD was performed according to manufacturer's protocol and as follows:</u>

1. The MSD kit used for this assay is Phospho KIT (Tyr721) Assay Whole Cell Lysate Kit (MSD: 515DPD). Reagents including blocking buffer, wash buffer, antibody solution and stop buffer were prepared according to manufacturer's protocol instruction.
2. All incubation steps were done by covering plate and incubating it at room temperature on a plate shaker at 700rpm for one hour. And all wash steps were done three times with 150μL Tris wash buffer per well.
3. Add 150μL per well of blocking buffer to the MSD plate, incubate and followed by washing three times
4. Transfer 25μL sample of lysate to the MSD plate, incubate and followed by washing three times.
5. Add 25μL detection antibody solution to the MSD plate, incubate and followed by washing three times.
6. Add 150μL per well of read buffer. Read plate on MSD Sector Imager within 5 minutes of adding read buffer.

Inhibition of PDGFRβ Autophosphorylation in SW579 Cells by HTRF

The goal of this assay was to assess the percent inhibition of autophosphorylation of PDGFRβ in SW579 cells (expressing wild type PDGFR) when treated with compounds in a dose-dependent manner.

SW579 cells were maintained in DMEM media supplemented with 10% FBS and grown in a 37°C humidified tissue culture incubator.

<u>Seeding was performed as follows:</u>

The cells were first washed with phenol red-free, serum-free DMEM, then seeded in the same medium at 30,000 cells per well in 15μL volume in 384 well plate. The plate was covered and incubated at 37°C in a humidified tissue culture incubator for 3 hours.

<u>Compound dosing was performed as follows:</u>

1. Transfer compounds from prediluted source plate to the cell plate. The final layout is 10-point, 3-fold serial titration of compounds covering the desired dose range.
2. Add hPDGFBB to a final concentration of 100ng/ml, and incubate for 15 minutes at 37°C
3. Add Lysis Buffer supplemented with protease inhibitor. Cover the plate and shake at room temperature on a plate shaker at 600 rpm for 20 minutes.

<u>The HTRF was performed according to manufacturer's protocol and as follows:</u>

1. The HTRF kit used for this assay is HTRF Human and Mouse phospho-PDGFRβ (PDGF Receptor Beta) (Tyr751) Detection Kit (revvity 64PDGPEH).
2. Prepare antibody mix and add 2.5uL antibody mix to a ProxiPlate
3. Transfer 10uL lysate to the plate. Cover the plate with foil seal, and incubate overnight at room temperature
4. Read luminescence on EnVision: channel 1 at 665nM and channel 2 at 620nM. Readout is channel 1 divided by channel 2 times 10000.

8. Quality control of tested compounds and molecular formula strings

GCV%: **Geometric Coefficient of Variation** expressed as a percentage

GSD: Geometric Standard Deviation; GSD of 1.5 means 65% of the data will fall between 1.5-fold of Geomean of each side, and 95% in the range of 1.5^2-fold (2.25-fold)

GSD=EXP(SQRT(LN((GCV/100)^2 + 1)))

Compound	Structure
1	n1(ncc(c1)-c2cnc(cc2OC(C)C)Nc3nc(ncc3)NC)C
2	Clc1nc(nc(c1)Nc2ncc(c(c2)OC(C)C)-c3cn(nc3)C)NC
3	Fc1nc(nc(c1)Nc2ncc(c(c2)OC(C)C)-c3cn(nc3)C)NC
4	n1(ncc(c1)-c2cnc(cc2OC(C)C)Nc3nc(nc(c3)C)NC)C
5	n1(ncc(c1)-c2cnc(cc2OC(C)C)Nc3nc(nc(c3)OC)NC)C
6	n1(ncc(c1)-c2cnc(cc2OC(C)C)Nc3nc(nc(c3)N)NC)C
7	n1(ncc(c1)-c2cnc(cc2OC(C)C)Nc3nc(nc(c3)N)C)C
8	n1(ncc(c1)-c2cnc(cc2OC(C)C)Nc3nc(nc(c3)N)C4CC4)C
9	N(C)c1nc(cc(n1)N)Nc3ncc2c(ncc(c2c3)C(C)C)OC
10	N(C(C)C)c1c(cnc(c1)Nc2nc(nc(c2)N)NC)C(=O)NC
11	N(C(C)C)c1c(cnc(c1)Nc2nc(nc(c2)N)C3CC3)C(=O)NC
12	FC(F)c1nc(cc(n1)N)Nc2ncc(c(c2)NC(C)C)C(=O)NC
13	FC(F)c1nc(cc(n1)N)Nc2ncc(c(c2)N[C@H](CF)C)C(=O)NC
14	FC(F)c1nc(cc(n1)N)Nc2ncc(c(c2)OC(C)C)-c3cn(nc3)C
15	FC(F)c1nc(cc(n1)N)Nc2ncc(c(c2)OC)-c3cn(nc3)C
16	FC(F)c1nc(cc(n1)N)Nc2ncc(c(c2)OC)-c3cn(nc3)CCNC
17	FC(F)c1nc(cc(n1)N)Nc2ncc(c(c2)OC)-c3cn(nc3)CC(O)(C)C
18	F[C@@H](C)c1nc(cc(n1)N)Nc2ncc(c(c2)OC(C)C)-c3cn(nc3)CC(O)(C)C

	pKIT_V654A HMC1.1 (11/13)				pKIT M07e (WT)				Phos PDGFRB_Y751			
Compound	n	Average in nM	GCV%	Standard Deviation	n	Average in nM	GCV%	Standard Deviation	n	Average in nM	GCV%	Standard Deviation
1	7	7.7	87	2.1	4	63.5	95	2.2	2	154.6	45	1.5
2	8	11.0	40	1.5	3	853.1	47	1.6	1	5357.9	-	-
3	7	3.8	58	1.7	2	52.9	30	1.3	2	119.3	2	1.0
4	4	35.3	25	1.3	2	373.8	22	1.2	2	367.1	29	1.3
5	6	75.4	58	1.7	4	1701.9	48	1.6	1	9837.1	-	-
6	4	5.6	42	1.5	2	358.8	33	1.4	2	365.8	56	1.7
7	2	25.6	57	1.7	2	264.3	29	1.3	-	-	-	-
8	2	14.6	41	1.5	4	232.1	39	1.5	4	546.0	45	1.5
9	4	12.4	12	1.1	2	236.0	32	1.4	3	678.9	1	1.0
10	4	13.0	32	1.4	1	649.8	-	-	4	6004.7	50	1.6
11	6	11.6	17	1.2	4	778.4	23	1.3	4	2447.1	59	1.7
12	3	4.0	30	1.3	2	27.7	69	1.9	-	-	-	-
13	6	3.2	39	1.5	5	65.6	56	1.7	-	-	-	-
14	2	3.1	7	1.1	2	19.4	19	1.2	-	-	-	-
15	2	2.0	7	1.1	4	70.8	33	1.4	-	-	-	-
16	2	8.7	14	1.1	2	1418.1	18	1.2	-	-	-	-
17	14	9.7	47	1.6	9	317.2	70	1.9	-	-	-	-
18	12	5.7	36	1.4	10	82.7	48	1.6	7	1251.4	45	1.5

9. *In Vivo* Pharmacokinetics and Pharmacodynamics Studies

PK/PD and efficacy studies on HMC1.1 11/13 subcutaneous xenograft model in NOD-SCID mice

All the procedures related to animal handling, care, and treatment in the study were performed according to the guidelines approved by the Institutional Animal Care and Use Committee (IACUC) of Blueprint Medicines (protocol number BPMC-2018-001) following the guidance of the Association for Assessment and Accreditation of Laboratory Animal Care (AAALAC).

Animals: NOD SCID female mice (strain code 394, 6 weeks, purchased from Charles River Laboratories) weighing approximately 18-20g were used for the studies.

Tumor inoculation: Each mouse was inoculated subcutaneously at the right flank with 1×10^7 HMC1.1 11/13 cells for tumor development. Dosing was started when the average tumor size reached approximately 150 - 200 mm^3 for tumor efficacy study or 300 – 400 mm^3 for PK/PD study. Treatment was given by oral gavage (PO doses).

Blood collection for PK analysis: Blood samples were collected from all animals at 4, 10, and 24 h post dose. Blood collection was performed by tail vein microsampling or cardiac puncture. Plasma was separated from blood by centrifugation at 6000xg for 10 min. Plasma samples were stored at -80℃ and then sent for PK analysis. Compound concentrations in both plasma and tumor were quantified using a liquid chromatography with tandem mass spectrometry (LC-MS/MS) method.

Tumor collection for PD analysis: Tumor samples were collected at termination and the tumor was split into 2~4 parts which were then snap frozen individually. The frozen tumor samples were stored at -80℃ until PD analysis.

Tumor volume: Tumor volume was calculated using the formula $V = 0.5ab^2$, where a and b are the long and short diameters of the tumor in mm, respectively.

Statistical analysis was performed by using a two-way RM ANOVA analysis followed by Dunnett's multiple comparison test.

10. Spectroscopic data of Compounds 1-18

Compound 1

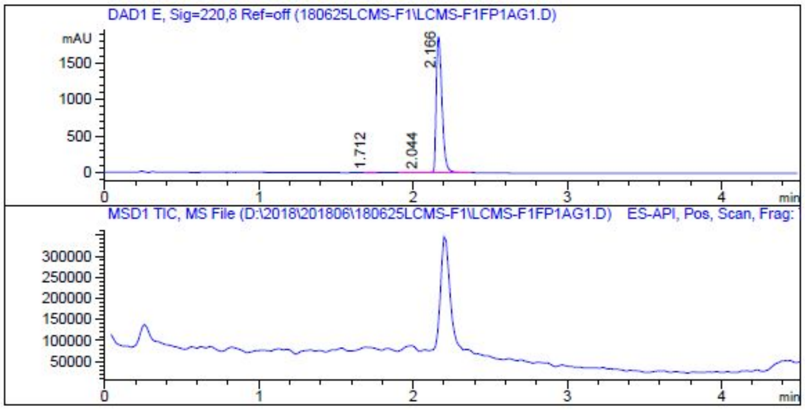

Report

Signal 1 : DAD1 E, Sig=220,8 Ref=off

Peak #	RT [min]	Height	Height %	Width [min]	Area	Area %
1	1.712	2.487	0.133	0.034	5.7160	0.116
2	2.044	6.354	0.340	0.084	40.4580	0.820
3	2.166	1861.009	99.527	0.041	4885.8096	99.064

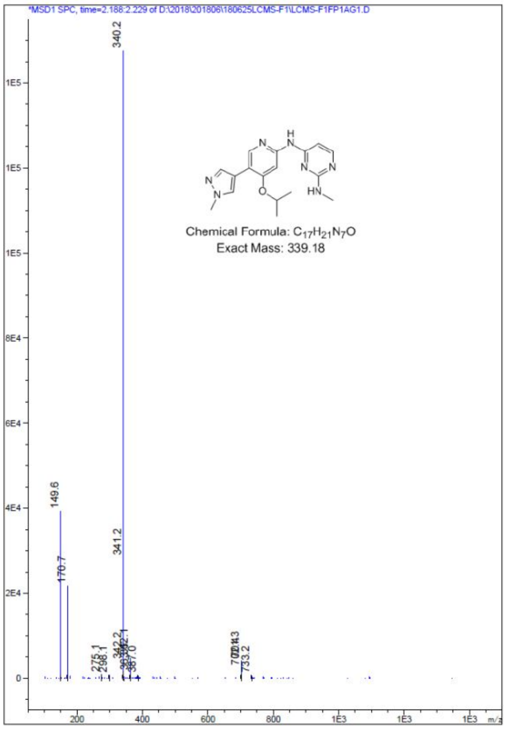

Compound 2

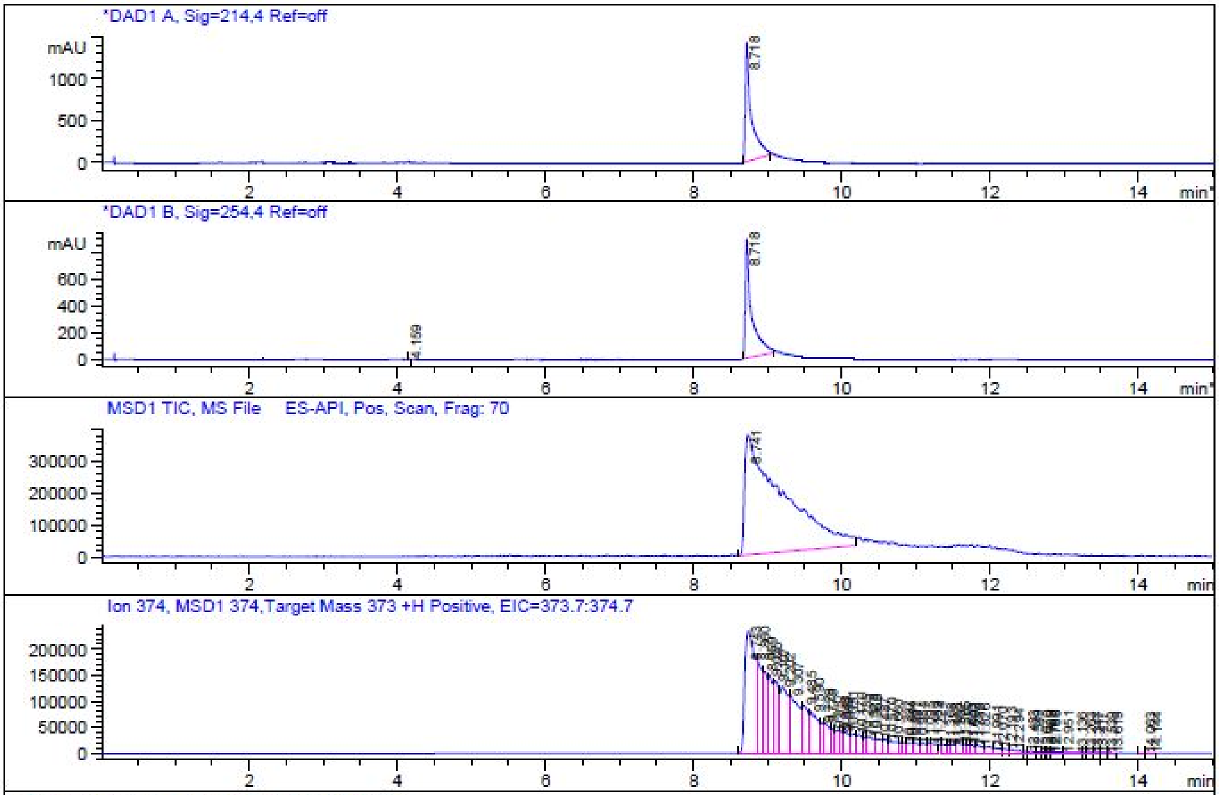

Integration Results for DAD1 A, Sig=214,4 Ref=off

RetTim	Width	Area	Height	Area%
8.72	0.08	8166.11	1428.17	100.00

Integration Results for DAD1 B, Sig=254,4 Ref=off

RetTim	Width	Area	Height	Area%
4.16	0.01	4.09	7.85	0.08
8.72	0.08	5358.80	894.60	99.92

Ret. Time: 8.74 <<<< POSITIVE SPECTRA >>>>

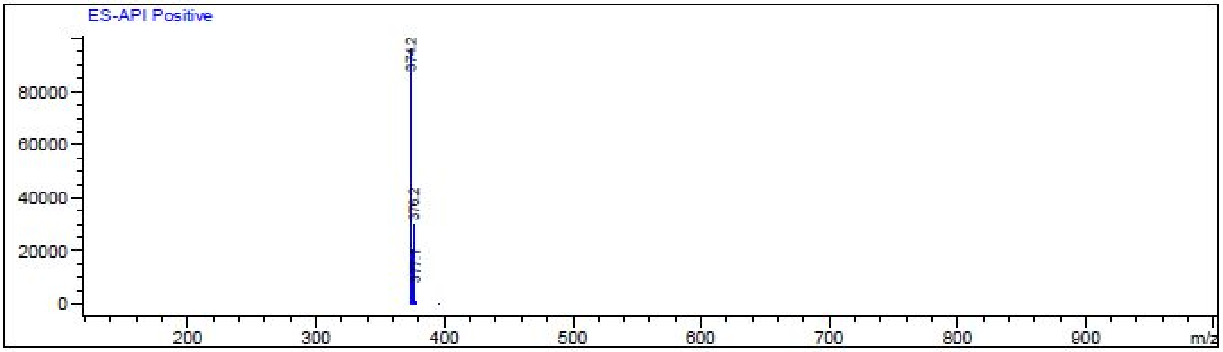

Compound 3

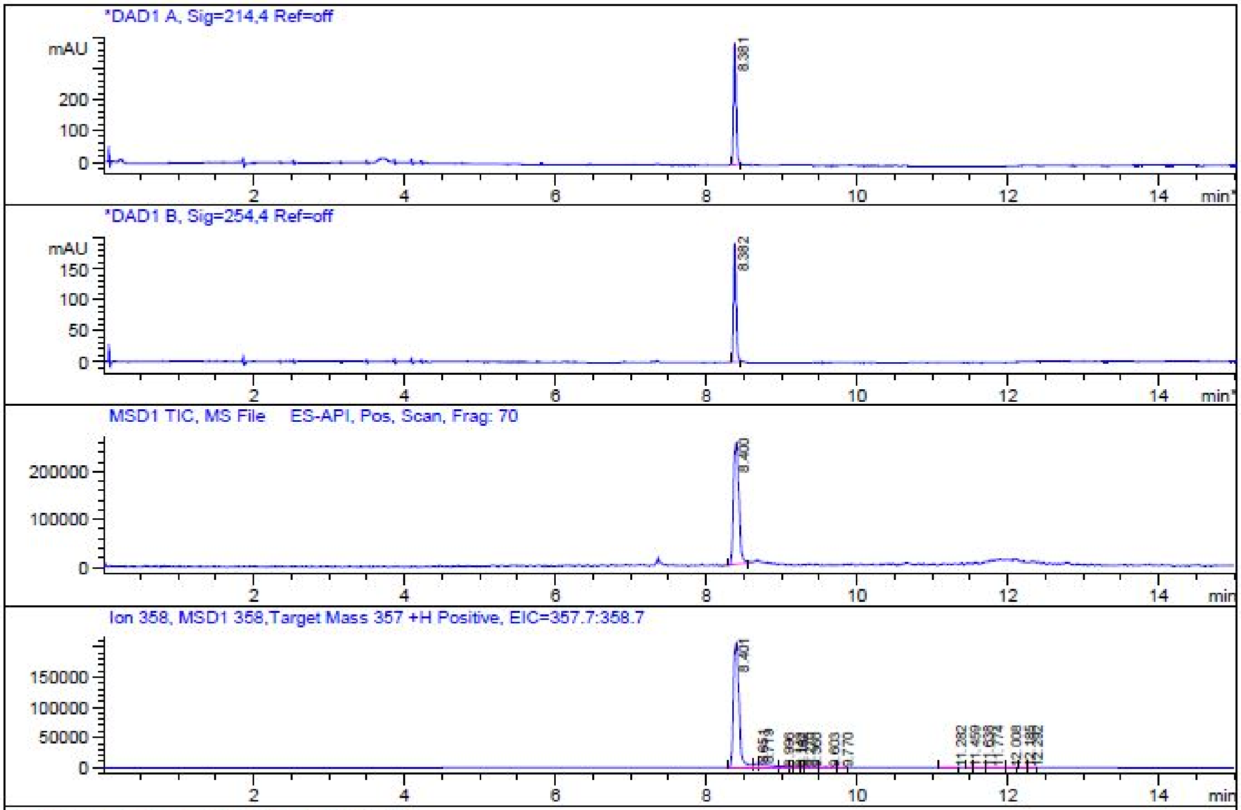

Integration Results for DAD1 A, Sig=214,4 Ref=off

RetTim	Width	Area	Height	Area%
8.38	0.04	915.81	390.17	100.00

Integration Results for DAD1 B, Sig=254,4 Ref=off

RetTim	Width	Area	Height	Area%
8.38	0.04	455.71	192.69	100.00

Ret. Time: 8.40 <<<< POSITIVE SPECTRA >>>>

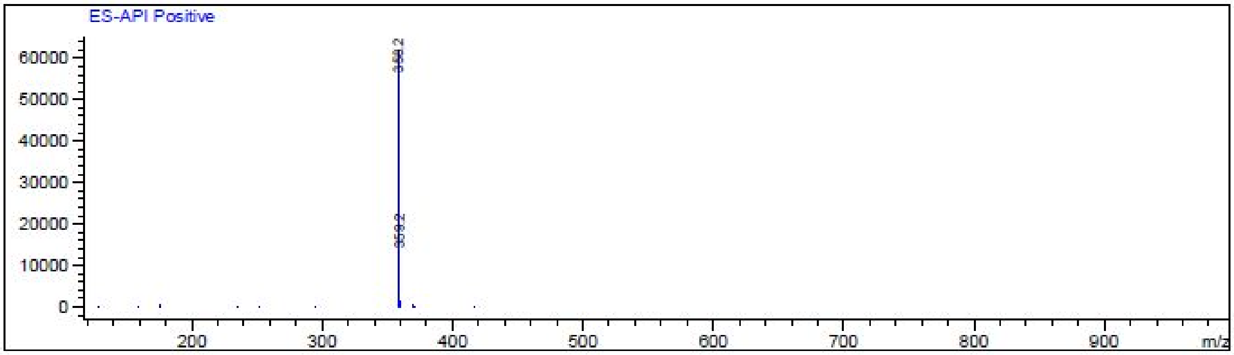

Compound 4

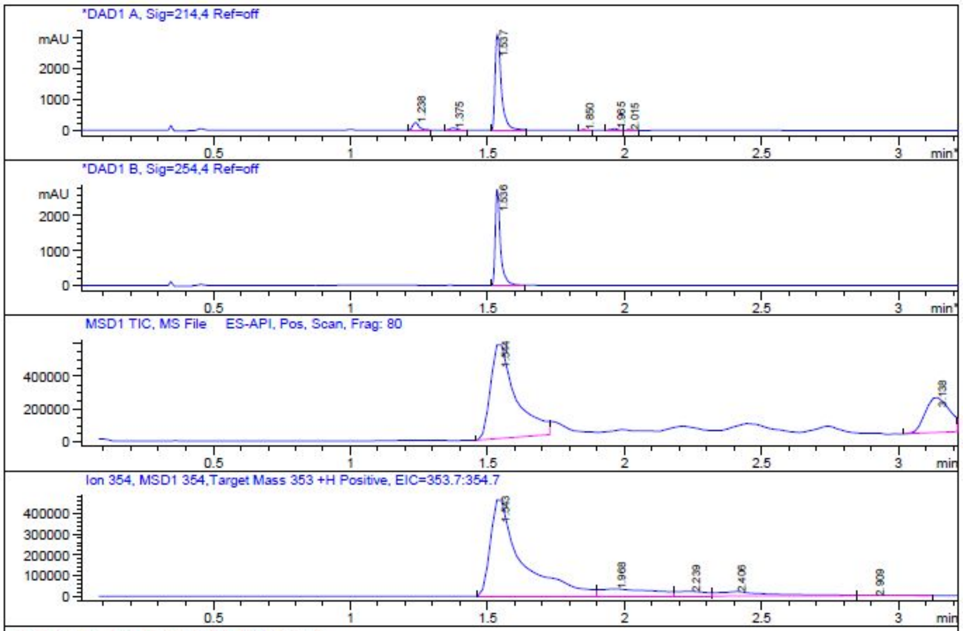

Integration Results for DAD1 A, Sig=214,4 Ref=off

RetTim	Width	Area	Height	Area%
1.24	0.02	380.19	244.22	6.91
1.38	0.03	116.38	67.52	2.12
1.54	0.02	4888.14	3091.15	88.87
1.85	0.02	16.01	13.38	0.29
1.96	0.02	77.58	46.33	1.41
2.01	0.02	22.28	17.09	0.41

Integration Results for DAD1 B, Sig=254,4 Ref=off

RetTim	Width	Area	Height	Area%
1.54	0.02	3576.34	2753.04	100.00

Ret. Time: 1.54 <<<< POSITIVE SPECTRA >>>>

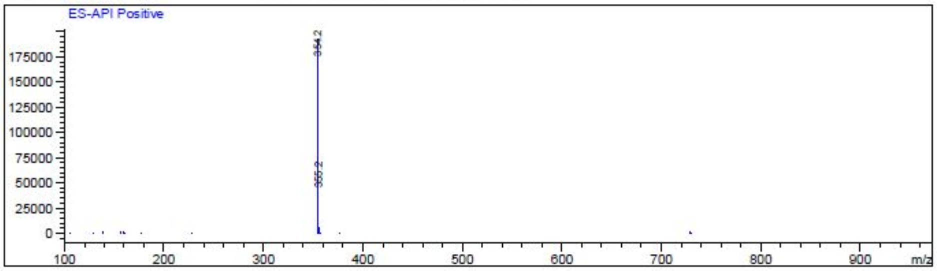

Ret. Time: 3.14

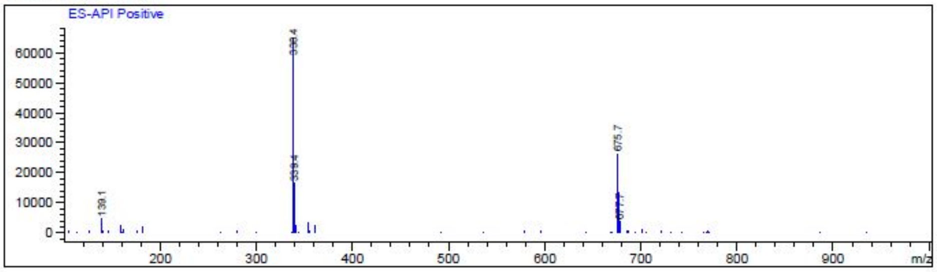

Compound 5

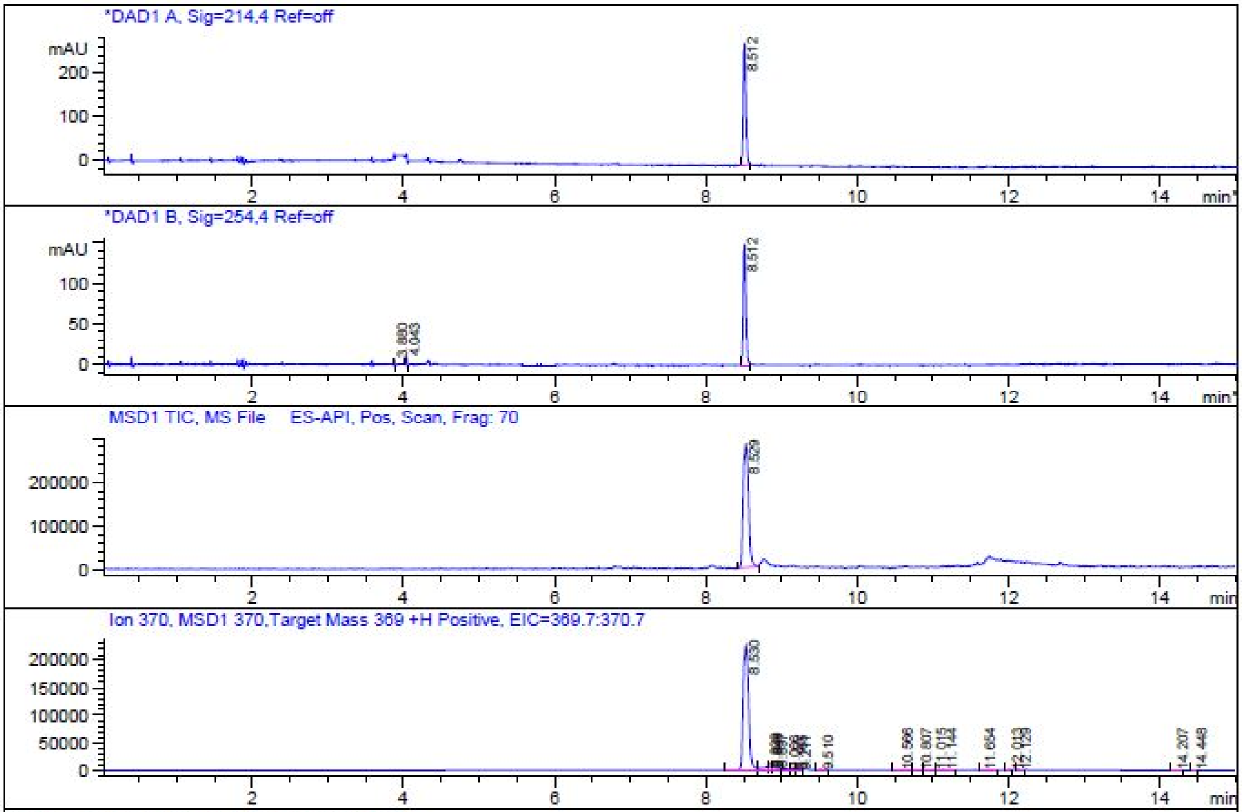

Integration Results for DAD1 A, Sig=214,4 Ref=off

RetTim	Width	Area	Height	Area%
8.51	0.04	687.42	278.71	100.00

Integration Results for DAD1 B, Sig=254,4 Ref=off

RetTim	Width	Area	Height	Area%
3.88	0.01	5.45	8.83	1.42
4.04	0.01	5.61	9.64	1.47
8.51	0.04	371.70	149.44	97.11

Ret. Time: 8.53 <<<< POSITIVE SPECTRA >>>>

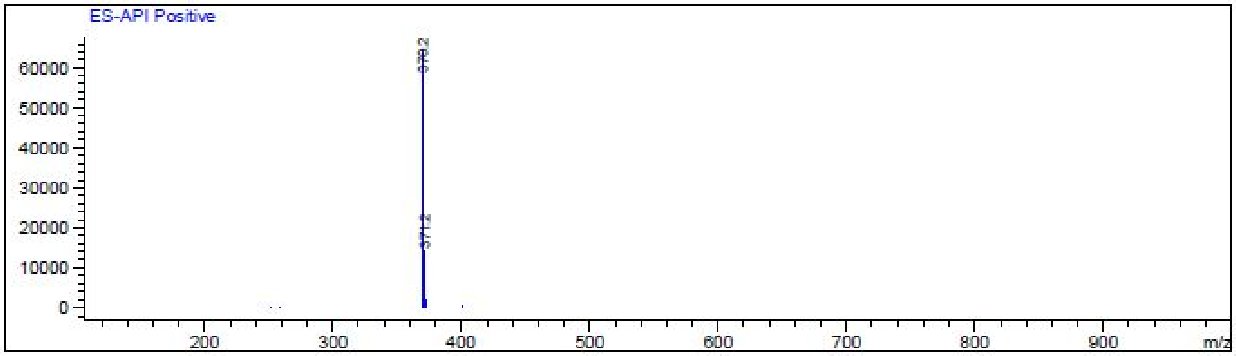

Compound 6

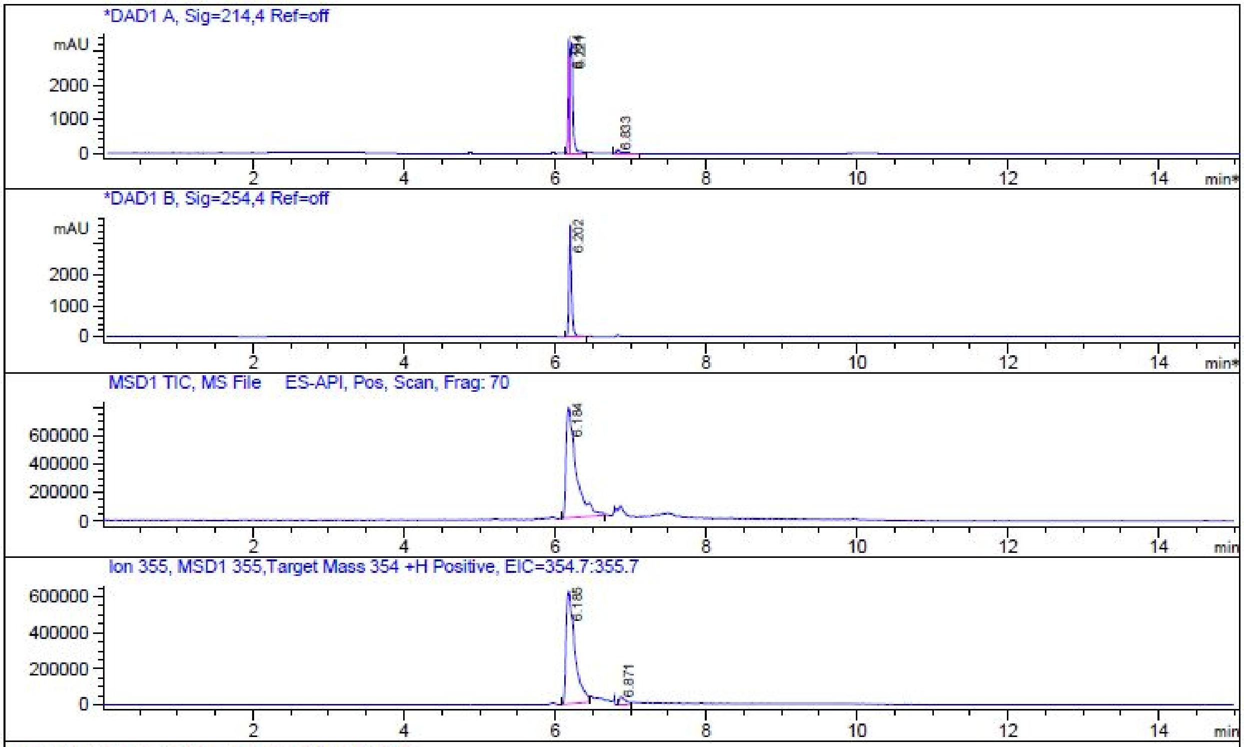

Integration Results for DAD1 A, Sig=214,4 Ref=off

RetTim	Width	Area	Height	Area%
6.18	0.02	4599.58	3284.14	36.39
6.22	0.03	7704.94	3233.25	60.97
6.83	0.05	333.68	102.09	2.64

Integration Results for DAD1 B, Sig=254,4 Ref=off

RetTim	Width	Area	Height	Area%
6.20	0.03	8424.17	3614.80	100.00

Ret. Time: 6.18 <<<< POSITIVE SPECTRA >>>>

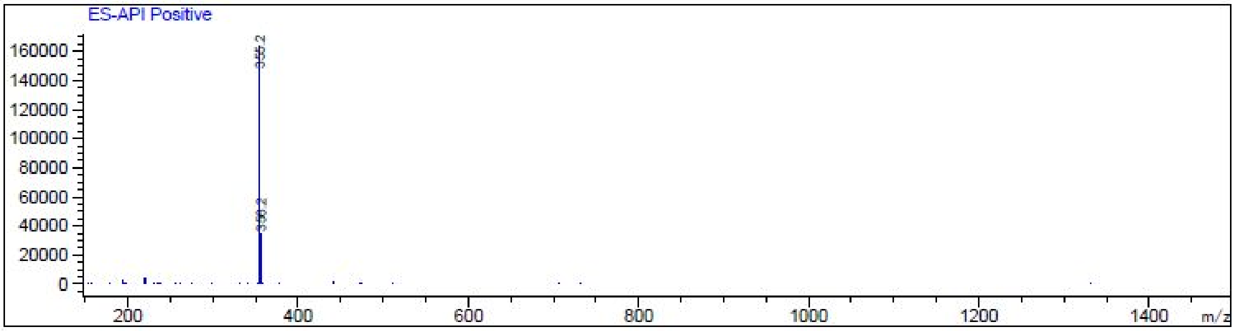

Compound 7

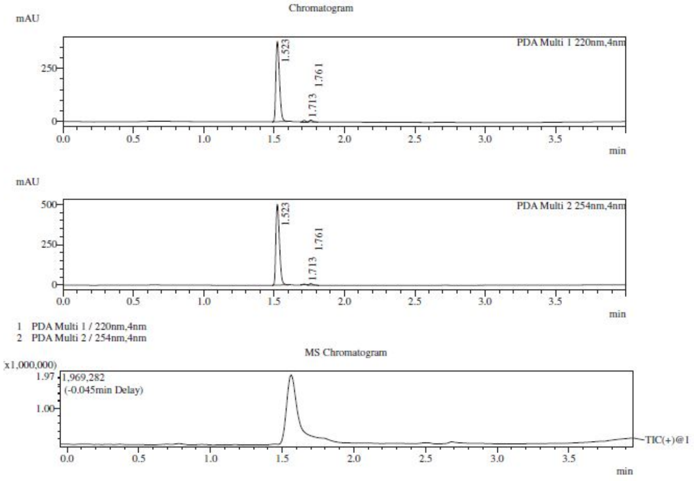

Integration Result

Peak Table

PDA Ch1 220nm

Peak#	Ret. Time	Height	Height%	USP Width	Area	Area%
1	1.523	376433	96.382	0.048	674911	97.209
2	1.713	6192	1.585	0.037	8213	1.183
3	1.761	7939	2.033	0.038	11162	1.608

Peak Table

PDA Ch2 254nm

Peak#	Ret. Time	Height	Height%	USP Width	Area	Area%
1	1.523	498076	96.776	0.048	891225	97.512
2	1.713	7023	1.365	0.036	9297	1.017
3	1.761	9568	1.859	0.038	13439	1.470

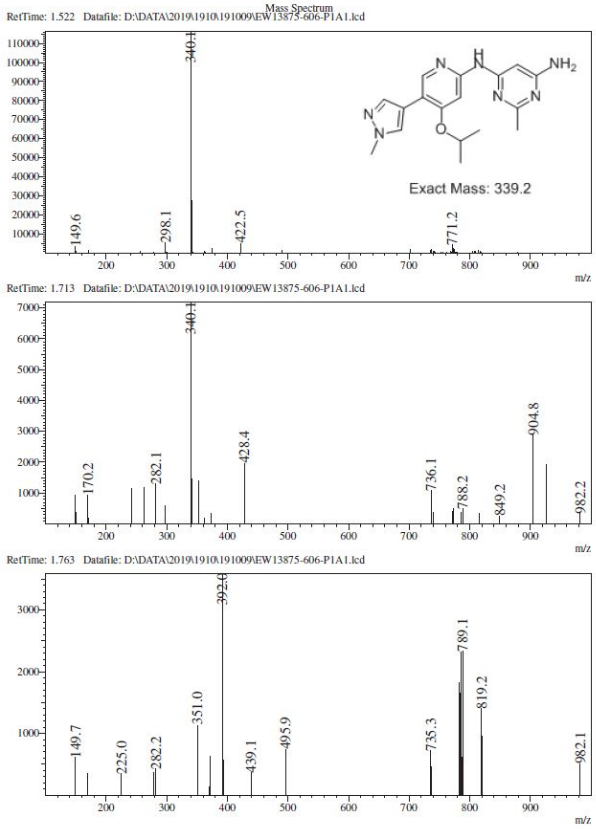

Compound 8

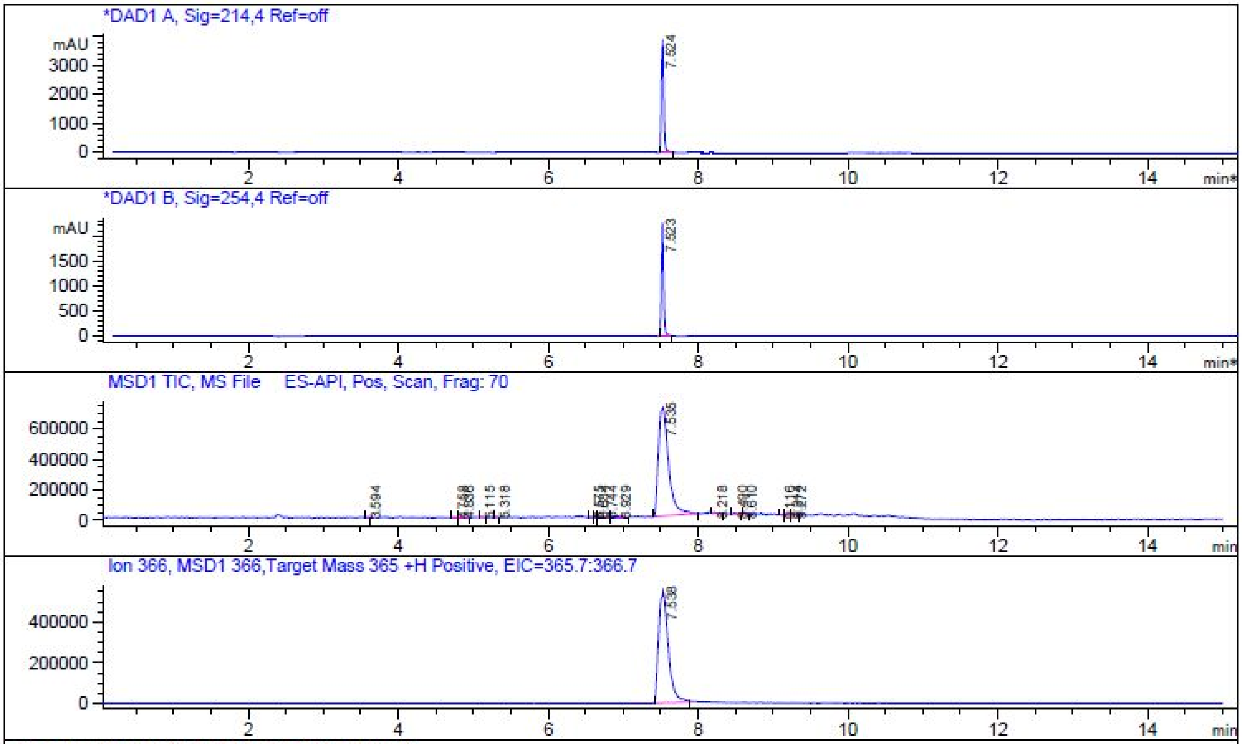

Integration Results for DAD1 A, Sig=214,4 Ref=off

RetTim	Width	Area	Height	Area%
7.52	0.04	9043.13	3673.16	100.00

Integration Results for DAD1 B, Sig=254,4 Ref=off

RetTim	Width	Area	Height	Area%
7.52	0.03	4733.47	2211.02	100.00

Ret. Time: 7.54

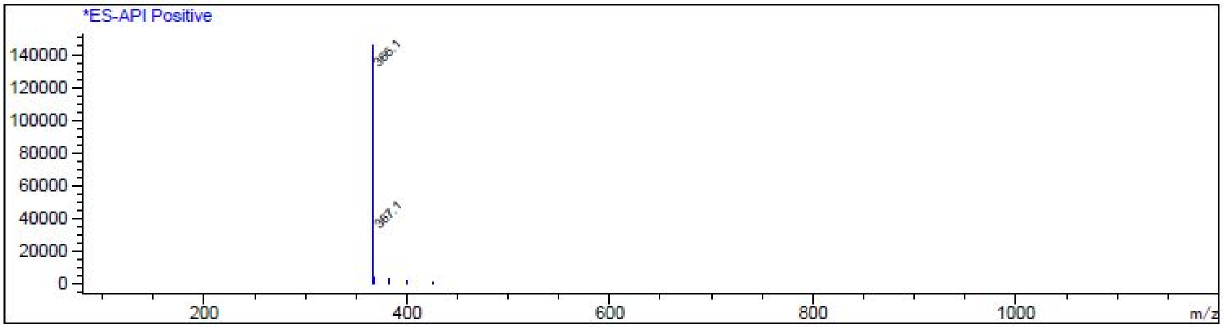

Compound 9

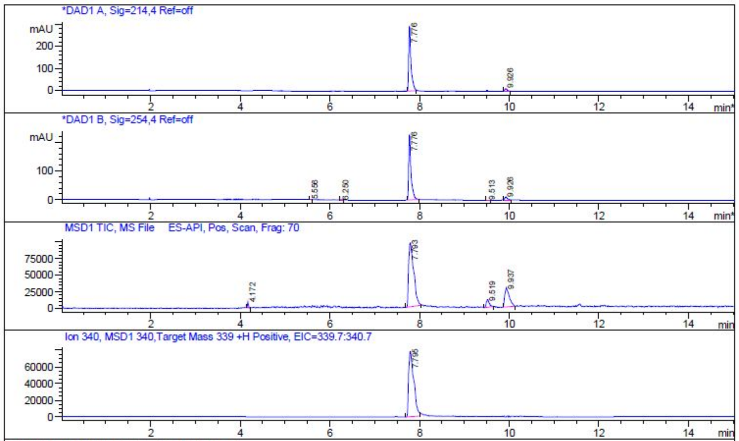

Integration Results for DAD1 A, Sig=214,4 Ref=off

RetTim	Width	Area	Height	Area%
7.78	0.05	1030.53	295.08	95.71
9.93	0.06	46.19	12.36	4.29

Integration Results for DAD1 B, Sig=254,4 Ref=off

RetTim	Width	Area	Height	Area%
5.56	0.02	0.93	0.56	0.11
6.25	0.03	0.89	0.43	0.10
7.78	0.05	806.05	227.87	94.95
9.51	0.03	0.92	0.34	0.11
9.93	0.06	40.13	10.35	4.73

Ret. Time: 4.17 <<<< POSITIVE SPECTRA >>>>

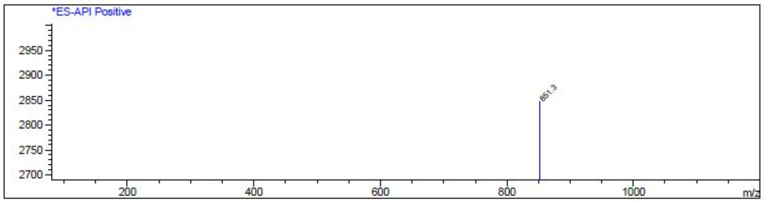

Ret. Time: 7.79

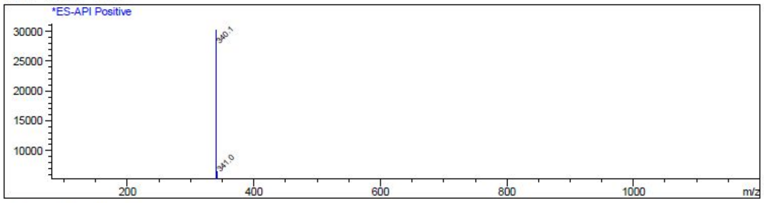

Ret. Time: 9.52

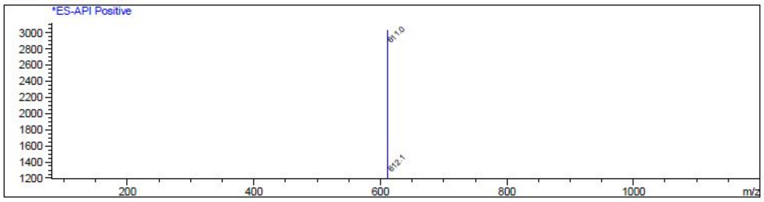

Ret. Time: 9.94

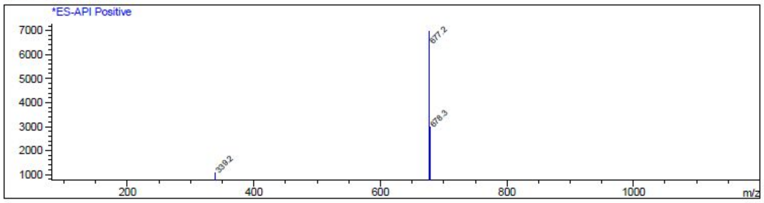

Compound 10

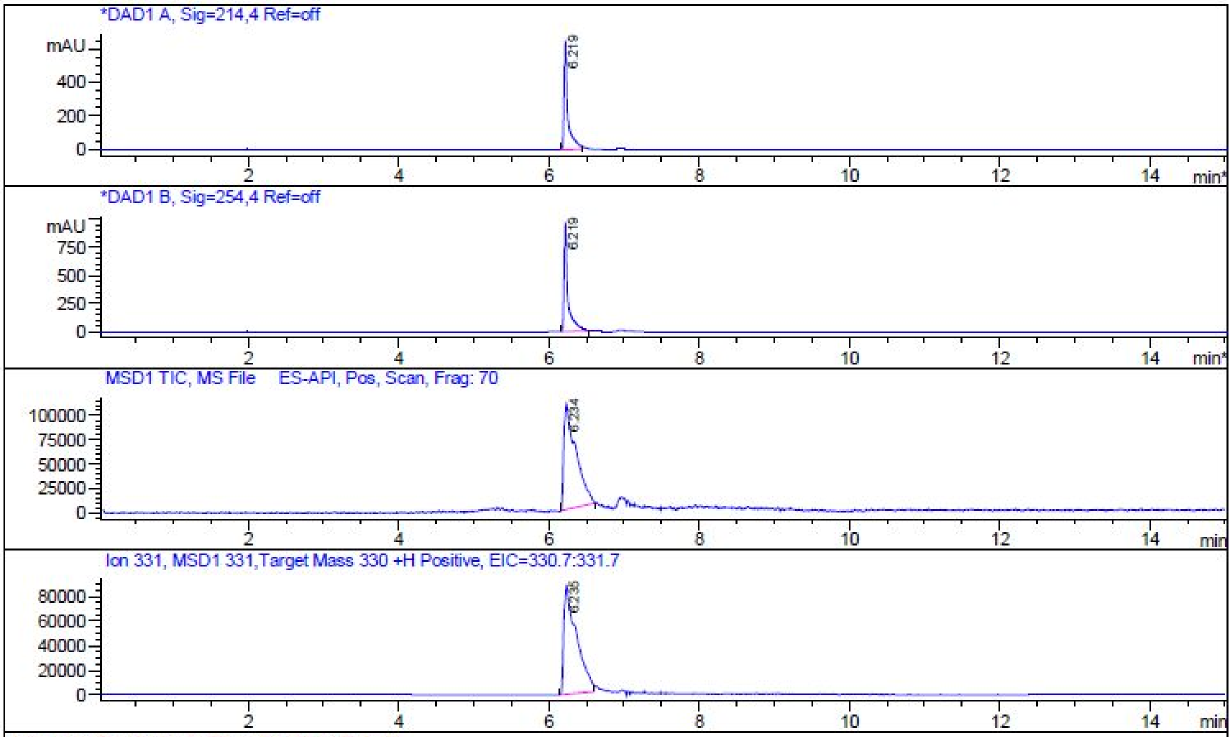

Integration Results for DAD1 A, Sig=214,4 Ref=off

RetTim	Width	Area	Height	Area%
6.22	0.05	2348.12	649.91	100.00

Integration Results for DAD1 B, Sig=254,4 Ref=off

RetTim	Width	Area	Height	Area%
6.22	0.05	3609.87	977.32	100.00

Ret. Time: 6.23 <<<< POSITIVE SPECTRA >>>>

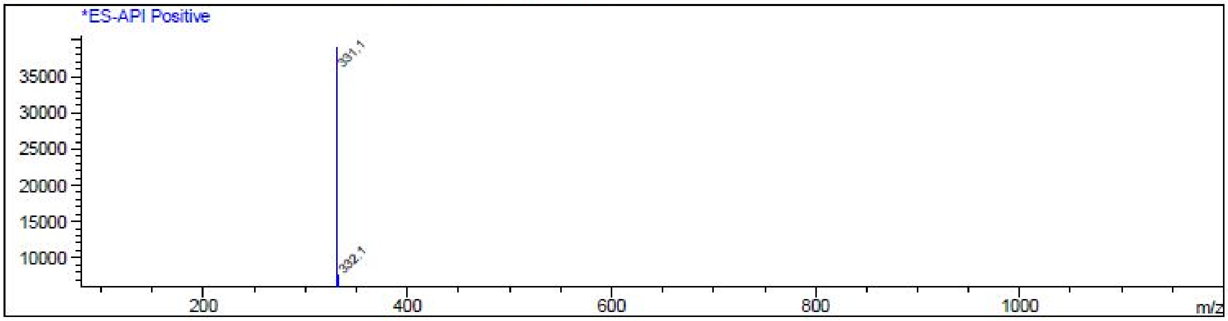

Compound 11

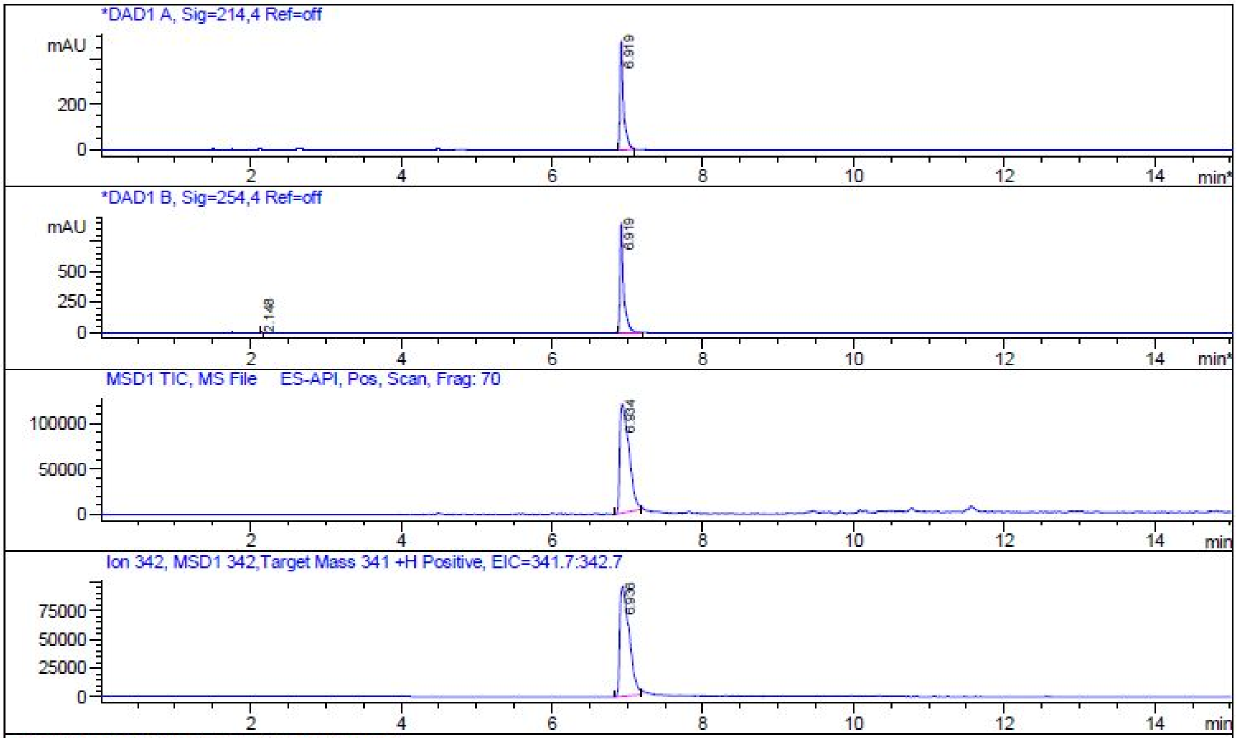

Integration Results for DAD1 A, Sig=214,4 Ref=off

RetTim	Width	Area	Height	Area%
6.92	0.05	1566.20	486.43	100.00

Integration Results for DAD1 B, Sig=254,4 Ref=off

RetTim	Width	Area	Height	Area%
2.15	0.01	2.03	2.47	0.07
6.92	0.05	2966.57	907.78	99.93

Ret. Time: 6.93 <<<< POSITIVE SPECTRA >>>>

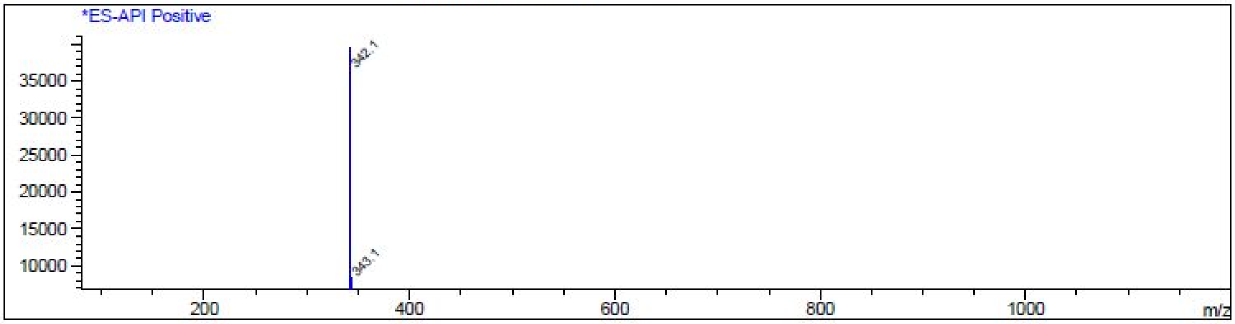

Compound 12

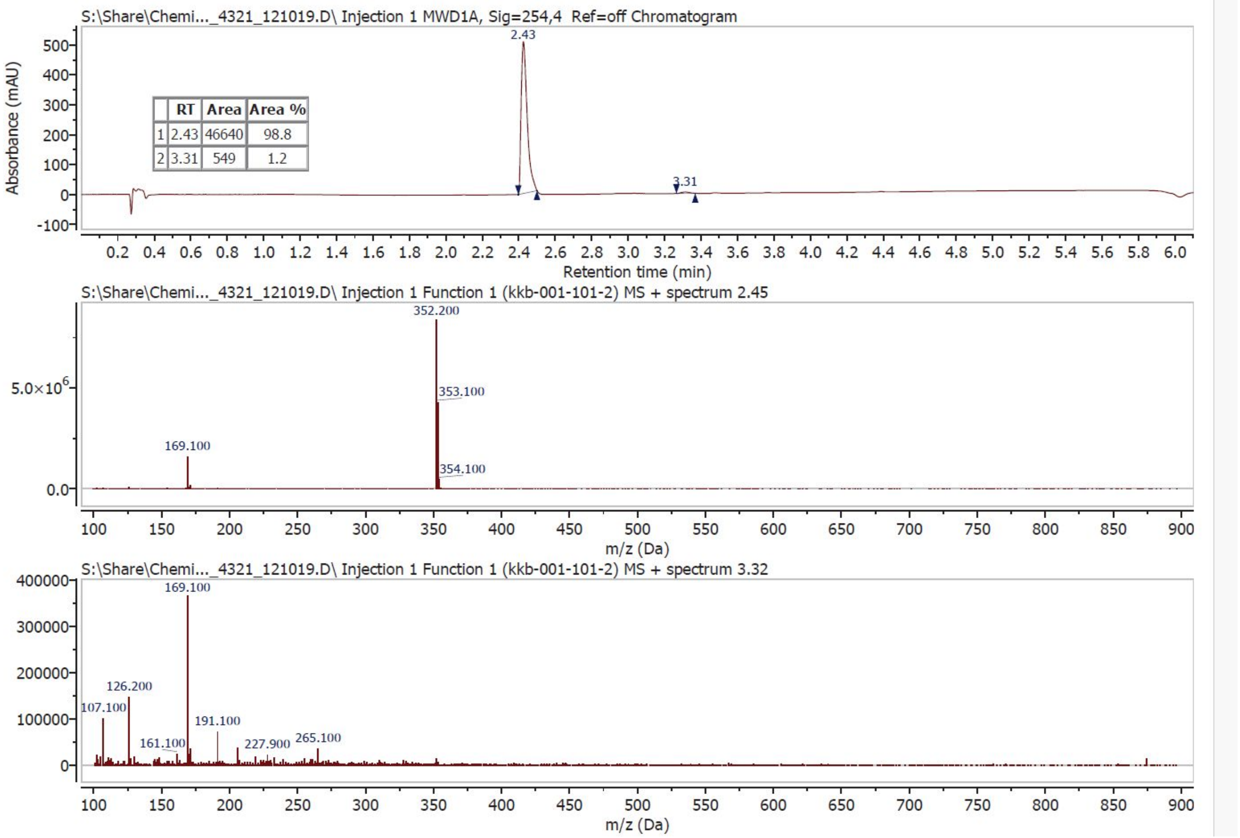
S:\Share\Chemi..._4321_121019.D\ Injection 1 MWD1A, Sig=254,4 Ref=off Chromatogram
Absorbance (mAU)
Retention time (min)
2.43
3.31
RT Area Area %
1 2.43 46640 98.8
2 3.31 549 1.2
S:\Share\Chemi..._4321_121019.D\ Injection 1 Function 1 (kkb-001-101-2) MS + spectrum 2.45
352.200
353.100
354.100
169.100
m/z (Da)
S:\Share\Chemi..._4321_121019.D\ Injection 1 Function 1 (kkb-001-101-2) MS + spectrum 3.32
169.100
126.200
107.100
161.100
191.100
227.900
265.100
m/z (Da)

Compound 13

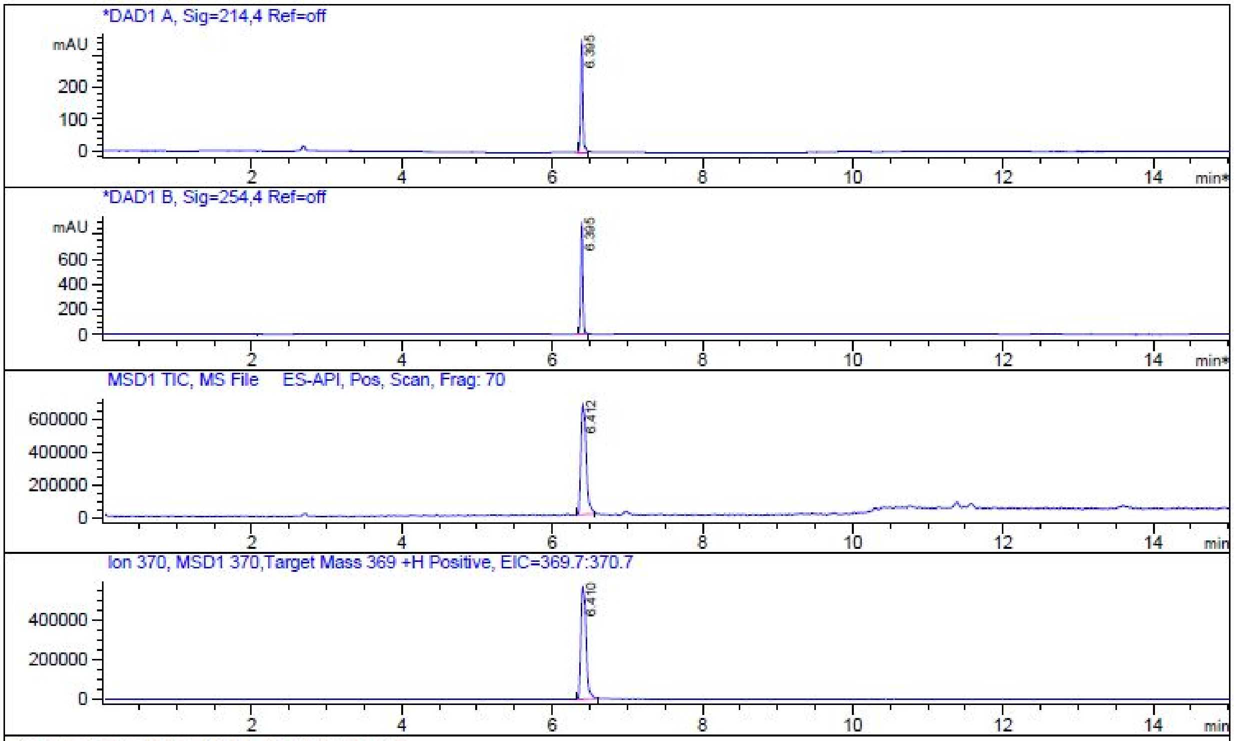

Integration Results for DAD1 A, Sig=214,4 Ref=off

RetTim	Width	Area	Height	Area%
6.40	0.03	759.96	350.00	100.00

Integration Results for DAD1 B, Sig=254,4 Ref=off

RetTim	Width	Area	Height	Area%
6.40	0.03	1871.66	882.55	100.00

Ret. Time: 6.41 <<<< POSITIVE SPECTRA >>>>

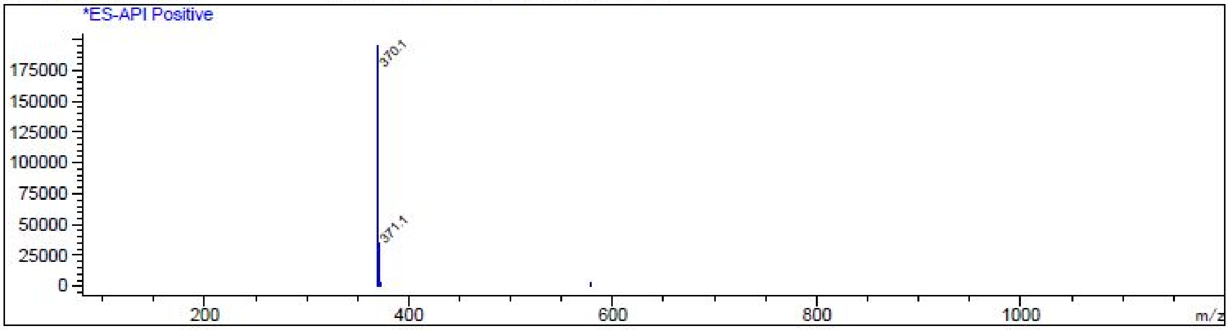

Compound 14

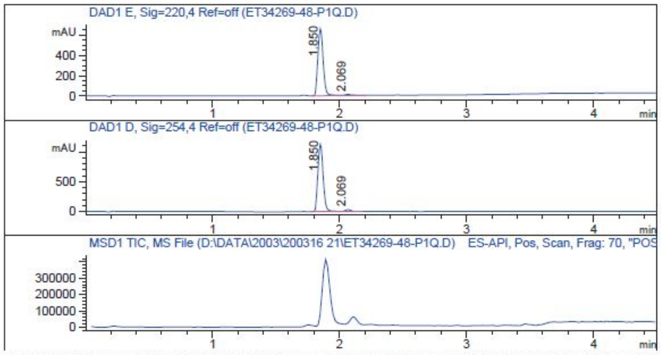

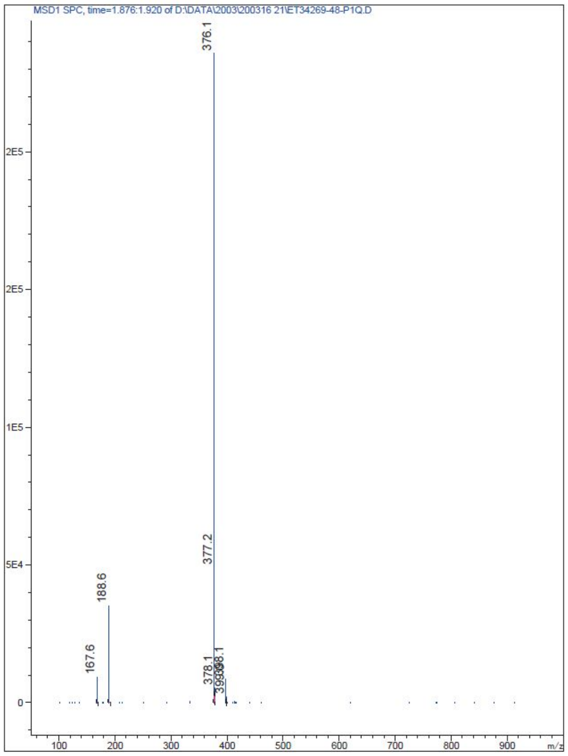

Report

Signal 1 : DAD1 E, Sig=220,4 Ref=off

Peak #	RT [min]	Height	Height %	Width [min]	Area	Area %
1	1.850	660.145	98.416	0.046	1903.682	97.176
2	2.069	10.626	1.584	0.073	55.314	2.824

Signal 2 : DAD1 D, Sig=254,4 Ref=off

Peak #	RT [min]	Height	Height %	Width [min]	Area	Area %
1	1.850	1133.753	97.107	0.046	3282.287	96.297
2	2.069	33.777	2.893	0.056	126.209	3.703

Signal 3 : MSD1 TIC, MS File

Peak #	RT [min]	Height	Height %	Width [min]	Area	Area %

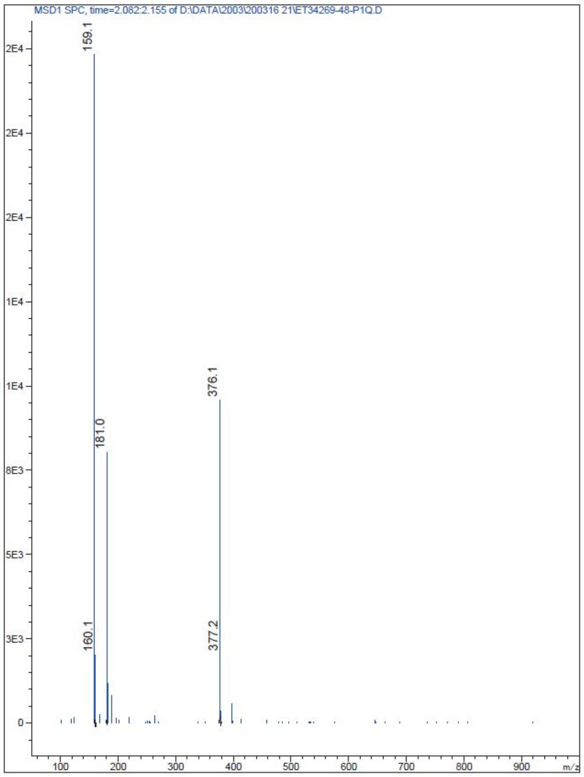

Compound 15

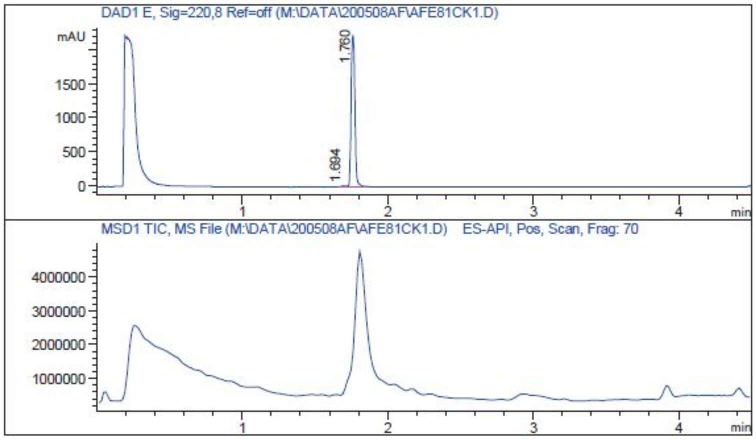

Report

Signal 1 : DAD1 E, Sig=220,8 Ref=off

Peak #	RT [min]	Height	Height %	Width [min]	Area	Area %
1	1.694	17.870	0.795	0.031	38.372	0.946
2	1.760	2228.689	99.205	0.030	4018.192	99.054

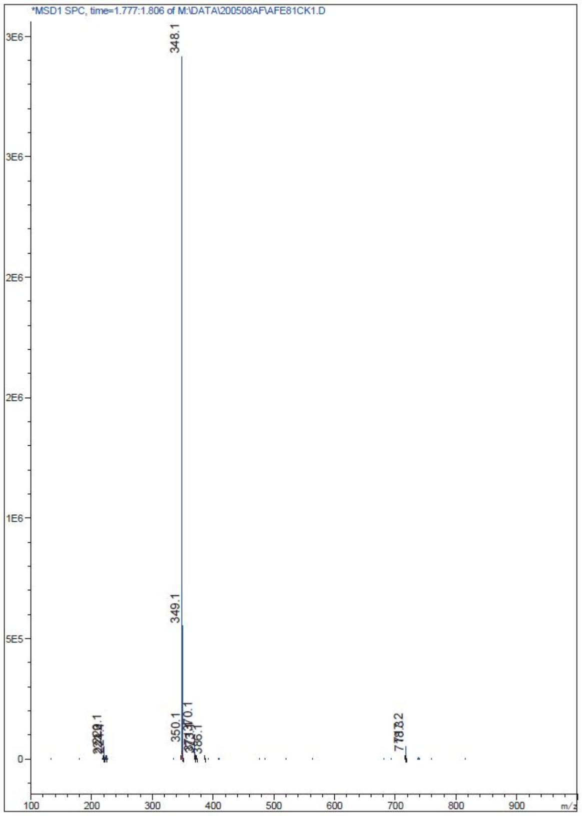

Compound 16

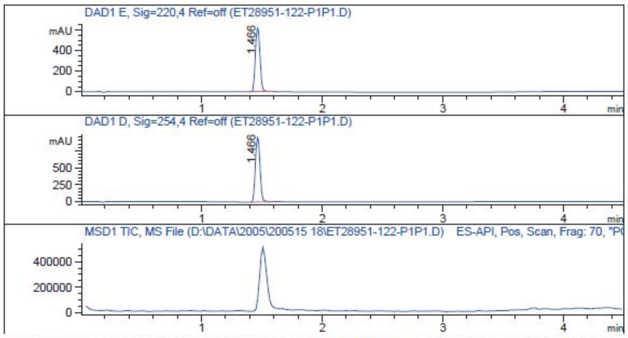

Report

Signal 1 : DAD1 E, Sig=220,4 Ref=off

Peak #	RT [min]	Height	Height %	Width [min]	Area	Area %
1	1.466	621.524	100.000	0.043	1670.842	100.000

Signal 2 : DAD1 D, Sig=254,4 Ref=off

Peak #	RT [min]	Height	Height %	Width [min]	Area	Area %
1	1.466	944.859	100.000	0.043	2541.119	100.000

Signal 3 : MSD1 TIC, MS File

Peak #	RT [min]	Height	Height %	Width [min]	Area	Area %

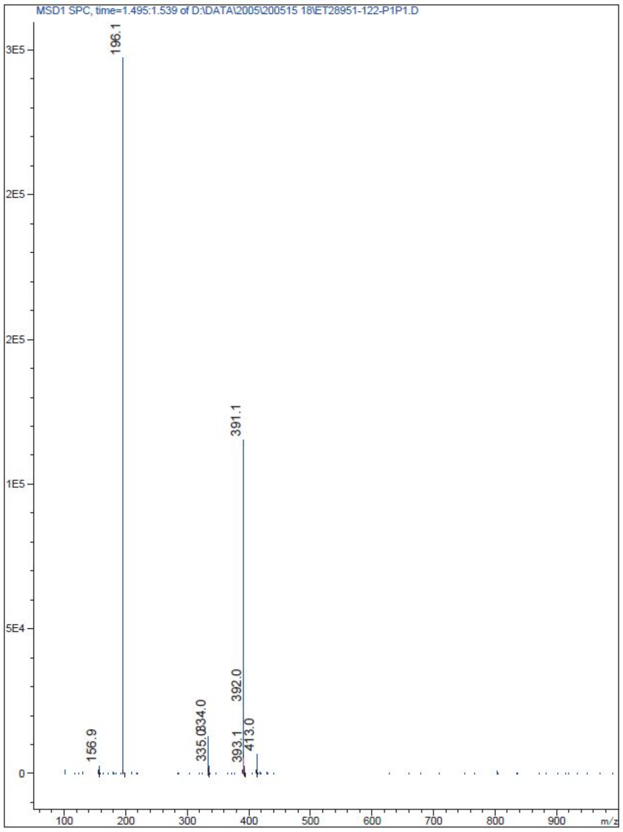

Compound 17

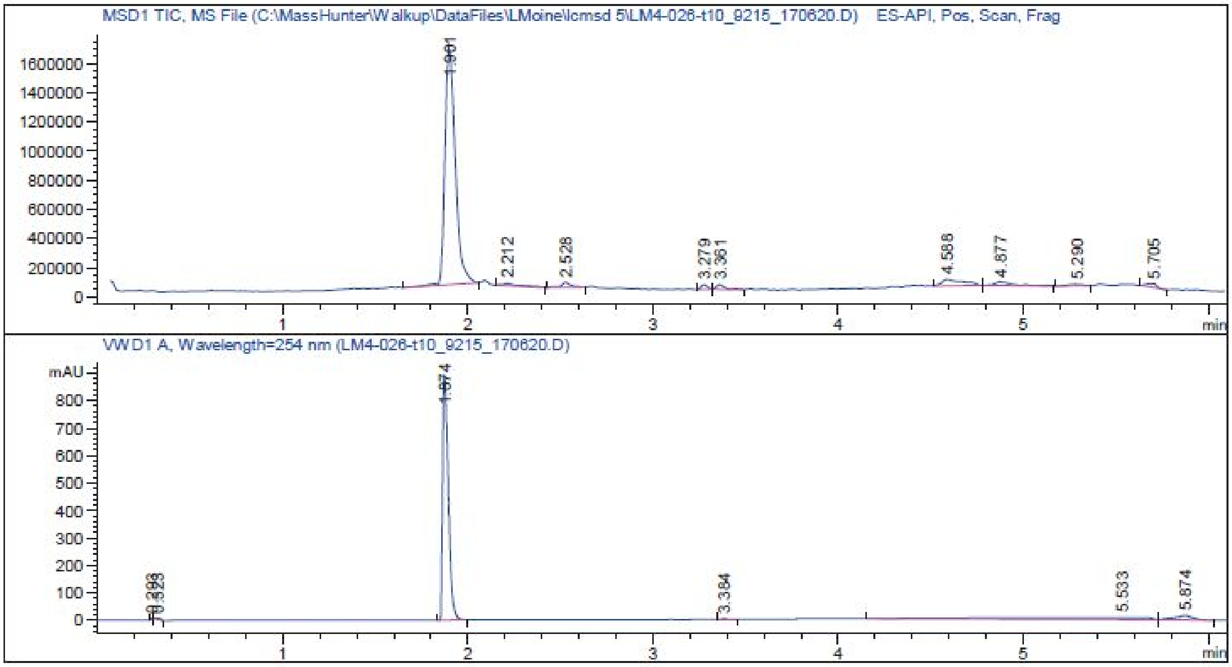

Retention Time (MS)	MS Area	Mol. Weight or Ion
1.901	6381981	407.10 I<br>406.10 I
2.212	104390	
2.528	126295	213.10 I
3.279	75390	239.10 I
3.361	99934	239.10 I
4.588	361591	102.20 I
4.877	194022	102.15 I
5.290	59135	102.20 I
5.705	97049	

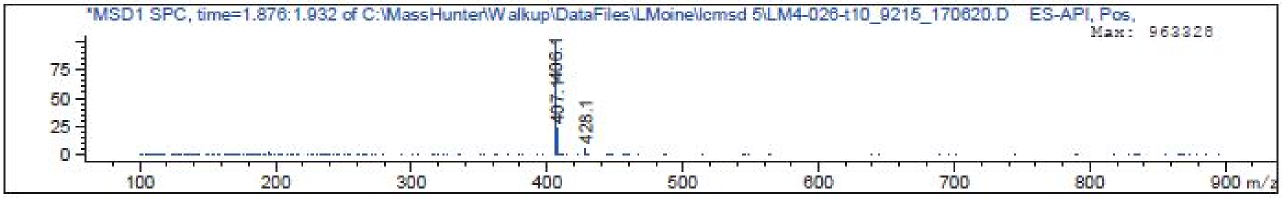

Compound 18

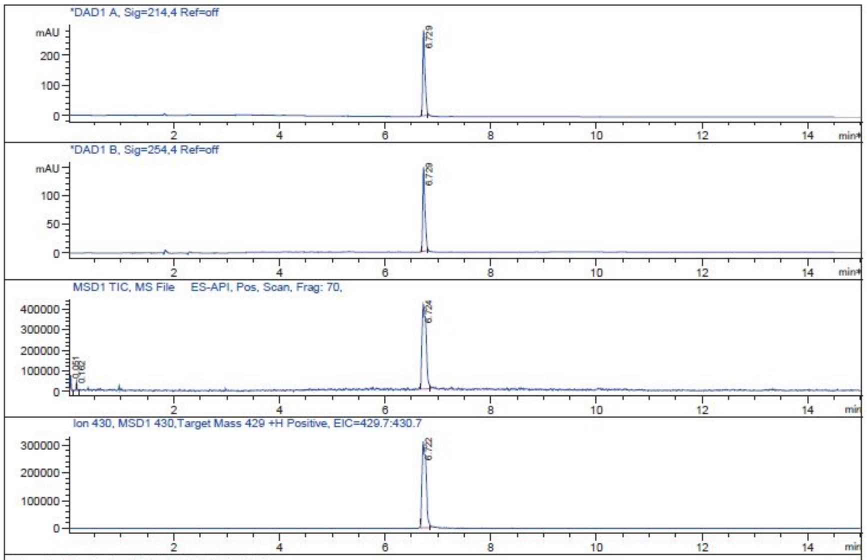

Integration Results for DAD1 A, Sig=214,4 Ref=off

RetTim	Width	Area	Height	Area%
6.73	0.04	841.78	285.61	100.00

Integration Results for DAD1 B, Sig=254,4 Ref=off

RetTim	Width	Area	Height	Area%
6.73	0.04	430.81	146.95	100.00

Ret. Time: 0.05 <<<< POSITIVE SPECTRA >>>>

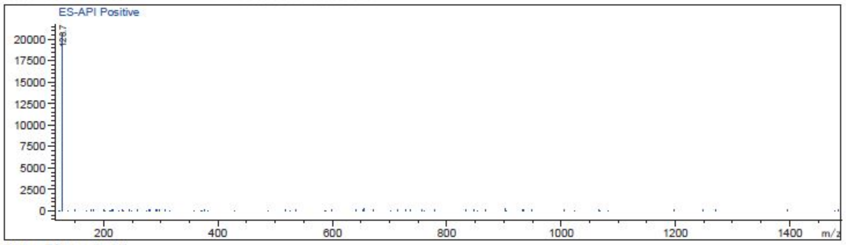

Ret. Time: 0.16

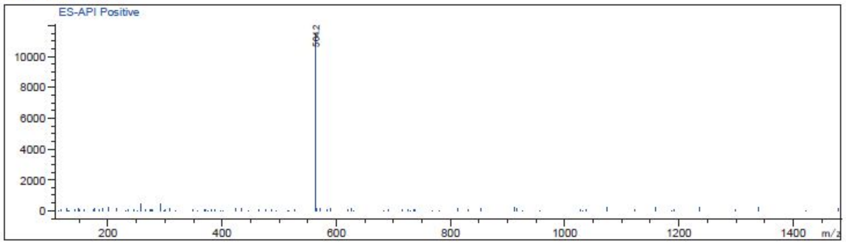

Ret. Time: 6.72

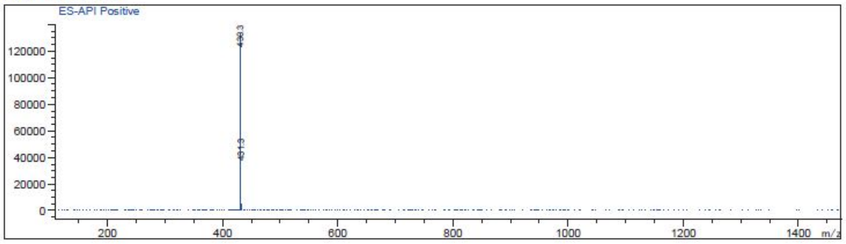

11. NMR spectra of Compounds 1-18

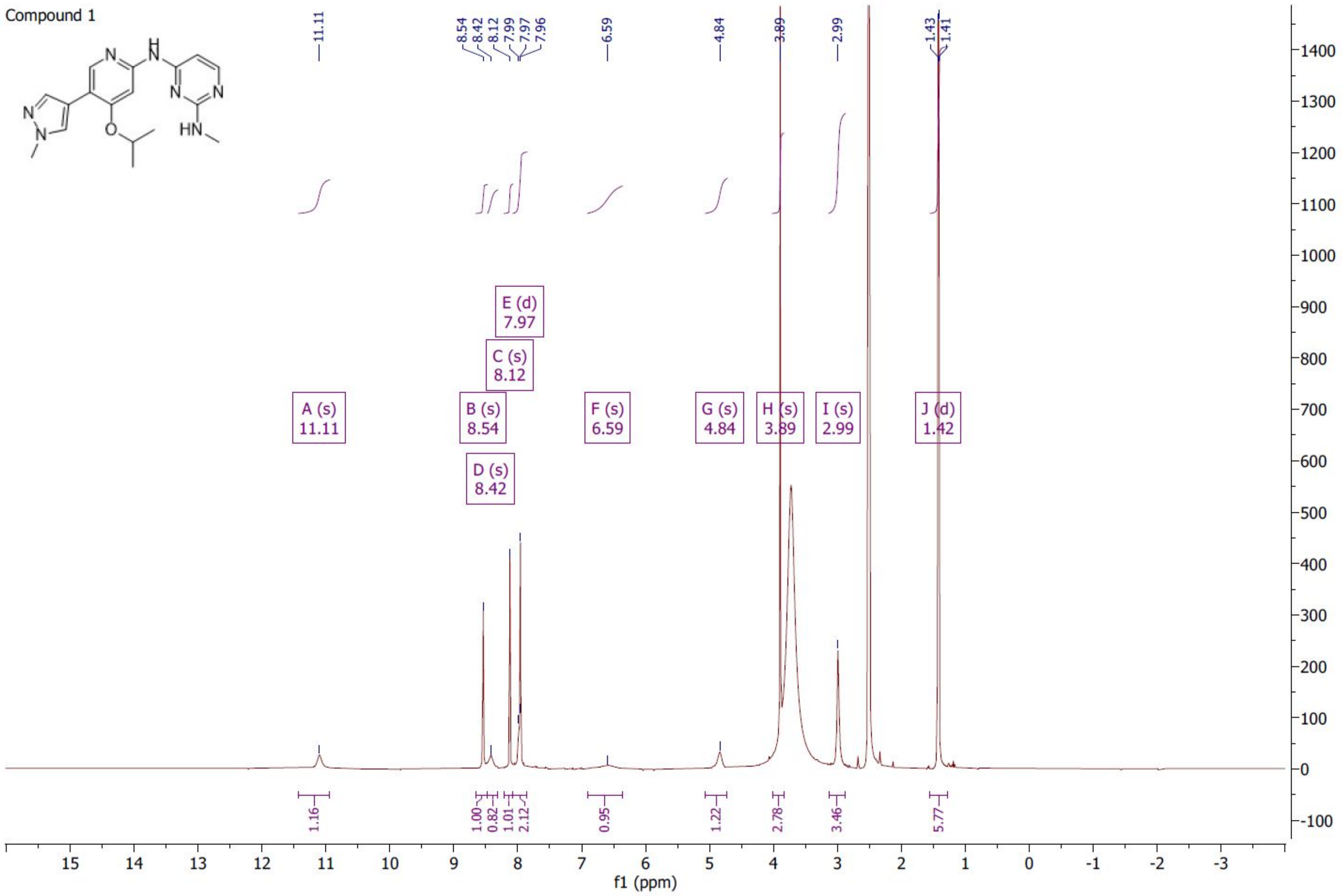

^{1}H NMR (400 MHz, DMSO) δ 11.11 (s, 1H), 8.54 (s, 1H), 8.42 (s, 1H), 8.12 (s, 1H), 7.97 (d, J = 11.3 Hz, 2H), 6.59 (s, 1H), 4.84 (s, 1H), 3.89 (s, 3H), 2.99 (s, 3H), 1.42 (d, J = 6.0 Hz, 6H).

Compound 2

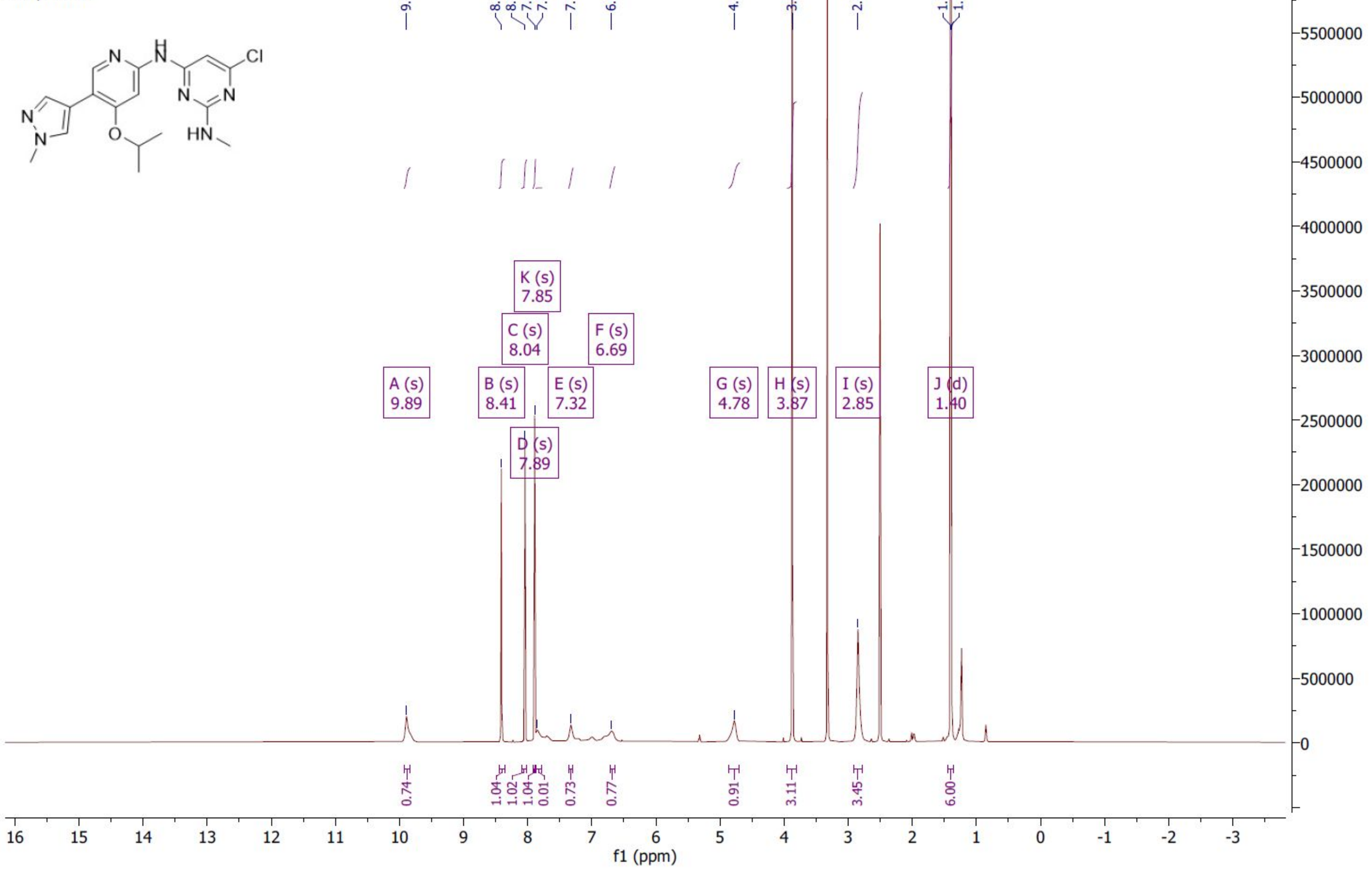

^{1}H NMR (500 MHz, DMSO) δ 9.89 (s, 1H), 8.41 (s, 1H), 8.04 (s, 1H), 7.89 (s, 1H), 7.85 (s, 1H), 7.32 (s, 1H), 6.69 (s, 1H), 4.78 (s, 1H), 3.87 (s, 3H), 2.85 (s, 3H), 1.40 (d, J = 6.1 Hz, 6H).

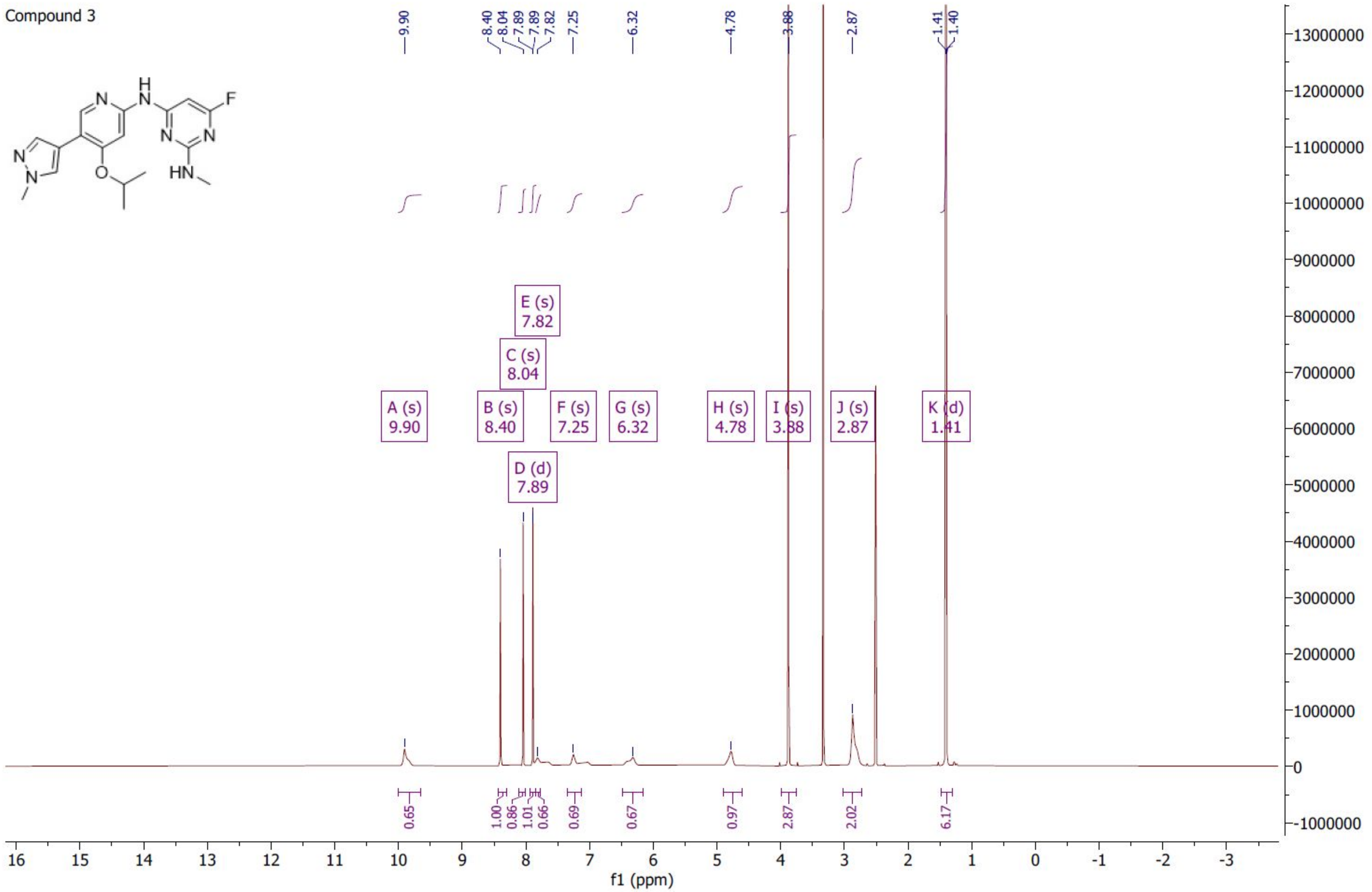

^{1}H NMR (500 MHz, DMSO) δ 9.90 (s, 1H), 8.40 (s, 1H), 8.04 (s, 1H), 7.89 (d, J = 0.7 Hz, 1H), 7.82 (s, 1H), 7.25 (s, 1H), 6.32 (s, 1H), 4.78 (s, 1H), 3.88 (s, 3H), 2.87 (s, 2H), 1.41 (d, J = 6.0 Hz, 6H).

Compound 4

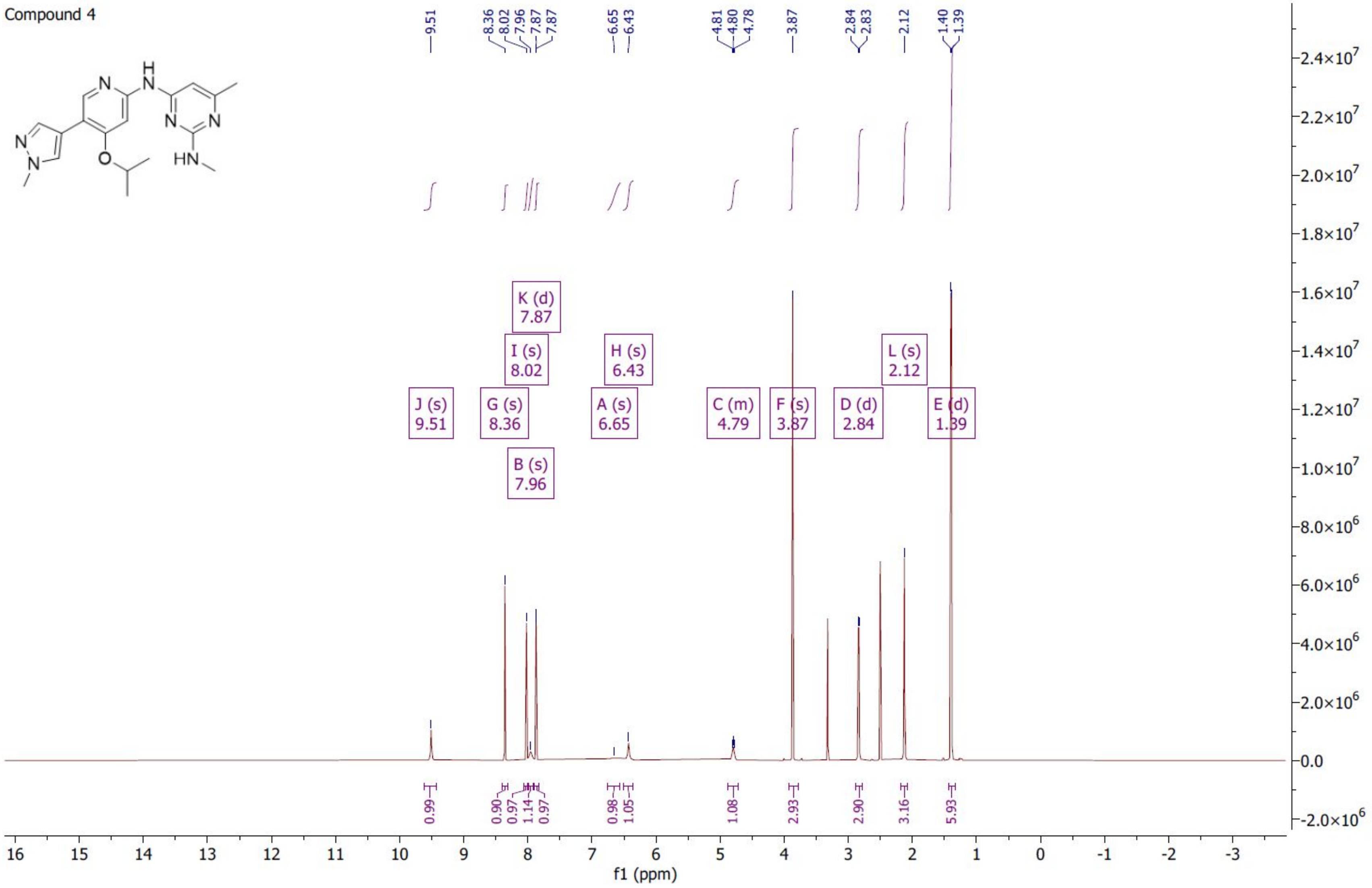

^{1}H NMR (500 MHz, DMSO) δ 9.51 (s, 1H), 8.36 (s, 1H), 8.02 (s, 1H), 7.96 (s, 1H), 7.87 (d, J = 0.7 Hz, 1H), 6.65 (s, 1H), 6.43 (s, 1H), 4.88 – 4.71 (m, 1H), 3.87 (s, 3H), 2.84 (d, J = 4.7 Hz, 3H), 2.12 (s, 3H), 1.39 (d, J = 6.0 Hz, 6H).

Compound 5

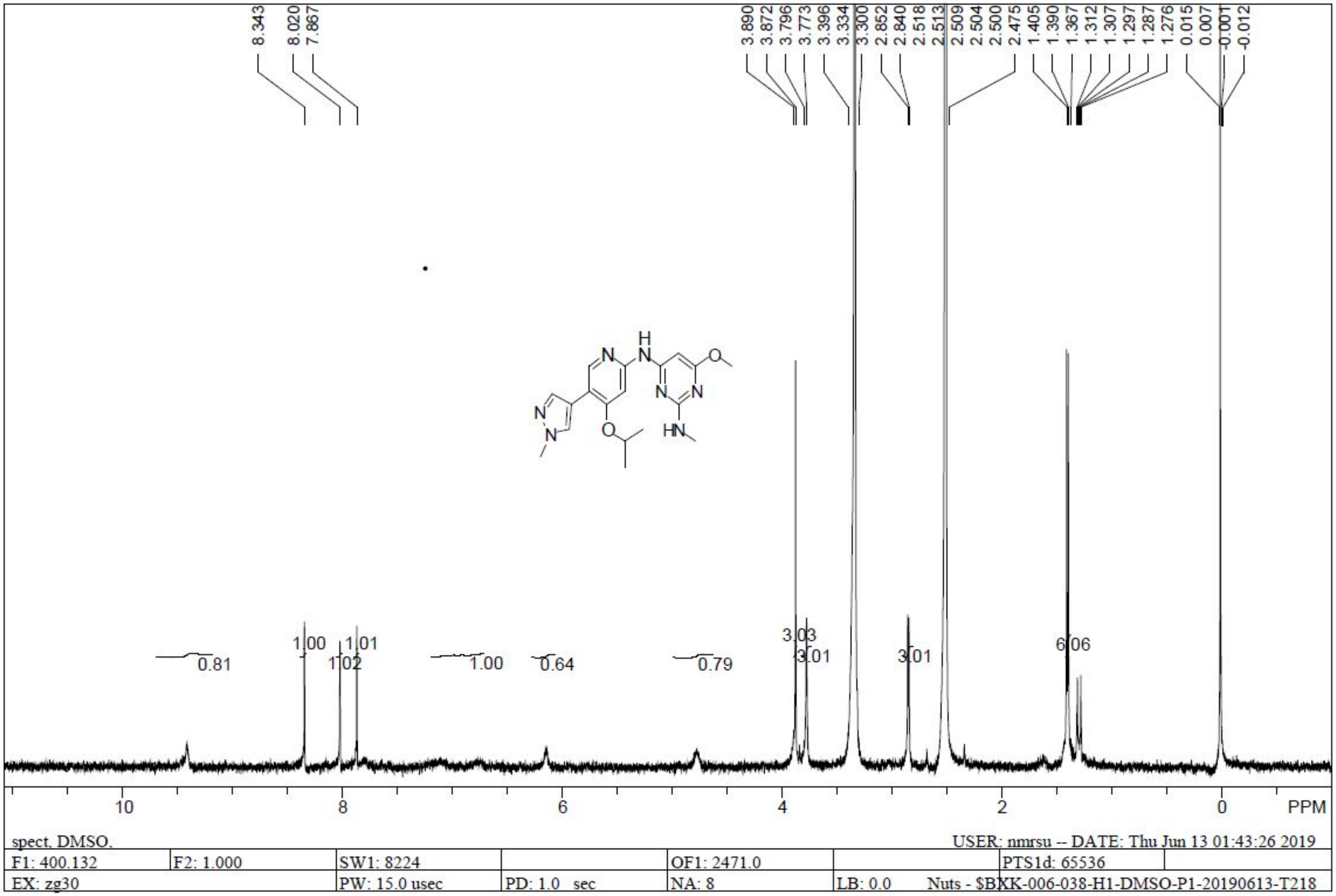

8.343
8.020
7.867
3.890
3.872
3.796
3.773
3.396
3.334
3.300
2.852
2.840
2.518
2.513
2.509
2.504
2.500
2.475
1.405
1.390
1.367
1.312
1.307
1.297
1.287
1.276
0.015
0.007
-0.001
-0.012
0.81
1.00
1.02
1.01
1.00
0.64
0.79
3.03
3.01
3.01
6.06
10
8
6
4
2
0
PPM
spect, DMSO,
USER: nmrsu -- DATE: Thu Jun 13 01:43:26 2019
F1: 400.132
F2: 1.000
SW1: 8224
OF1: 2471.0
PTS1d: 65536
EX: zg30
PW: 15.0 usec
PD: 1.0 sec
NA: 8
LB: 0.0
Nuts - $BXK-006-038-H1-DMSO-P1-20190613-T218

Compound 6

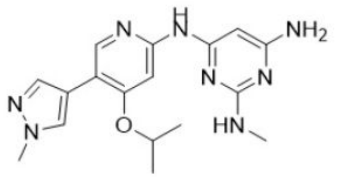

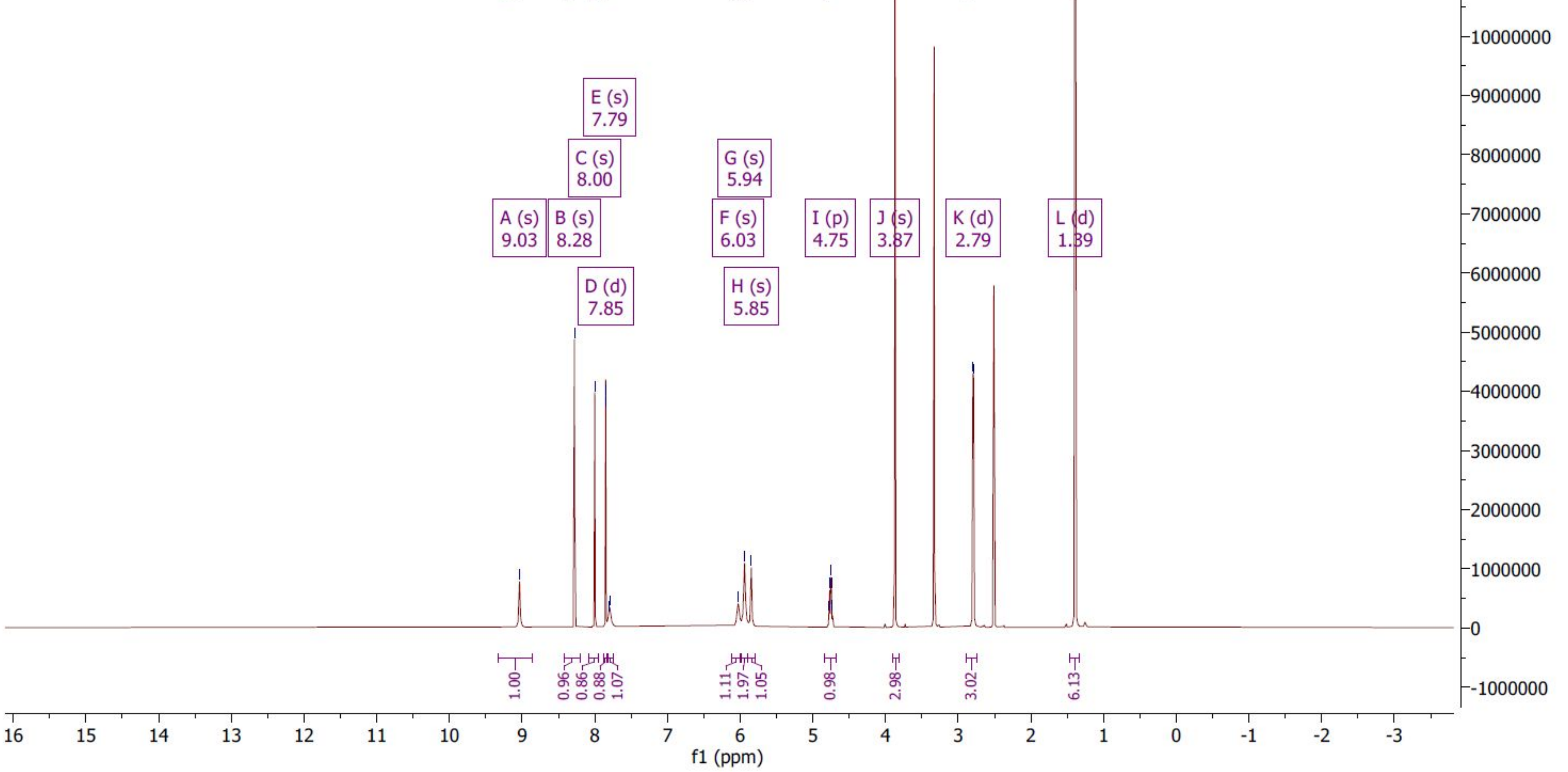

^{1}H NMR (500 MHz, DMSO) δ 9.03 (s, 1H), 8.28 (s, 1H), 8.00 (s, 1H), 7.85 (d, J = 0.9 Hz, 1H), 7.79 (s, 1H), 6.03 (s, 1H), 5.94 (s, 2H), 5.85 (s, 1H), 4.75 (p, J = 6.0 Hz, 1H), 3.87 (s, 3H), 2.79 (d, J = 4.8 Hz, 3H), 1.39 (d, J = 6.0 Hz, 6H).

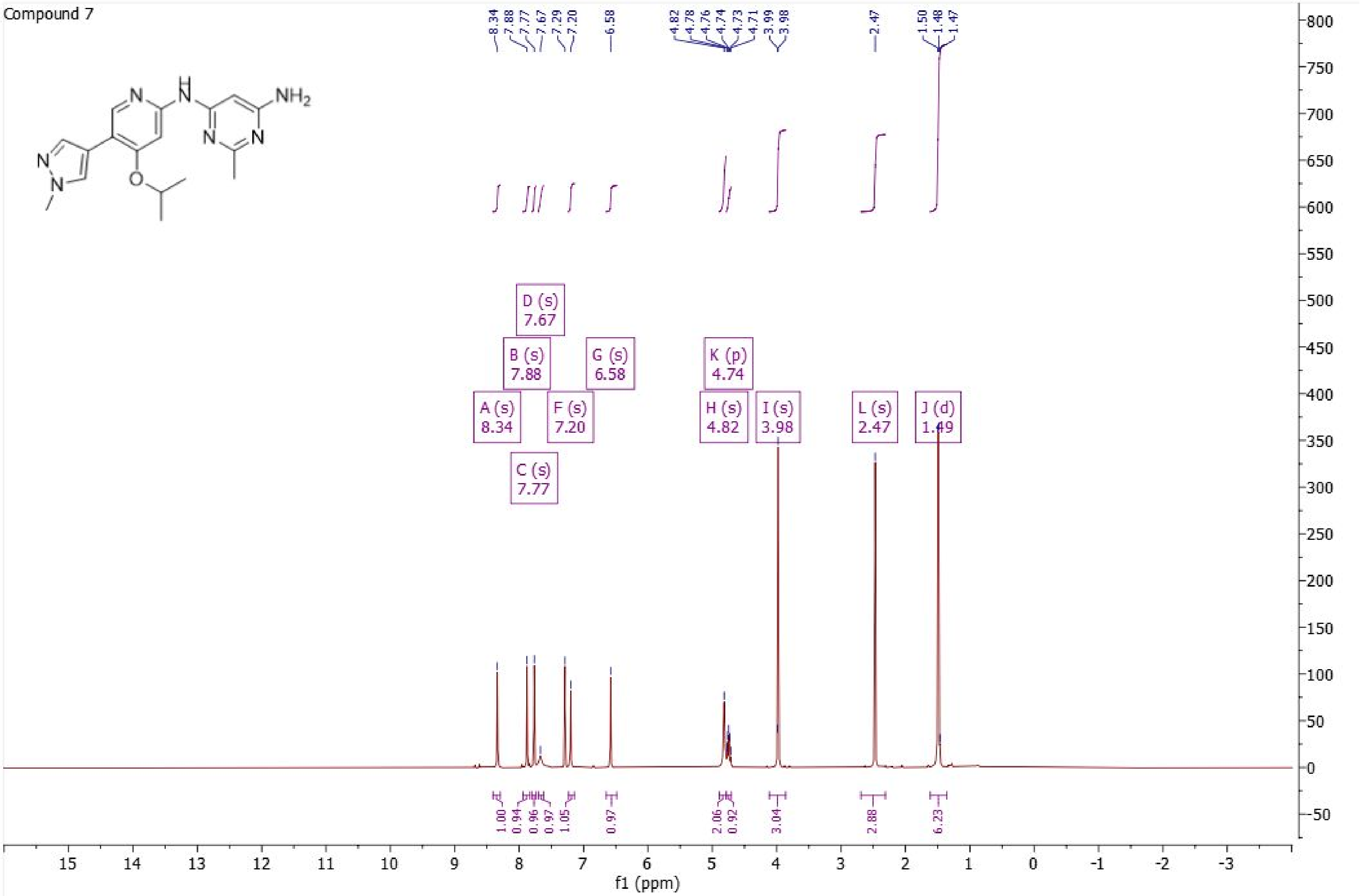

^{1}H NMR (400 MHz, $CDCl_3$) δ 8.34 (s, 1H), 7.88 (s, 1H), 7.77 (s, 1H), 7.67 (s, 1H), 7.20 (s, 1H), 6.58 (s, 1H), 4.82 (s, 2H), 4.74 (p, J = 6.1 Hz, 1H), 3.98 (s, 3H), 1.49 (d, J = 6.0 Hz, 6H).

Compound 8

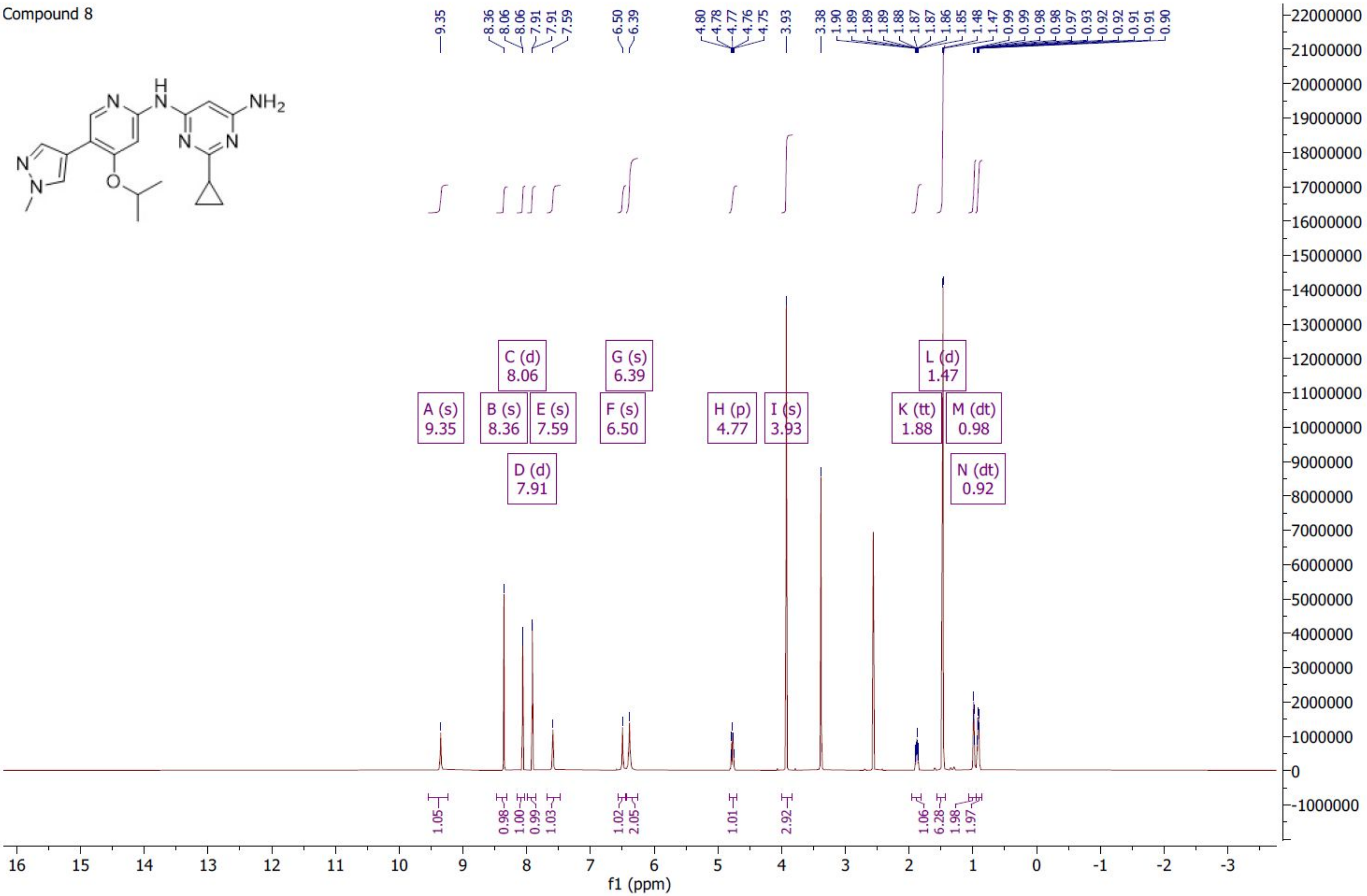

^{1}H NMR (500 MHz, DMSO) δ 9.35 (s, 1H), 8.36 (s, 1H), 8.06 (d, J = 0.7 Hz, 1H), 7.91 (d, J = 0.8 Hz, 1H), 7.59 (s, 1H), 6.50 (s, 1H), 6.39 (s, 2H), 4.77 (p, J = 6.0 Hz, 1H), 3.93 (s, 3H), 1.88 (tt, J = 8.1, 4.7 Hz, 1H), 1.47 (d, J = 6.0 Hz, 6H), 0.98 (dt, J = 5.4, 2.8 Hz, 2H), 0.92 (dt, J = 8.3, 3.1 Hz, 2H).

Compound 9

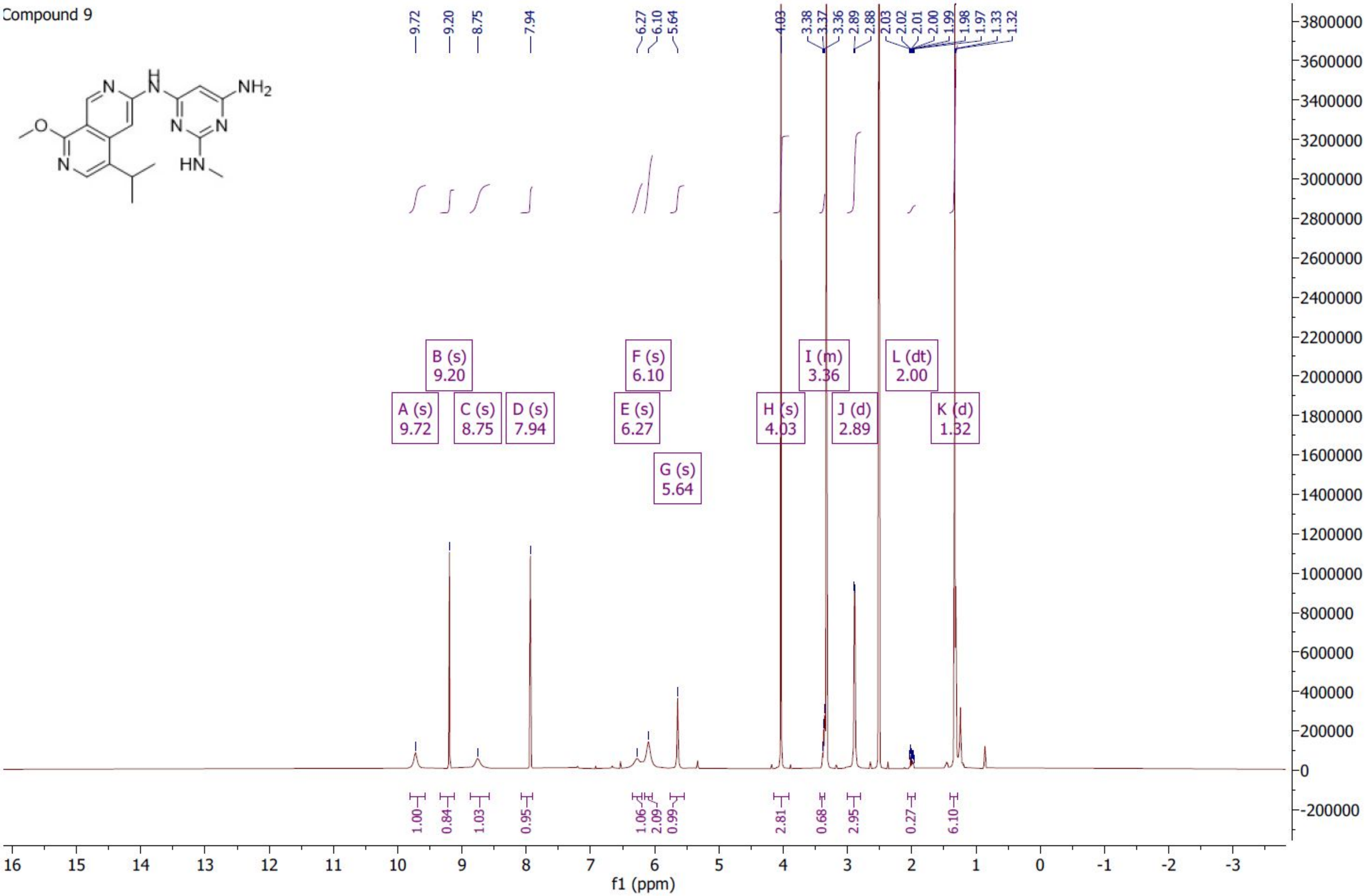

^{1}H NMR (500 MHz, DMSO) δ 9.72 (s, 1H), 9.20 (s, 1H), 8.75 (s, 1H), 7.94 (s, 1H), 6.27 (s, 1H), 6.10 (s, 2H), 5.64 (s, 1H), 4.03 (s, 3H), 3.43 – 3.35 (m, 1H), 2.89 (d, J = 4.7 Hz, 3H), 1.32 (d, J = 6.8 Hz, 6H).

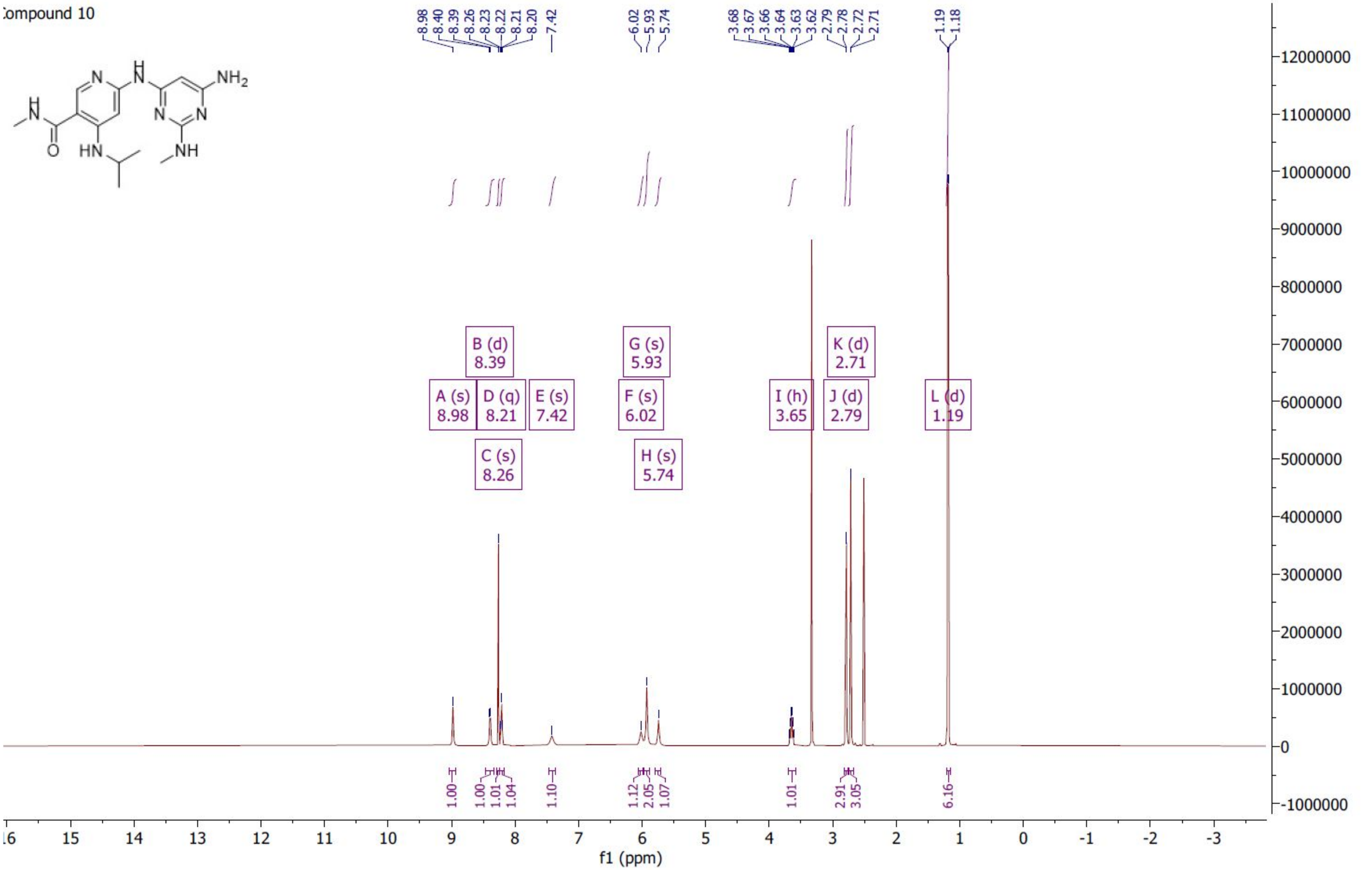

^{1}H NMR (500 MHz, DMSO) δ 8.98 (s, 1H), 8.39 (d, J = 7.5 Hz, 1H), 8.26 (s, 1H), 8.21 (q, J = 4.5 Hz, 1H), 7.42 (s, 1H), 6.02 (s, 1H), 5.93 (s, 2H), 5.74 (s, 1H), 3.65 (h, J = 6.5 Hz, 1H), 2.79 (d, J = 4.8 Hz, 3H), 2.71 (d, J = 4.4 Hz, 3H), 1.19 (d, J = 6.3 Hz, 6H).

Compound 11

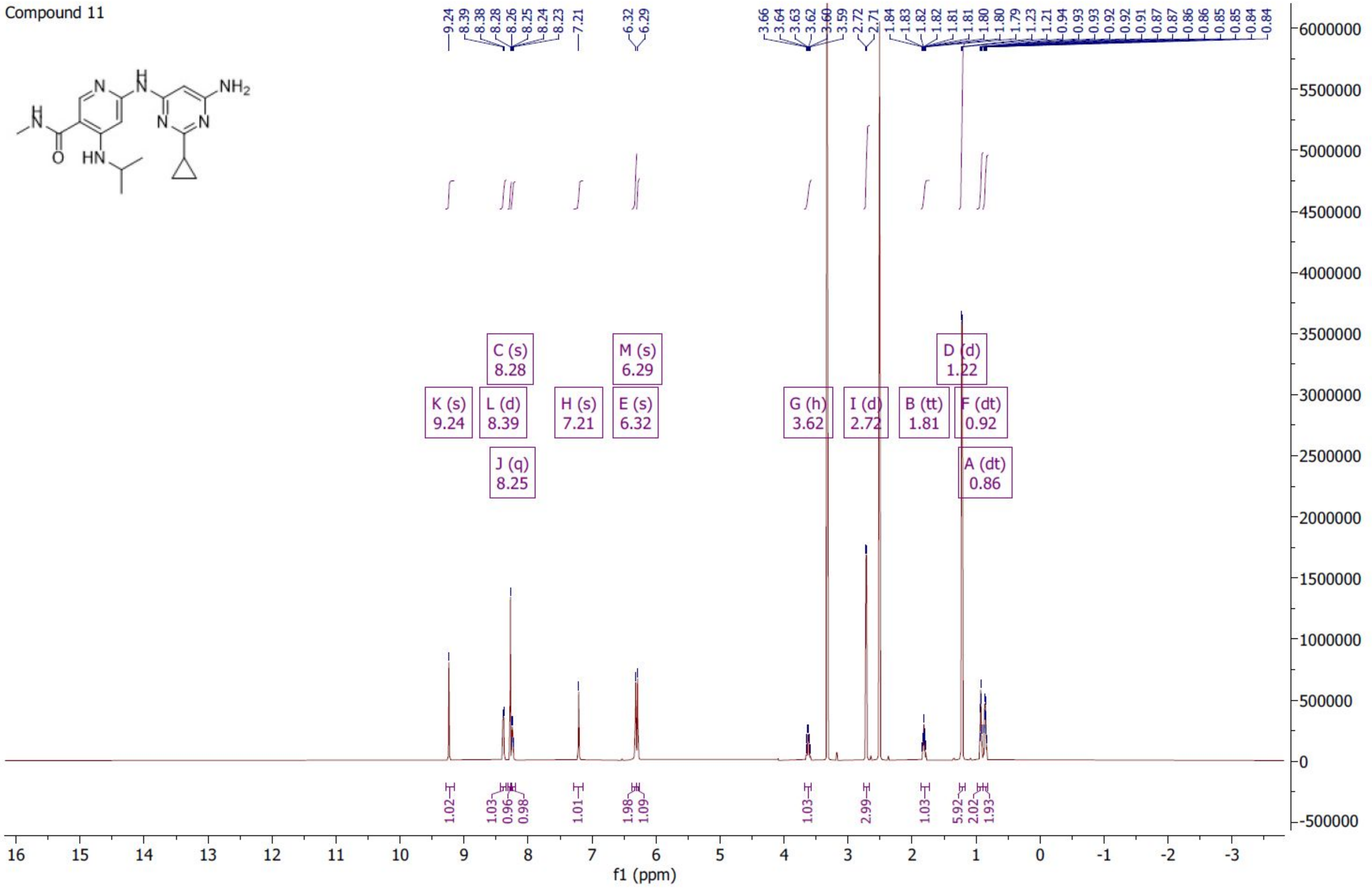

^{1}H NMR (500 MHz, DMSO) δ 9.24 (s, 1H), 8.39 (d, *J* = 7.3 Hz, 1H), 8.28 (s, 1H), 8.25 (q, *J* = 4.5 Hz, 1H), 7.21 (s, 1H), 6.32 (s, 2H), 6.29 (s, 1H), 3.62 (h, *J* = 6.5 Hz, 1H), 2.72 (d, *J* = 4.5 Hz, 3H), 1.81 (tt, *J* = 8.1, 4.7 Hz, 1H), 1.22 (d, *J* = 6.3 Hz, 6H), 0.92 (dt, *J* = 5.1, 2.8 Hz, 2H), 0.86 (dt, *J* = 8.2, 3.0 Hz, 2H).

Compound 12

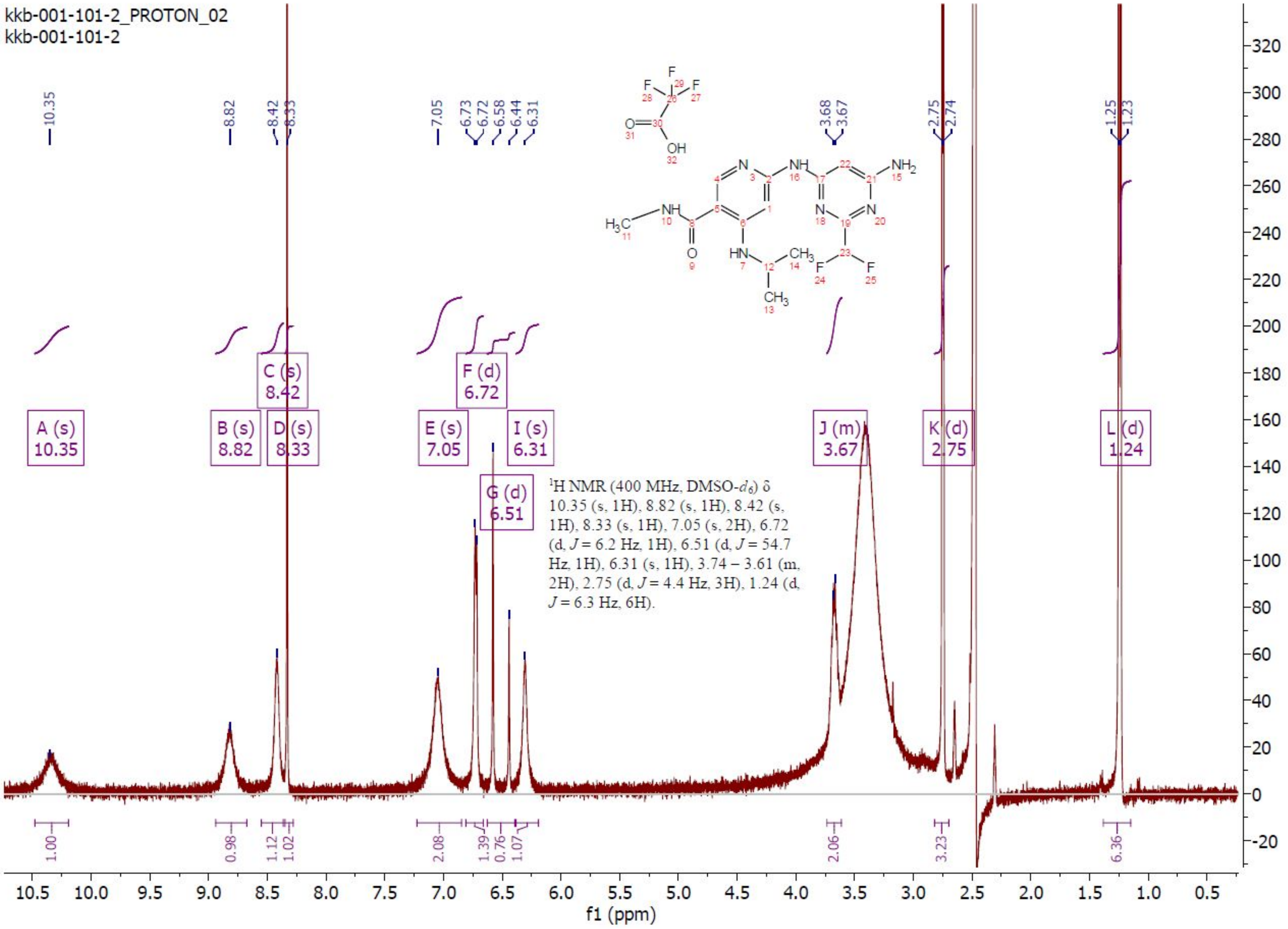
kkb-001-101-2_PROTON_02
kkb-001-101-2
A (s) 10.35
B (s) 8.82
C (s) 8.42
D (s) 8.33
E (s) 7.05
F (d) 6.72
G (d) 6.51
I (s) 6.31
J (m) 3.67
K (d) 2.75
L (d) 1.24
^{1}H NMR (400 MHz, DMSO-d_6) δ 10.35 (s, 1H), 8.82 (s, 1H), 8.42 (s, 1H), 8.33 (s, 1H), 7.05 (s, 2H), 6.72 (d, J = 6.2 Hz, 1H), 6.51 (d, J = 54.7 Hz, 1H), 6.31 (s, 1H), 3.74 – 3.61 (m, 2H), 2.75 (d, J = 4.4 Hz, 3H), 1.24 (d, J = 6.3 Hz, 6H).
f1 (ppm)

Compound 13

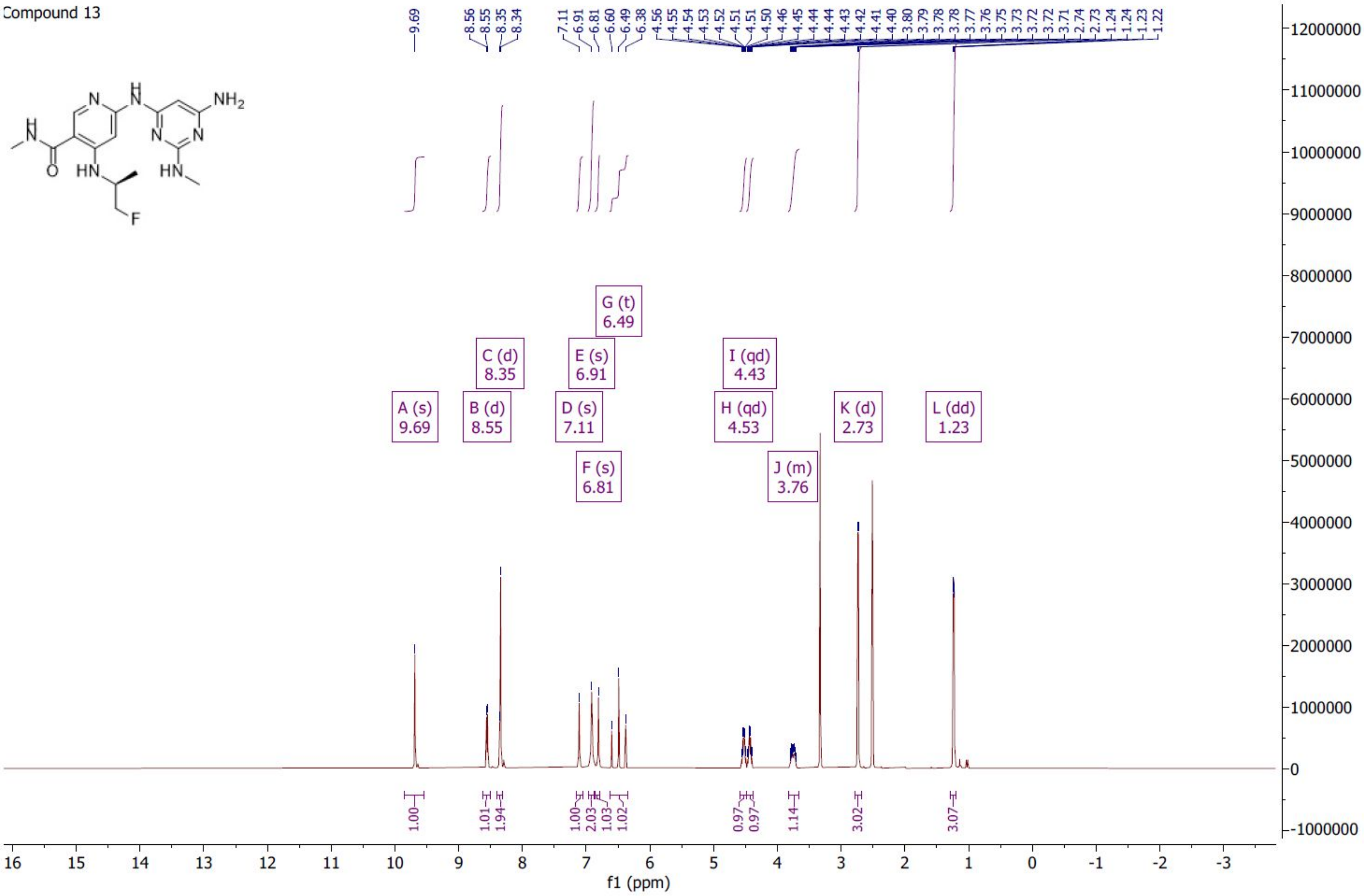

^{1}H NMR (500 MHz, DMSO) δ 9.69 (s, 1H), 8.55 (d, J = 7.6 Hz, 1H), 8.35 (d, J = 6.1 Hz, 2H), 7.11 (s, 1H), 6.91 (s, 2H), 6.81 (s, 1H), 6.49 (t, J = 54.9 Hz, 1H), 4.53 (qd, J = 9.4, 4.5 Hz, 1H), 4.43 (qd, J = 9.3, 4.5 Hz, 1H), 3.82 – 3.66 (m, 1H), 2.73 (d, J = 4.4 Hz, 3H), 1.23 (dd, J = 6.5, 1.2 Hz, 3H).

Compound 14

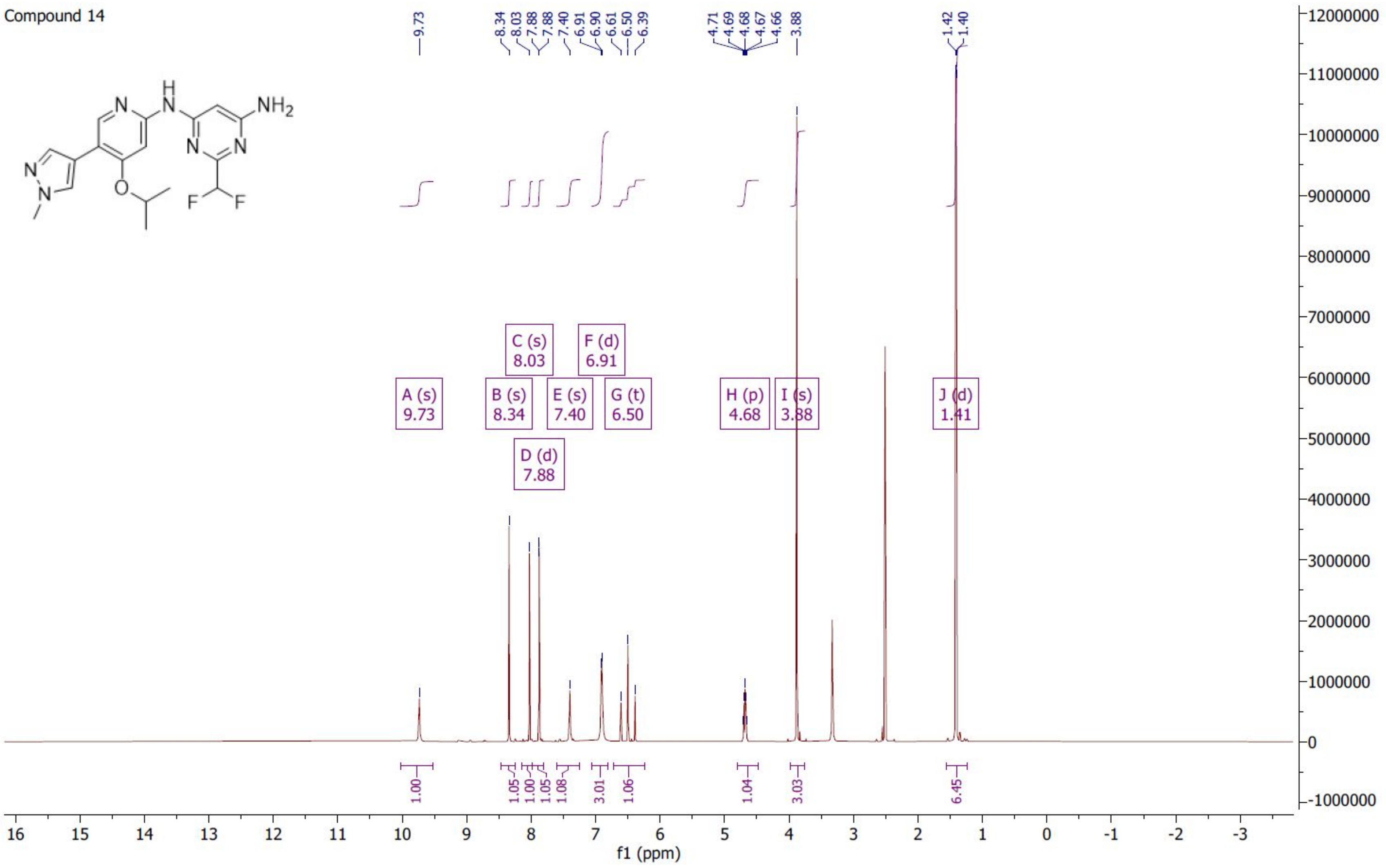

^{1}H NMR (500 MHz, DMSO) δ 9.73 (s, 1H), 8.34 (s, 1H), 8.03 (s, 1H), 7.88 (d, J = 0.8 Hz, 1H), 7.40 (s, 1H), 6.91 (d, J = 5.0 Hz, 3H), 6.50 (t, J = 54.9 Hz, 1H), 4.68 (p, J = 6.0 Hz, 1H), 3.88 (s, 3H), 1.41 (d, J = 6.0 Hz, 6H).

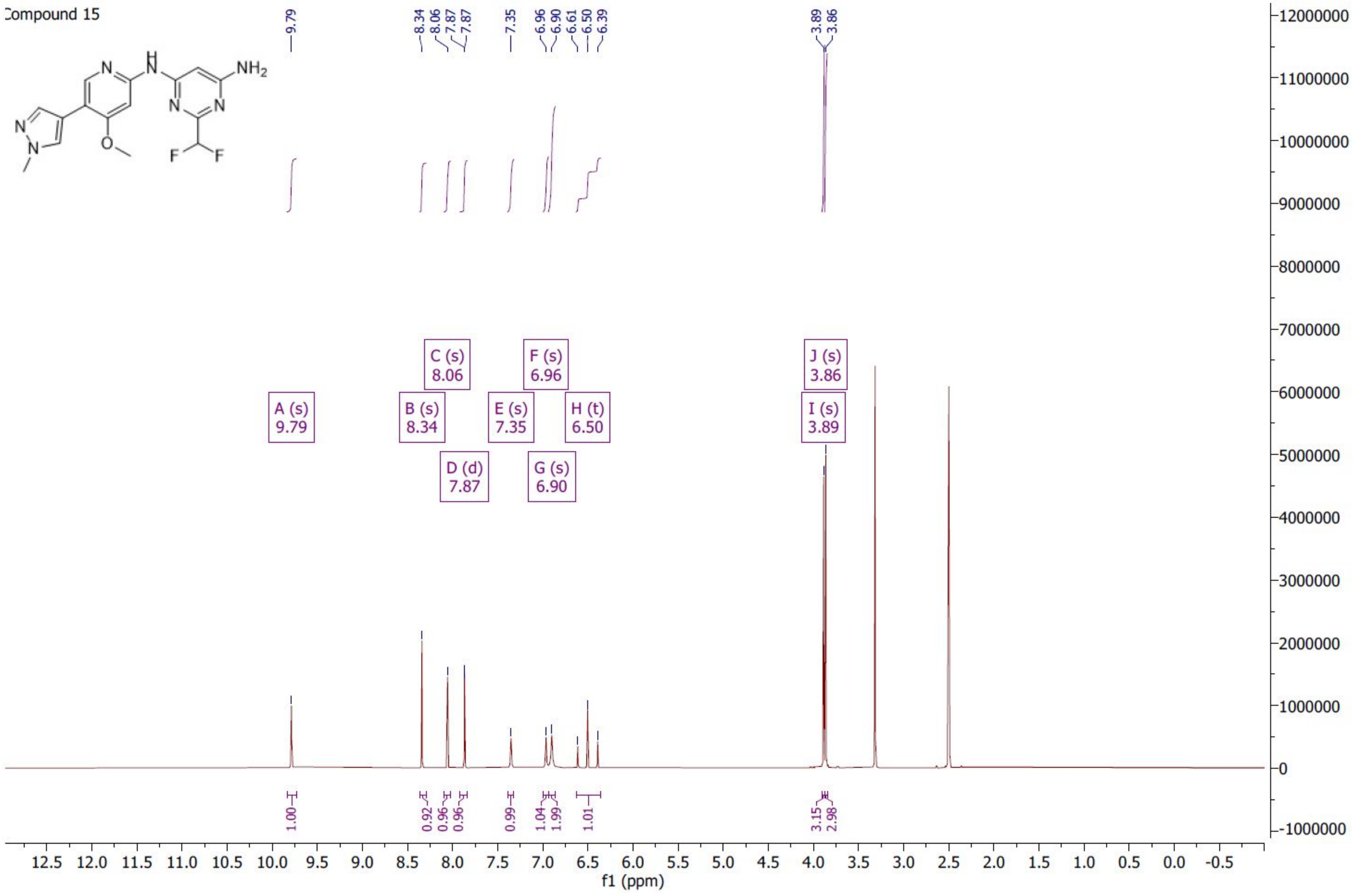

^{1}H NMR (500 MHz, DMSO) δ 9.79 (s, 1H), 8.34 (s, 1H), 8.06 (s, 1H), 7.87 (d, J = 0.8 Hz, 1H), 7.35 (s, 1H), 6.96 (s, 1H), 6.90 (s, 2H), 6.50 (t, J = 54.9 Hz, 1H), 3.89 (s, 3H), 3.86 (s, 3H).

Compound 16

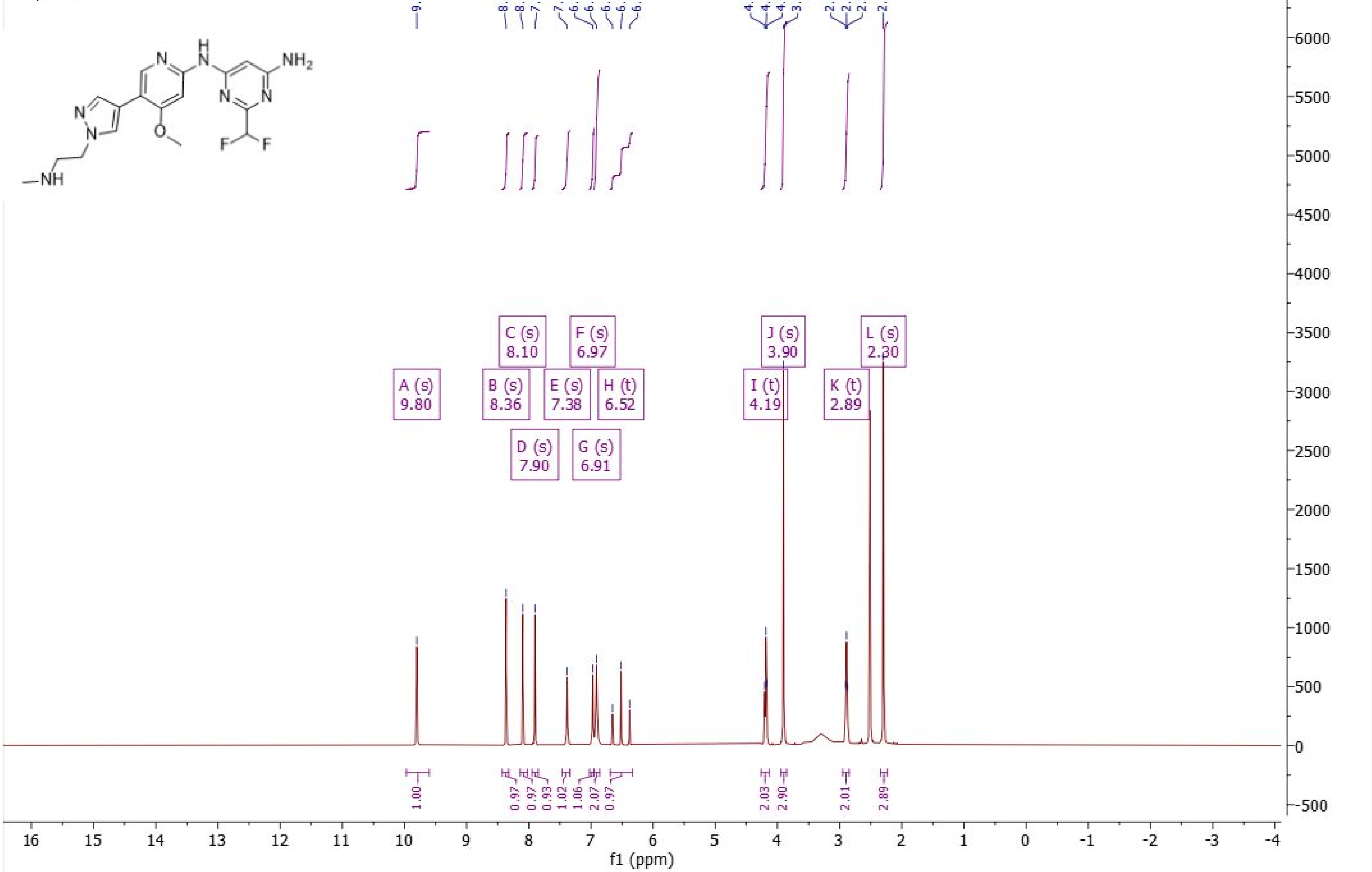

^{1}H NMR (400 MHz, DMSO) δ 9.80 (s, 1H), 8.36 (s, 1H), 8.10 (s, 1H), 7.90 (s, 1H), 7.38 (s, 1H), 6.97 (s, 1H), 6.91 (s, 2H), 6.52 (t, J = 54.8 Hz, 1H), 4.19 (t, J = 6.3 Hz, 2H), 3.90 (s, 3H), 2.89 (t, J = 6.3 Hz, 2H), 2.30 (s, 3H).

Compound 17

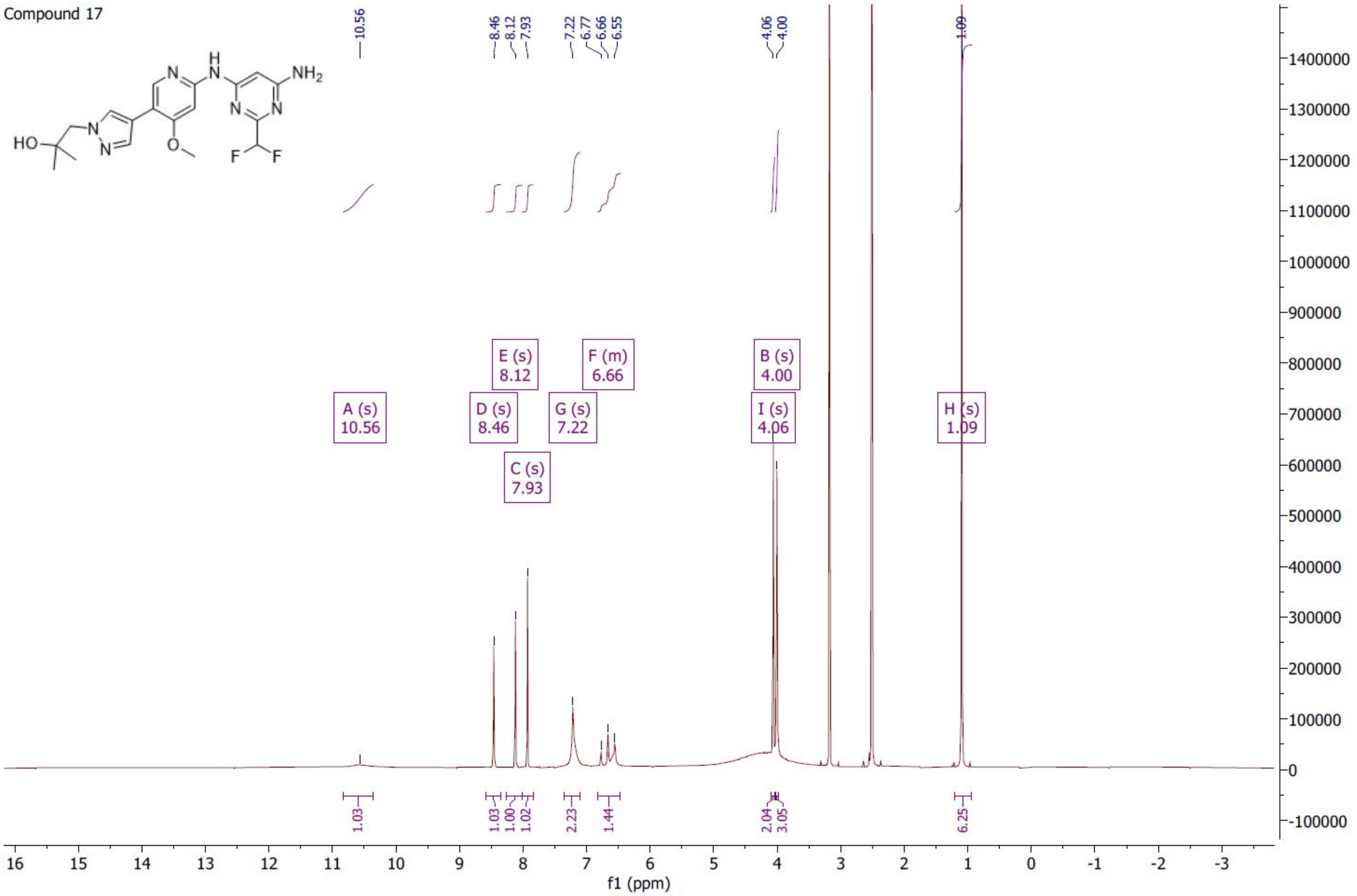

^{1}H NMR (500 MHz, DMSO) δ 10.56 (s, 1H), 8.46 (s, 1H), 8.12 (s, 1H), 7.93 (s, 1H), 7.22 (s, 2H), 6.82 – 6.47 (m, 2H), 4.06 (s, 2H), 4.00 (s, 3H), 1.09 (s, 6H).

Compound 18

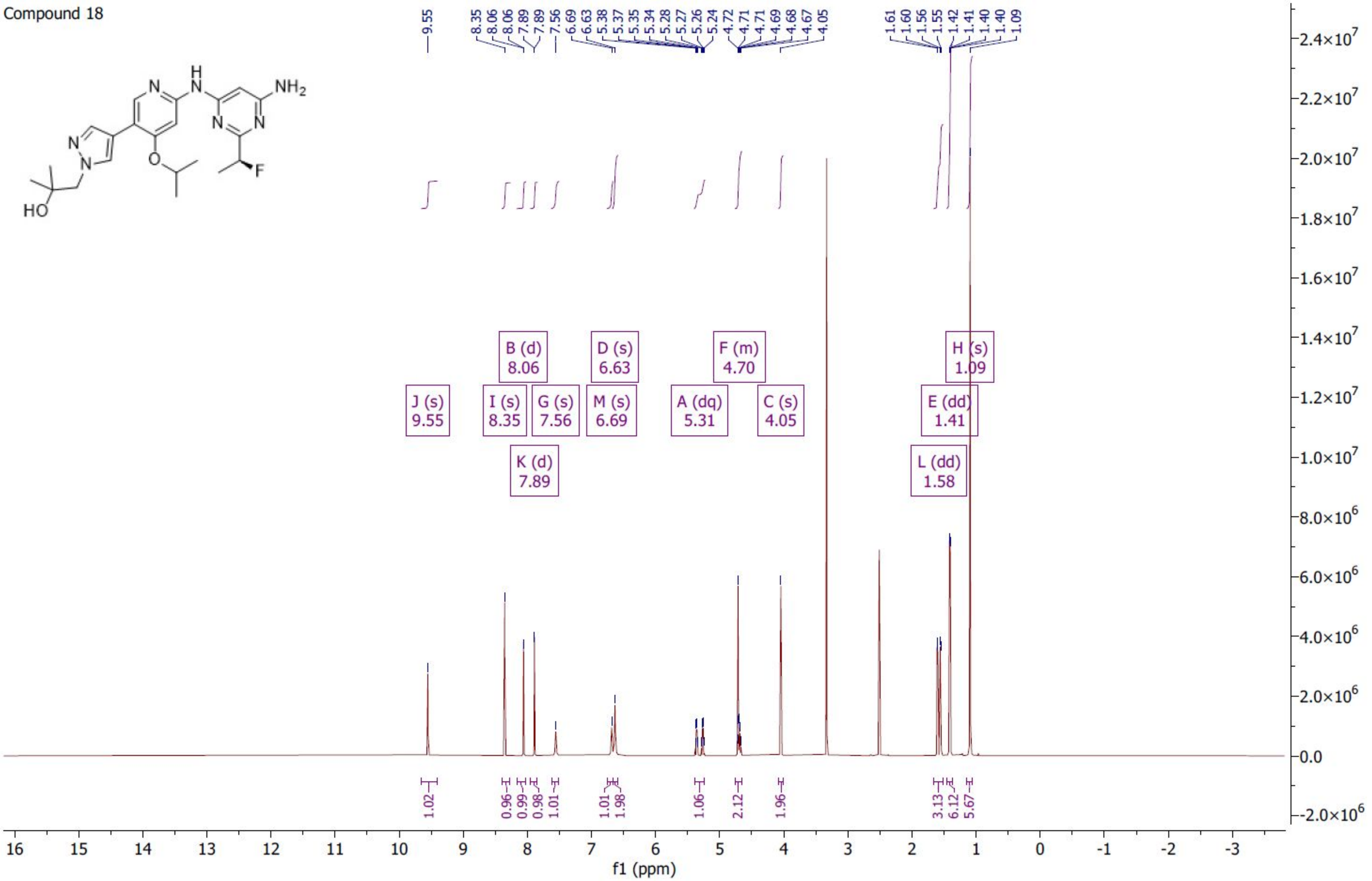

^{1}H NMR (500 MHz, DMSO) δ 9.55 (s, 1H), 8.35 (s, 1H), 8.06 (d, J = 0.8 Hz, 1H), 7.89 (d, J = 0.7 Hz, 1H), 7.56 (s, 1H), 6.69 (s, 1H), 6.63 (s, 2H), 5.31 (dq, J = 48.5, 6.5 Hz, 1H), 4.76 – 4.66 (m, 2H), 4.05 (s, 2H), 1.58 (dd, J = 24.2, 6.5 Hz, 3H), 1.41 (dd, J = 6.0, 1.5 Hz, 6H), 1.09 (s, 6H).
